# The global burden of cardiovascular diseases and type 2 diabetes attributable to low physical activity, 1990–2019: an analysis from the global burden of disease study

**DOI:** 10.3389/fcvm.2023.1247705

**Published:** 2023-12-19

**Authors:** Junhan Zhang, Zongxiang Yuan, Chuye Mo, Yiwen Kang, Fengyi Wang, Xueqin Wei, Shihui Huang, Fang Qin, Junjun Jiang, Hao Liang, Li Ye

**Affiliations:** Guangxi Key Laboratory of AIDS Prevention and Treatment, School of Public Health, Guangxi Medical University, Nanning, China

**Keywords:** cardiovascular diseases, type 2 diabetes, low physical activity, global burden of diseases, epidemiology

## Abstract

**Background:**

Cardiovascular diseases (CVD) and type 2 diabetes (T2D) account for the majority of the burden of noncommunicable disease caused by low physical activity (LPA). In order to inform future interventions, this study aims to assess the burden and trends in mortality and disability-adjusted life years (DALYs) of CVD and T2D attributable to LPA by year, location, sex, and age from 1990 to 2019.

**Methods:**

Mortality, DALYs, and their age-standardised rates (ASMR, ASDR) for CVD and T2D attributable to LPA were retrieved from Global Burden of Disease (GBD) 2019. The estimated annual percentage changes (EAPCs) were calculated using linear regression model to describe the trend over time.

**Results:**

From 1990 to 2019, the number of deaths caused by both CVD and T2D due to LPA increased significantly globally. However, the overall ASMR and ASDR for CVD declined over this same period [EAPC for ASMR (CVD) = −1.44 (95% CI: −1.50–1.38), EAPC for ASDR (CVD) = −1.30 (95% CI: −1.35 to −1.25)]. In terms of disparities, ASMR (CVD) and ASDR (CVD) in North Africa and the Middle East were consistently higher than the global average; also, the sex difference in ASMR was greatest in Central Asia. ASMR among people aged 25–44 in high Socio-Demographic Index (SDI) region has increased significantly over the past three decades. ASMR (T2D) due to LPA showed an increasing trend year by year, with EAPC = 0.26 (95% CI: 0.13–0.39), and this rate increased faster in males than in females. Consistent with cardiovascular diseases, ASMR of type 2 diabetes attributable to LPA increased among people aged 25–44, while decreased in other age groups in high SDI region.

**Conclusion:**

Interventions targeting LPA are warranted in controlling the burden of cardiovascular diseases and type 2 diabetes. Countries should adapt strategies to their local contexts, considering the sex and age differences among their populations. The 25–44 age group should be given special attention to prevent the disease burden from worsening among younger people.

## 1. Introduction

With the development of medical technologies, the improvement of living environment and the change of lifestyle, the global burden of disease has progressively transitioned from a predominance of infectious diseases to noncommunicable diseases (1). “Burden of disease” refers to the sum of challenges and losses associated with disease, including health, social aspects, and costs to society (2). Cardiovascular disease (CVD) and type 2 diabetes (T2D) are two common noncommunicable diseases caused by a combination of genes, environment and lifestyle. Cardiovascular diseases constitute the predominant cause of global mortality, claiming an estimated 17.9 million lives annually (3). In addition, an estimated 380 million people worldwide will be affected by type 2 diabetes by 2025 (4). Growing evidence indicates that diabetes and cardiovascular diseases can exacerbate each other's disease burden (5, 6), meanwhile low physical activity (LPA) has been reported to be a significant behavioral risk factor for the burden of both of these diseases.

Behavioral risk factors such as low physical activity (LPA) and high body mass index (BMI) have greatly contributed to the burden of noncommunicable disease nowadays. In 2019, 0.83 million deaths were attributed to LPA, with cardiovascular diseases and type 2 diabetes together accounting for 91.93% (7). Physical activity has been demonstrated to have a positive effect on noncommunicable disease. For example, study conducted in the elderly found that high levels of physical activity (>79.4 MET hour per week) is beneficial for reducing the risk of cardiovascular diseases (8). Additionally, previous studies indicated that replacing sedentary behavior with light physical activity proves effective in reducing risk and mortality rates of diabetes (9, 10). Besides that, a meta-analysis confirmed that achieving the World Health Organization (WHO) recommended level of physical activity was associated with a 23% reduction in the risk of cardiovascular mortality, and a 26% reduction for type 2 diabetes risk (11).

While previous studies have estimated the overall burden of diseases attributable to LPA (7) and analyzed breast cancer in particular (12). There appears to be a gap in comprehensive investigations concerning the burden of cardiovascular diseases and type 2 diabetes attributable to LPA. Notably, these two diseases bear the heaviest burden from the impact of LPA.

In this study, we used the Global Burden of Disease 2019 (GBD 2019) to conduct an in-depth analysis of the burden of cardiovascular diseases and type 2 diabetes due to LPA. Mortality, disability-adjusted life years (DALYs), and their age-standardised rates were employed to quantify the burden of disease. We provide a detailed comparison of the burden and trends of these two diseases by sex and age at the global, regional, and national levels. Our findings may offer evidence-based guidance for precision prevention and control the disease tailored to specific populations and contexts.

## 2. Materials and methods

### 2.1. Data source and definition

The data used in this study including annual numbers and age-standardised rates (ASRs) of mortality and disability-adjusted life years (DALYs) from 1990 to 2019 by sex, age, region, and country was obtained from Global Burden of Disease Study 2019 (http://ghdx.healthdata.org/gbd-results-tool), which was created by multinational collaboration. The platform provides data on 369 diseases and 87 risk factors from 204 countries and territories (13, 14). Countries and territories were further divided into 21 GBD regions according to their geographic locations and 5 Socio-demographic Index (SDI) regions (low, low-middle, middle, high-middle, and high SDI regions) based on the quintile of SDI, a comprehensive index measuring the socio-economic development level of a country or region, which combines per capita income, education level and total fertility rate (15).

Detailed diagnosis and definition of cardiovascular diseases, including ischemic heart disease (IHD) and stroke have been described in previous GBD reports (16). Type 2 diabetes was defined as fasting plasma glucose (FPG) ≥7 mmol/L or those on medication with drug or insulin (17). To assess the impact of physical inactivity on global health, GBD 2019 used two standardised questionnaires, the Global Physical Activity Questionnaire (GPAQ) and the International Physical Activity Questionnaire (IPAQ), as well as other data sources, to collect the frequency, duration and intensity of physical activity from adults over 25 years of age across different countries, years, age groups and sexes. Physical activity exposure was estimated using metabolic equivalents (MET), which is defined as the ratio of caloric consumption of an active individual to the basal metabolic rate at rest. Based on MET min per week, physical activity levels were divided into four categories: inactive (<600 MET min per week), low activity (600–3,999 MET min per week), moderate activity (4,000–7,999 MET min per week) and high activity (≥8,000 MET min per week) (18, 19). LPA was defined as <3,000 MET min per week in the GBD study (20).

### 2.2. Estimation methods

The estimation methods used for GBD 2019 are available elsewhere (13). The disease burden attributable to LPA was estimated using the comparative risk assessment established earlier. In short, the correlation between risk factors and outcomes was first determined, then exposure levels and distributions were estimated with the lowest observed exposure level as the theoretical minimum risk exposure level (TMREL). Based on the numerical input of variables such as age, sex, and year, the population attributable fraction (PAF) was calculated by a special formula (14). The PAF was then multiplied by the total burden of disease to obtain the LPA-specific disease burden. All estimates were presented with 95% Uncertainty Intervals (UIs), which were the 25th and 975th ordered values of 1,000 draws.

### 2.3. Statistical analyses

The ASR (per 100,000 population) was calculated with the GBD world population standard (21). To evaluate the magnitude and trend of ASR of mortality and DALY attributable to LPA for cardiovascular diseases and type 2 diabetes over time, the estimated annual percentage change (EAPC) and corresponding 95% Confidence Interval (CI) were calculated with the linear regression model reported by Hankey et al. (22). Positive EAPC represents an upward trend for ASR, and negative EAPC represents a downward trend. The percent change in death cases from 1990 to 2019 was obtained by the formula: (number of deaths in 2019—number of deaths 1990)/the number of deaths in 1990. The percentage change in mortality rate was calculated in the similar way. Spearman rank correlation analysis was used to evaluate the correlation strength and direction of SDI with ASR (including ASMR and ASDR). In addition, in this study, age groups were reclassified into five groups (25–44, 45–59, 60–74, 75–94, and 95+ years) to reflect the changes in disease burden at different life stages (youth, middle-aged, middle-old aged, old aged, the longevous).

All statistical analyses were performed using R software (Version R-4.2.3), and image presentations were performed using R software and GraphPad Prism (Version 8.3.0).

## 3. Results

### 3.1. Cardiovascular diseases

#### 3.1.1. Overall burden and trends of cardiovascular diseases attributable to LPA

The absolute number of deaths caused by cardiovascular disease has risen globally, but the age-standardised rates of mortality and disability have declined

**Figure 1:**
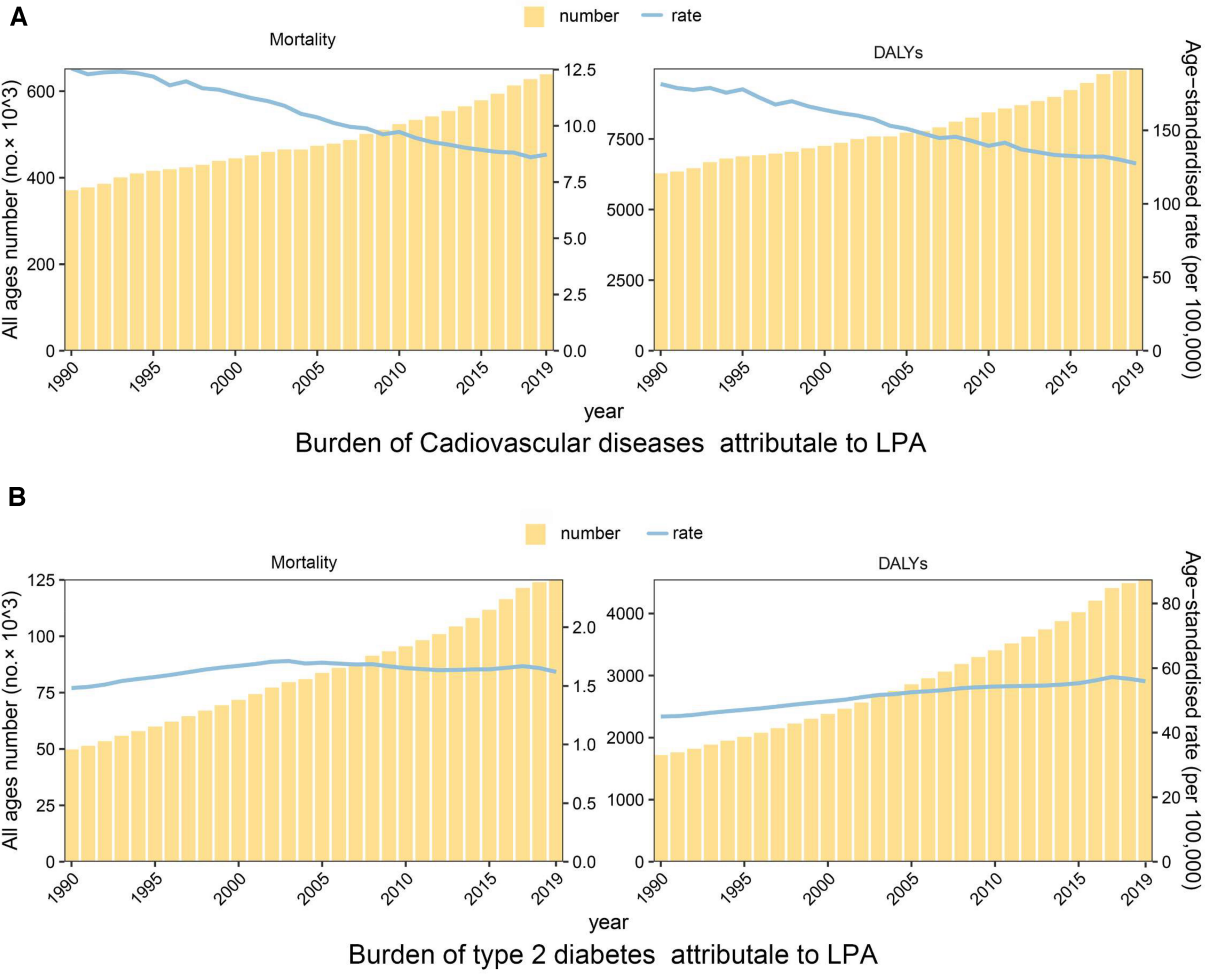
Temporal trend of the disease burden of cardiovascular diseases and type 2 diabetes attributable to LPA, 1990–2019. (**A**) Mortality and DALYs of cardiovascular diseases. (**B**) Mortality and DALYs of type 2 diabetes.

From 1990 to 2019, the number of deaths caused by CVD due to LPA has increased significantly worldwide. In 2019, there were 639.17 thousand deaths (95% UI: 272.01–1,216.53), which is about 1.7 times the number of deaths in 1990. Nevertheless, the overall mortality rate has been decreasing [EAPC = −1.44 (95% CI: −1.50 to −1.38)], with a global ASMR of 12.55 in 1990 (95% UI: 5.12–24.23) and 8.60 in 2019 (95% UI: 3.68–16.28) (Figure 1A, Table 1). The corresponding proportions were 6.52 (95% UI: 2.36–13.31) for IHD, and 2.08 (95% UI: 0.41–5.33) for stroke (Supplementary Tables S1, S2). Similar to mortality, there was an overall annual decrease in DALY rates related to CVD attributable to LPA [EAPC = −1.30 (95% CI: −1.35 to −1.25)] across the years. While the ASDRs decreased at a slower rate than ASMRs [EAPC = −1.44 (95% CI: −1.50 to −1.38)] (Figure 1A, Table 1).

**Table 1:** DALYs and mortality of cardiovascular diseases attributable to LPA in 27 GBD regions in 1990 and 2019.

Characteristics	DALYs (95% UI)					Mortality (95% UI)				
	1990		2019		EAPC	1990		2019		EAPC
	Number (no. ×10^3^)	ASR per 100,000	Number (no. ×10^3^)	ASR per 100,000		Number (no. ×10^3^)	ASR per 100,000	Number (no. ×10^3^)	ASR per 100,000	
Global	6,282.52 (2,334.97–13,255.09)	181.64 (71.59–374.01)	9,996.08 (4,130.11–20,323.34)	127.52 (53.07–256.55)	−1.30 (−1.35 to −1.25)	371.04 (147.62–740.49)	12.55 (5.12–24.23)	639.17 (272.01–1,216.53)	8.60 (3.68–16.28)	−1.44 (−1.50 to −1.38)
Sex										
Males	2,925.51 (931.02–6,625.10)	189.29 (63.56–416.06)	4,875.35 (1,759.62–10,554.52)	137.97 (51.37–297.15)	−1.19 (−1.24 to −1.14)	151.55 (52.04–326.48)	12.37 (4.34–26.54)	276.54 (106.00–579.77)	8.88 (3.48–18.23)	−1.27 (−1.32 to −1.22)
Females	3,357.02 (1,400.73–6,630.04)	171.74 (72.88–333.55)	5,120.73 (2,304.98–9,564.41)	116.83 (52.54–218.39)	−1.38 (−1.44 to −1.33)	219.50 (94.67–406.82)	12.38 (5.31–22.47)	362.64 (164.75–645.83)	8.24 (3.75–14.69)	−1.53 (−1.60 to −1.46)
Location										
Low SDI	274.41 (98.16–607.12)	135.89 (55.04–288.03)	548.78 (208.49–1,207.54)	121.16 (50.37–250.85)	−0.19 (−0.29 to −0.09)	12.59 (5.14–26.53)	8.20 (3.57–16.11)	27.25 (11.57–55.67)	7.57 (3.40–14.70)	−0.10 (−0.20–0.00)
Low-middle SDI	839.68 (341.48–1,791.85)	166.24 (72.72–332.32)	1,805.92 (770.39–3,678.28)	149.18 (66.98–297.34)	−0.22 (−0.29 to −0.16)	40.23 (17.81–79.79)	10.45 (4.82–19.57)	100.59 (46.34–191.61)	9.79 (4.59–18.04)	−0.15 (−0.22 to −0.07)
Middle SDI	1,421.78 (552.26–3,044.03)	166.56 (68.64–341.96)	3,294.77 (1,344.45–6,944.41)	150.93 (62.57–310.60)	−0.34 (−0.39 to −0.28)	72.65 (30.25–147.04)	10.92 (4.73–21.32)	194.91 (82.24–384.85)	10.33 (4.49–19.58)	−0.19 (−0.25 to −0.12)
High-middle SDI	1,964.08 (759.34–4,141.25)	211.98 (84.64–430.41)	2,791.23 (1,175.77–5,461.18)	140.39 (59.39–274.78)	−1.59 (−1.70 to −1.47)	121.12 (49.72–238.36)	15.26 (6.49–29.41)	199.97 (85.79–365.65)	10.40 (4.49–18.96)	−1.46 (−1.57 to −1.34)
High SDI	1,778.15 (591.50–3,743.66)	169.67 (56.06–357.65)	1,547.31 (591.46–3,097.46)	78.52 (30.01–161.50)	−3.06 (−3.25 to −2.87)	124.20 (43.40–247.10)	12.09 (4.24–24.02)	115.98 (45.33–219.52)	5.04 (1.93–9.75)	−3.47 (−3.64 to −3.30)
Central Asia	88.90 (33.91–189.84)	216.60 (83.11–455.68)	145.95 (53.87–328.43)	262.11 (102.93–556.53)	0.24 (−0.14–0.62)	5.91 (2.37–12.05)	15.78 (6.50–31.84)	9.11 (3.67–18.77)	19.67 (8.24–39.29)	0.37 (0.00–0.74)
Central Europe	319.50 (124.96–692.09)	239.68 (96.18–510.96)	314.77 (134.83–620.01)	140.97 (60.21–288.46)	−2.19 (−2.31 to −2.07)	20.66 (8.53–42.75)	17.44 (7.42–34.70)	25.06 (11.18–47.68)	11.14 (4.95–21.18)	−1.88 (−1.99 to −1.77)
Eastern Europe	595.22 (240.75–1,301.24)	240.34 (98.65–526.89)	725.60 (301.03–1,489.93)	203.85 (85.15–425.82)	−0.78 (−1.18 to −0.39)	40.73 (17.01–83.67)	18.53 (7.80–36.59)	57.00 (24.75–108.46)	16.20 (7.09–30.76)	−0.65 (−1.03 to −0.26)
Australasia	47.34 (15.59–100.58)	208.37 (69.44–441.04)	44.35 (17.02–83.68)	82.99 (30.47–160.10)	−3.65 (−3.82 to −3.48)	3.21 (1.13–6.37)	15.01 (5.33–29.48)	3.74 (1.51–6.63)	6.36 (2.60–11.49)	−3.54 (−3.72 to −3.35)
High-income Asia Pacific	201.82 (64.21–447.30)	111.90 (36.37–244.67)	216.25 (74.21–461.37)	40.68 (13.90–89.90)	−3.59 (−3.77 to −3.42)	13.12 (4.55–27.65)	8.20 (2.93–17.16)	18.00 (6.37–36.45)	2.78 (0.98–5.68)	−3.87 (−4.08 to −3.66)
High-income North America	573.17 (173.26–1,286.88)	158.25 (47.05–362.23)	430.18 (128.45–1,005.31)	66.43 (19.29–158.41)	−3.38 (−3.60 to −3.16)	40.41 (12.42–84.84)	10.90 (3.39–22.98)	31.50 (9.91–69.39)	4.33 (1.33–9.71)	−3.65 (−3.95 to −3.34)
Southern Latin America	21.80 (6.93–58.97)	50.98 (16.24–136.69)	25.04 (8.63–61.41)	29.61 (10.19–72.23)	−1.63 (−1.84 to −1.43)	1.35 (0.43–3.54)	3.49 (1.10–8.97)	1.76 (0.60–4.06)	2.04 (0.69–4.71)	−1.60 (−1.85 to −1.35)
Western Europe	1,116.77 (383.73–2,260.11)	188.55 (65.10–382.79)	807.10 (305.56–1,517.06)	76.51 (27.70–153.85)	−3.66 (−3.92 to −3.40)	80.58 (29.73–154.83)	13.78 (5.08–26.19)	70.67 (28.15–127.13)	5.91 (2.35–10.85)	−3.41 (−3.62 to −3.19)
Andean Latin America	13.80 (3.70–35.04)	73.65 (19.82–185.04)	30.42 (8.63–70.77)	56.06 (16.12–129.15)	−0.89 (−1.11 to −0.68)	0.82 (0.22–2.00)	4.96 (1.39–11.87)	2.01 (0.60–4.54)	3.85 (1.17–8.69)	−0.79 (−0.99 to −0.60)
Caribbean	67.70 (25.46–132.16)	275.47 (104.27–527.98)	114.74 (45.82–220.27)	222.40 (88.81–426.66)	−0.73 (−0.93 to −0.54)	4.12 (1.64–7.51)	18.43 (7.62–33.16)	7.24 (3.22–12.77)	13.91 (6.20–24.54)	−1.00 (−1.19 to −0.81)
Central Latin America	57.47 (16.94–141.74)	75.61 (22.63–184.23)	140.26 (40.64–337.36)	61.72 (18.16–148.46)	−0.92 (−1.14 to −0.70)	3.21 (0.96–7.71)	5.01 (1.53–11.81)	9.38 (2.91–21.57)	4.28 (1.33–9.80)	−0.88 (−1.13 to −0.64)
Tropical Latin America	358.33 (149.76–633.59)	424.66 (188.35–730.73)	542.33 (269.40–878.67)	229.60 (116.10–370.11)	−2.02 (−2.11 to −1.93)	17.25 (7.90–28.89)	25.43 (12.25–40.36)	30.50 (16.84–46.67)	13.50 (7.45–20.55)	−2.07 (−2.20 to −1.94)
North Africa and Middle East	958.67 (415.16–1,823.78)	618.90 (290.34–1,116.85)	1,984.20 (923.06–3,607.54)	493.78 (241.44–856.21)	−0.90 (−0.96 to −0.84)	45.86 (22.04–81.18)	36.57 (18.63–61.57)	100.04 (51.42–168.30)	29.73 (15.72–48.05)	−0.80 (−0.85 to −0.74)
South Asia	750.16 (260.23–1,736.41)	161.58 (62.08–341.88)	1,629.94 (607.61–3,594.94)	135.30 (54.09–282.99)	−0.24 (−0.42 to −0.05)	34.87 (13.36–74.46)	10.16 (4.18–20.09)	93.60 (38.45–188.87)	9.28 (3.97–18.40)	−0.16 (−0.36–0.05)
East Asia	728.33 (269.40–1,661.80)	113.66 (43.25–239.45)	1,917.27 (713.67–4,107.17)	110.68 (42.79–231.46)	−0.10 (−0.23–0.03)	39.97 (15.14–84.56)	8.22 (3.25–16.07)	130.52 (49.62–262.60)	8.66 (3.44–17.06)	0.26 (0.07–0.45)
Oceania	4.85 (1.47–12.36)	188.79 (64.42–440.83)	13.27 (4.13–33.00)	213.59 (76.01–490.45)	0.40 (0.30–0.51)	0.20 (0.06–0.47)	10.82 (4.12–22.87)	0.56 (0.19–1.31)	12.39 (4.89–25.88)	0.44 (0.37–0.51)
Southeast Asia	225.94 (72.36–548.65)	104.05 (34.20–245.88)	598.33 (196.91–1,357.63)	111.46 (38.29–252.21)	0.22 (0.14–0.30)	11.41 (3.82–27.37)	6.56 (2.24–14.62)	32.20 (11.27–70.49)	7.04 (2.50–15.04)	0.22 (0.12–0.33)
Central Sub-Saharan Africa	24.25 (7.88–57.30)	129.67 (46.96–293.43)	55.30 (18.54–132.46)	127.57 (47.27–287.73)	−0.14 (−0.20 to −0.08)	1.05 (0.37–2.44)	7.75 (2.99–16.78)	2.67 (0.98–5.84)	8.08 (3.20–16.96)	0.07 (0.02–0.13)
Eastern Sub-Saharan Africa	23.86 (7.55–70.93)	37.07 (12.47–101.15)	50.42 (16.88–146.66)	36.56 (12.78–98.39)	−0.06 (−0.08 to −0.04)	1.06 (0.36–2.89)	2.18 (0.77–5.62)	2.47 (0.87–6.61)	2.28 (0.81–5.68)	0.18 (0.15–0.21)
Southern Sub-Saharan Africa	31.21 (11.69–66.33)	119.32 (46.50–242.73)	60.87 (23.71–127.85)	123.47 (50.02–252.87)	0.44 (−0.14–1.03)	1.50 (0.62–2.99)	6.95 (2.95–13.61)	3.36 (1.42–6.70)	8.19 (3.51–15.91)	0.94 (0.38–1.51)
Western Sub-Saharan Africa	73.41 (23.55–171.35)	98.34 (33.43–228.88)	149.51 (50.02–350.95)	94.70 (34.02–217.72)	−0.20 (−0.29 to −0.12)	3.76 (1.30–8.63)	6.27 (2.37–13.82)	7.79 (2.83–17.64)	6.15 (2.32–13.55)	−0.13 (−0.19 to −0.06)

#### 3.1.2. Impact of LPA on cardiovascular diseases based on SDI and geographical regions

The burden of cardiovascular diseases attributable to LPA varied considerably across 5 SDI regions, but mortality rates have been declining across all regions

**Figure 2:**
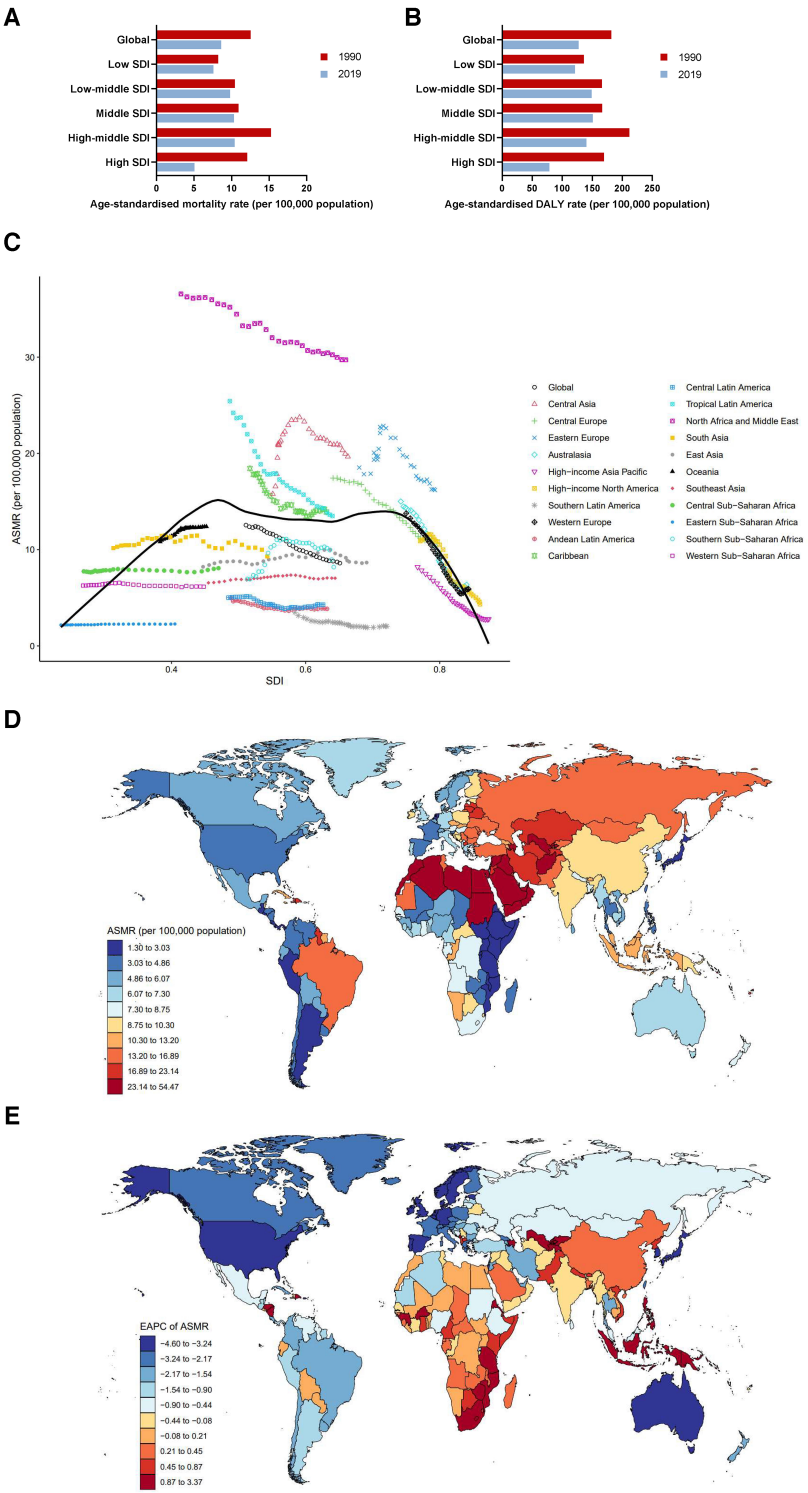
Burden of cardiovascular diseases attributable to LPA by location. (**A**) ASMRs globally and in territories with low to high SDIs in 1990 and 2019. (**B**) ASDRs globally and in territories with low to high SDIs in 1990 and 2019. (**C**) ASMRs across 21 GBD regions by SDI, 1990–2019. (**D**) ASMRs in 204 countries and territories by geographical region in 2019. (**E**) EAPCs of ASMRs in 204 countries and territories from 1990 to 2019.

Burden of cardiovascular diseases attributable to LPA varied considerably across the 5 SDI regions. The highest number of deaths and mortality rates were observed in high-middle SDI region in 2019, with an ASMR (CVD) of 10.40 (95% UI: 4.49–18.96). The death number in middle SDI region was second highest, reaching 194.91 (95% UI: 82.24–384.85) thousand. The lowest death number was observed in low SDI region [27.25 (95% UI: 11.57–55.67) thousand]. Whereas the lowest ASMR (CVD) was in high SDI region [5.04 (95% UI: 1.93–9.75)]. Mortality rates have been declining annually in all regions, particularly in high SDI region, ASMR (CVD) dropped by an average of −3.47 (95% CI: −3.64 to −3.30) from 1990 to 2019 (Figure 2A, Table 1). As for DALYs, the ASDR (CVD) in middle SDI region was highest [150.93 (95% UI: 62.57–310.60)] in 2019, followed by low-middle SDI region [149.18 (95% UI: 66.98–297.34)] (Figure 2B, Table 1).

Figure 2C demonstrates the changes in ASMRs (CVD) across 21 GBD regions ranging from lowest to highest SDI over a 30-year period. In regions with SDI <0.5, ASMRs (CVD) remained low and relatively stable from 1990 to 2019. However, it showed a significant declining trend along with a gradual improvement of SDI. High-income Asia Pacific had the fastest rate of decline, with EAPC = −3.87 (95% CI: −4.08 to −3.66). It is worth noting that although ASMRs (CVD) in North Africa and the Middle East decreased year-over-year, they were still far above the global average [29.73 (95% UI: 15.72–48.05) in 2019]. In addition, ASMRs (CVD) in Central Asia [19.67 (95% UI: 8.24–39.29) in 2019], Eastern Europe [16.20 (95% UI: 7.09–30.76) in 2019], Caribbean [13.91 (95% UI:6.20–24.54) in 2019], Tropical Latin America [13.50 (95% UI: 7.45–20.55) in 2019], and Central Europe [11.14 (95% UI:4.95–21.18) in 2019] were all higher than the global average throughout the study period (Table 1). The IHD-related ASMRs were consistent with total cardiovascular diseases (Supplementary Figure S5, Table S1), while stroke-related ASMRs were highest in Tropical Latin America among 21 GBD regions from 1990 to 1996, followed by Eastern Europe, North Africa, and the Middle East. As ASMR (stroke) in Eastern Europe continued to rise, it surpassed Tropical Latin America in 1997 to become the world's number one, maintaining the position until 2005. Unlike Eastern Europe and Tropical Latin America, ASMR (stroke) in North Africa and the Middle East has remained almost unchanged during the past 3 decades and has invariably ranked as the highest since 2005 (Supplementary Figure S6A, Table S2). The DALYs (stroke) in 21 GBD regions were similar to that in mortality (Supplementary Figure S6B, Table S2).

At the national level, India had the highest number of deaths in 2019, with a total of 72.63 (95% UI: 29.52–146.98) thousand deaths, and the ASMR (CVD) was 8.95 (95% UI: 3.78–17.30). Russian Federation suffered the second highest number of deaths [38.17 (95% UI: 17.12–73.05) thousand], followed by Brazil [30.23 (95% UI: 16.77–46.33) thousand]. Sudan [54.47 (95% UI: 33.18–81.52) thousand], Oman [49.40 (95% UI: 27.21–76.03) thousand], and Syrian Arab Republic [48.17 (95% UI: 24.45–79.90) thousand] had the three highest ASMRs (CVD) of all countries in 2019. On the contrary, the lowest ASMRs (CVD) were found in Guatemala [1.30 (95% UI: 0.43–4.18)], Argentina [1.34 (95% UI: 0.45–3.68)], and Kenya [1.72 (95% UI: 0.61–4.43)]. Despite a downward trend in global ASMR (CVD) from 1990 to 2019, some countries showed an upward trend, particularly Uzbekistan [EAPC = 3.37 (95% CI: 2.81–3.94)], Tajikistan [EAPC = 2.99 (95% CI: 2.64–3.33)], and Azerbaijan [EAPC = 2.29 (95% CI: 2.07–2.51)]. Instead, countries with the largest decreasing EAPC trends in ASMRs (CVD) over the past decades included Republic of Korea [EAPC = −4.60 (95% CI: −4.81 to −4.39)], Israel [EAPC = −4.42 (95% CI: −4.66 to −4.19)], and Portugal [EAPC = −4.38 (95% CI: −4.79 to −3.97)] (Figures 2C–E, Supplementary Table S3).

An extremely high ASDR (CVD), much higher than that in any other country, was observed in Sudan [979.12 (95% UI: 556.55–1,534.28)], followed by Saudi Arabia [790.15 (95% UI: 457.57–1,212.79)], and Egypt [774.32 (95% UI: 345.95–1,418.98)]. In line with ASMRs (CVD), Guatemala, Argentina and Kenya also had the lowest ASDRs (CVD) among the 204 countries and territories (Supplementary Figures S1B–D, Table S3). Data for IHD and stroke are shown in Supplementary Tables S4, S5, respectively. Pictorial presentations of the data are shown in Supplementary Figures S5C, S6C.

#### 3.1.3. Sex differences in cardiovascular diseases burden due to LPA

Males have higher ASMR (CVD) due to LPA than females, but the time trends of ASMR (CVD) decreased in both sexes, with a greater reduction in females (−33.40%) than in males (−28.24%)

**Figure 3:**
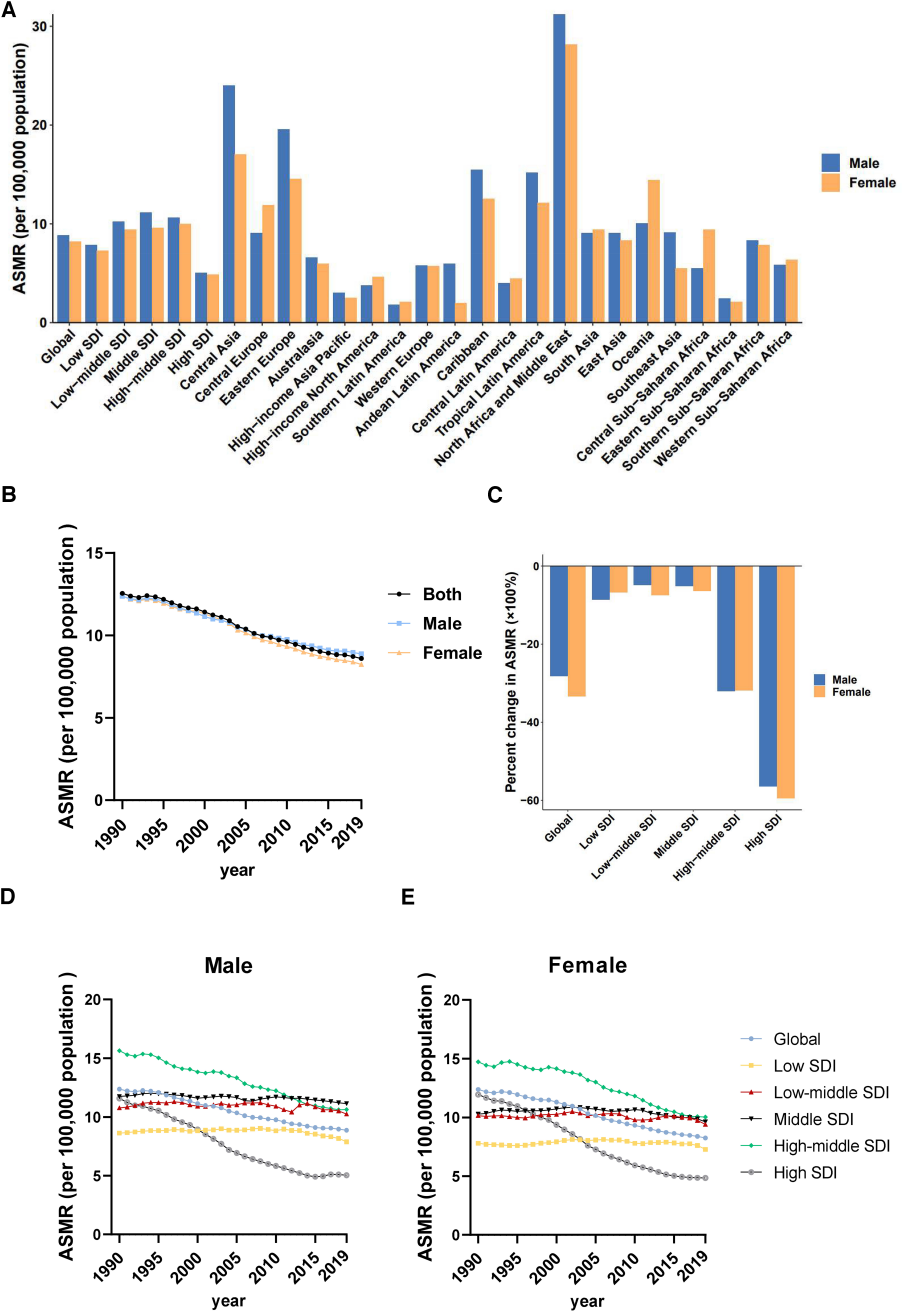
Sex differences and trends of cardiovascular diseases mortality rates attributable to LPA in different regions. (**A**) ASMRs in males and females globally, in territories with low to high SDIs and in 21 GBD regions in 2019. (**B**) Temporal trend of ASMRs globally by sex, 1990–2019. (**C**) Percent changes in ASMRs in males and females between 1990 and 2019. (**D**) Temporal trend of ASMRs in males by SDI, 1990–2019. (**E**) Temporal trend of ASMRs in females by SDI, 1990–2019.

In 2019, death cases of cardiovascular diseases attributable to LPA in males was 276.54 (95% UI: 106.00–579.77) thousand, with an ASMR (CVD) of 8.88 (95% UI: 3.48–18.23). Death cases of females [362.64 (95% UI: 164.75–645.83) thousand] was higher than males, but ASMR [8.24 (95% UI: 3.75–14.69)] (Table 1) was lower than males. From 1990 to 2019, the ASMR (CVD) of cardiovascular diseases attributable to LPA decreased globally in both sexes, with a greater percent change in females (−33.40%) than in males (−28.24%). However, the decline was mainly attributed to improvements in high-middle and high SDI regions. Notably, despite a significant decreasing trend in ASMR (CVD), the highest ASMR (CVD) in females had persistently occurred in high-middle SDI region over the past three decades. Meanwhile, high-middle SDI region also had the highest ASMR (CVD) in males before 2013 (Figures 3B–E). Amongst the 21 GBD regions, North Africa and the Middle East had the highest ASMRs (CVD) in both sexes (males: 31.23 [95% UI: 15.74–51.74], females: 28.15 [95% UI: 15.62–44.52]), while the lowest ASMRs (CVD) for males and females were in Southern Latin America and Andean Latin America, respectively. The largest difference in ASMRs (CVD) between the two sexes was found in Central Asia (Figure 3A).

Similarly, at the global level, males had higher ASDR (CVD) than females, and a larger reduction in DALYs was observed in females than in males from 1990 to 2019. The percent change of ASDR (CVD) from 1990 to 2019 showed that high SDI region had the fastest decrease, both in males (−51.80%) and females (−55.62%), while middle SDI and low-middle SDI regions had a relatively slow decline in ASDRs (CVD). Moreover, in 2019, middle SDI and low-middle SDI regions had the highest ASDRs (CVD) in males and in females, respectively (Table 1, Supplementary Figure S2).

#### 3.1.4. Burden and changes of cardiovascular diseases attributable to LPA at different age groups

The absolute number of deaths due to CVD rose in each age group; however, time trends of ASMRs (CVD) attributable to LPA generally improved across age groups, although they worsened in those aged 25–44 years in the high SDI regions

**Figure 4:**
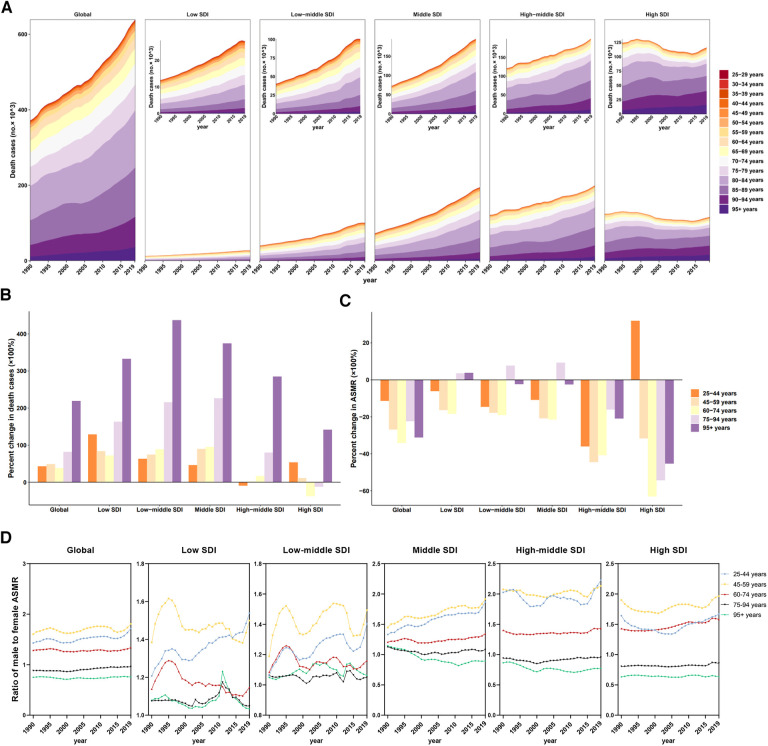
Mortality of cardiovascular disease attributable to LPA by age groups in regions with different SDI. (**A**) Contribution of each age group to total deaths over the period from 1990 to 2019 by SDI region. (**B**) Percent changes in deaths in five age groups between 1990 and 2019 by SDI region. (**C**) Percent changes in ASMR in five age groups between 1990 and 2019 by SDI region. (**D**) Ratio of males to females ASMRs by age group and by SDI region, 1990–2019.

In age-specific analysis, the number of deaths increased with age, particularly in the 80–84 age group, which accounted for the majority of deaths. In 2019, the 80–84 age group had the highest death cases across low, low-middle, middle, and high-middle SDI regions, while the 85–89 age group had the highest death cases in high SDI region (Figure 4A). However, the rise in deaths across all age groups exhibited a swifter pace in regions with lower SDI between 1990 and 2019. In addition, analysis of the redefined five age groups revealed that globally, the total number of deaths in all age groups showed increases over the 30-year study period (1990–2019), with the largest increase occurred in the 95+ age group (218.86%), followed by the 75–94 age group (Figure 4B). Remarkably, percent change of death case in 25–44 age group were increased in all regions except high-middle SDI region. Particularly alarming is the severe situation in both low and high SDI regions. In low SDI region, the percent change of death cases in the 25–44 age group reached a staggering 128.65%. Meanwhile, in the high SDI region, the percent change of death cases in the 25–44 age group exceeded that of the 45–94 age group (Figure 4B).

Additionally, the ASMRs (CVD) and ASDRs (CVD) experienced a full-scale decline at global level in all age groups. However, a notable outlier was observed in the 25–44 age group in the high SDI region, where a significant increase occurred, with a percent change of 34.05% in ASMR (Figure 4C, Supplementary Table S7).

On the other hand, except in those over 75 years old, males had a higher ASMR (CVD) and a higher ASDR (CVD) related to cardiovascular diseases attributable to LPA than females over the past 3 decades, with the highest ratio of ASMR (CVD) in males to females in the 45–59 age group. The highest ratio of ASMR (CVD) in males to females among individuals aged 25–44 was in high-middle SDI region, reaching 2.23 in 2019, whereas the fastest change was observed in middle SDI region (percent change = 40.27%) (Figure 4D, Supplementary Table S7).

### 3.2. Type 2 diabetes

#### 3.2.1. Overall burden and trends of type 2 diabetes attributable to LPA

Both absolute number and age-standardised rates of mortality and disability in type 2 diabetes attributable to LPA have been rising globally

In 2019, the global estimate of death number of type 2 diabetes attributable to LPA was 125.20 (95% UI: 62.10–208.35) thousand. Globally, the death number had gradually increased over the past 30 years [49.78 (95% UI: 24.52–84.62) thousand in 1990]. Notably, the ASMRs (T2D) also increased by 9.46% from 1.48 (95% UI: 0.75–2.46) in 1990 to 1.62 (95% UI: 0.81–2.68) in 2019, with an EAPC of 0.26 (95% CI: 0.13–0.39) (Figure 1B, Table 2). Following a comparable upward trajectory as mortality, the DALYs reached 4549.21 (95% UI: 2,188.52–7,969.50) thousand in 2019, with an ASDR (T2D) of 55.92 (95% UI: 27.16–97.60) that increased by 24.27% from 1990 (Figure 1B, Table 2).

**Table 2:** DALYs and mortality of type 2 diabetes attributable—LPA in 27 GBD regions in 1990 and 2019.

Characteristics	DALYs (95% UI)					Mortality (95% UI)				
	1990		2019		EAPC	1990		2019		EAPC
	Number (no. ×10^3^)	ASR per 100,000	Number (no. ×10^3^)	ASR per 100,000		Number (no. ×10^3^)	ASR per 100,000	Number (no. ×10^3^)	ASR per 100,000	
Global	1,719.78 (782.03–3,071.19)	45.00 (21.34–79.47)	4,549.21 (2,188.52–7,969.50)	55.92 (27.16–97.60)	0.84 (0.78–0.89)	49.78 (24.52–84.62)	1.48 (0.75–2.46)	125.20 (62.10–208.35)	1.62 (0.81–2.68)	0.26 (0.13–0.39)
Sex										
Males	691.48 (288.49–1,292.43)	40.46 (17.92–73.94)	2,038.79 (898.39–3,704.69)	54.30 (24.96–97.58)	0.99 (0.93–1.05)	18.92 (8.50–33.82)	1.35 (0.63–2.36)	54.02 (25.71–91.47)	1.62 (0.79–2.75)	0.51 (0.37–0.65)
Females	1,028.29 (500.43–1,788.87)	49.07 (24.11–84.59)	2,510.42 (1,251.71–4,297.55)	57.51 (28.60–98.42)	0.73 (0.66–0.79)	30.86 (16.02–50.92)	1.58 (0.82–2.59)	71.18 (37.04–115.56)	1.62 (0.84–2.63)	0.09 (−0.04–0.23)
Location										
Low SDI	104.47 (44.73–200.82)	47.90 (21.76–88.05)	283.47 (125.34–520.12)	58.51 (27.26–105.95)	0.96 (0.84–1.09)	3.14 (1.42–5.88)	1.82 (0.86–3.25)	8.24 (3.93–14.33)	2.12 (1.02–3.62)	0.73 (0.56–0.89)
Low-middle SDI	280.19 (131.64–505.04)	51.33 (25.51–90.20)	872.09 (429.42–1,524.25)	68.11 (34.22–115.41)	1.19 (1.09–1.29)	8.25 (4.09–14.16)	1.93 (0.98–3.21)	27.38 (14.02–45.50)	2.53 (1.29–4.12)	1.07 (0.91–1.24)
Middle SDI	466.86 (208.93–839.38)	48.08 (22.40–84.40)	1,545.75 (707.73–2,717.55)	63.83 (30.05–110.33)	1.04 (0.99–1.09)	13.06 (6.11–22.87)	1.65 (0.81–2.81)	44.19 (21.59–74.54)	2.10 (1.03–3.48)	0.89 (0.82–0.96)
High-middle SDI	445.75 (213.45–759.00)	43.02 (21.15–72.53)	963.08 (481.37–1,662.41)	47.56 (23.77–81.85)	0.39 (0.29–0.49)	12.57 (6.48–20.29)	1.36 (0.71–2.16)	25.58 (13.38–40.53)	1.30 (0.68–2.05)	−0.20 (−0.33 to −0.07)
High SDI	420.34 (187.17–765.89)	40.49 (17.80–74.82)	878.97 (410.80–1,574.37)	49.72 (23.04–91.82)	0.63 (0.54–0.73)	12.70 (6.14–21.32)	1.21 (0.58–2.03)	19.64 (9.61–32.22)	0.91 (0.45–1.50)	−1.48 (−1.81 to −1.15)
Central Asia	7.89 (3.33–15.68)	17.37 (7.63–33.61)	31.38 (13.23–62.16)	44.88 (19.81–86.45)	3.18 (3.01–3.36)	0.17 (0.08–0.31)	0.39 (0.19–0.73)	0.77 (0.35–1.45)	1.27 (0.59–2.28)	3.78 (3.47–4.08)
Central Europe	45.11 (20.12–84.30)	30.98 (14.15–58.51)	90.79 (42.42–166.95)	42.89 (19.62–79.48)	1.30 (1.13–1.47)	1.03 (0.49–1.82)	0.73 (0.36–1.29)	2.03 (0.99–3.45)	0.89 (0.43–1.51)	1.03 (0.86–1.19)
Eastern Europe	28.58 (12.62–54.22)	10.57 (4.71–20.02)	56.12 (25.84–102.86)	15.82 (7.30–29.48)	1.69 (1.49–1.90)	0.52 (0.25–0.94)	0.20 (0.10–0.36)	1.39 (0.65–2.45)	0.38 (0.18–0.68)	2.39 (1.72–3.06)
Australasia	8.51 (3.87–14.84)	36.74 (16.53–64.08)	25.58 (12.64–43.24)	52.79 (25.28–91.17)	1.08 (0.92–1.25)	0.29 (0.14–0.47)	1.31 (0.66–2.12)	0.70 (0.38–1.09)	1.26 (0.67–1.96)	−0.49 (−0.84 to −0.13)
High-income Asia Pacific	64.23 (25.20–123.52)	32.10 (12.70–61.41)	132.13 (56.07–245.25)	31.76 (12.03–62.99)	−0.36 (−0.48 to −0.24)	1.44 (0.64–2.53)	0.78 (0.35–1.35)	2.50 (1.16–4.19)	0.45 (0.21–0.75)	−2.14 (−2.31 to −1.98)
High-income North America	160.63 (69.01–298.57)	46.34 (19.49–86.93)	293.35 (122.94–566.71)	49.02 (20.35–96.05)	0.50 (0.28–0.72)	4.60 (2.11–7.94)	1.25 (0.57–2.18)	6.25 (2.81–11.34)	0.93 (0.41–1.68)	−1.67 (−2.29 to −1.05)
Southern Latin America	6.08 (2.28–13.20)	13.39 (5.01–28.74)	20.06 (8.32–39.51)	24.08 (10.00–47.72)	2.31 (2.09–2.54)	0.20 (0.07–0.42)	0.48 (0.18–1.00)	0.56 (0.24–1.07)	0.65 (0.28–1.24)	1.26 (1.00–1.52)
Western Europe	268.52 (127.15–460.60)	46.11 (21.17–79.76)	476.39 (227.16–827.13)	54.14 (24.70–98.00)	0.22 (−0.02–0.45)	9.54 (4.82–15.31)	1.58 (0.80–2.55)	13.01 (6.71–20.62)	1.13 (0.58–1.80)	−1.52 (−1.72 to −1.32)
Andean Latin America	5.46 (1.95–11.19)	27.91 (10.18–55.80)	28.41 (11.30–54.79)	51.53 (20.66–98.65)	2.18 (2.10–2.26)	0.17 (0.07–0.33)	0.96 (0.37–1.84)	0.91 (0.39–1.64)	1.70 (0.73–3.06)	2.10 (1.93–2.26)
Caribbean	40.81 (19.67–69.12)	157.87 (77.22–265.74)	95.19 (48.13–160.75)	184.13 (93.50–310.57)	0.47 (0.41–0.53)	1.29 (0.69–2.03)	5.30 (2.86–8.28)	2.60 (1.41–4.05)	5.02 (2.74–7.80)	−0.30 (−0.37 to −0.22)
Central Latin America	74.83 (28.32–147.11)	88.57 (34.44–171.13)	224.20 (85.60–435.23)	95.09 (36.58–183.95)	0.21 (−0.00–0.43)	2.12 (0.85–4.04)	2.84 (1.17–5.31)	6.71 (2.77–12.47)	2.99 (1.24–5.51)	−0.13 (−0.34–0.08)
Tropical Latin America	171.72 (93.70–263.76)	181.03 (101.53–273.50)	429.05 (248.38–650.92)	175.99 (101.65–266.03)	0.01 (−0.04–0.06)	4.34 (2.52–6.31)	5.42 (3.22–7.85)	11.49 (6.87–16.45)	4.97 (2.95–7.11)	−0.24 (−0.30 to −0.17)
North Africa and Middle East	179.40 (89.64–298.64)	106.43 (56.11–173.23)	685.05 (356.44–1,111.48)	154.04 (82.71–245.60)	1.53 (1.40–1.66)	4.95 (2.67–7.89)	3.59 (2.01–5.57)	14.39 (8.09–22.10)	3.92 (2.23–5.85)	0.51 (0.36–0.65)
South Asia	251.82 (113.58–461.28)	51.55 (24.76–91.45)	783.77 (374.09–1,426.19)	62.25 (30.66–109.68)	1.14 (0.90–1.38)	7.24 (3.45–12.78)	2.04 (1.02–3.43)	26.17 (13.09–43.74)	2.52 (1.28–4.10)	0.95 (0.60–1.30)
East Asia	192.12 (83.66–357.29)	24.27 (11.02–43.07)	487.17 (208.48–922.44)	24.61 (10.86–45.77)	−0.37 (−0.54 to −0.21)	4.63 (2.14–8.44)	0.72 (0.34–1.25)	13.18 (6.12–23.19)	0.77 (0.36–1.34)	−0.03 (−0.24–0.18)
Oceania	5.35 (2.19–10.51)	182.23 (80.14–342.27)	20.00 (8.28–38.49)	281.36 (125.34–525.55)	1.32 (1.03–1.61)	0.17 (0.07–0.31)	7.41 (3.46–13.27)	0.60 (0.27–1.12)	11.04 (5.29–19.34)	1.14 (0.86–1.42)
Southeast Asia	112.73 (43.75–228.12)	47.40 (19.61–94.33)	403.33 (160.62–772.59)	69.05 (28.79–130.61)	1.25 (1.17–1.33)	3.83 (1.58–7.64)	1.89 (0.80–3.57)	12.94 (5.53–24.21)	2.51 (1.11–4.57)	0.96 (0.90–1.03)
Central Sub-Saharan Africa	16.63 (6.35–34.50)	77.24 (31.74–152.66)	44.91 (17.45–90.16)	86.51 (35.70–163.78)	0.35 (0.28–0.42)	0.49 (0.19–0.95)	3.03 (1.28–5.62)	1.18 (0.50–2.23)	3.06 (1.39–5.47)	−0.05 (−0.10–0.00)
Eastern Sub-Saharan Africa	14.61 (5.53–32.30)	21.55 (8.29–47.28)	30.57 (11.64–68.15)	21.37 (8.19–44.96)	−0.08 (−0.11 to −0.05)	0.53 (0.21–1.17)	0.97 (0.39–2.05)	1.11 (0.43–2.34)	0.96 (0.38–1.95)	−0.10 (−0.14 to −0.06)
Southern Sub-Saharan Africa	28.91 (13.36–50.29)	105.29 (49.54–180.12)	86.87 (39.28–151.74)	160.80 (73.46–275.93)	2.00 (1.45–2.55)	0.95 (0.45–1.59)	3.99 (1.94–6.55)	3.14 (1.49–5.20)	6.74 (3.22–11.16)	2.43 (1.81–3.05)
Western Sub-Saharan Africa	35.84 (14.96–69.95)	44.43 (19.27–84.71)	104.88 (43.24–200.94)	61.31 (26.15–113.36)	1.06 (0.89–1.24)	1.28 (0.56–2.44)	1.90 (0.84–3.48)	3.56 (1.53–6.45)	2.56 (1.12–4.54)	1.01 (0.81–1.21)

#### 3.2.2. Impact of LPA on type 2 diabetes based on SDI and geographical regions

ASMR (T2D) and ASDR (T2D) attributable to LPA in 2019 exceed that in 1990 globally, exhibiting greater increases in lower SDI regions

**Figure 5:**
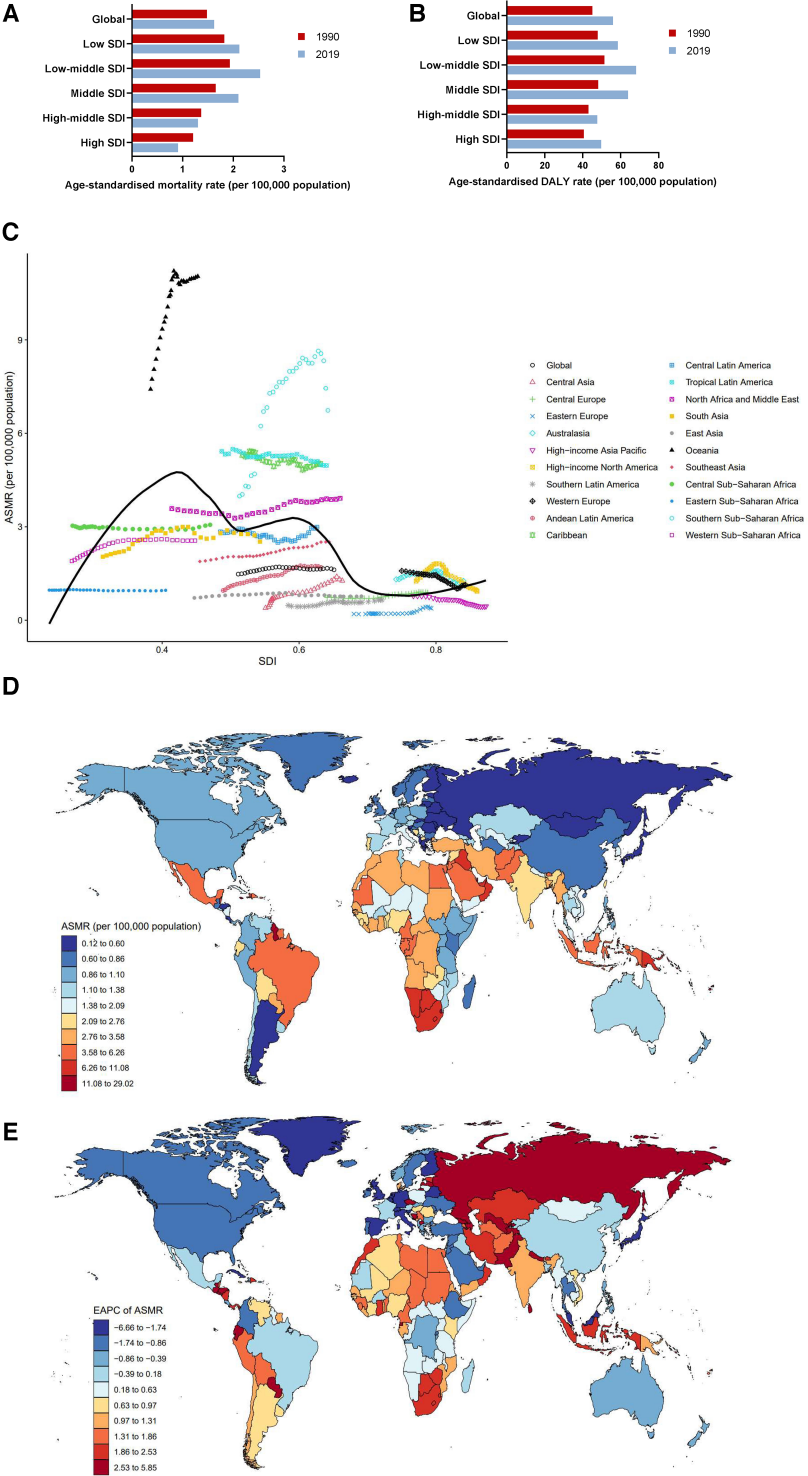
Burden of type 2 diabetes attributable to LPA by location. (**A**) ASMRs globally and in territories with low to high SDIs in 1990 and 2019. (**B**) ASDRs globally and in territories with low to high SDIs in 1990 and 2019. (**C**) ASMRs across 21 GBD regions by SDI, 1990–2019. (**D**) ASMRs in 204 countries and territories by geographical region in 2019. (**E**) EAPCs of ASMRs in 204 countries and territories from 1990 to 2019.

In 2019, the highest ASMR (T2D) and ASDR (T2D) of type 2 diabetes attributable to LPA were observed in the low-middle SDI region, while lowest ASMR (T2D) and ASDR (T2D) were observed in the high SDI region and high-middle SDI region, respectively. Except for the high-middle and high SDI regions, such as Western Europe [EAPC = −1.52 (95% CI: −1.72 to −1.32)], all other regions (low SDI, low-middle SDI, middle SDI) showed an upward trend in mortality rates from 1990 to 2019 (Table 2, Figure 5A). In all of the 5 SDI regions, the absolute number of DALYs and ASDRs (T2D) increased (Table 2, Figure 5B). Among the 21 GBD regions, only high-income Asia Pacific [EAPC = −0.36 (95% CI: −0.48 to −0.24)], East Asia [EAPC = −0.37 (95% CI: −0.54 to −0.21)], and Eastern Sub-Saharan Africa [EAPC = −0.08 (95% CI: −0.11 to −0.05)] showed a decreasing trend in ASDR (T2D). Oceania maintained the highest ASMR (T2D) and ASDR (T2D) from 1990 to 2019, whereas Central Asia showed the sharpest rise in ASDR (T2D) over the study period [EAPC = 3.18 (95% CI: 3.01–3.36)] (Table 2, Figure 5C, Supplementary Figure S3A).

The burden of type 2 diabetes attributable to LPA varied dramatically across 204 countries and territories. In 2019, the highest ASMR (T2D) and ASDR (T2D) were recorded in Fiji (ASMR = 29.02 [95% UI: 14.77–49.57], ASDR = 672.73 [95% UI: 317.76–1,188.43]), while the lowest ASMR (T2D) and ASDR (T2D) were in Ukraine (ASMR = 0.12 [95% UI: 0.05–0.23], ASDR = 9.33 [95% UI: 3.80–18.97]). From 1990 to 2019, some countries experienced rapid increases in ASMRs (T2D), particularly Russia and Central Asia countries such as Kazakhstan and Uzbekistan, although their ASMRs (T2D) in 2019 were relatively low (Supplementary Figure S3C). The country with the fastest increase in ASMR (T2D) was Uzbekistan, with EAPC = 5.85 (95% CI: 5.26–6.44). In contrast, there were annual decreases in ASMR (T2D) in a few countries such as Singapore, Japan, Switzerland, and Cyprus, all of which had high SDI scores (Figures 5D–E, Supplementary Table S6). For DALYs, India [576.28 (95% UI: 277.33–1,049.49) thousand], Brazil [424.72 (95% UI: 246.90–644.02) thousand], and United States of America [268.54 (95% UI: 112.24–521.56) thousand] had the highest absolute number of DALYs in 2019. Most of developing countries in Asia and Africa showed an upward trend in ASDR (T2D) from 1990 to 2019, with Uzbekistan [EAPC = 5.05 (95% CI: 4.71–5.39)] showing the fastest increase (Supplementary Figure S3D, Table S6). The associations between ASDR (T2D) and SDI scores across 204 countries and territories in 2019 are shown in Supplementary Figure S3B.

#### 3.2.3. Sex differences in type 2 diabetes burden due to LPA

Females have higher ASMR (T2D) due to LPA than males, but the situation may be reversed in the near future, given the sharp increase of ASMR in males

**Figure 6:**
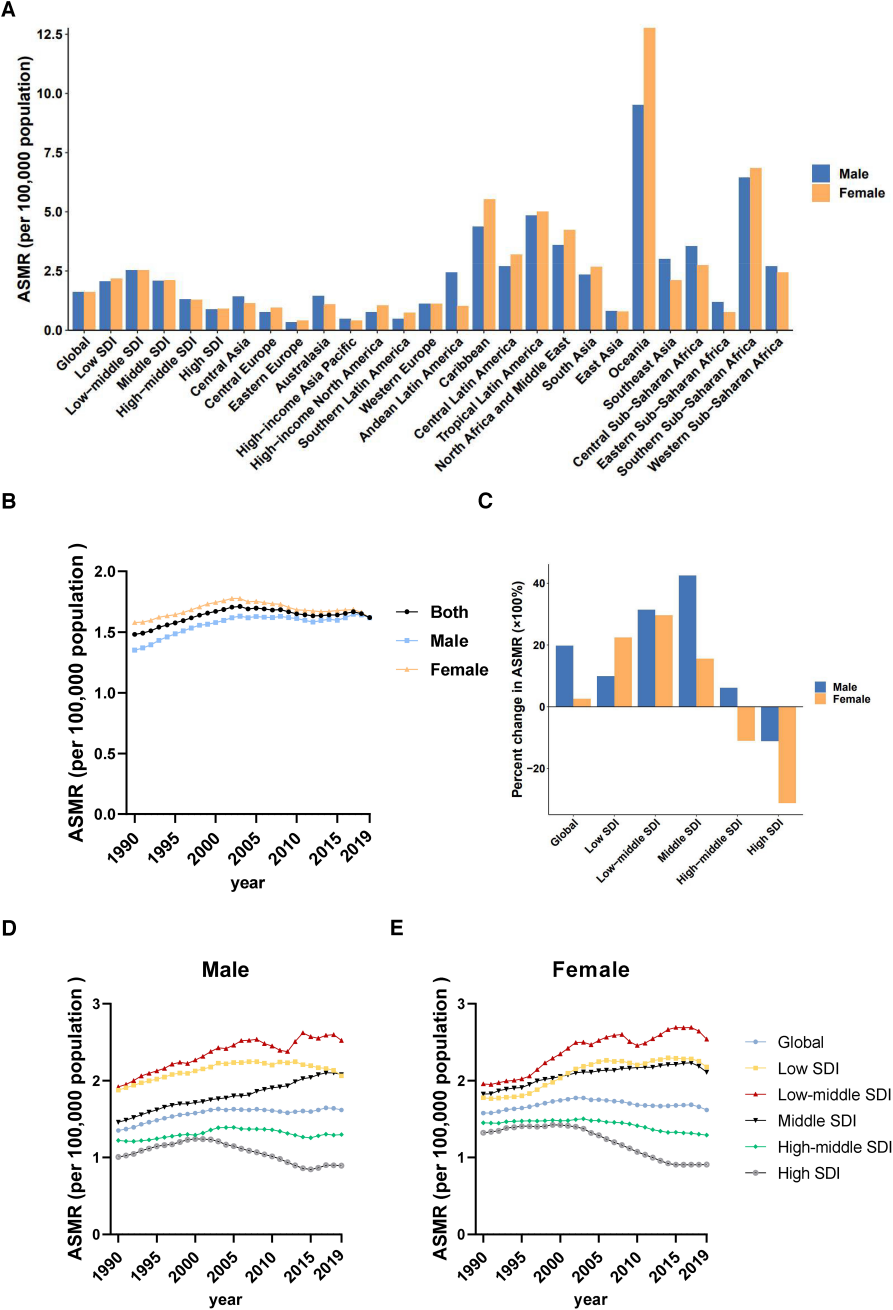
Sex differences and trends of type 2 diabetes mortality rates attributable to LPA in different regions. (**A**) ASMRs in males and females globally, in territories with low to high SDIs and in 21 GBD regions in 2019. (**B**) Temporal trend of ASMRs globally by sex, 1990–2019. (**C**) Percent changes in ASMRs in males and females between 1990 and 2019. (**D**) Temporal trend of ASMRs in males by SDI, 1990–2019. (**E**) Temporal trend of ASMRs in females by SDI, 1990–2019.

Globally, the number of deaths increased in both males and females during the past 3 decades, reaching 54.02 (95% UI: 25.71–91.47) thousand in males and 71.18 (95% UI: 37.04–115.56) thousand in females in 2019. The ASMRs (T2D) in females had consistently been higher than that in males, and the gap appeared to reduce over time (Table 2, Figure 6B). In high SDI region, the ASMRs (T2D) in both sexes showed a downward trend, and the decline for female was greater than males (as measured by percent change in ASMR 1990–2019). In addition, high SDI region was the only region that facing a decrease in ASMR (T2D) for males. On the other hand, ASMR (T2D) in males was found to have the largest increase in the middle SDI region between 1990 and 2019, with a 42.50% increase in ASMR (T2D) (Figures 6C–E). In 2019, the highest ASMRs (T2D) in males [9.50 (95% UI: 3.93–17.98)] and females [12.77 (95% UI: 6.49–21.19)] were both observed in Oceania, while the lowest ASMRs (T2D) in males and females were in Eastern Europe [0.33 (95% UI: 0.16–0.58)] and High-income Asia Pacific [0.41 (95% UI: 0.20–0.67)], respectively (Figure 6A).

As for DALYs, except for low SDI region, the ASDRs (T2D) in males rose much sharper than that in females, which also explains the gradual reduction of sex difference. Furthermore, the region with the largest percentage increase in the type 2 diabetes-related ASDR (T2D) in males was the middle SDI region (49.64%), whereas the region with the fastest growth for females was the low-middle SDI region (29.09%) (Supplementary Figure S4). In 2019, the highest ASDRs (T2D) in males [208.55(95% UI: 78.18–428.11)] and females [360.65 (95% UI: 173.36–632.22)] were observed in Oceania, while the lowest ASDRs (T2D) in both sexes were observed in Eastern Europe (Supplementary Figure S4A).

#### 3.2.4. Burden and changes of type 2 diabetes attributable to LPA at different age groups

Increase in ASMR (T2D) for the 25–44 age group was enormous globally, particularly notable in high SDI region

**Figure 7:**
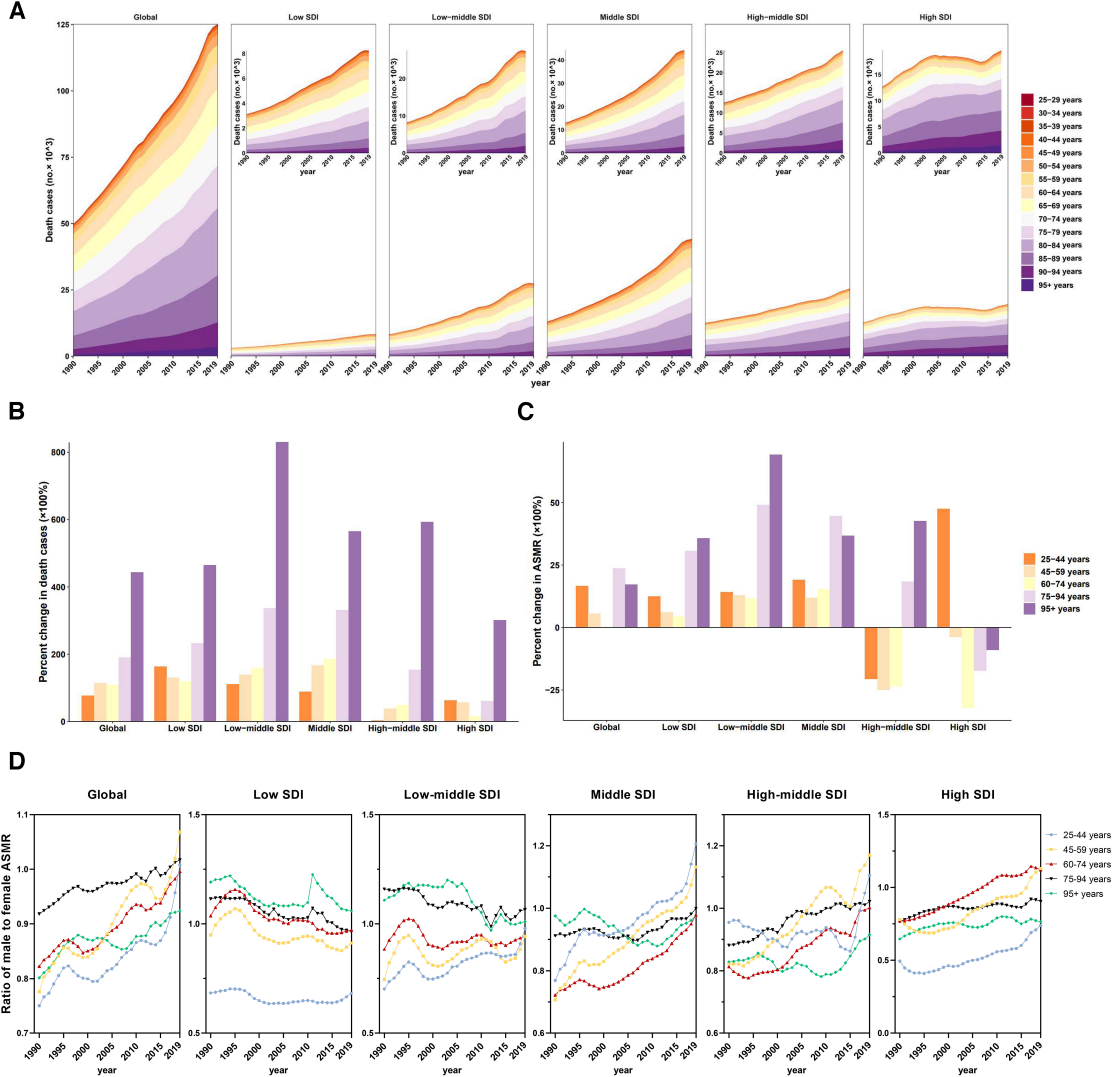
Mortality of type 2 diabetes attributable to LPA by age groups in regions with different SDI. (**A**) Contribution of each age group to total deaths over the period from 1990 to 2019 by SDI region. (**B**) Percent changes in deaths in five age groups between 1990 and 2019 by SDI region. (**C**) Percent changes in ASMR in five age groups between 1990 and 2019 by SDI region. (**D**) Ratio of males to females ASMRs by age group and by SDI region, 1990–2019.

Figure 7A exhibited the contribution of different age groups to the deaths of type 2 diabetes attributable to LPA from 1990 to 2019. The highest number of deaths was observed in the 80–84 age group (global cumulative deaths = 484. 960 thousand) in low-middle, middle, high-middle, and high SDI regions. However, the age group with the most deaths in low SDI region advanced to 70–74 years old (Figure 7A). The analysis of changes in death cases revealed that the age group of 95+ years had the largest increase from 1990 to 2019 (percent change = 443.09%), particularly in the low-middle SDI region, where the increase reached 829.69%. In the 25–44 age group, the percent change of death cases decreased as SDI increased. And the largest change (162.74%) occurred in low SDI region, surpassing even that of the 45–74 age group (Figure 7B).

From 1990 to 2019, ASMRs (T2D) increased significantly in both the 25–59 and 75+ age groups, while it remained nearly unchanged among those aged 60–74. The predominant factor influencing the trend in the 60–74 age group is the decline observed in regions with higher SDI. It is worth noting that although the largest increase of deaths in 25–44 age group was observed in low SDI region, the largest increase of ASMR (T2D) occurred in high SDI region, where all other age groups showed a downward trend (Figure 7C). As for DALYs, the ASDRs (T2D) of all age groups increased in all 5 SDI regions, including high SDI region (Supplementary Table S8).

Based on sex-specific data, the ASMR (T2D) in males was persistently lower than that in females in all age groups, but the gap was gradually narrowing over time. In 2019, the ratio of males to females ASMR (T2D) in the >75 age group was greater than 1, mainly due to the contributions from low and low-middle SDI regions. The largest change in the ratio of males to females ASMR (T2D) (0.78 in 1990, 1.07 in 2019) occurred in the 45–59 age group over the past 30 years (Figure 7D). In 2019, the largest sex difference in ASDR (T2D) was observed in the 25–44 age group, with females being approximately 1.21 times as numerous as males. The largest percentage changes in ASDR (T2D) in both sexes were also observed in the 25–44 age group (76.78% for males, 42.34% for females) (Supplementary Table S8).

## 4. Discussion

The present study indicates that the absolute number of mortality and DALYs from cardiovascular diseases and type 2 diabetes attributable to LPA increased from 1990 to 2019. However, the time trends attributable to LPA of ASMR (CVD) and ASDR (CVD) showed a significant downward trend, while ASMR (T2D) ASDR (T2D) showed an upward trend. In 2019, the death burden of cardiovascular diseases attributable to LPA was about five times that of type 2 diabetes, while the DALYs was twice as high for type 2 diabetes vs. cardiovascular diseases. These findings are consistent with the known association of lower rates of physical activity for people with type 2 diabetes and the disabling complications of retinopathy and neuropathy—though causality is not necessarily implied as these complications also serve as barriers to physical activity (23). In view of the large increase in longevity, the quality of those extra years becomes even more important (24). Type 2 Diabetes is incurable and is significantly associated with the risk of death from cardiovascular diseases (25, 26). Given the rising type 2 diabetes-related burden, especially in regions with fast-growing diabetes rates, increasing awareness of the double burden of diseases is an urgency.

In terms of SDI, our findings revealed that middle SDI and high-middle SDI regions bear the heaviest burden of cardiovascular diseases attributable to LPA, whereas low SDI region has the lowest death number and the second-lowest ASMR (CVD) across all regions. Notably, previous studies have indicated that people with low income and education level have a higher prevalence of physical inactivity (27). This is inconsistent with our results, and the reason may involve the lower level of medical diagnosis and treatment in low SDI region, potentially resulting in underestimation. In contrast, the middle SDI region was undergoing rapid economic and technological development, which led to more sedentary lifestyle among people because of work demands or high use of smart phones (28, 29). On the other hand, both ASMR (CVD) and ASDR (CVD) were the lowest in high SDI region in 2019, which also exhibited the most significant decline across all regions in the past 30 years. This is reasonable, as a higher SDI corresponds to higher per capita income, fertility and average education level in a region (15). And these factors are closely related to the public's awareness of healthful physical activity and the level of healthcare.

The burden of IHD, the most prevalent type of CVD, was consistent with that of all CVDs in our study. Both GBD data and WHO data revealed that Eastern Europe had the highest ASDR for all-cause IHD. However, our results showed that the highest ASMR and ASDR of IHD attributable to LPA occurred in the North Africa and Middle East region, where rates remained higher than the global average throughout the 30-year observation period. As for stroke, the highest ASDR for all-cause stroke was reported in Oceania (16, 30). However, our results showed that the North Africa and Middle East region also ranked first in ASMR (stroke) and ASDR (stroke) in 2019. Some studies have elucidated that 49% of adults and 75% of young people in 17 countries in the North Africa and Middle East region did not exercise sufficiently, which was related to lack of social support and inadequate sports facilities (31, 32). In addition, we observed that ASMR (stroke) in Eastern Europe kept elevated for a long time (1991–2000), while declined rapidly since 2000. This indicates that the implementation of the European Stroke Organisation (ESO) Guideline, with its emphasis on the importance of physical activity for stroke prevention, has achieved encouraging results in preventing and controlling stroke (33).

Our results showed that the burden of type 2 diabetes attributable to LPA was also the heaviest in the middle SDI region, especially in Oceania countries such as Fiji. According to the WHO report in 2022, 81%−86% of children and adolescents (11–17 years) and 11%−24% of adults (18+ years) in Fiji lacked sufficient physical activity. In Fiji, 85% of deaths were due to noncommunicable diseases, of which the largest proportion was cardiovascular diseases and diabetes (34). This suggests that urgent measures should be taken in these areas to reverse this negative situation. In our study, the ASMR of type 2 diabetes attributable to LPA increased year by year in most countries, with the fastest increase in Central Asian countries such as Kazakhstan and Uzbekistan, while high SDI countries such as Singapore, Japan, Switzerland, and Cyprus showed a downward trend. One of the reasons for this opposite trend may be the differences in the health-care system. In Kazakhstan, public health expenditure is only 1.8 percent of GDP, and public health care, long-term care and rehabilitation are underdeveloped at all levels of the country's health-care system (35). Uzbekistan encounters comparable challenges (36). In contrast, Singapore has achieved universal health coverage through a mixed-financing system, with national healthcare expenditures of 4.47 percent of GDP underpinning the country's preventive, clinical, and rehabilitative healthcare (37). Similarly, other high SDI countries such as Japan and Switzerland also have very comprehensive and advanced healthcare systems (37). Unfortunately, although the ASMR (T2D) declined, ASDR (T2D) still had a slight increase in high SDI region. In fact, the proportion of diabetes deaths due to LPA (15.68%) is higher than the proportion of all-cause diabetes deaths (12.63%) (38) in high SDI region in 2019. This indicates that LPA-related diabetes burden in high SDI region should not be ignored. Given the distribution of people's concept levels and the complexity of social culture and environment in different regions, the government should take region-specific interventions to promote physical activity and reduce the burden of cardiovascular diseases and type 2 diabetes.

Sex-specific disparity exists in the burden of cardiovascular diseases attributable to LPA. The ASMR (CVD) for males is higher than that for females, and the decline from 1990 to 2019 is smaller for males than for females. Especially noteworthy is that Central Asia, Eastern Europe, and Andean Latin America have the largest sex gap. A possible explanation for this phenomenon is that these regions have a large sex difference in employment; as a result, males may have less time and willingness to participate in physical activity after work, while females may be active as they perform household chores/child care and may have more leisure time to actively exercise (39). To the extent this is true in these regions, the government may incentivize active commuting to work (e.g., bicycle, walking, public transportation) and/or may encourage males to engage in extra physical activity after work by emphasizing the importance of physical activity for health.

In contrast, for type 2 diabetes attributable to LPA, the ASMR (T2D) for females is higher than that for males. Nevertheless, this gender gap is narrowing year by year and is exhibiting a trend of reversal. The region with the largest difference in 2019 is Oceania, where the ASMR (T2D) for females is as high as 12.77%. To try to understand this difference further, according to a report by the Australian government, 59% of females (18+ years) did not meet the physical activity guidelines (40), while for males (18+ years) it was 50% (41). In addition, a study in New Zealand also showed that males meet the physical activity recommendation significantly more than females (42). The New Zealand study also pointed out that socio-economic deprivation reduces the probability of females meeting the exercise standard, while this has little effect on males (42). These indicate that socio-demographic characteristics of different subgroups should be taken into account by policymakers when designing interventions, and that these interventions should be adapted to the specific contexts of each subgroup.

In this study, mortality from cardiovascular diseases attributable to LPA increases with advancing age. The majority of deaths related to cardiovascular diseases occurred in 80–84 years old people, while the largest increase in the number of deaths was in the 95+ age group. Therefore, in older adults, varying levels of functional disability often necessitate a tailored physical activity intervention that aligns with both functional status and patient-centered goals of care. In addition, ASMR (CVD) and ASDR (CVD) for all ages and regions have declined in recent decades, except for 25–44 age group in high SDI region, which is closely associated with healthcare improvements worldwide. Similarly, the burden of type 2 diabetes attributable to LPA in high SDI region was also found to have a trend of younger age. From 1990 to 2019, ASMR of type 2 diabetes attributable to LPA rose for 25–44 age group, while declined for other age groups. In fact, a growing number of articles show an increasing disease burden of type 2 diabetes for the younger generation (43). Consistent with that, in our study, ASDR (T2D) of 25–44 age group increased fastest among five age groups in global level. Our results strongly emphasize the need for youth in high SDI region to increase physical activity as a crucial measure in reducing the burden of cardiovascular diseases and diabetes. At the same time, countries in other SDI regions should take warning to monitor physical activity for youth, enhance their awareness, and preventing any escalation of unhealthy trends over time.

Finally, the burden of other noncommunicable disease due to LPA cannot be ignored. For example, colorectal cancer accounts for 7% of total LPA-related death, and colorectal cancer also increases the risk of cardiovascular diseases (44). From another perspective, engaging in physical activity proves highly beneficial for improving physical function during and after cancer treatment. A prior meta-analysis indicated that physical activity could lower the risk of mortality for breast and colorectal cancer patients (19). Recognizing the importance of physical activity, the WHO has encouraged various activities and puts forth policy recommendations, such as using mass media to raise awareness about the benefits of exercise (45).

Our study has some limitations: First, due to the inability to collect individual patient information such as religious beliefs and occupations, which may affect physical activities, we cannot provide analysis from personal perspective. Second, differences in medical professional knowledge and equipment across countries may be one important source of geographical heterogeneity. Since the multiple risk factors and complications of cardiovascular diseases and diabetes, it is hard to distinguish deaths attributable to LPA, thus making it difficult to accurately evaluate the mortality rate. Third, our study does not further analyze local characteristics such as differences between provinces or states, which requires further investigation.

## 5. Conclusion

Threat of cardiovascular diseases and type 2 diabetes attributable to LPA remains one of the major challenges for public health. Screening and protection should be strategically directed towards populations currently bearing and escalating the heaviest burden. Notably, individuals aged 25–44 years old, particularly in high SDI countries, are a notable outlier in the improvements seen by other age groups. Gender disparities revealed higher ASMR (CVD) and a greater increase of ASMR (T2D) in males compared to females. Geographically, North Africa and the Middle East, and Oceania are the regions most affected by cardiovascular diseases and type 2 diabetes.

Generally, it is crucial to implement comprehensive and context-specific interventions for promoting of physical activity, along with routine assessments of cardiorespiratory fitness, to reduce the burden of cardiovascular diseases and type 2 diabetes. Meanwhile, vigilance should be heightened to prevent one disease to the detriment of another.

## Data Availability

The original contributions presented in the study are included in the article/**Supplementary Material**, further inquiries can be directed to the corresponding authors.

## Supplementary material

SUPPLEMENTARY FIGURE S1

DALYs of cardiovascular diseases attributable to LPA by location. (**A**) ASDRs across 21 GBD regions by SDI, 1990-2019. (**B**) ASDRs in 204 countries and territories by SDI in 2019. (**C**) ASDRs in 204 countries and territories by geographical region in 2019. (**D**) EAPCs of ASDRs in 204 countries and territories from 1990 to 2019.

SUPPLEMENTARY FIGURE S2

Sex differences and trends of cardiovascular diseases DALY rates attributable to LPA in different regions. (**A**). ASDRs in males and females globally, in territories with low to high SDIs and in 21 GBD regions in 2019. (**B**) Temporal trend of ASDRs globally by sex, 1990–2019. (**C**) Percent changes in ASDRs in males and females between 1990 and 2019. (**D**) Temporal trend of ASDRs in males by SDI, 1990–2019. E. Temporal trend of ASDRs in females by SDI, 1990–2019.

SUPPLEMENTARY FIGURE S3

DALYs of type 2 diabetes attributable to LPA by location. (**A**) ASDRs across 21 GBD regions by SDI, 1990–2019. (**B**) ASDRs in 204 countries and territories by SDI in 2019. (**C**) ASDRs in 204 countries and territories by geographical region in 2019. (**D**) EAPCs of ASDRs in 204 countries and territories from 1990 to 2019.

SUPPLEMENTARY FIGURE S4

Sex differences and trends of type 2 diabetes DALY rates attributable to LPA in different regions. (**A**) ASDRs in males and females globally, in territories with low to high SDIs and in 21 GBD regions in 2019. (**B**) Temporal trend of ASDRs globally by sex, 1990–2019. (**C**) Percent changes in ASDRs in males and females between 1990 and 2019. (**D**) Temporal trend of ASDRs in males by SDI, 1990–2019. E. Temporal trend of ASDRs in females by SDI, 1990–2019.

SUPPLEMENTARY FIGURE S5

Burden of IHD attributable to LPA by location. (**A**) ASMRs s across 21 GBD regions by SDI, 1990–2019. (**B**) ASDRs across 21 GBD regions by SDI, 1990–2019. (**C**) ASDRs in 204 countries and territories by SDI in 2019.

SUPPLEMENTARY FIGURE S6

Burden of stroke attributable to LPA by location. (**A**) ASMRs across 21 GBD regions by SDI, 1990–2019. (**B**) ASDRs across 21 GBD regions by SDI, 1990–2019. (**C**) ASDRs in 204 countries and territories by SDI in 2019.
