# Supplementary figures and images for: The global burden of cardiovascular diseases and type 2 diabetes attributable to low physical activity, 1990–2019: an analysis from the global burden of disease study
Source: Front Cardiovasc Med. 2023 Dec 19;10:1247705. doi: 10.3389/fcvm.2023.1247705 (PMC10762785; doi:10.3389/fcvm.2023.1247705)

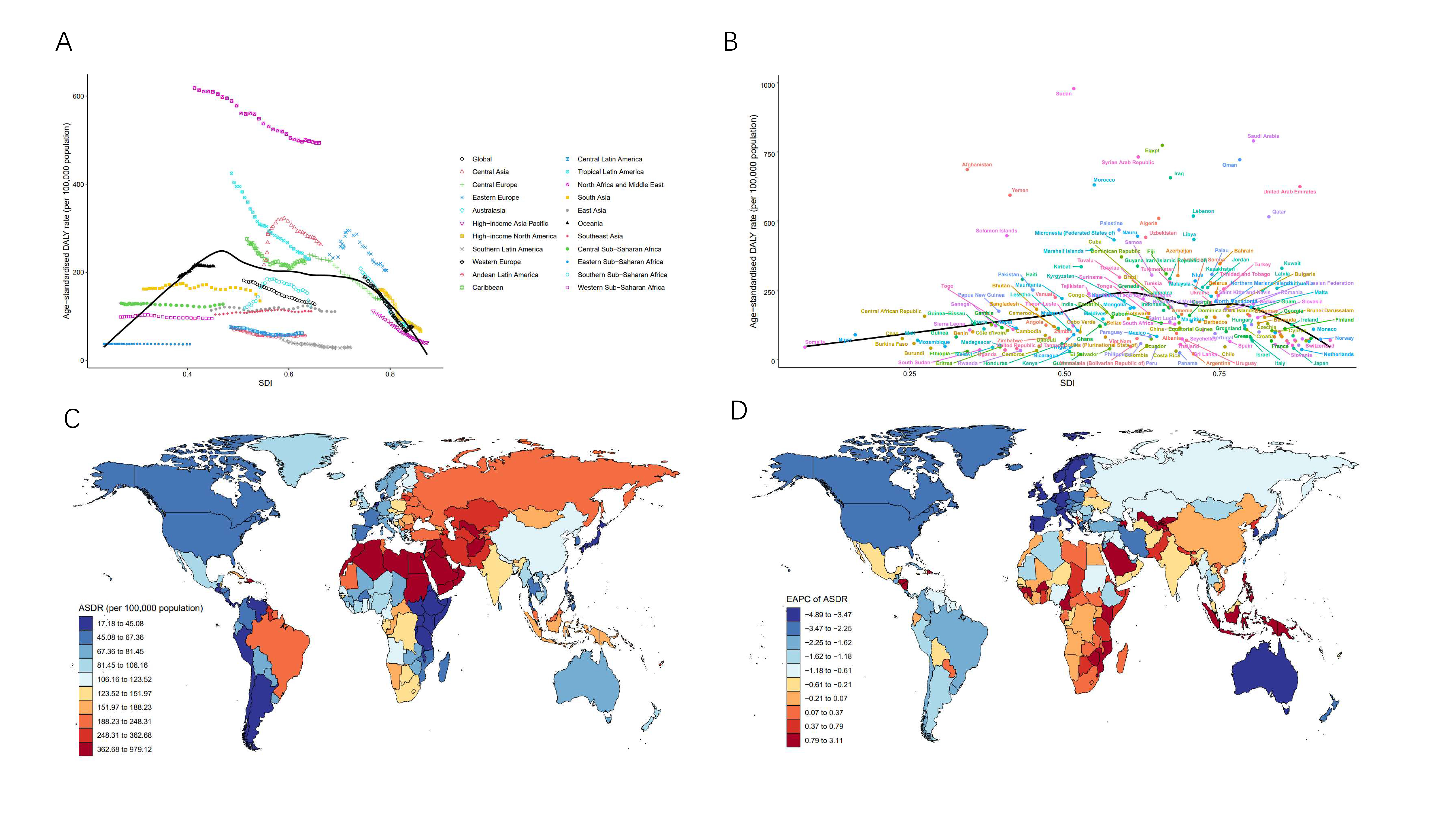

Supplement: Supplementary file 1 [file Image1.jpeg]

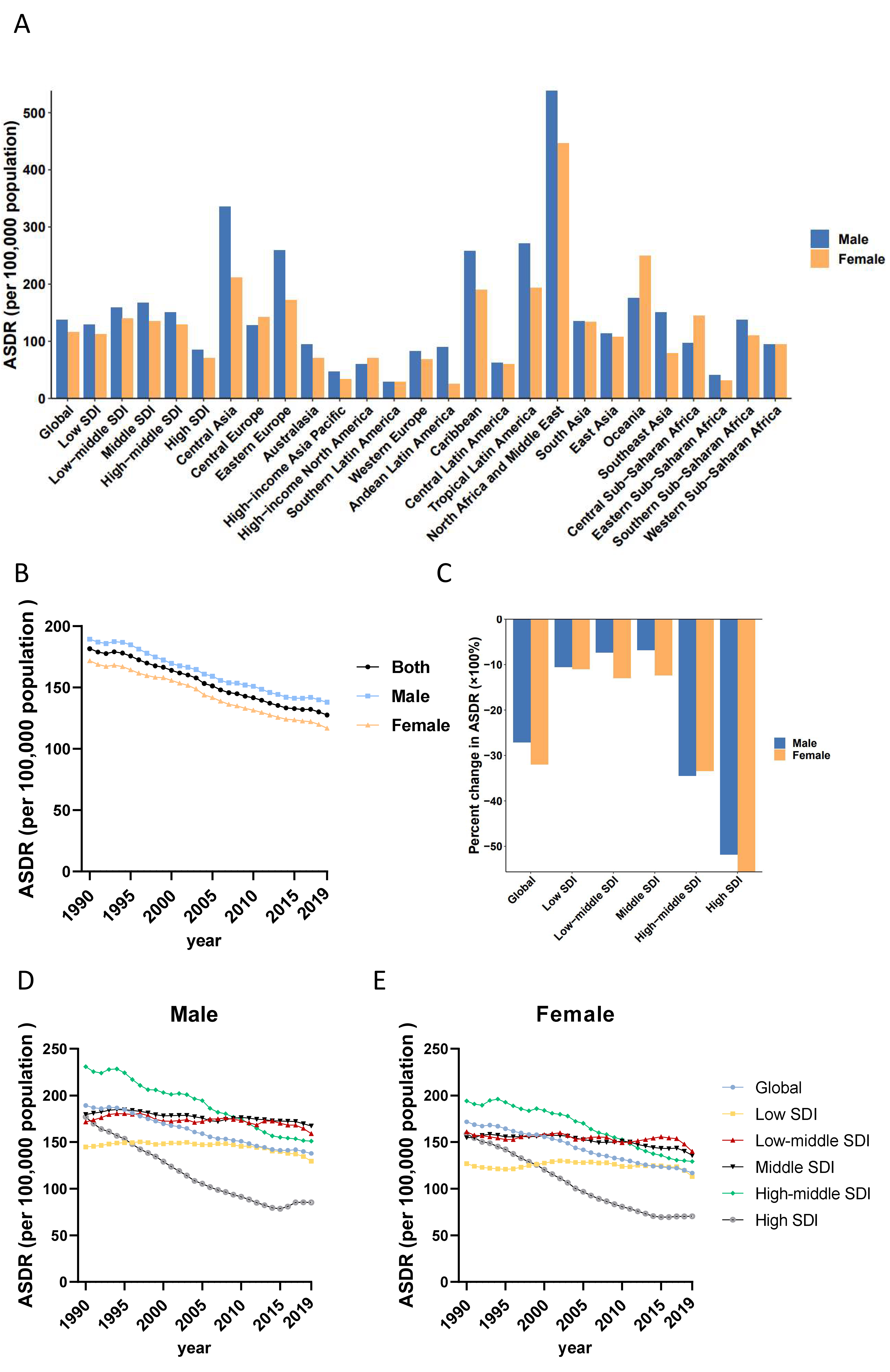

Supplement: Supplementary file 2 [file Image2.jpeg]

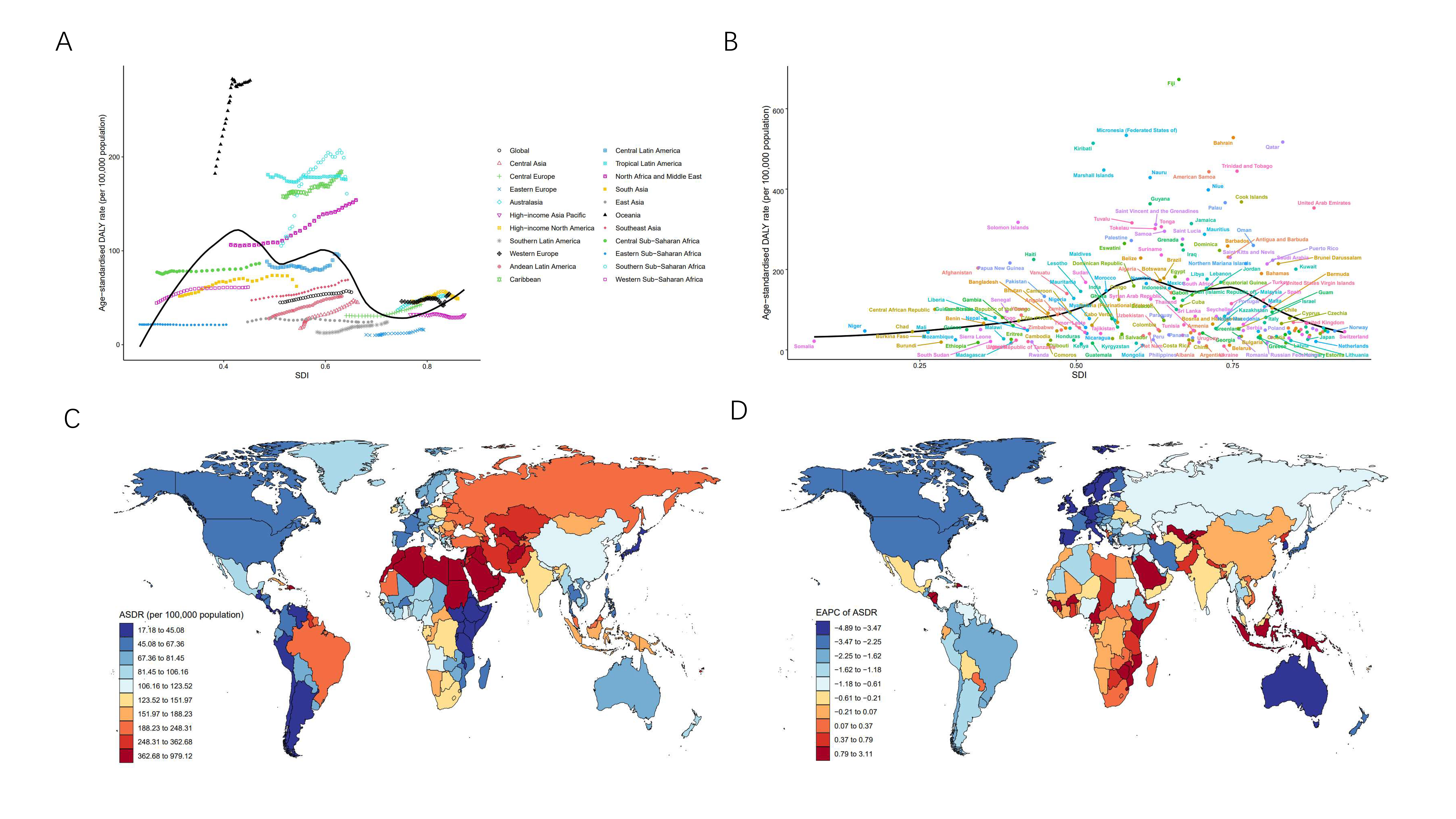

Supplement: Supplementary file 3 [file Image3.jpeg]

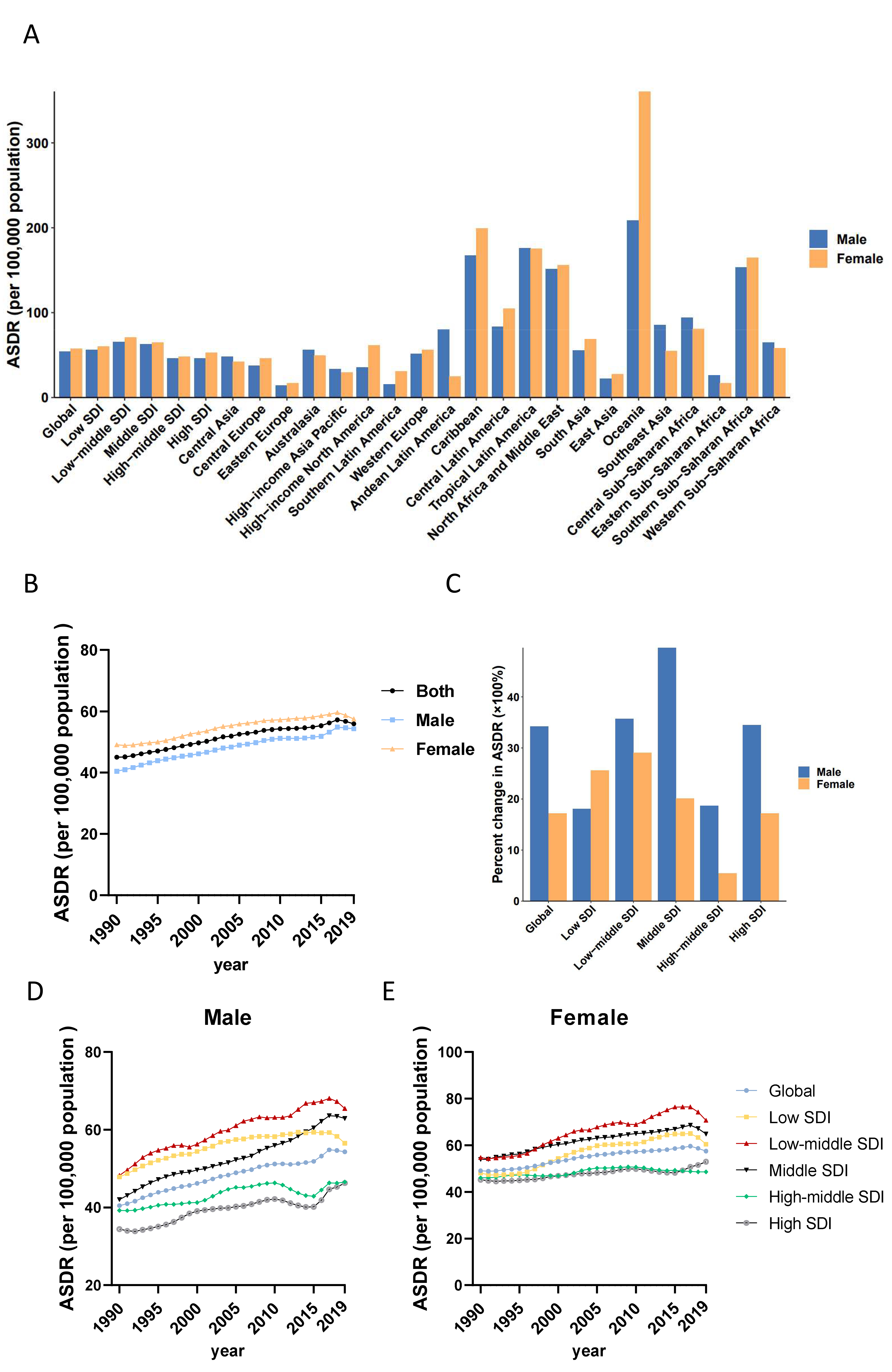

Supplement: Supplementary file 4 [file Image4.jpeg]
