# Supplementary material for: The global burden of cardiovascular diseases and type 2 diabetes attributable to low physical activity, 1990–2019: an analysis from the global burden of disease study
Source: Front Cardiovasc Med. 2023 Dec 19;10:1247705. doi: 10.3389/fcvm.2023.1247705 (PMC10762785; doi:10.3389/fcvm.2023.1247705)
Supplement: Supplementary file 7 [file Table1.docx]

| **Supplemental table 1. DALYs and mortality of ischemic heart disease attributable to LPA in 27 GBD regions in 1990 and 2019.** | | | | | | | | | | | | | |  |
| --- | --- | --- | --- | --- | --- | --- | --- | --- | --- | --- | --- | --- | --- | --- |
|  | **DALYs (95% UI)** | | | | | |  | **Mortality (95% UI)** | | | | | |  |
|  | **1990** | |  | **2019** | | **EAPC** |  | **1990** | |  | **2019** | | **EAPC** |  |
| **location** | **Number(no.×10^3^)** | **ASR per100,000** |  | **Number(no.×10^3^)** | **ASR per100,000** |  |  | **Number(no.×10^3^)** | **ASR per100,000** |  | **Number(no.×10^3^)** | **ASR per100,000** |  |  |
| Global | 4843.16 (1512.33to11108.88) | 138.09 (45.07to310.37) | | 7586.67 (2613.51to16747.20) | 96.36 (33.45to210.82) | 0.57 (0.43to0.77) |  | 283.40 (93.69to616.21) | 9.46 (3.25to19.81) |  | 486.78 (175.73to1003.28) | 6.52 (2.36to13.31) | -1.38 (-1.44to-1.33) | |
| Low SDI | 227.93 (70.03to548.03) | 109.42 (36.81to249.75) | | 444.41 (142.17to1102.47) | 95.98 (33.19to221.83) | 0.95 (0.72to1.22) |  | 10.32 (3.48to23.50) | 6.43 (2.31to13.64) |  | 21.78 (7.76to49.05) | 5.88 (2.18to12.37) | -0.12 (-0.21to-0.02) | |
| Low-middle SDI | 679.88 (232.16to1539.60) | 129.77 (47.51to276.28) | | 1425.61 (525.87to3215.24) | 115.78 (43.75to252.27) | 1.10 (0.85to1.40) |  | 31.73 (11.71to66.54) | 7.88 (3.11to15.69) |  | 78.06 (30.30to162.72) | 7.45 (3.00to15.08) | -0.08 (-0.15to-0.01) | |
| Middle SDI | 1103.71 (359.86to2513.29) | 126.57 (44.00to276.25) | | 2496.72 (850.68to5569.00) | 113.33 (39.76to246.54) | 1.26 (1.01to1.59) |  | 56.16 (19.97to120.75) | 8.26 (3.08to16.87) |  | 149.36 (53.17to312.41) | 7.85 (2.87to16.10) | -0.13 (-0.19to-0.07) | |
| High-middle SDI | 1419.31 (467.33to3240.83) | 151.73 (51.22to338.05) | | 2026.21 (709.42to4377.07) | 101.94 (35.87to220.16) | 0.43 (0.29to0.64) |  | 86.90 (30.02to184.01) | 10.85 (3.86to22.54) |  | 146.99 (53.63to295.04) | 7.65 (2.81to15.30) | -1.28 (-1.37to-1.19) | |
| High SDI | 1408.70 (386.77to3287.84) | 134.55 (36.78to314.84) | | 1187.24 (377.14to2623.82) | 61.08 (18.77to135.67) | -0.16 (-0.25to0.04) |  | 98.09 (29.00to214.28) | 9.53 (2.88to20.87) |  | 90.21 (29.29to190.86) | 3.98 (1.29to8.46) | -3.46 (-3.63to-3.29) | |
| Central Asia | 71.59 (23.15to169.54) | 174.87 (57.67to407.18) | | 120.26 (38.63to287.85) | 216.60 (72.36to491.58) | 0.68 (0.52to0.84) |  | 4.84 (1.70to10.66) | 12.96 (4.56to28.13) |  | 7.61 (2.60to17.06) | 16.40 (5.75to35.02) | 0.42 (0.04to0.81) |  |
| Central Europe | 239.60 (80.19to562.51) | 179.52 (61.21to417.98) | | 224.89 (80.78to503.01) | 101.18 (36.11to230.31) | -0.06 (-0.19to0.12) |  | 15.61 (5.56to34.96) | 13.16 (4.86to28.83) |  | 18.32 (6.83to38.45) | 8.19 (3.06to17.14) | -1.97 (-2.08to-1.86) | |
| Eastern Europe | 412.44 (138.36to963.69) | 166.86 (58.55to382.25) | | 537.80 (187.10to1235.81) | 151.38 (52.69to349.23) | 0.30 (0.16to0.49) |  | 28.55 (10.21to62.77) | 13.02 (4.70to28.06) |  | 42.50 (15.48to90.13) | 12.11 (4.42to25.79) | -0.31 (-0.66to0.04) |  |
| Australasia | 39.86 (10.72to92.86) | 174.23 (47.52to400.98) | | 35.23 (10.77to71.87) | 66.60 (20.16to141.03) | -0.12 (-0.27to0.19) |  | 2.66 (0.77to5.70) | 12.26 (3.61to25.76) |  | 2.94 (0.96to5.53) | 5.06 (1.63to9.61) | -3.63 (-3.82to-3.45) | |
| High-income Asia Pacific | 129.95 (30.53to318.49) | 70.87 (17.23to170.87) | | 132.67 (34.38to309.07) | 25.60 (6.27to62.21) | 0.02 (-0.14to0.32) |  | 8.43 (2.10to19.43) | 5.15 (1.35to11.65) |  | 11.72 (3.30to25.58) | 1.87 (0.50to4.13) | -3.49 (-3.72to-3.27) | |
| High-income North America | 487.75 (117.13to1206.32) | 135.03 (32.31to330.41) | | 348.77 (77.57to884.27) | 54.00 (11.84to140.93) | -0.28 (-0.46to-0.13) | | 34.92 (9.12to79.50) | 9.43 (2.47to21.43) |  | 26.43 (6.43to63.31) | 3.66 (0.87to9.00) | -3.71 (-4.01to-3.41) | |
| Southern Latin America | 15.83 (4.33to49.36) | 36.98 (10.06to113.63) | | 17.42 (4.71to49.01) | 20.67 (5.61to58.09) | 0.10 (-0.11to0.44) |  | 0.99 (0.26to2.87) | 2.56 (0.67to7.27) |  | 1.23 (0.33to3.25) | 1.43 (0.38to3.78) | -1.76 (-2.03to-1.48) | |
| Western Europe | 856.11 (238.62to1941.24) | 145.37 (40.41to332.26) | | 612.37 (191.01to1282.73) | 59.20 (17.49to129.24) | -0.28 (-0.36to-0.14) | | 60.23 (18.32to126.11) | 10.30 (3.17to21.57) |  | 53.34 (17.66to104.85) | 4.53 (1.47to8.97) | -3.33 (-3.55to-3.11) | |
| Andean Latin America | 11.25 (2.30to30.82) | 59.73 (12.33to158.21) | | 24.86 (5.55to64.71) | 45.69 (10.46to116.94) | 1.21 (0.71to2.02) |  | 0.66 (0.14to1.70) | 4.01 (0.87to10.08) |  | 1.63 (0.39to3.84) | 3.12 (0.75to7.31) | -0.76 (-0.97to-0.56) | |
| Caribbean | 56.77 (17.18to120.02) | 230.00 (71.21to472.83) | | 93.49 (29.76to192.06) | 181.00 (57.64to370.12) | 0.65 (0.40to0.99) |  | 3.41 (1.15to6.56) | 15.17 (5.16to29.05) |  | 5.75 (2.06to10.60) | 11.04 (3.97to20.39) | -1.15 (-1.37to-0.94) | |
| Central Latin America | 45.47 (10.65to126.47) | 59.58 (14.05to162.74) | | 118.10 (27.57to311.59) | 51.86 (12.24to136.06) | 1.60 (1.21to2.17) |  | 2.55 (0.60to6.67) | 3.96 (0.94to10.25) |  | 7.96 (1.99to20.20) | 3.62 (0.92to9.15) | -0.62 (-0.86to-0.37) | |
| Tropical Latin America | 275.84 (91.60to523.58) | 315.91 (115.23to575.97) | | 418.21 (170.02to740.63) | 174.80 (73.60to305.30) | 0.52 (0.34to1.00) |  | 12.68 (4.98to22.21) | 18.00 (7.65to30.59) |  | 22.10 (11.13to35.66) | 9.66 (4.97to15.27) | -2.02 (-2.15to-1.90) | |
| North Africa and Middle East | 832.66 (316.96to1677.98) | 528.28 (216.95to998.43) | | 1633.89 (646.95to3133.03) | 402.08 (167.58to728.70) | 0.96 (0.70to1.33) |  | 39.49 (16.65to73.11) | 30.78 (13.64to53.53) | | 82.36 (35.94to142.31) | 24.12 (11.23to40.28) | -0.98 (-1.05to-0.90) | |
| South Asia | 636.87 (185.46to1528.41) | 131.77 (43.37to297.85) | | 1370.43 (452.17to3320.68) | 112.02 (38.19to255.13) | 1.15 (0.80to1.66) |  | 28.84 (9.38to65.30) | 7.96 (2.74to16.83) |  | 77.34 (27.46to169.13) | 7.53 (2.78to15.63) | 0.05 (-0.14to0.25) |  |
| East Asia | 453.35 (138.94to1110.22) | 71.36 (22.89to162.43) | | 1242.53 (369.03to2924.86) | 72.87 (22.11to166.30) | 1.74 (1.13to2.51) |  | 25.81 (8.15to58.58) | 5.37 (1.81to11.43) |  | 91.19 (28.14to202.76) | 6.14 (1.92to13.28) | 0.72 (0.49to0.95) |  |
| Oceania | 4.17 (1.03to11.31) | 157.83 (44.16to400.79) | | 11.52 (2.93to30.40) | 181.24 (54.60to440.95) | 1.77 (1.25to2.42) |  | 0.17 (0.05to0.44) | 9.07 (2.80to20.91) |  | 0.50 (0.15to1.20) | 10.56 (3.42to23.66) | 0.51 (0.43to0.60) |  |
| Southeast Asia | 158.48 (39.33to423.85) | 70.74 (18.28to182.97) | | 409.68 (105.04to1025.77) | 74.31 (20.06to179.50) | 1.59 (1.21to2.11) |  | 7.94 (2.07to20.23) | 4.41 (1.21to10.85) |  | 21.74 (5.99to51.20) | 4.63 (1.31to10.72) | 0.05 (-0.04to0.15) |  |
| Central Sub-Saharan Africa | 19.15 (5.17to52.12) | 98.34 (29.94to247.89) | | 42.89 (11.76to111.70) | 95.54 (28.60to233.88) | 1.24 (0.74to1.93) |  | 0.83 (0.25to2.09) | 5.79 (1.87to13.65) |  | 2.06 (0.62to5.01) | 5.97 (1.91to13.53) | 0.02 (-0.04to0.08) |  |
| Eastern Sub-Saharan Africa | 16.97 (4.89to58.72) | 25.58 (7.28to83.45) |  | 34.08 (9.90to116.24) | 24.09 (7.07to73.72) | 1.01 (0.53to1.54) |  | 0.76 (0.22to2.47) | 1.50 (0.45to4.39) |  | 1.68 (0.49to5.02) | 1.50 (0.44to4.22) | -0.01 (-0.04to0.01) |  |
| Southern Sub-Saharan Africa | 22.73 (6.70to52.99) | 84.34 (26.30to193.42) | | 42.47 (13.29to97.37) | 83.56 (27.23to184.05) | 0.87 (0.66to1.16) |  | 1.06 (0.35to2.31) | 4.76 (1.58to10.30) |  | 2.30 (0.77to4.84) | 5.43 (1.83to11.05) | 0.77 (0.23to1.31) |  |
| Western Sub-Saharan Africa | 56.35 (15.69to155.77) | 74.71 (22.07to196.57) | | 115.11 (31.65to301.45) | 72.34 (20.85to180.61) | 1.04 (0.55to1.48) |  | 2.93 (0.88to7.52) | 4.79 (1.48to11.65) |  | 6.10 (1.80to15.02) | 4.71 (1.44to11.15) | -0.10 (-0.16to-0.03) |  |

| **Supplemental table 2. DALYs and mortality of stroke attributable to LPA in 27 GBD regions in 1990 and 2019** | | | | | | | | | | | | |  |
| --- | --- | --- | --- | --- | --- | --- | --- | --- | --- | --- | --- | --- | --- |
|  | **DALYs (95% UI)** | | | | | |  | **Mortality (95% UI)** | | | | | |
|  | **1990** | |  | **2019** | | **EAPC** |  | **1990** | |  | **2019** | | **EAPC** |
| **location** | **Number(no.×10^3^)** | **ASR per100,000** |  | **Number(no.×10^3^)** | **ASR per100,000** |  |  | **Number(no.×10^3^)** | **ASR per100,000** |  | **Number(no.×10^3^)** | **ASR per100,000** |  |
| Global | 1439.37 (240.41to3930.84) | 43.55 (7.56to117.17) | | 2409.41 (432.87to6377.62) | 31.16 (5.69to82.02) | 0.67 (0.53to0.94) | | 87.65 (15.90to231.49) | 3.09 (0.55to8.09) | | 152.39 (30.08to391.95) | 2.08 (0.41to5.33) | 0.74 (0.58to1.02) |
| Low SDI | 46.47 (7.88to135.74) | 26.46 (4.90to70.41) | | 104.37 (18.94to291.40) | 25.17 (4.97to66.78) | 1.25 (0.95to1.63) | | 2.27 (0.42to5.91) | 1.77 (0.35to4.45) | | 5.47 (1.12to14.16) | 1.69 (0.37to4.21) | 1.41 (1.03to1.91) |
| Low-middle SDI | 159.80 (29.89to425.23) | 36.47 (7.32to90.60) | | 380.32 (77.83to980.67) | 33.40 (7.26to83.02) | 1.38 (1.09to1.86) | | 8.50 (1.73to21.31) | 2.57 (0.55to6.20) | | 22.53 (4.84to55.39) | 2.34 (0.52to5.64) | 1.65 (1.29to2.18) |
| Middle SDI | 318.07 (51.64to888.91) | 40.00 (6.90to107.25) | | 798.05 (132.91to2197.51) | 37.60 (6.64to102.39) | 1.51 (1.15to1.98) | | 16.49 (2.95to44.22) | 2.66 (0.49to6.99) | | 45.54 (8.38to122.34) | 2.48 (0.47to6.52) | 1.76 (1.29to2.34) |
| High-middle SDI | 544.77 (97.24to1481.80) | 60.25 (11.38to159.34) | | 765.02 (143.53to2006.72) | 38.45 (7.27to100.71) | 0.40 (0.27to0.63) | | 34.23 (6.62to89.41) | 4.41 (0.89to11.25) | | 52.98 (10.64to135.01) | 2.75 (0.56to6.97) | 0.55 (0.38to0.79) |
| High SDI | 369.45 (50.90to1071.30) | 35.12 (4.87to101.83) | | 360.07 (53.90to991.98) | 17.44 (2.53to48.12) | -0.03 (-0.12to0.17) | | 26.11 (3.68to72.93) | 2.55 (0.36to7.12) | | 25.77 (3.97to68.45) | 1.06 (0.17to2.83) | -0.01 (-0.13to0.17) |
| Central Asia | 17.32 (3.37to51.89) | 41.73 (8.14to121.31) | | 25.69 (4.52to77.00) | 45.51 (8.54to127.74) | 0.48 (0.28to0.69) | | 1.06 (0.21to2.89) | 2.82 (0.56to7.55) | | 1.50 (0.30to4.14) | 3.27 (0.66to8.64) | 0.41 (0.20to0.61) |
| Central Europe | 79.90 (15.35to224.18) | 60.16 (11.85to166.43) | | 89.88 (18.64to236.69) | 39.78 (8.10to104.77) | 0.12 (-0.07to0.36) | | 5.05 (1.06to13.49) | 4.27 (0.91to11.14) | | 6.74 (1.45to17.40) | 2.95 (0.64to7.59) | 0.34 (0.12to0.60) |
| Eastern Europe | 182.78 (35.80to537.95) | 73.48 (14.43to212.75) | | 187.80 (38.59to518.17) | 52.47 (10.86to145.86) | 0.03 (-0.13to0.25) | | 12.18 (2.47to32.93) | 5.51 (1.14to14.65) | | 14.49 (3.10to37.85) | 4.09 (0.89to10.62) | 0.19 (-0.02to0.46) |
| Australasia | 7.49 (0.96to21.16) | 34.15 (4.49to95.82) | | 9.11 (1.32to23.19) | 16.39 (2.31to43.06) | 0.22 (-0.04to0.77) | | 0.56 (0.07to1.49) | 2.75 (0.36to7.23) | | 0.79 (0.11to1.94) | 1.30 (0.19to3.20) | 0.43 (0.12to1.13) |
| High-income Asia Pacific | 71.87 (7.75to229.02) | 41.04 (4.50to128.67) | | 83.58 (9.40to251.24) | 15.08 (1.63to45.91) | 0.16 (-0.03to0.49) | | 4.70 (0.53to14.41) | 3.05 (0.35to9.21) | | 6.28 (0.74to18.23) | 0.92 (0.11to2.66) | 0.34 (0.09to0.73) |
| High-income North America | 85.43 (10.23to254.53) | 23.21 (2.74to69.47) | | 81.41 (9.32to253.57) | 12.43 (1.36to38.85) | -0.05 (-0.32to0.22) | | 5.48 (0.67to16.06) | 1.47 (0.18to4.30) | | 5.07 (0.64to15.69) | 0.67 (0.08to2.08) | -0.08 (-0.34to0.22) |
| Southern Latin America | 5.97 (0.95to22.52) | 13.99 (2.19to53.06) | | 7.62 (1.10to24.43) | 8.94 (1.27to29.11) | 0.28 (-0.04to0.90) | | 0.36 (0.06to1.26) | 0.93 (0.14to3.20) | | 0.54 (0.08to1.70) | 0.62 (0.09to1.95) | 0.50 (0.09to1.34) |
| Western Europe | 260.67 (38.21to714.05) | 43.18 (6.37to118.74) | | 194.73 (29.67to496.39) | 17.31 (2.80to45.65) | -0.25 (-0.34to-0.10) | | 20.35 (2.90to53.61) | 3.48 (0.50to9.14) | | 17.33 (2.59to43.02) | 1.38 (0.21to3.47) | -0.15 (-0.26to0.04) |
| Andean Latin America | 2.56 (0.28to8.44) | 13.92 (1.54to45.00) | | 5.56 (0.61to17.15) | 10.37 (1.15to31.69) | 1.18 (0.52to2.30) | | 0.15 (0.02to0.49) | 0.96 (0.10to3.00) | | 0.38 (0.04to1.16) | 0.73 (0.08to2.25) | 1.46 (0.67to2.76) |
| Caribbean | 10.94 (1.61to28.40) | 45.47 (7.02to115.61) | | 21.25 (3.34to52.41) | 41.40 (6.49to101.95) | 0.94 (0.63to1.47) | | 0.70 (0.10to1.72) | 3.26 (0.50to7.81) | | 1.49 (0.23to3.45) | 2.88 (0.43to6.65) | 1.12 (0.79to1.73) |
| Central Latin America | 12.00 (1.33to40.75) | 16.03 (1.87to51.76) | | 22.16 (2.63to69.39) | 9.86 (1.17to30.91) | 0.85 (0.52to1.38) | | 0.65 (0.08to2.07) | 1.05 (0.13to3.36) | | 1.42 (0.18to4.48) | 0.66 (0.08to2.07) | 1.18 (0.73to1.82) |
| Tropical Latin America | 82.50 (12.28to197.53) | 108.75 (17.24to243.98) | | 124.11 (26.05to253.59) | 54.80 (11.78to110.74) | 0.50 (0.22to1.47) | | 4.57 (0.72to9.94) | 7.43 (1.30to15.42) | | 8.40 (1.99to16.17) | 3.84 (0.92to7.35) | 0.84 (0.48to1.87) |
| North Africa and Middle East | 126.01 (25.50to301.95) | 90.62 (19.10to204.65) | | 350.31 (68.62to814.28) | 91.71 (18.75to203.41) | 1.78 (1.37to2.28) | | 6.37 (1.41to14.00) | 5.79 (1.32to12.19) | | 17.68 (3.96to37.43) | 5.60 (1.34to11.58) | 1.77 (1.36to2.26) |
| South Asia | 113.30 (19.46to320.75) | 29.81 (5.62to78.29) | | 259.51 (50.79to712.46) | 23.27 (4.73to60.92) | 1.29 (0.87to1.98) | | 6.03 (1.16to16.08) | 2.20 (0.44to5.70) | | 16.25 (3.42to41.01) | 1.75 (0.38to4.38) | 1.70 (1.14to2.56) |
| East Asia | 274.97 (41.57to813.91) | 42.30 (6.71to113.68) | | 674.73 (105.20to1966.72) | 37.81 (5.97to106.95) | 1.45 (0.85to2.11) | | 14.16 (2.18to39.38) | 2.84 (0.46to7.69) | | 39.34 (6.14to110.56) | 2.52 (0.41to6.94) | 1.78 (0.99to2.55) |
| Oceania | 0.68 (0.09to2.05) | 30.96 (4.69to83.98) | | 1.74 (0.22to5.06) | 32.35 (5.01to86.03) | 1.56 (1.22to2.05) | | 0.03 (0.00to0.07) | 1.75 (0.28to4.63) | | 0.07 (0.01to0.19) | 1.83 (0.33to4.76) | 1.71 (1.25to2.37) |
| Southeast Asia | 67.47 (8.29to216.59) | 33.32 (4.23to102.76) | | 188.66 (24.64to593.17) | 37.15 (4.99to113.26) | 1.80 (1.28to2.39) | | 3.47 (0.45to10.45) | 2.15 (0.28to6.46) | | 10.46 (1.38to32.19) | 2.41 (0.32to7.31) | 2.01 (1.45to2.71) |
| Central Sub-Saharan Africa | 5.10 (0.69to15.38) | 31.34 (4.87to87.39) | | 12.41 (1.96to36.23) | 32.03 (5.49to88.24) | 1.43 (0.95to2.17) | | 0.22 (0.03to0.63) | 1.95 (0.33to5.41) | | 0.62 (0.11to1.68) | 2.11 (0.39to5.67) | 1.84 (1.17to2.88) |
| Eastern Sub-Saharan Africa | 6.90 (1.31to29.02) | 11.49 (2.23to44.04) | | 16.34 (3.14to66.48) | 12.46 (2.47to46.29) | 1.37 (0.97to1.87) | | 0.30 (0.06to1.09) | 0.68 (0.13to2.39) | | 0.79 (0.16to2.75) | 0.78 (0.15to2.62) | 1.63 (1.10to2.31) |
| Southern Sub-Saharan Africa | 8.48 (1.31to22.95) | 34.97 (5.95to92.33) | | 18.40 (3.13to49.35) | 39.91 (7.25to102.72) | 1.17 (0.92to1.63) | | 0.43 (0.08to1.12) | 2.19 (0.41to5.59) | | 1.06 (0.21to2.70) | 2.76 (0.55to6.93) | 1.45 (1.15to1.96) |
| Western Sub-Saharan Africa | 17.06 (2.47to54.20) | 23.63 (3.71to70.29) | | 34.40 (5.01to107.44) | 22.36 (3.59to65.07) | 1.02 (0.66to1.42) | | 0.83 (0.13to2.45) | 1.49 (0.25to4.22) | | 1.69 (0.28to4.82) | 1.44 (0.25to4.01) | 1.04 (0.62to1.54) |

**Supplemental table 3. DALYs and mortality of cardiovascular diseases attributable to LPA in** **204 countries and territories in in 1990 and 2019**

|  | **DALYs (95% UI)** | | | | | |  | **Mortality (95% UI)** | | | | | |
| --- | --- | --- | --- | --- | --- | --- | --- | --- | --- | --- | --- | --- | --- |
|  | **1990** | |  | **2019** | | **EAPC** |  | **1990** | |  | **2019** | | **EAPC** |
| **location** | **Number(no.×10^3^)** | **ASR per100,000** |  | **Number(no.×10^3^)** | **ASR per100,000** |  |  | **Number(no.×10^3^)** | **ASR per100,000** |  | **Number(no.×10^3^)** | **ASR per100,000** |  |
| Afghanistan | 51.76 (19.30to106.74) | 784.23 (323.50to1539.91) | | 80.81 (31.31to171.36) | 686.16 (309.81to1320.89) | -0.54 (-0.62to-0.47) | | 2.35 (1.00to4.45) | 43.05 (20.45to76.72) | | 3.45 (1.55to6.62) | 39.42 (18.78to67.93) | -0.37 (-0.43to-0.32) |
| Albania | 1.66 (0.63to3.84) | 95.06 (37.48to212.63) | | 3.88 (1.44to8.61) | 93.53 (34.83to207.20) | 0.33 (0.10to0.55) | | 0.11 (0.04to0.23) | 6.95 (2.78to14.44) | | 0.28 (0.11to0.62) | 7.13 (2.84to15.28) | 0.50 (0.26to0.74) |
| Algeria | 69.82 (31.07to133.85) | 773.63 (372.66to1344.73) | | 142.22 (69.09to245.04) | 509.60 (262.48to842.86) | -1.49 (-1.58to-1.39) | | 3.51 (1.62to6.17) | 55.56 (28.59to89.89) | | 8.47 (4.57to13.67) | 38.05 (21.02to59.93) | -1.26 (-1.35to-1.17) |
| American Samoa | 0.05 (0.02to0.10) | 236.41 (91.69to482.27) | | 0.11 (0.04to0.23) | 258.34 (104.36to502.69) | 0.15 (-0.00to0.30) | | 0.00 (0.00to0.00) | 13.76 (6.02to25.60) | | 0.01 (0.00to0.01) | 14.74 (6.55to25.96) | 0.15 (0.03to0.26) |
| Andorra | 0.05 (0.01to0.10) | 103.13 (35.03to217.50) | | 0.10 (0.04to0.20) | 67.99 (25.52to137.60) | -1.62 (-1.77to-1.46) | | 0.00 (0.00to0.01) | 7.64 (2.69to14.97) | | 0.01 (0.00to0.02) | 5.06 (2.08to9.36) | -1.54 (-1.70to-1.37) |
| Angola | 3.83 (1.19to8.94) | 117.77 (40.12to266.54) | | 10.84 (3.62to25.63) | 123.00 (45.96to267.49) | -0.03 (-0.13to0.07) | | 0.16 (0.05to0.37) | 6.82 (2.60to15.11) | | 0.50 (0.18to1.09) | 7.80 (3.04to16.42) | 0.30 (0.19to0.40) |
| Antigua and Barbuda | 0.11 (0.04to0.21) | 192.92 (71.60to385.01) | | 0.14 (0.05to0.26) | 150.94 (59.77to283.40) | -1.12 (-1.30to-0.94) | | 0.01 (0.00to0.01) | 13.18 (5.32to24.32) | | 0.01 (0.00to0.02) | 11.11 (4.68to19.53) | -0.86 (-1.03to-0.69) |
| Argentina | 10.23 (3.47to33.37) | 34.09 (11.50to107.88) | | 10.49 (3.57to29.14) | 19.16 (6.51to54.12) | -1.56 (-1.91to-1.21) | | 0.63 (0.21to1.85) | 2.30 (0.73to6.67) | | 0.75 (0.25to2.05) | 1.34 (0.45to3.68) | -1.33 (-1.74to-0.91) |
| Armenia | 4.37 (1.64to9.58) | 202.92 (78.20to426.95) | | 6.56 (2.46to14.08) | 162.62 (61.31to345.91) | -1.50 (-1.71to-1.29) | | 0.30 (0.12to0.61) | 15.90 (6.41to31.94) | | 0.50 (0.20to1.01) | 13.05 (5.27to26.41) | -1.54 (-1.79to-1.29) |
| Australia | 39.93 (13.53to84.22) | 211.86 (72.24to441.67) | | 36.39 (13.86to67.44) | 80.64 (29.72to154.90) | -3.85 (-4.03to-3.68) | | 2.73 (0.96to5.35) | 15.44 (5.58to29.91) | | 3.06 (1.24to5.43) | 6.16 (2.48to11.11) | -3.80 (-4.00to-3.60) |
| Austria | 23.85 (8.77to47.69) | 190.71 (70.04to392.00) | | 18.55 (7.10to35.97) | 88.00 (32.70to177.80) | -3.36 (-3.64to-3.08) | | 1.81 (0.71to3.41) | 14.80 (5.84to27.62) | | 1.72 (0.68to3.15) | 7.41 (2.88to13.91) | -2.96 (-3.23to-2.69) |
| Azerbaijan | 8.45 (3.01to19.54) | 190.37 (67.61to437.33) | | 18.82 (6.58to42.37) | 302.11 (113.88to648.75) | 1.52 (1.33to1.71) | | 0.55 (0.21to1.19) | 13.11 (5.02to27.52) | | 1.18 (0.43to2.53) | 24.81 (9.76to50.43) | 2.29 (2.07to2.51) |
| Bahamas | 0.31 (0.11to0.65) | 219.53 (78.85to437.19) | | 0.58 (0.22to1.21) | 162.76 (64.73to321.77) | -1.22 (-1.39to-1.05) | | 0.02 (0.01to0.03) | 14.02 (5.57to25.65) | | 0.03 (0.01to0.06) | 10.31 (4.44to18.65) | -1.31 (-1.52to-1.10) |
| Bahrain | 1.38 (0.52to2.70) | 913.57 (429.11to1611.56) | | 2.51 (1.05to4.74) | 345.39 (176.50to572.10) | -3.75 (-3.99to-3.50) | | 0.06 (0.03to0.10) | 57.25 (29.45to92.16) | | 0.10 (0.05to0.18) | 24.03 (13.11to37.83) | -3.13 (-3.40to-2.86) |
| Bangladesh | 46.54 (15.99to104.40) | 117.48 (42.52to255.75) | | 136.54 (50.57to300.41) | 117.90 (44.08to252.87) | 0.16 (-0.33to0.66) | | 2.58 (0.98to5.48) | 7.87 (3.06to15.97) | | 8.27 (3.21to16.94) | 8.23 (3.26to16.29) | 0.28 (-0.27to0.84) |
| Barbados | 0.73 (0.31to1.28) | 235.14 (99.06to419.60) | | 0.75 (0.33to1.34) | 152.35 (67.25to271.89) | -1.82 (-2.05to-1.59) | | 0.05 (0.02to0.09) | 17.20 (7.53to29.07) | | 0.05 (0.02to0.09) | 10.80 (4.84to18.53) | -1.88 (-2.13to-1.64) |
| Belarus | 28.69 (11.67to60.56) | 234.24 (97.13to490.09) | | 40.39 (16.60to82.73) | 241.40 (97.45to502.63) | -0.18 (-0.48to0.11) | | 2.17 (0.92to4.24) | 18.73 (7.98to36.05) | | 3.25 (1.39to6.35) | 19.21 (8.22to37.73) | -0.16 (-0.38to0.06) |
| Belgium | 30.49 (10.81to60.28) | 194.40 (67.73to387.64) | | 21.44 (8.48to39.35) | 81.04 (30.68to156.53) | -3.51 (-3.81to-3.22) | | 2.19 (0.83to4.08) | 14.12 (5.42to26.33) | | 1.82 (0.75to3.15) | 6.02 (2.43to10.61) | -3.35 (-3.57to-3.13) |
| Belize | 0.17 (0.06to0.35) | 194.12 (68.90to390.32) | | 0.37 (0.14to0.76) | 146.81 (57.26to282.31) | -1.41 (-1.88to-0.95) | | 0.01 (0.00to0.02) | 13.18 (5.01to24.38) | | 0.02 (0.01to0.04) | 9.51 (4.05to16.57) | -1.50 (-1.95to-1.04) |
| Benin | 1.76 (0.58to4.03) | 97.72 (33.32to220.08) | | 4.12 (1.37to9.85) | 98.75 (35.63to222.91) | 0.07 (0.02to0.12) | | 0.10 (0.03to0.22) | 6.13 (2.23to13.66) | | 0.22 (0.08to0.48) | 6.29 (2.43to13.95) | 0.14 (0.10to0.18) |
| Bermuda | 0.21 (0.08to0.42) | 365.40 (140.39to709.89) | | 0.18 (0.07to0.32) | 130.33 (54.15to242.01) | -3.75 (-4.05to-3.45) | | 0.01 (0.01to0.02) | 25.12 (10.49to45.06) | | 0.01 (0.01to0.02) | 9.58 (4.22to16.50) | -3.53 (-3.79to-3.26) |
| Bhutan | 0.32 (0.12to0.73) | 164.83 (67.28to347.59) | | 0.89 (0.36to1.85) | 180.69 (76.04to364.75) | 0.56 (0.42to0.69) | | 0.02 (0.01to0.03) | 10.65 (4.65to20.40) | | 0.05 (0.02to0.11) | 12.60 (5.74to23.64) | 0.77 (0.67to0.87) |
| Bolivia (Plurinational State of) | 2.35 (0.58to6.36) | 84.95 (21.29to221.72) | | 6.11 (1.59to14.90) | 79.92 (21.18to193.03) | -0.32 (-0.53to-0.11) | | 0.13 (0.03to0.33) | 5.60 (1.48to13.88) | | 0.37 (0.10to0.90) | 5.69 (1.55to13.20) | -0.07 (-0.29to0.15) |
| Bosnia and Herzegovina | 5.36 (1.87to12.65) | 158.38 (58.74to358.53) | | 7.74 (3.05to16.80) | 136.97 (54.46to299.06) | -0.93 (-1.14to-0.72) | | 0.29 (0.11to0.68) | 10.81 (4.11to23.42) | | 0.54 (0.22to1.10) | 10.24 (4.22to20.76) | -0.45 (-0.69to-0.21) |
| Botswana | 0.61 (0.19to1.42) | 125.77 (43.13to283.17) | | 1.74 (0.55to4.20) | 150.77 (51.63to342.78) | 0.38 (0.05to0.71) | | 0.03 (0.01to0.07) | 7.80 (2.86to18.15) | | 0.08 (0.03to0.19) | 9.43 (3.53to20.34) | 0.47 (0.21to0.73) |
| Brazil | 356.74 (149.02to630.01) | 434.53 (193.21to747.30) | | 538.08 (266.16to870.05) | 233.09 (118.20to375.73) | -2.05 (-2.14to-1.95) | | 17.15 (7.86to28.64) | 26.12 (12.63to41.38) | | 30.23 (16.77to46.33) | 13.69 (7.58to20.84) | -2.11 (-2.24to-1.98) |
| Brunei Darussalam | 0.18 (0.05to0.44) | 234.86 (70.96to515.78) | | 0.33 (0.09to0.81) | 151.29 (49.52to328.13) | -1.46 (-1.58to-1.35) | | 0.01 (0.00to0.02) | 15.38 (5.14to31.13) | | 0.02 (0.00to0.04) | 10.67 (3.65to21.82) | -1.23 (-1.38to-1.08) |
| Bulgaria | 24.82 (9.85to53.23) | 250.24 (102.56to531.70) | | 28.06 (11.79to59.57) | 189.97 (78.77to401.38) | -1.62 (-1.93to-1.30) | | 1.56 (0.64to3.24) | 19.61 (8.32to39.26) | | 2.17 (0.94to4.42) | 15.05 (6.52to30.56) | -1.61 (-1.94to-1.29) |
| Burkina Faso | 1.63 (0.52to4.33) | 44.16 (14.58to111.72) | | 4.38 (1.49to11.85) | 56.97 (19.46to144.39) | 0.95 (0.62to1.28) | | 0.08 (0.03to0.20) | 2.75 (0.93to6.77) | | 0.23 (0.08to0.58) | 3.63 (1.30to8.74) | 1.13 (0.82to1.44) |
| Burundi | 0.99 (0.32to2.97) | 46.86 (15.75to132.01) | | 1.51 (0.49to4.70) | 39.58 (13.43to113.86) | -0.74 (-0.80to-0.68) | | 0.05 (0.02to0.13) | 2.70 (0.92to6.90) | | 0.07 (0.02to0.20) | 2.44 (0.81to6.36) | -0.43 (-0.48to-0.37) |
| Cabo Verde | 0.18 (0.06to0.44) | 74.33 (23.72to184.21) | | 0.41 (0.14to0.96) | 98.58 (34.47to227.63) | 0.38 (0.07to0.69) | | 0.01 (0.00to0.03) | 5.00 (1.67to11.84) | | 0.03 (0.01to0.06) | 6.87 (2.51to15.87) | 0.56 (0.19to0.93) |
| Cambodia | 3.24 (0.96to8.22) | 89.16 (28.03to217.26) | | 8.25 (2.65to20.08) | 86.35 (27.81to206.08) | -0.17 (-0.23to-0.11) | | 0.16 (0.05to0.39) | 5.64 (1.83to12.64) | | 0.48 (0.16to1.13) | 6.07 (2.02to13.70) | 0.21 (0.16to0.27) |
| Cameroon | 3.17 (1.00to7.58) | 86.90 (29.24to196.30) | | 10.77 (3.53to25.84) | 106.82 (37.94to241.58) | 0.80 (0.57to1.03) | | 0.16 (0.06to0.37) | 5.65 (2.05to12.06) | | 0.53 (0.19to1.18) | 6.74 (2.55to14.40) | 0.71 (0.52to0.89) |
| Canada | 45.28 (13.84to99.70) | 142.37 (43.72to310.44) | | 49.03 (16.43to102.45) | 65.97 (21.77to140.55) | -3.14 (-3.35to-2.92) | | 3.18 (1.01to6.72) | 10.40 (3.39to21.71) | | 3.99 (1.42to7.77) | 4.92 (1.72to9.76) | -3.14 (-3.35to-2.93) |
| Central African Republic | 1.49 (0.46to3.63) | 151.76 (54.23to348.69) | | 2.75 (0.87to6.65) | 155.38 (56.55to354.05) | 0.20 (0.14to0.26) | | 0.06 (0.02to0.15) | 8.66 (3.34to19.46) | | 0.12 (0.04to0.27) | 9.21 (3.51to20.06) | 0.37 (0.30to0.44) |
| Chad | 1.74 (0.56to4.32) | 69.18 (22.61to169.49) | | 3.56 (1.10to9.10) | 74.55 (24.47to188.04) | 0.45 (0.39to0.51) | | 0.09 (0.03to0.24) | 4.39 (1.50to10.44) | | 0.18 (0.06to0.45) | 4.70 (1.66to11.31) | 0.38 (0.34to0.42) |
| Chile | 7.21 (2.17to16.50) | 82.90 (25.60to189.16) | | 10.68 (3.56to23.93) | 44.73 (15.03to100.20) | -1.96 (-2.06to-1.86) | | 0.46 (0.14to1.05) | 6.10 (1.87to14.21) | | 0.74 (0.25to1.67) | 3.12 (1.07to7.02) | -2.10 (-2.24to-1.97) |
| China | 704.55 (259.46to1607.40) | 114.27 (43.33to241.33) | | 1863.34 (691.44to3992.27) | 112.34 (43.43to234.77) | -0.07 (-0.20to0.07) | | 38.64 (14.63to81.33) | 8.28 (3.27to16.14) | | 126.99 (48.10to255.58) | 8.85 (3.51to17.36) | 0.31 (0.11to0.51) |
| Colombia | 13.10 (3.77to32.76) | 83.55 (24.37to208.62) | | 26.31 (7.44to65.83) | 48.42 (13.48to122.48) | -2.09 (-2.28to-1.91) | | 0.73 (0.21to1.84) | 5.52 (1.59to13.84) | | 1.93 (0.56to4.62) | 3.37 (0.97to8.08) | -1.90 (-2.07to-1.73) |
| Comoros | 0.06 (0.02to0.21) | 32.29 (10.72to105.90) | | 0.14 (0.05to0.43) | 31.03 (10.92to93.13) | -0.31 (-0.40to-0.22) | | 0.00 (0.00to0.01) | 1.93 (0.65to5.71) | | 0.01 (0.00to0.02) | 1.95 (0.67to5.36) | -0.10 (-0.18to-0.03) |
| Congo | 1.73 (0.54to4.14) | 187.31 (66.14to433.66) | | 3.71 (1.22to8.47) | 170.22 (63.05to369.93) | -0.40 (-0.50to-0.31) | | 0.07 (0.03to0.17) | 10.86 (4.09to22.91) | | 0.17 (0.06to0.38) | 10.67 (4.29to21.92) | -0.10 (-0.17to-0.02) |
| Cook Islands | 0.02 (0.01to0.04) | 166.70 (59.69to366.50) | | 0.04 (0.01to0.07) | 156.64 (59.73to315.08) | -0.34 (-0.46to-0.22) | | 0.00 (0.00to0.00) | 9.82 (3.70to20.01) | | 0.00 (0.00to0.00) | 9.15 (3.90to16.85) | -0.36 (-0.49to-0.24) |
| Costa Rica | 0.69 (0.21to2.03) | 42.09 (12.23to121.24) | | 1.48 (0.44to4.07) | 29.03 (8.67to79.93) | -1.77 (-2.06to-1.48) | | 0.05 (0.01to0.13) | 2.97 (0.84to8.15) | | 0.11 (0.03to0.29) | 2.10 (0.62to5.62) | -1.77 (-2.09to-1.45) |
| Côte d'Ivoire | 3.71 (1.08to8.99) | 114.30 (37.13to268.71) | | 9.18 (2.82to22.06) | 103.82 (34.56to240.47) | -0.16 (-0.32to-0.01) | | 0.16 (0.05to0.39) | 7.11 (2.46to16.36) | | 0.43 (0.14to1.00) | 6.53 (2.34to14.54) | -0.18 (-0.27to-0.08) |
| Croatia | 10.35 (4.12to23.06) | 184.17 (74.58to402.19) | | 10.18 (4.23to20.51) | 107.19 (44.51to218.62) | -1.73 (-1.90to-1.56) | | 0.72 (0.29to1.44) | 14.53 (6.09to27.88) | | 0.86 (0.37to1.67) | 9.17 (4.01to17.59) | -1.32 (-1.53to-1.12) |
| Cuba | 28.27 (10.04to54.72) | 282.25 (100.81to544.11) | | 37.09 (15.16to69.21) | 188.21 (76.90to359.86) | -1.68 (-1.92to-1.44) | | 1.85 (0.72to3.41) | 19.74 (7.83to35.96) | | 2.71 (1.22to4.70) | 12.98 (5.83to22.70) | -1.77 (-2.01to-1.52) |
| Cyprus | 1.42 (0.42to3.24) | 226.60 (71.71to493.34) | | 1.76 (0.56to3.91) | 101.13 (33.12to220.22) | -3.29 (-3.45to-3.13) | | 0.09 (0.03to0.20) | 18.19 (5.96to37.75) | | 0.13 (0.04to0.27) | 8.34 (2.97to16.85) | -3.17 (-3.32to-3.02) |
| Czechia | 34.55 (13.87to75.74) | 260.48 (106.36to572.80) | | 28.07 (11.76to57.60) | 128.47 (53.66to264.59) | -2.59 (-2.69to-2.49) | | 2.36 (0.98to4.99) | 19.27 (8.21to39.32) | | 2.37 (1.02to4.63) | 10.68 (4.56to20.86) | -2.17 (-2.31to-2.03) |
| Democratic People's Republic of Korea | 12.95 (4.46to30.63) | 109.81 (40.46to252.07) | | 33.93 (12.35to79.09) | 118.21 (44.11to267.20) | 0.39 (0.19to0.59) | | 0.70 (0.26to1.54) | 7.56 (2.87to16.00) | | 2.12 (0.80to4.57) | 8.34 (3.25to17.19) | 0.51 (0.27to0.75) |
| Democratic Republic of the Congo | 16.38 (5.33to39.00) | 126.93 (45.71to284.77) | | 36.35 (11.88to86.97) | 124.03 (45.20to279.60) | -0.16 (-0.22to-0.09) | | 0.71 (0.25to1.64) | 7.68 (2.94to16.47) | | 1.79 (0.65to4.01) | 7.88 (3.08to16.29) | 0.02 (-0.04to0.08) |
| Denmark | 17.91 (5.72to38.20) | 209.92 (66.32to457.34) | | 8.27 (2.85to16.48) | 66.34 (22.42to135.17) | -4.72 (-5.10to-4.33) | | 1.34 (0.45to2.71) | 15.37 (5.24to31.13) | | 0.71 (0.27to1.34) | 5.32 (1.95to9.97) | -4.37 (-4.69to-4.05) |
| Djibouti | 0.04 (0.01to0.13) | 37.20 (12.78to103.16) | | 0.21 (0.07to0.66) | 45.26 (15.11to126.75) | 0.75 (0.68to0.82) | | 0.00 (0.00to0.00) | 2.28 (0.80to5.99) | | 0.01 (0.00to0.03) | 2.85 (0.98to7.30) | 0.85 (0.78to0.93) |
| Dominica | 0.15 (0.06to0.30) | 200.62 (74.62to409.11) | | 0.14 (0.06to0.27) | 152.03 (60.21to289.88) | -1.03 (-1.18to-0.88) | | 0.01 (0.00to0.02) | 14.40 (5.70to27.14) | | 0.01 (0.00to0.02) | 10.98 (4.66to19.95) | -0.98 (-1.10to-0.87) |
| Dominican Republic | 8.72 (3.16to17.00) | 250.68 (98.40to464.30) | | 33.73 (12.49to65.93) | 370.09 (143.84to704.84) | 2.19 (1.90to2.47) | | 0.45 (0.19to0.80) | 15.93 (6.86to27.02) | | 1.83 (0.79to3.27) | 21.71 (9.63to38.32) | 2.08 (1.75to2.40) |
| Ecuador | 3.73 (1.01to9.08) | 76.04 (21.11to177.02) | | 10.16 (2.99to23.83) | 72.91 (21.87to167.54) | -0.15 (-0.36to0.07) | | 0.21 (0.06to0.49) | 5.22 (1.48to11.69) | | 0.63 (0.19to1.39) | 5.15 (1.61to11.05) | 0.07 (-0.17to0.31) |
| Egypt | 203.05 (79.89to409.34) | 782.03 (344.61to1466.18) | | 439.90 (177.75to864.45) | 774.32 (345.95to1418.98) | 0.05 (-0.04to0.13) | | 9.56 (4.29to17.27) | 47.00 (23.30to78.17) | | 20.17 (9.02to37.08) | 45.49 (21.97to78.15) | 0.01 (-0.11to0.12) |
| El Salvador | 1.14 (0.35to3.31) | 39.66 (12.37to112.78) | | 2.37 (0.67to6.91) | 37.51 (10.66to109.20) | -0.21 (-0.35to-0.07) | | 0.07 (0.02to0.19) | 2.56 (0.76to6.93) | | 0.18 (0.05to0.50) | 2.70 (0.72to7.47) | 0.21 (0.08to0.34) |
| Equatorial Guinea | 0.23 (0.08to0.56) | 136.91 (46.38to310.43) | | 0.52 (0.18to1.18) | 131.44 (50.56to274.09) | -0.34 (-0.52to-0.16) | | 0.01 (0.00to0.02) | 7.81 (2.79to16.89) | | 0.03 (0.01to0.06) | 8.75 (3.52to17.43) | 0.22 (0.10to0.35) |
| Eritrea | 0.31 (0.09to0.99) | 37.46 (12.09to106.46) | | 0.94 (0.31to2.99) | 44.53 (15.08to119.88) | 0.71 (0.65to0.76) | | 0.01 (0.00to0.03) | 2.10 (0.69to5.56) | | 0.04 (0.01to0.11) | 2.78 (0.97to7.10) | 1.06 (0.99to1.13) |
| Estonia | 4.75 (1.93to10.41) | 243.66 (100.39to533.53) | | 3.26 (1.38to6.67) | 102.83 (43.55to218.21) | -3.67 (-3.99to-3.36) | | 0.36 (0.15to0.74) | 19.90 (8.56to40.22) | | 0.30 (0.13to0.59) | 9.02 (3.83to17.99) | -3.39 (-3.68to-3.09) |
| Eswatini | 0.31 (0.10to0.71) | 122.63 (42.22to275.32) | | 0.76 (0.24to1.74) | 151.74 (53.18to330.18) | 0.84 (0.45to1.24) | | 0.01 (0.01to0.03) | 7.45 (2.81to15.66) | | 0.03 (0.01to0.08) | 9.24 (3.44to19.71) | 0.86 (0.52to1.20) |
| Ethiopia | 6.93 (2.05to22.19) | 37.77 (11.58to111.79) | | 10.20 (3.28to30.02) | 28.05 (9.17to77.29) | -1.28 (-1.35to-1.20) | | 0.27 (0.08to0.81) | 1.98 (0.62to5.35) | | 0.52 (0.17to1.42) | 1.76 (0.57to4.56) | -0.58 (-0.63to-0.53) |
| Fiji | 0.96 (0.31to2.36) | 300.05 (109.36to674.37) | | 1.98 (0.67to4.55) | 308.65 (113.41to651.69) | -0.21 (-0.39to-0.03) | | 0.04 (0.01to0.09) | 17.43 (6.53to35.83) | | 0.09 (0.03to0.19) | 18.53 (7.46to35.94) | -0.16 (-0.37to0.05) |
| Finland | 17.14 (5.25to37.64) | 239.31 (73.20to534.61) | | 15.49 (5.30to30.70) | 109.94 (36.55to226.31) | -3.02 (-3.27to-2.77) | | 1.16 (0.39to2.37) | 16.61 (5.62to33.97) | | 1.40 (0.53to2.63) | 8.94 (3.28to16.99) | -2.39 (-2.59to-2.19) |
| France | 129.06 (49.69to238.07) | 147.01 (55.95to276.60) | | 107.46 (45.61to188.68) | 66.04 (26.65to124.38) | -3.07 (-3.29to-2.85) | | 9.75 (4.01to17.04) | 10.87 (4.46to18.98) | | 9.65 (4.28to16.39) | 4.86 (2.14to8.24) | -3.05 (-3.23to-2.87) |
| Gabon | 0.59 (0.21to1.36) | 123.22 (46.26to275.80) | | 1.13 (0.40to2.61) | 130.23 (47.92to288.91) | 0.17 (0.01to0.34) | | 0.03 (0.01to0.07) | 7.82 (3.15to16.03) | | 0.06 (0.02to0.13) | 8.73 (3.50to17.61) | 0.36 (0.22to0.50) |
| Gambia | 0.27 (0.09to0.65) | 93.30 (31.63to216.38) | | 0.96 (0.31to2.36) | 113.97 (39.58to276.03) | 0.72 (0.60to0.84) | | 0.01 (0.00to0.03) | 6.04 (2.17to13.48) | | 0.05 (0.02to0.13) | 7.40 (2.77to16.84) | 0.78 (0.69to0.88) |
| Georgia | 13.68 (5.02to30.76) | 262.43 (103.50to572.66) | | 10.48 (4.07to22.31) | 158.80 (60.55to346.30) | -2.09 (-2.38to-1.79) | | 0.92 (0.36to1.92) | 20.57 (8.24to41.99) | | 0.83 (0.34to1.66) | 11.68 (4.70to23.78) | -2.36 (-2.69to-2.03) |
| Germany | 236.68 (72.56to523.83) | 177.67 (53.90to398.99) | | 168.89 (55.25to354.15) | 75.59 (23.68to164.59) | -3.58 (-4.01to-3.15) | | 17.89 (5.72to38.89) | 13.59 (4.38to29.39) | | 14.77 (4.95to29.94) | 6.09 (2.04to12.54) | -3.26 (-3.58to-2.94) |
| Ghana | 5.17 (1.67to11.62) | 100.66 (34.64to229.28) | | 16.10 (5.53to36.82) | 120.38 (45.20to265.65) | 0.70 (0.62to0.78) | | 0.25 (0.08to0.56) | 6.46 (2.43to14.26) | | 0.84 (0.31to1.86) | 7.93 (3.18to16.66) | 0.83 (0.75to0.92) |
| Greece | 18.68 (5.75to44.25) | 126.64 (39.54to298.40) | | 24.00 (8.02to51.29) | 81.49 (25.97to184.42) | -2.27 (-2.72to-1.83) | | 1.39 (0.45to3.10) | 10.11 (3.39to22.22) | | 2.18 (0.75to4.49) | 6.50 (2.22to13.33) | -2.19 (-2.59to-1.80) |
| Greenland | 0.05 (0.02to0.12) | 195.24 (63.35to445.80) | | 0.06 (0.02to0.14) | 99.73 (31.06to221.98) | -3.01 (-3.30to-2.73) | | 0.00 (0.00to0.01) | 13.31 (4.40to28.48) | | 0.00 (0.00to0.01) | 6.89 (2.26to14.59) | -3.09 (-3.41to-2.76) |
| Grenada | 0.22 (0.08to0.43) | 282.74 (100.25to563.15) | | 0.18 (0.07to0.36) | 185.50 (70.59to358.44) | -1.52 (-1.77to-1.26) | | 0.02 (0.01to0.03) | 19.14 (7.40to35.40) | | 0.01 (0.00to0.02) | 12.59 (5.19to23.05) | -1.54 (-1.78to-1.30) |
| Guam | 0.11 (0.04to0.27) | 199.78 (70.83to428.98) | | 0.33 (0.12to0.72) | 181.71 (64.39to390.20) | -0.05 (-0.41to0.31) | | 0.01 (0.00to0.01) | 13.76 (5.31to27.14) | | 0.02 (0.01to0.04) | 10.66 (4.16to21.17) | -0.78 (-1.24to-0.32) |
| Guatemala | 0.81 (0.31to3.66) | 24.80 (9.17to102.38) | | 1.72 (0.58to6.86) | 17.18 (5.91to62.93) | -1.67 (-1.98to-1.37) | | 0.04 (0.01to0.15) | 1.53 (0.54to5.61) | | 0.10 (0.03to0.36) | 1.30 (0.43to4.18) | -1.04 (-1.26to-0.81) |
| Guinea | 2.01 (0.63to4.88) | 68.54 (22.35to160.98) | | 3.92 (1.27to9.99) | 80.12 (26.70to197.07) | 0.89 (0.78to1.01) | | 0.11 (0.04to0.26) | 4.41 (1.51to10.03) | | 0.22 (0.08to0.53) | 5.13 (1.81to11.92) | 0.88 (0.76to1.00) |
| Guinea-Bissau | 0.44 (0.13to1.12) | 123.38 (38.73to302.79) | | 0.79 (0.23to1.97) | 128.14 (41.97to307.64) | 0.30 (0.21to0.39) | | 0.02 (0.01to0.05) | 7.13 (2.36to16.74) | | 0.04 (0.01to0.09) | 7.73 (2.76to17.47) | 0.44 (0.37to0.51) |
| Guyana | 1.43 (0.48to3.10) | 421.18 (147.82to871.80) | | 1.88 (0.65to4.07) | 336.94 (124.14to686.42) | -0.74 (-0.88to-0.59) | | 0.07 (0.03to0.15) | 25.94 (10.16to49.67) | | 0.10 (0.04to0.19) | 21.17 (8.71to39.22) | -0.76 (-0.97to-0.56) |
| Haiti | 9.26 (2.97to20.72) | 337.21 (117.76to697.32) | | 16.94 (5.71to39.78) | 289.11 (104.44to611.60) | -0.37 (-0.49to-0.24) | | 0.45 (0.16to0.94) | 21.02 (7.75to40.47) | | 0.89 (0.33to1.83) | 18.94 (7.25to36.13) | -0.23 (-0.33to-0.14) |
| Honduras | 0.74 (0.22to2.20) | 38.30 (11.77to112.08) | | 2.85 (0.86to8.11) | 55.66 (17.17to149.26) | 1.32 (1.10to1.53) | | 0.04 (0.01to0.11) | 2.41 (0.72to6.58) | | 0.18 (0.05to0.47) | 4.11 (1.26to10.63) | 1.78 (1.45to2.10) |
| Hungary | 24.18 (9.74to54.11) | 178.13 (72.69to393.22) | | 22.63 (9.49to47.74) | 110.57 (45.96to235.26) | -1.86 (-2.01to-1.71) | | 1.55 (0.64to3.37) | 12.68 (5.50to26.79) | | 1.84 (0.79to3.76) | 8.71 (3.73to17.87) | -1.55 (-1.76to-1.33) |
| Iceland | 0.61 (0.21to1.25) | 207.11 (67.97to428.26) | | 0.52 (0.19to1.01) | 84.70 (29.92to168.57) | -3.45 (-3.56to-3.34) | | 0.04 (0.02to0.09) | 14.63 (5.20to28.17) | | 0.05 (0.02to0.08) | 6.47 (2.48to11.89) | -3.17 (-3.29to-3.05) |
| India | 593.00 (200.60to1386.31) | 162.93 (62.22to348.35) | | 1224.25 (469.04to2749.13) | 126.96 (50.28to269.51) | -0.36 (-0.55to-0.16) | | 26.48 (9.87to57.67) | 10.22 (4.13to20.66) | | 72.63 (29.52to146.98) | 8.95 (3.78to17.30) | -0.25 (-0.46to-0.04) |
| Indonesia | 110.29 (34.30to258.75) | 131.77 (41.69to301.64) | | 319.20 (99.98to731.46) | 176.47 (58.93to397.94) | 1.04 (0.93to1.15) | | 5.11 (1.63to11.88) | 7.96 (2.71to17.53) | | 15.93 (5.38to35.05) | 11.31 (4.07to24.26) | 1.27 (1.15to1.40) |
| Iran (Islamic Republic of) | 108.80 (40.83to221.27) | 506.41 (215.61to939.51) | | 194.51 (81.15to369.27) | 290.88 (126.53to531.39) | -2.44 (-2.67to-2.21) | | 5.03 (2.13to9.47) | 31.88 (14.79to55.31) | | 11.80 (5.38to20.47) | 19.79 (9.30to33.41) | -2.14 (-2.37to-1.91) |
| Iraq | 54.21 (25.30to96.44) | 751.47 (364.20to1287.83) | | 130.39 (59.52to236.15) | 656.27 (324.56to1104.84) | -0.65 (-0.71to-0.58) | | 2.84 (1.44to4.64) | 44.56 (23.52to71.34) | | 6.65 (3.47to10.92) | 41.14 (22.53to64.65) | -0.40 (-0.48to-0.32) |
| Ireland | 14.62 (5.78to27.93) | 368.44 (146.32to697.40) | | 9.53 (3.99to17.00) | 124.43 (52.49to223.59) | -4.48 (-4.74to-4.21) | | 0.94 (0.40to1.65) | 25.23 (11.09to43.49) | | 0.75 (0.35to1.25) | 9.56 (4.37to15.91) | -4.00 (-4.24to-3.75) |
| Israel | 9.88 (3.26to20.69) | 210.93 (70.04to440.28) | | 8.22 (3.10to15.56) | 66.06 (24.23to127.60) | -4.71 (-4.97to-4.45) | | 0.64 (0.22to1.26) | 14.91 (5.35to28.78) | | 0.68 (0.27to1.20) | 5.08 (2.05to9.11) | -4.42 (-4.66to-4.19) |
| Italy | 167.51 (64.51to317.98) | 191.11 (73.52to365.58) | | 135.37 (53.94to244.93) | 76.77 (29.48to145.34) | -3.62 (-3.86to-3.38) | | 11.99 (4.80to21.85) | 14.33 (5.82to25.90) | | 12.67 (5.42to21.77) | 6.29 (2.59to10.96) | -3.21 (-3.39to-3.03) |
| Jamaica | 3.13 (1.31to5.61) | 170.42 (71.26to306.52) | | 4.80 (2.04to8.74) | 153.59 (65.25to282.64) | -0.29 (-0.59to0.02) | | 0.23 (0.10to0.39) | 12.48 (5.27to21.48) | | 0.36 (0.16to0.63) | 10.49 (4.52to18.55) | -0.56 (-0.86to-0.26) |
| Japan | 153.06 (48.62to337.03) | 98.67 (32.31to216.45) | | 169.43 (58.86to362.67) | 39.19 (13.20to86.29) | -3.27 (-3.44to-3.10) | | 10.53 (3.67to22.09) | 7.48 (2.63to15.63) | | 14.62 (5.10to29.39) | 2.61 (0.91to5.31) | -3.75 (-3.96to-3.55) |
| Jordan | 4.40 (1.78to8.86) | 409.73 (176.58to765.32) | | 13.83 (5.90to27.16) | 258.85 (120.40to468.76) | -2.14 (-2.41to-1.86) | | 0.22 (0.10to0.41) | 26.54 (12.33to46.23) | | 0.69 (0.32to1.22) | 16.90 (8.39to28.25) | -2.07 (-2.33to-1.80) |
| Kazakhstan | 29.99 (11.69to62.47) | 276.68 (110.77to569.98) | | 35.57 (13.97to73.35) | 260.54 (107.43to526.13) | -1.02 (-1.65to-0.38) | | 1.95 (0.81to3.82) | 20.25 (8.78to38.71) | | 2.40 (1.04to4.67) | 20.83 (9.07to39.18) | -0.58 (-1.11to-0.05) |
| Kenya | 1.57 (0.51to4.76) | 22.58 (7.77to65.26) | | 5.06 (1.68to15.15) | 27.57 (9.51to76.93) | 0.91 (0.76to1.05) | | 0.08 (0.03to0.21) | 1.42 (0.50to3.69) | | 0.24 (0.08to0.67) | 1.72 (0.61to4.43) | 0.84 (0.72to0.96) |
| Kiribati | 0.11 (0.04to0.27) | 322.96 (114.20to723.48) | | 0.21 (0.07to0.52) | 334.43 (123.01to747.26) | -0.06 (-0.20to0.09) | | 0.00 (0.00to0.01) | 16.04 (6.35to33.52) | | 0.01 (0.00to0.02) | 18.16 (7.12to36.23) | 0.29 (0.17to0.42) |
| Kuwait | 3.20 (1.40to5.98) | 515.84 (263.78to861.40) | | 9.21 (4.04to16.65) | 329.11 (168.01to542.21) | -1.24 (-1.61to-0.86) | | 0.13 (0.07to0.22) | 29.12 (16.44to44.63) | | 0.39 (0.20to0.64) | 18.35 (10.48to28.35) | -1.27 (-1.61to-0.93) |
| Kyrgyzstan | 4.88 (1.95to10.69) | 170.69 (67.99to370.12) | | 7.75 (2.90to16.79) | 211.20 (82.29to451.36) | 0.85 (0.39to1.31) | | 0.33 (0.14to0.69) | 12.07 (5.02to24.85) | | 0.56 (0.22to1.18) | 16.89 (6.89to34.14) | 1.45 (0.90to2.00) |
| Lao People's Democratic Republic | 1.87 (0.56to4.88) | 109.49 (34.10to265.36) | | 3.89 (1.18to9.71) | 111.37 (35.49to271.00) | -0.27 (-0.46to-0.09) | | 0.09 (0.03to0.22) | 6.73 (2.11to15.78) | | 0.21 (0.07to0.51) | 7.59 (2.39to17.31) | 0.14 (-0.01to0.28) |
| Latvia | 12.59 (5.37to25.60) | 356.74 (153.79to722.38) | | 11.53 (5.28to21.55) | 247.05 (112.66to474.73) | -1.55 (-1.74to-1.37) | | 0.94 (0.42to1.77) | 28.18 (12.54to51.99) | | 0.99 (0.47to1.75) | 20.40 (9.60to36.96) | -1.32 (-1.47to-1.18) |
| Lebanon | 14.04 (5.66to27.04) | 680.16 (300.43to1260.79) | | 26.86 (11.91to46.91) | 518.01 (229.50to898.09) | -0.71 (-0.98to-0.44) | | 0.71 (0.32to1.26) | 40.96 (19.01to70.93) | | 1.59 (0.76to2.61) | 31.72 (15.09to51.78) | -0.64 (-0.84to-0.45) |
| Lesotho | 0.56 (0.18to1.26) | 64.86 (21.82to149.47) | | 1.16 (0.36to2.96) | 107.17 (36.82to257.50) | 2.24 (1.98to2.50) | | 0.03 (0.01to0.07) | 4.05 (1.44to9.06) | | 0.06 (0.02to0.13) | 6.67 (2.48to14.68) | 2.24 (1.99to2.49) |
| Liberia | 1.13 (0.37to2.66) | 115.93 (39.70to261.91) | | 1.91 (0.62to4.51) | 109.61 (37.76to253.18) | 0.02 (-0.16to0.19) | | 0.06 (0.02to0.13) | 7.36 (2.65to16.39) | | 0.10 (0.03to0.23) | 7.03 (2.62to15.44) | 0.08 (-0.08to0.24) |
| Libya | 7.61 (3.25to13.98) | 431.87 (200.75to771.98) | | 21.37 (9.77to38.51) | 433.35 (209.40to750.34) | 0.12 (-0.03to0.26) | | 0.40 (0.20to0.69) | 25.61 (12.91to42.68) | | 1.07 (0.55to1.81) | 25.07 (13.04to41.03) | 0.12 (-0.04to0.29) |
| Lithuania | 15.74 (6.80to29.97) | 348.97 (150.45to667.73) | | 17.38 (8.06to32.12) | 256.71 (118.25to492.99) | -1.10 (-1.28to-0.91) | | 1.21 (0.52to2.19) | 27.81 (12.12to50.23) | | 1.53 (0.73to2.68) | 21.66 (10.31to38.23) | -0.86 (-1.03to-0.68) |
| Luxembourg | 1.07 (0.35to2.25) | 197.16 (65.77to416.83) | | 0.72 (0.25to1.41) | 64.82 (21.84to131.42) | -4.20 (-4.45to-3.94) | | 0.08 (0.03to0.15) | 14.68 (5.26to29.10) | | 0.06 (0.02to0.11) | 5.06 (1.80to9.47) | -3.95 (-4.16to-3.74) |
| Madagascar | 2.05 (0.66to6.27) | 45.08 (15.24to127.38) | | 4.47 (1.38to14.09) | 49.83 (16.70to139.57) | 0.27 (0.14to0.40) | | 0.10 (0.03to0.27) | 2.72 (0.96to7.07) | | 0.20 (0.07to0.56) | 3.07 (1.05to7.73) | 0.35 (0.23to0.48) |
| Malawi | 1.42 (0.46to3.92) | 43.56 (14.97to113.66) | | 2.68 (0.90to7.25) | 43.40 (15.33to109.83) | 0.13 (0.03to0.22) | | 0.06 (0.02to0.17) | 2.58 (0.91to6.22) | | 0.14 (0.05to0.33) | 2.73 (0.99to6.54) | 0.28 (0.19to0.37) |
| Malaysia | 19.06 (6.11to40.38) | 229.53 (78.55to474.88) | | 56.23 (18.94to120.42) | 227.32 (80.67to463.14) | -0.30 (-0.49to-0.11) | | 0.95 (0.34to1.91) | 13.20 (4.85to25.83) | | 2.77 (1.04to5.53) | 13.17 (5.18to24.96) | -0.55 (-0.86to-0.23) |
| Maldives | 0.22 (0.06to0.53) | 293.91 (90.57to658.82) | | 0.40 (0.13to0.92) | 144.92 (49.36to315.42) | -3.11 (-3.32to-2.90) | | 0.01 (0.00to0.02) | 17.49 (5.91to36.72) | | 0.02 (0.01to0.05) | 9.71 (3.60to19.47) | -2.62 (-2.80to-2.44) |
| Mali | 2.34 (0.73to5.95) | 70.98 (23.43to174.73) | | 4.77 (1.56to11.95) | 68.23 (23.43to165.46) | -0.14 (-0.20to-0.08) | | 0.12 (0.04to0.30) | 4.75 (1.61to11.06) | | 0.27 (0.09to0.64) | 4.68 (1.67to11.13) | -0.02 (-0.08to0.03) |
| Malta | 1.77 (0.77to3.11) | 431.62 (192.62to750.02) | | 1.82 (0.89to2.95) | 188.62 (88.90to317.29) | -2.95 (-3.04to-2.86) | | 0.11 (0.05to0.18) | 29.01 (14.13to47.25) | | 0.14 (0.07to0.22) | 13.54 (6.88to21.07) | -2.68 (-2.77to-2.59) |
| Marshall Islands | 0.05 (0.02to0.12) | 342.75 (122.38to749.06) | | 0.12 (0.04to0.30) | 395.57 (139.80to859.91) | 0.41 (0.21to0.61) | | 0.00 (0.00to0.00) | 19.69 (7.58to39.91) | | 0.00 (0.00to0.01) | 22.24 (8.65to44.14) | 0.41 (0.27to0.56) |
| Mauritania | 2.88 (1.18to5.33) | 310.49 (131.30to562.25) | | 4.19 (1.86to7.72) | 219.74 (100.03to392.37) | -1.31 (-1.44to-1.18) | | 0.14 (0.06to0.25) | 17.71 (8.03to31.06) | | 0.22 (0.10to0.39) | 13.55 (6.49to23.58) | -1.01 (-1.14to-0.89) |
| Mauritius | 2.10 (0.64to4.72) | 311.79 (99.57to685.83) | | 2.23 (0.74to4.77) | 137.19 (47.72to290.69) | -3.74 (-4.15to-3.32) | | 0.10 (0.03to0.22) | 18.25 (6.45to37.92) | | 0.13 (0.05to0.27) | 8.75 (3.33to17.10) | -3.38 (-3.76to-3.01) |
| Mexico | 35.23 (10.09to84.18) | 90.66 (26.40to214.31) | | 90.48 (25.51to210.85) | 82.27 (23.65to187.48) | -0.57 (-0.84to-0.30) | | 1.95 (0.58to4.46) | 6.09 (1.86to13.81) | | 5.85 (1.77to12.87) | 5.75 (1.76to12.47) | -0.66 (-1.00to-0.32) |
| Micronesia (Federated States of) | 0.16 (0.05to0.38) | 373.07 (132.57to837.12) | | 0.27 (0.08to0.66) | 431.92 (160.69to931.42) | 0.38 (0.20to0.56) | | 0.01 (0.00to0.01) | 20.51 (8.40to42.06) | | 0.01 (0.00to0.02) | 24.70 (10.42to47.74) | 0.55 (0.41to0.68) |
| Monaco | 0.16 (0.06to0.31) | 198.78 (73.05to404.81) | | 0.12 (0.05to0.21) | 108.39 (43.69to205.65) | -2.24 (-2.48to-2.00) | | 0.01 (0.01to0.02) | 14.76 (5.88to27.18) | | 0.01 (0.00to0.02) | 8.19 (3.50to14.57) | -2.17 (-2.39to-1.94) |
| Mongolia | 1.83 (0.59to4.54) | 212.80 (72.72to503.60) | | 2.97 (0.99to7.78) | 184.23 (66.96to424.98) | -1.19 (-1.58to-0.80) | | 0.11 (0.04to0.25) | 15.48 (5.58to34.28) | | 0.17 (0.06to0.39) | 14.54 (5.53to30.25) | -0.75 (-1.13to-0.36) |
| Montenegro | 0.62 (0.23to1.42) | 108.24 (41.26to239.10) | | 1.12 (0.43to2.49) | 120.92 (47.59to264.08) | 0.70 (0.56to0.83) | | 0.04 (0.02to0.08) | 7.67 (3.06to15.50) | | 0.08 (0.03to0.16) | 9.01 (3.67to18.81) | 0.97 (0.79to1.16) |
| Morocco | 75.05 (31.29to141.74) | 617.02 (285.73to1091.95) | | 174.75 (80.34to313.05) | 630.61 (313.47to1089.66) | -0.01 (-0.16to0.15) | | 3.74 (1.78to6.50) | 37.40 (18.79to62.60) | | 9.41 (4.85to15.73) | 40.08 (21.29to64.72) | 0.14 (-0.09to0.38) |
| Mozambique | 1.74 (0.56to5.14) | 34.89 (11.84to96.31) | | 4.37 (1.39to12.89) | 46.75 (15.50to130.91) | 1.51 (1.32to1.69) | | 0.08 (0.03to0.22) | 2.09 (0.74to5.41) | | 0.21 (0.07to0.56) | 2.78 (0.97to7.26) | 1.40 (1.25to1.56) |
| Myanmar | 24.50 (7.37to60.75) | 125.14 (39.79to296.81) | | 40.45 (13.03to95.15) | 104.30 (34.02to239.09) | -0.77 (-0.82to-0.71) | | 1.21 (0.38to2.86) | 7.82 (2.56to17.64) | | 2.43 (0.80to5.45) | 7.27 (2.55to16.18) | -0.35 (-0.40to-0.31) |
| Namibia | 1.20 (0.42to2.54) | 182.86 (66.73to380.89) | | 2.39 (0.89to4.83) | 185.84 (70.80to362.39) | -0.19 (-0.47to0.09) | | 0.06 (0.02to0.12) | 10.70 (4.24to21.21) | | 0.13 (0.05to0.24) | 11.25 (4.46to21.61) | -0.06 (-0.30to0.19) |
| Nauru | 0.01 (0.00to0.03) | 372.53 (131.99to839.96) | | 0.02 (0.01to0.04) | 445.02 (161.16to982.67) | 0.43 (0.16to0.71) | | 0.00 (0.00to0.00) | 21.17 (8.02to43.87) | | 0.00 (0.00to0.00) | 25.00 (10.02to50.12) | 0.46 (0.24to0.68) |
| Nepal | 7.46 (2.58to18.03) | 97.18 (36.31to218.92) | | 21.16 (7.30to50.57) | 112.04 (40.96to256.70) | 0.49 (0.33to0.65) | | 0.36 (0.14to0.82) | 6.12 (2.44to13.05) | | 1.22 (0.46to2.65) | 7.71 (3.12to15.98) | 0.80 (0.66to0.95) |
| Netherlands | 17.46 (4.72to43.05) | 85.59 (23.36to208.36) | | 12.90 (3.78to30.71) | 35.06 (10.22to82.88) | -3.78 (-4.29to-3.26) | | 1.26 (0.34to3.01) | 6.20 (1.68to14.76) | | 1.08 (0.33to2.40) | 2.78 (0.85to6.26) | -3.28 (-3.73to-2.83) |
| New Zealand | 7.41 (2.12to17.08) | 192.04 (56.24to441.12) | | 7.95 (2.83to15.37) | 95.72 (33.18to189.82) | -2.58 (-2.80to-2.37) | | 0.48 (0.15to1.02) | 12.95 (4.19to27.43) | | 0.67 (0.26to1.23) | 7.46 (2.84to13.89) | -2.13 (-2.28to-1.97) |
| Nicaragua | 0.47 (0.14to1.38) | 34.74 (10.75to98.50) | | 1.80 (0.53to5.02) | 51.15 (14.94to143.06) | 0.94 (0.52to1.35) | | 0.03 (0.01to0.08) | 2.40 (0.71to6.38) | | 0.13 (0.03to0.35) | 4.17 (1.11to11.13) | 1.47 (0.95to2.00) |
| Niger | 2.11 (0.63to5.03) | 94.54 (30.27to219.89) | | 5.49 (1.70to13.54) | 88.29 (30.46to207.94) | -0.22 (-0.32to-0.13) | | 0.10 (0.03to0.23) | 5.97 (2.09to13.38) | | 0.27 (0.09to0.65) | 5.74 (2.11to12.84) | -0.07 (-0.16to0.01) |
| Nigeria | 38.09 (12.13to94.76) | 101.36 (33.83to243.25) | | 64.27 (20.79to154.66) | 88.73 (30.92to205.18) | -0.69 (-0.86to-0.53) | | 2.00 (0.68to4.76) | 6.52 (2.37to14.83) | | 3.42 (1.18to7.84) | 5.86 (2.14to12.90) | -0.58 (-0.73to-0.44) |
| Niue | 0.01 (0.00to0.01) | 261.70 (99.67to550.96) | | 0.01 (0.00to0.01) | 283.31 (106.80to600.82) | 0.07 (-0.08to0.21) | | 0.00 (0.00to0.00) | 16.28 (6.67to31.76) | | 0.00 (0.00to0.00) | 17.04 (7.10to33.03) | -0.03 (-0.15to0.10) |
| North Macedonia | 3.19 (1.26to7.02) | 206.10 (84.46to440.18) | | 5.31 (2.07to11.34) | 208.32 (86.38to440.31) | -0.13 (-0.41to0.15) | | 0.20 (0.08to0.41) | 15.04 (6.41to31.09) | | 0.36 (0.15to0.77) | 17.19 (7.50to35.97) | 0.46 (0.11to0.81) |
| Northern Mariana Islands | 0.03 (0.01to0.07) | 189.72 (75.21to382.91) | | 0.09 (0.03to0.22) | 211.87 (84.24to442.08) | 0.40 (0.33to0.46) | | 0.00 (0.00to0.00) | 11.46 (4.75to21.93) | | 0.00 (0.00to0.01) | 12.49 (5.30to24.00) | 0.31 (0.25to0.36) |
| Norway | 15.44 (4.97to32.24) | 215.74 (67.55to462.83) | | 7.74 (2.88to15.09) | 72.48 (25.89to145.93) | -4.09 (-4.22to-3.97) | | 1.10 (0.38to2.19) | 14.52 (5.08to29.00) | | 0.66 (0.26to1.20) | 5.47 (2.08to10.17) | -3.75 (-3.90to-3.60) |
| Oman | 4.93 (1.88to10.32) | 886.32 (390.64to1689.89) | | 9.14 (4.07to16.57) | 722.07 (375.70to1173.20) | -0.55 (-0.69to-0.40) | | 0.22 (0.09to0.43) | 53.74 (25.86to95.24) | | 0.42 (0.21to0.70) | 49.40 (27.21to76.03) | -0.10 (-0.29to0.09) |
| Pakistan | 102.84 (37.55to224.95) | 195.88 (76.22to411.62) | | 247.11 (87.32to549.87) | 250.19 (97.12to515.07) | 0.61 (0.27to0.94) | | 5.42 (2.20to10.89) | 11.86 (5.02to22.92) | | 11.42 (4.52to23.35) | 14.86 (6.36to28.23) | 0.62 (0.35to0.89) |
| Palau | 0.02 (0.01to0.06) | 281.27 (99.90to625.28) | | 0.06 (0.02to0.14) | 306.64 (117.82to664.26) | 0.20 (0.11to0.29) | | 0.00 (0.00to0.00) | 16.05 (6.16to33.26) | | 0.00 (0.00to0.01) | 17.37 (7.26to34.12) | 0.16 (0.09to0.24) |
| Palestine | 4.79 (2.10to9.25) | 612.08 (282.83to1118.98) | | 9.10 (4.01to16.52) | 467.60 (227.59to804.92) | -1.23 (-1.40to-1.05) | | 0.27 (0.13to0.47) | 38.36 (18.97to65.19) | | 0.48 (0.24to0.82) | 30.99 (16.08to50.65) | -0.98 (-1.19to-0.77) |
| Panama | 0.49 (0.16to1.43) | 35.32 (11.20to100.98) | | 0.97 (0.29to2.63) | 22.99 (6.87to62.25) | -1.47 (-1.66to-1.27) | | 0.03 (0.01to0.09) | 2.52 (0.76to6.76) | | 0.08 (0.02to0.20) | 1.73 (0.51to4.51) | -1.33 (-1.52to-1.15) |
| Papua New Guinea | 2.12 (0.61to5.57) | 134.54 (43.46to327.41) | | 7.16 (2.09to18.61) | 173.59 (56.06to416.86) | 0.98 (0.84to1.13) | | 0.08 (0.03to0.22) | 7.57 (2.54to16.68) | | 0.29 (0.09to0.72) | 10.08 (3.75to22.06) | 1.11 (0.97to1.25) |
| Paraguay | 1.59 (0.47to3.98) | 78.87 (22.87to193.73) | | 4.24 (1.22to10.41) | 81.06 (23.51to197.80) | 0.18 (-0.09to0.45) | | 0.11 (0.03to0.24) | 5.60 (1.67to12.98) | | 0.28 (0.08to0.65) | 5.44 (1.61to12.71) | -0.04 (-0.31to0.23) |
| Peru | 7.72 (2.03to19.60) | 69.91 (18.78to173.26) | | 14.15 (4.08to32.88) | 43.50 (12.49to101.37) | -1.52 (-1.82to-1.22) | | 0.47 (0.13to1.15) | 4.73 (1.28to11.32) | | 1.00 (0.30to2.18) | 3.02 (0.90to6.54) | -1.43 (-1.70to-1.16) |
| Philippines | 8.62 (2.68to24.88) | 39.47 (12.40to102.79) | | 38.38 (11.65to110.59) | 56.14 (17.36to152.84) | 1.88 (1.46to2.29) | | 0.46 (0.14to1.20) | 2.99 (0.92to7.37) | | 1.93 (0.58to5.20) | 3.51 (1.06to9.06) | 1.22 (0.86to1.59) |
| Poland | 119.37 (44.23to254.00) | 290.78 (110.23to611.66) | | 92.28 (36.80to182.36) | 124.81 (49.67to249.58) | -3.32 (-3.46to-3.18) | | 7.50 (3.03to15.03) | 19.99 (8.17to39.52) | | 7.41 (3.15to14.14) | 9.64 (4.08to18.53) | -2.95 (-3.07to-2.83) |
| Portugal | 34.13 (12.19to68.14) | 265.63 (98.91to524.89) | | 25.62 (10.14to47.26) | 92.41 (35.72to173.50) | -4.42 (-4.82to-4.02) | | 2.38 (0.90to4.64) | 20.44 (7.81to38.76) | | 2.20 (0.90to3.93) | 7.08 (2.83to12.74) | -4.38 (-4.79to-3.97) |
| Puerto Rico | 8.25 (3.19to15.62) | 234.90 (91.91to444.94) | | 7.45 (3.20to14.05) | 98.41 (40.11to194.51) | -3.23 (-3.41to-3.05) | | 0.53 (0.22to0.94) | 16.22 (6.82to28.41) | | 0.55 (0.24to0.96) | 6.23 (2.68to11.15) | -3.61 (-3.79to-3.43) |
| Qatar | 0.76 (0.29to1.48) | 855.71 (401.93to1455.74) | | 2.74 (1.07to5.20) | 515.40 (275.70to828.36) | -1.98 (-2.25to-1.71) | | 0.03 (0.01to0.05) | 54.72 (27.63to88.19) | | 0.10 (0.04to0.17) | 39.47 (22.04to60.85) | -1.28 (-1.56to-0.99) |
| Republic of Korea | 45.20 (14.13to102.34) | 198.67 (65.98to431.42) | | 42.21 (14.48to92.95) | 50.51 (17.24to109.91) | -4.89 (-5.08to-4.69) | | 2.41 (0.81to5.19) | 14.25 (5.06to28.74) | | 3.09 (1.09to6.38) | 3.96 (1.40to8.03) | -4.60 (-4.81to-4.39) |
| Republic of Moldova | 9.00 (3.48to19.04) | 270.16 (109.88to550.00) | | 11.28 (4.61to23.20) | 193.38 (78.96to399.44) | -1.47 (-1.71to-1.23) | | 0.64 (0.26to1.29) | 22.69 (9.35to44.20) | | 0.90 (0.37to1.77) | 15.59 (6.43to30.44) | -1.68 (-1.93to-1.43) |
| Romania | 58.57 (24.47to126.85) | 250.84 (107.74to529.71) | | 73.24 (32.84to142.45) | 181.59 (79.96to368.75) | -1.56 (-1.77to-1.35) | | 3.86 (1.66to7.80) | 19.62 (8.53to38.88) | | 5.90 (2.69to11.05) | 14.37 (6.56to26.98) | -1.43 (-1.63to-1.23) |
| Russian Federation | 395.40 (158.58to867.75) | 251.59 (105.90to545.27) | | 480.63 (206.13to972.41) | 200.07 (85.92to407.34) | -0.89 (-1.37to-0.40) | | 26.42 (11.13to54.14) | 19.18 (8.31to37.67) | | 38.17 (17.12to73.05) | 16.11 (7.27to30.71) | -0.64 (-1.13to-0.15) |
| Rwanda | 1.02 (0.32to2.87) | 41.44 (13.48to110.37) | | 1.48 (0.50to4.20) | 31.18 (10.64to81.77) | -1.51 (-1.72to-1.29) | | 0.05 (0.02to0.13) | 2.47 (0.85to6.41) | | 0.08 (0.03to0.19) | 2.09 (0.72to5.16) | -0.91 (-1.07to-0.76) |
| Saint Kitts and Nevis | 0.15 (0.06to0.30) | 424.89 (155.38to821.17) | | 0.11 (0.04to0.23) | 211.75 (83.78to407.58) | -2.34 (-2.57to-2.11) | | 0.01 (0.00to0.02) | 29.43 (11.58to52.27) | | 0.01 (0.00to0.01) | 15.05 (6.12to26.98) | -1.95 (-2.17to-1.74) |
| Saint Lucia | 0.20 (0.07to0.38) | 256.96 (100.05to478.12) | | 0.28 (0.11to0.53) | 135.00 (53.35to255.20) | -2.69 (-3.10to-2.28) | | 0.01 (0.01to0.02) | 19.54 (8.07to35.09) | | 0.02 (0.01to0.03) | 9.85 (4.14to18.14) | -2.97 (-3.40to-2.54) |
| Saint Vincent and the Grenadines | 0.19 (0.07to0.37) | 285.37 (108.46to543.60) | | 0.27 (0.11to0.52) | 218.12 (85.53to408.15) | -0.83 (-1.14to-0.52) | | 0.01 (0.00to0.02) | 20.43 (8.09to37.10) | | 0.02 (0.01to0.03) | 15.94 (6.71to27.52) | -0.70 (-1.03to-0.37) |
| Samoa | 0.23 (0.09to0.48) | 288.53 (116.46to589.39) | | 0.41 (0.16to0.88) | 304.24 (123.90to623.78) | 0.04 (-0.07to0.15) | | 0.01 (0.00to0.02) | 17.33 (7.50to32.75) | | 0.02 (0.01to0.04) | 18.15 (7.93to34.19) | 0.04 (-0.04to0.11) |
| San Marino | 0.04 (0.01to0.07) | 114.25 (42.24to223.21) | | 0.06 (0.02to0.11) | 73.51 (26.68to151.44) | -1.53 (-1.66to-1.40) | | 0.00 (0.00to0.01) | 8.86 (3.46to16.21) | | 0.00 (0.00to0.01) | 5.67 (2.21to11.09) | -1.54 (-1.65to-1.44) |
| Sao Tome and Principe | 0.05 (0.02to0.11) | 88.64 (30.74to202.84) | | 0.11 (0.04to0.24) | 120.64 (41.94to276.00) | 0.96 (0.85to1.07) | | 0.00 (0.00to0.01) | 5.93 (2.20to13.16) | | 0.01 (0.00to0.01) | 8.17 (3.04to17.26) | 1.11 (1.04to1.17) |
| Saudi Arabia | 39.76 (19.06to69.03) | 728.99 (373.70to1192.37) | | 155.08 (77.55to250.82) | 790.15 (457.57to1212.79) | 1.03 (0.65to1.42) | | 1.80 (0.94to2.90) | 42.04 (23.18to64.58) | | 5.31 (2.91to8.36) | 41.46 (25.78to60.63) | 0.23 (0.00to0.47) |
| Senegal | 3.80 (1.20to8.57) | 133.06 (45.40to296.42) | | 8.01 (2.65to18.67) | 120.77 (42.54to272.69) | -0.40 (-0.50to-0.30) | | 0.19 (0.06to0.41) | 8.10 (2.92to17.60) | | 0.42 (0.15to0.94) | 7.61 (2.80to16.37) | -0.27 (-0.35to-0.19) |
| Serbia | 20.15 (8.21to42.72) | 208.91 (86.75to436.81) | | 26.14 (10.96to55.02) | 176.41 (75.33to370.19) | -0.90 (-1.11to-0.70) | | 1.38 (0.57to2.83) | 15.78 (6.68to31.87) | | 1.98 (0.85to4.03) | 14.93 (6.59to30.03) | -0.44 (-0.61to-0.27) |
| Seychelles | 0.08 (0.02to0.17) | 135.11 (42.91to312.39) | | 0.10 (0.03to0.24) | 105.39 (35.13to236.65) | -1.04 (-1.16to-0.91) | | 0.00 (0.00to0.01) | 8.52 (2.96to19.37) | | 0.01 (0.00to0.01) | 6.79 (2.35to14.81) | -0.84 (-0.98to-0.70) |
| Sierra Leone | 1.84 (0.57to4.38) | 106.52 (34.51to245.89) | | 3.32 (1.07to8.04) | 105.99 (36.64to251.11) | 0.23 (0.11to0.35) | | 0.10 (0.03to0.23) | 6.74 (2.34to14.98) | | 0.17 (0.06to0.41) | 6.71 (2.46to15.19) | 0.23 (0.12to0.35) |
| Singapore | 3.38 (0.93to7.90) | 173.89 (50.69to395.67) | | 4.28 (1.33to9.42) | 58.10 (18.88to124.46) | -4.05 (-4.18to-3.92) | | 0.18 (0.05to0.39) | 10.81 (3.53to22.69) | | 0.27 (0.09to0.57) | 3.93 (1.36to8.03) | -3.73 (-3.88to-3.57) |
| Slovakia | 13.90 (5.16to30.86) | 244.14 (91.09to535.33) | | 13.50 (5.35to28.49) | 150.41 (59.82to316.74) | -1.75 (-1.98to-1.52) | | 0.90 (0.36to1.88) | 17.12 (6.98to35.55) | | 1.05 (0.43to2.13) | 11.98 (5.00to24.32) | -1.22 (-1.41to-1.04) |
| Slovenia | 2.77 (1.09to6.13) | 117.61 (46.61to259.99) | | 2.62 (1.08to5.48) | 52.89 (21.31to112.79) | -3.15 (-3.43to-2.86) | | 0.19 (0.08to0.40) | 8.63 (3.55to17.91) | | 0.22 (0.09to0.44) | 4.06 (1.69to8.14) | -2.99 (-3.24to-2.74) |
| Solomon Islands | 0.50 (0.13to1.31) | 377.59 (111.39to926.74) | | 1.33 (0.38to3.45) | 446.81 (144.85to1068.82) | 0.51 (0.37to0.66) | | 0.02 (0.00to0.04) | 18.81 (6.20to43.11) | | 0.05 (0.02to0.12) | 23.53 (8.38to51.44) | 0.69 (0.56to0.81) |
| Somalia | 0.89 (0.28to2.93) | 41.57 (13.93to122.03) | | 2.41 (0.76to8.43) | 43.47 (14.87to129.61) | 0.47 (0.36to0.57) | | 0.04 (0.01to0.11) | 2.37 (0.82to6.52) | | 0.10 (0.03to0.31) | 2.59 (0.90to6.99) | 0.68 (0.55to0.81) |
| South Africa | 26.58 (9.98to54.96) | 129.81 (50.92to258.33) | | 50.33 (20.15to100.96) | 126.68 (51.66to246.12) | 0.27 (-0.38to0.92) | | 1.27 (0.52to2.44) | 7.35 (3.13to14.04) | | 2.84 (1.22to5.38) | 8.43 (3.68to15.88) | 0.88 (0.24to1.53) |
| South Sudan | 0.69 (0.23to1.99) | 33.89 (11.50to94.35) | | 1.06 (0.34to3.15) | 33.66 (10.96to92.46) | 0.09 (0.01to0.17) | | 0.03 (0.01to0.09) | 2.08 (0.71to5.29) | | 0.05 (0.02to0.14) | 2.09 (0.71to5.17) | 0.17 (0.08to0.26) |
| Spain | 83.68 (28.08to174.98) | 158.15 (53.55to330.08) | | 72.64 (26.57to138.58) | 64.75 (22.44to132.25) | -3.48 (-3.70to-3.26) | | 5.97 (2.11to11.58) | 11.79 (4.19to22.67) | | 6.27 (2.41to11.37) | 4.75 (1.77to8.73) | -3.49 (-3.69to-3.29) |
| Sri Lanka | 9.52 (2.87to24.55) | 109.62 (33.64to281.58) | | 16.29 (5.01to38.74) | 73.22 (23.51to170.32) | -0.84 (-1.00to-0.67) | | 0.54 (0.17to1.39) | 7.74 (2.48to18.14) | | 1.01 (0.32to2.40) | 5.24 (1.66to11.89) | -0.62 (-0.83to-0.40) |
| Sudan | 108.14 (55.29to172.20) | 1200.60 (651.50to1857.80) | | 178.82 (94.63to294.59) | 979.12 (556.55to1534.28) | -0.82 (-0.86to-0.78) | | 4.70 (2.63to7.15) | 63.88 (38.02to94.67) | | 8.14 (4.77to12.43) | 54.47 (33.18to81.52) | -0.67 (-0.71to-0.63) |
| Suriname | 0.65 (0.22to1.34) | 264.65 (92.93to532.81) | | 1.15 (0.42to2.27) | 200.70 (76.10to388.43) | -1.09 (-1.46to-0.73) | | 0.04 (0.01to0.07) | 16.83 (6.54to31.24) | | 0.07 (0.03to0.12) | 12.36 (4.99to22.18) | -1.17 (-1.58to-0.76) |
| Sweden | 28.70 (8.55to63.20) | 175.27 (51.27to395.08) | | 16.74 (5.53to35.63) | 68.57 (22.41to151.52) | -4.26 (-4.57to-3.95) | | 2.18 (0.67to4.54) | 12.92 (3.98to27.15) | | 1.51 (0.52to3.03) | 5.46 (1.85to11.19) | -3.85 (-4.12to-3.58) |
| Switzerland | 19.11 (6.83to37.30) | 170.88 (59.81to342.87) | | 14.31 (5.46to26.50) | 68.40 (25.40to133.20) | -3.54 (-3.68to-3.39) | | 1.48 (0.55to2.73) | 12.94 (4.76to23.98) | | 1.37 (0.54to2.48) | 5.76 (2.23to10.50) | -3.19 (-3.36to-3.03) |
| Syrian Arab Republic | 39.42 (16.56to78.95) | 802.50 (370.08to1489.49) | | 76.64 (32.80to145.94) | 732.19 (343.02to1296.49) | -0.73 (-0.91to-0.56) | | 1.99 (0.98to3.55) | 48.20 (24.56to82.41) | | 3.91 (1.83to6.82) | 48.17 (24.45to79.90) | -0.38 (-0.54to-0.21) |
| Taiwan (Province of China) | 10.83 (3.69to24.28) | 92.25 (32.83to200.05) | | 20.00 (7.45to41.79) | 49.85 (18.58to104.88) | -2.16 (-2.33to-1.99) | | 0.63 (0.22to1.33) | 6.65 (2.46to13.45) | | 1.41 (0.53to2.85) | 3.40 (1.27to6.96) | -2.40 (-2.60to-2.21) |
| Tajikistan | 3.22 (1.11to7.71) | 120.84 (41.58to289.09) | | 7.11 (2.39to18.72) | 236.75 (83.46to544.66) | 2.53 (2.29to2.77) | | 0.22 (0.08to0.50) | 8.24 (3.00to18.57) | | 0.42 (0.15to0.99) | 18.45 (6.48to40.90) | 2.99 (2.64to3.33) |
| Thailand | 21.15 (6.63to50.38) | 73.38 (23.62to169.44) | | 52.88 (17.81to115.44) | 53.65 (18.13to117.92) | -1.58 (-1.73to-1.42) | | 1.17 (0.38to2.67) | 4.99 (1.68to11.13) | | 3.33 (1.14to7.22) | 3.44 (1.18to7.45) | -1.81 (-1.97to-1.64) |
| Timor-Leste | 0.18 (0.06to0.43) | 88.32 (28.94to204.67) | | 0.79 (0.24to1.93) | 120.42 (39.08to285.04) | 1.20 (1.06to1.34) | | 0.01 (0.00to0.02) | 5.96 (2.06to13.28) | | 0.05 (0.01to0.11) | 8.64 (2.85to19.46) | 1.44 (1.31to1.57) |
| Togo | 1.09 (0.35to2.62) | 105.47 (35.96to245.07) | | 3.25 (1.03to7.99) | 108.02 (37.46to257.80) | 0.10 (0.03to0.18) | | 0.05 (0.02to0.13) | 6.70 (2.47to15.24) | | 0.15 (0.05to0.37) | 6.82 (2.52to15.72) | 0.10 (0.05to0.15) |
| Tokelau | 0.00 (0.00to0.01) | 234.95 (84.87to543.32) | | 0.00 (0.00to0.01) | 254.42 (95.01to536.39) | 0.30 (0.15to0.45) | | 0.00 (0.00to0.00) | 13.91 (5.50to29.33) | | 0.00 (0.00to0.00) | 15.42 (6.26to30.18) | 0.45 (0.30to0.60) |
| Tonga | 0.07 (0.03to0.17) | 150.14 (56.45to324.53) | | 0.13 (0.05to0.27) | 165.87 (62.75to342.91) | 0.17 (-0.07to0.41) | | 0.00 (0.00to0.01) | 9.01 (3.56to18.09) | | 0.01 (0.00to0.01) | 10.14 (4.18to19.51) | 0.30 (0.07to0.52) |
| Trinidad and Tobago | 3.16 (1.22to6.21) | 406.85 (162.64to778.31) | | 4.55 (1.89to8.70) | 254.03 (105.65to477.24) | -2.17 (-2.40to-1.95) | | 0.18 (0.07to0.32) | 26.24 (11.65to45.91) | | 0.27 (0.12to0.48) | 15.87 (7.17to27.80) | -2.21 (-2.43to-2.00) |
| Tunisia | 7.61 (2.37to17.84) | 185.46 (61.45to416.65) | | 21.49 (7.40to47.94) | 188.41 (66.93to417.54) | -0.16 (-0.31to-0.02) | | 0.43 (0.14to0.97) | 13.09 (4.71to27.75) | | 1.40 (0.51to2.94) | 13.46 (4.92to27.71) | -0.36 (-0.69to-0.03) |
| Turkey | 126.45 (46.95to259.34) | 375.01 (149.18to727.27) | | 192.87 (83.23to352.60) | 229.73 (100.65to414.18) | -1.92 (-2.12to-1.72) | | 6.45 (2.66to11.98) | 21.81 (9.24to39.87) | | 12.21 (5.80to20.53) | 15.36 (7.39to25.88) | -1.12 (-1.38to-0.86) |
| Turkmenistan | 3.74 (1.34to8.44) | 242.04 (90.97to520.78) | | 8.90 (3.26to20.25) | 277.51 (107.28to589.99) | -0.30 (-0.65to0.05) | | 0.23 (0.09to0.49) | 17.72 (6.87to36.29) | | 0.55 (0.22to1.14) | 19.62 (7.99to39.59) | -0.42 (-0.74to-0.10) |
| Tuvalu | 0.02 (0.01to0.04) | 271.45 (95.29to609.49) | | 0.03 (0.01to0.06) | 296.54 (111.58to649.30) | 0.25 (0.14to0.36) | | 0.00 (0.00to0.00) | 15.67 (5.96to32.63) | | 0.00 (0.00to0.00) | 17.94 (7.01to36.00) | 0.39 (0.26to0.51) |
| Uganda | 1.66 (0.54to4.68) | 30.85 (10.49to80.70) | | 3.89 (1.30to11.45) | 33.41 (11.67to89.30) | 0.07 (-0.17to0.31) | | 0.08 (0.03to0.22) | 1.95 (0.68to4.94) | | 0.20 (0.07to0.52) | 2.14 (0.74to5.29) | 0.19 (-0.02to0.41) |
| Ukraine | 129.04 (48.75to289.62) | 197.77 (75.86to438.53) | | 161.12 (58.72to353.52) | 205.07 (75.24to456.79) | -0.42 (-0.72to-0.13) | | 8.99 (3.46to19.25) | 15.36 (6.02to31.56) | | 11.86 (4.35to25.06) | 15.32 (5.74to32.19) | -0.58 (-0.87to-0.30) |
| United Arab Emirates | 4.15 (1.82to7.14) | 951.40 (529.83to1486.47) | | 30.45 (12.51to54.36) | 624.52 (321.65to984.11) | -1.76 (-2.22to-1.30) | | 0.13 (0.06to0.21) | 55.49 (32.71to83.15) | | 0.81 (0.35to1.41) | 34.49 (18.96to52.26) | -1.94 (-2.49to-1.38) |
| United Kingdom | 246.40 (86.49to496.17) | 267.75 (92.53to557.01) | | 134.14 (53.17to252.91) | 99.56 (37.86to195.91) | -4.02 (-4.25to-3.79) | | 16.72 (6.35to32.06) | 18.06 (6.82to34.53) | | 10.76 (4.59to18.80) | 7.21 (3.02to12.79) | -3.78 (-4.00to-3.55) |
| United Republic of Tanzania | 2.84 (0.92to8.11) | 31.41 (10.56to85.40) | | 7.90 (2.61to22.58) | 38.08 (13.02to100.51) | 0.78 (0.71to0.85) | | 0.14 (0.05to0.37) | 1.97 (0.68to5.01) | | 0.42 (0.15to1.11) | 2.46 (0.86to6.22) | 0.93 (0.83to1.03) |
| United States of America | 527.83 (160.37to1189.38) | 160.02 (47.78to367.22) | | 381.09 (110.61to912.72) | 66.42 (18.87to160.55) | -3.40 (-3.63to-3.16) | | 37.22 (11.57to78.48) | 10.96 (3.41to23.21) | | 27.50 (8.27to62.42) | 4.26 (1.27to9.79) | -3.69 (-4.02to-3.36) |
| United States Virgin Islands | 0.13 (0.04to0.29) | 181.12 (58.29to404.42) | | 0.27 (0.09to0.58) | 157.02 (57.53to331.25) | -0.41 (-0.62to-0.20) | | 0.01 (0.00to0.02) | 13.44 (4.43to28.56) | | 0.02 (0.01to0.04) | 12.08 (4.76to23.19) | -0.34 (-0.55to-0.13) |
| Uruguay | 4.36 (1.02to11.21) | 114.66 (26.83to295.12) | | 3.87 (1.05to9.44) | 68.20 (17.67to163.31) | -1.76 (-1.88to-1.64) | | 0.26 (0.07to0.67) | 7.09 (1.80to17.79) | | 0.27 (0.08to0.64) | 4.22 (1.19to9.90) | -1.83 (-1.95to-1.71) |
| Uzbekistan | 18.73 (6.93to40.96) | 185.90 (69.63to397.28) | | 47.78 (16.17to112.96) | 441.29 (169.09to948.37) | 3.11 (2.56to3.66) | | 1.30 (0.51to2.72) | 13.89 (5.52to28.43) | | 2.49 (0.89to5.66) | 35.21 (13.66to72.64) | 3.37 (2.81to3.94) |
| Vanuatu | 0.10 (0.03to0.27) | 185.34 (59.85to452.62) | | 0.33 (0.11to0.85) | 223.55 (74.87to544.91) | 0.45 (0.33to0.57) | | 0.00 (0.00to0.01) | 11.13 (3.74to25.67) | | 0.02 (0.01to0.04) | 13.71 (4.75to30.25) | 0.60 (0.51to0.68) |
| Venezuela (Bolivarian Republic of) | 4.80 (1.48to14.39) | 53.61 (16.35to155.14) | | 12.28 (3.63to36.08) | 45.00 (13.31to132.23) | -0.90 (-1.11to-0.70) | | 0.27 (0.08to0.75) | 3.48 (1.02to9.42) | | 0.82 (0.24to2.33) | 3.10 (0.90to8.77) | -0.64 (-0.86to-0.41) |
| Viet Nam | 24.83 (7.75to62.79) | 70.49 (22.41to174.72) | | 58.48 (18.93to145.86) | 74.78 (24.34to182.30) | 0.37 (0.32to0.43) | | 1.57 (0.50to3.71) | 4.94 (1.56to11.51) | | 3.85 (1.23to9.37) | 5.45 (1.75to13.08) | 0.49 (0.43to0.54) |
| Yemen | 28.73 (10.75to61.13) | 663.62 (286.98to1286.02) | | 69.49 (29.20to138.12) | 593.50 (269.21to1092.16) | -0.47 (-0.55to-0.38) | | 1.28 (0.55to2.49) | 38.66 (18.90to69.08) | | 3.37 (1.55to6.12) | 36.61 (18.44to62.97) | -0.25 (-0.32to-0.18) |
| Zambia | 1.65 (0.51to4.30) | 67.12 (21.85to166.67) | | 4.06 (1.32to10.28) | 72.89 (25.33to172.04) | 0.13 (0.00to0.26) | | 0.08 (0.02to0.19) | 3.91 (1.29to9.31) | | 0.19 (0.07to0.46) | 4.55 (1.64to10.36) | 0.42 (0.33to0.50) |
| Zimbabwe | 1.96 (0.64to5.11) | 59.10 (20.42to147.26) | | 4.49 (1.41to11.69) | 77.12 (25.52to194.51) | 1.17 (0.98to1.35) | | 0.10 (0.03to0.25) | 3.85 (1.33to9.06) | | 0.22 (0.07to0.56) | 4.86 (1.71to11.88) | 1.04 (0.86to1.22) |

**Supplemental table 4. DALYs and mortality of IHD attributable to LPA in 204 countries and territories in 1990 and 2019**

|  | **DALYs (95% UI)** | | | | | |  | **Mortality (95% UI)** | | | | | |
| --- | --- | --- | --- | --- | --- | --- | --- | --- | --- | --- | --- | --- | --- |
|  | **1990** | |  | **2019** | | **EAPC** |  | **1990** | |  | **2019** | | **EAPC** |
| **location** | **Number(no.×10^3^)** | **ASR per100,000** |  | **Number(no.×10^3^)** | **ASR per100,000** |  |  | **Number(no.×10^3^)** | **ASR per100,000** |  | **Number(no.×10^3^)** | **ASR per100,000** |  |
| Afghanistan | 46.13 (14.76to99.88) | 685.67 (241.41to1407.95) | | 67.14 (21.92to150.95) | 556.27 (208.74to1124.38) | -0.87 (-0.95to-0.78) | | 2.06 (0.76to4.07) | 36.68 (14.50to68.73) | | 2.82 (1.07to5.73) | 31.31 (12.88to57.08) | -0.67 (-0.73to-0.61) |
| Albania | 1.34 (0.43to3.34) | 76.44 (25.32to184.64) | | 3.18 (1.01to7.95) | 76.50 (24.57to191.29) | 0.37 (0.15to0.59) | | 0.09 (0.03to0.21) | 5.68 (2.03to12.95) | | 0.24 (0.08to0.56) | 5.96 (2.00to13.90) | 0.57 (0.33to0.80) |
| Algeria | 59.23 (21.81to121.27) | 635.40 (265.70to1171.16) | | 112.59 (46.60to209.20) | 399.31 (180.04to707.19) | -1.70 (-1.82to-1.59) | | 2.95 (1.19to5.40) | 44.89 (20.28to76.95) | | 6.71 (3.18to11.33) | 29.82 (14.71to48.00) | -1.41 (-1.52to-1.30) |
| American Samoa | 0.04 (0.01to0.09) | 187.42 (60.39to406.30) | | 0.10 (0.03to0.21) | 212.18 (69.15to449.35) | 0.30 (0.14to0.46) | | 0.00 (0.00to0.00) | 10.90 (3.84to21.58) | | 0.00 (0.00to0.01) | 12.18 (4.49to23.25) | 0.34 (0.22to0.47) |
| Andorra | 0.04 (0.01to0.09) | 80.99 (22.86to186.17) | | 0.08 (0.02to0.17) | 53.63 (15.68to117.43) | -1.63 (-1.78to-1.48) | | 0.00 (0.00to0.00) | 5.95 (1.74to12.40) | | 0.01 (0.00to0.01) | 4.02 (1.34to7.99) | -1.50 (-1.65to-1.34) |
| Angola | 2.96 (0.73to8.11) | 87.53 (24.51to229.88) | | 8.15 (2.17to21.34) | 88.72 (25.55to217.09) | -0.16 (-0.27to-0.05) | | 0.13 (0.04to0.33) | 5.03 (1.56to12.15) | | 0.38 (0.11to0.93) | 5.59 (1.71to13.02) | 0.18 (0.06to0.29) |
| Antigua and Barbuda | 0.08 (0.03to0.17) | 150.10 (44.19to320.83) | | 0.10 (0.03to0.21) | 113.94 (36.05to223.01) | -1.20 (-1.37to-1.03) | | 0.01 (0.00to0.01) | 9.95 (3.24to19.61) | | 0.01 (0.00to0.01) | 8.20 (2.84to14.91) | -0.88 (-1.04to-0.73) |
| Argentina | 7.42 (2.54to28.98) | 24.70 (8.26to95.02) | | 7.42 (2.31to25.68) | 13.61 (4.26to47.52) | -1.56 (-1.92to-1.19) | | 0.46 (0.14to1.62) | 1.68 (0.51to5.97) | | 0.55 (0.15to1.71) | 0.98 (0.27to3.06) | -1.24 (-1.68to-0.79) |
| Armenia | 3.53 (1.13to8.53) | 164.45 (54.11to386.30) | | 5.56 (1.89to12.68) | 138.01 (46.80to314.46) | -1.36 (-1.59to-1.13) | | 0.25 (0.08to0.57) | 13.13 (4.56to28.81) | | 0.43 (0.15to0.94) | 11.23 (4.00to24.48) | -1.41 (-1.68to-1.14) |
| Australia | 33.59 (9.22to77.15) | 176.93 (49.36to401.69) | | 28.81 (8.73to58.18) | 64.51 (19.71to136.30) | -4.00 (-4.18to-3.82) | | 2.26 (0.65to4.74) | 12.62 (3.71to25.89) | | 2.40 (0.82to4.51) | 4.88 (1.63to9.25) | -3.91 (-4.11to-3.70) |
| Austria | 17.71 (5.30to38.77) | 142.44 (42.44to316.68) | | 15.45 (5.08to31.71) | 73.33 (23.86to154.74) | -3.04 (-3.35to-2.74) | | 1.33 (0.42to2.74) | 10.84 (3.48to22.27) | | 1.47 (0.52to2.86) | 6.36 (2.19to12.52) | -2.45 (-2.77to-2.13) |
| Azerbaijan | 7.59 (2.44to18.22) | 171.12 (55.52to402.58) | | 16.60 (5.19to39.95) | 267.26 (88.35to601.13) | 1.35 (1.14to1.56) | | 0.50 (0.17to1.12) | 11.92 (4.11to26.52) | | 1.06 (0.34to2.38) | 22.12 (7.74to47.42) | 2.11 (1.87to2.34) |
| Bahamas | 0.25 (0.07to0.57) | 178.20 (52.75to383.64) | | 0.48 (0.14to1.06) | 129.77 (41.09to276.28) | -1.31 (-1.49to-1.12) | | 0.01 (0.00to0.03) | 11.04 (3.59to21.50) | | 0.03 (0.01to0.05) | 7.96 (2.78to15.22) | -1.41 (-1.64to-1.19) |
| Bahrain | 1.28 (0.44to2.57) | 822.13 (338.96to1446.74) | | 2.19 (0.80to4.30) | 288.95 (131.58to491.15) | -4.08 (-4.34to-3.81) | | 0.05 (0.02to0.10) | 50.96 (23.75to84.03) | | 0.09 (0.04to0.16) | 20.00 (9.75to32.22) | -3.44 (-3.71to-3.17) |
| Bangladesh | 33.64 (9.88to83.55) | 81.72 (25.28to194.72) | | 97.57 (29.27to239.19) | 81.75 (25.05to196.87) | 0.14 (-0.31to0.59) | | 1.75 (0.56to3.93) | 5.11 (1.70to10.94) | | 5.50 (1.80to12.44) | 5.29 (1.80to11.54) | 0.20 (-0.27to0.67) |
| Barbados | 0.52 (0.17to1.00) | 170.08 (56.74to338.83) | | 0.52 (0.19to0.97) | 106.26 (37.54to199.40) | -1.98 (-2.21to-1.75) | | 0.04 (0.01to0.07) | 12.03 (4.50to22.17) | | 0.04 (0.01to0.06) | 7.19 (2.83to12.63) | -2.08 (-2.33to-1.84) |
| Belarus | 22.68 (8.00to50.79) | 186.09 (65.93to413.18) | | 33.22 (11.52to73.75) | 198.78 (68.83to445.65) | -0.00 (-0.30to0.29) | | 1.77 (0.65to3.74) | 15.35 (5.71to32.17) | | 2.71 (0.97to5.68) | 16.06 (5.77to33.81) | -0.04 (-0.25to0.17) |
| Belgium | 23.64 (6.78to51.28) | 151.49 (43.12to337.21) | | 16.26 (5.19to32.29) | 62.78 (19.22to132.17) | -3.60 (-3.93to-3.27) | | 1.66 (0.53to3.32) | 10.69 (3.46to21.58) | | 1.37 (0.48to2.56) | 4.61 (1.58to8.78) | -3.36 (-3.61to-3.11) |
| Belize | 0.15 (0.04to0.31) | 163.43 (49.36to349.52) | | 0.31 (0.09to0.67) | 119.14 (37.25to246.41) | -1.60 (-2.09to-1.11) | | 0.01 (0.00to0.02) | 10.90 (3.57to21.58) | | 0.02 (0.01to0.03) | 7.51 (2.57to14.19) | -1.71 (-2.19to-1.24) |
| Benin | 1.31 (0.35to3.52) | 72.45 (19.62to190.58) | | 3.16 (0.86to8.39) | 74.87 (21.45to191.32) | 0.19 (0.14to0.24) | | 0.07 (0.02to0.18) | 4.57 (1.28to11.26) | | 0.17 (0.05to0.42) | 4.74 (1.43to11.27) | 0.23 (0.19to0.27) |
| Bermuda | 0.19 (0.06to0.39) | 318.01 (100.37to647.21) | | 0.15 (0.05to0.28) | 107.27 (36.28to207.57) | -3.96 (-4.30to-3.63) | | 0.01 (0.00to0.02) | 21.54 (7.50to40.16) | | 0.01 (0.00to0.02) | 7.85 (3.00to13.92) | -3.70 (-4.00to-3.40) |
| Bhutan | 0.27 (0.08to0.67) | 131.70 (46.76to299.70) | | 0.75 (0.25to1.64) | 149.79 (52.64to317.60) | 0.71 (0.54to0.87) | | 0.01 (0.00to0.03) | 8.22 (3.15to16.93) | | 0.05 (0.02to0.09) | 10.26 (3.86to20.11) | 0.98 (0.85to1.11) |
| Bolivia (Plurinational State of) | 1.91 (0.36to5.47) | 68.16 (12.95to193.34) | | 4.99 (1.06to12.76) | 64.65 (13.87to163.30) | -0.28 (-0.49to-0.08) | | 0.10 (0.02to0.29) | 4.46 (0.85to12.34) | | 0.30 (0.07to0.76) | 4.56 (0.99to11.34) | -0.03 (-0.25to0.19) |
| Bosnia and Herzegovina | 3.69 (1.01to9.17) | 109.31 (31.70to264.07) | | 4.80 (1.43to11.70) | 85.58 (25.84to207.70) | -1.43 (-1.63to-1.24) | | 0.21 (0.06to0.50) | 7.66 (2.43to17.62) | | 0.34 (0.11to0.79) | 6.53 (2.12to14.94) | -0.99 (-1.20to-0.77) |
| Botswana | 0.42 (0.11to1.13) | 82.62 (23.09to215.78) | | 1.27 (0.31to3.44) | 104.47 (28.48to268.31) | 0.54 (0.17to0.91) | | 0.02 (0.01to0.05) | 4.96 (1.47to11.95) | | 0.06 (0.02to0.15) | 6.30 (1.96to14.79) | 0.65 (0.37to0.92) |
| Brazil | 274.68 (91.19to520.81) | 323.16 (118.31to588.48) | | 414.95 (168.85to734.27) | 177.48 (75.02to310.13) | -1.95 (-2.03to-1.87) | | 12.61 (4.96to22.02) | 18.48 (7.88to31.26) | | 21.90 (11.04to34.92) | 9.79 (5.05to15.40) | -2.06 (-2.19to-1.94) |
| Brunei Darussalam | 0.14 (0.03to0.39) | 175.25 (40.23to416.78) | | 0.27 (0.06to0.73) | 118.27 (30.39to278.39) | -1.21 (-1.34to-1.08) | | 0.01 (0.00to0.02) | 11.54 (3.05to25.61) | | 0.01 (0.00to0.03) | 8.50 (2.39to18.45) | -0.92 (-1.07to-0.78) |
| Bulgaria | 18.09 (6.06to43.34) | 184.80 (64.12to435.44) | | 18.06 (6.19to41.66) | 123.59 (43.07to295.90) | -2.35 (-2.77to-1.93) | | 1.17 (0.40to2.67) | 14.87 (5.34to33.11) | | 1.42 (0.50to3.15) | 9.94 (3.57to21.77) | -2.37 (-2.80to-1.95) |
| Burkina Faso | 1.29 (0.37to3.85) | 34.66 (9.96to99.73) | | 3.61 (1.03to10.40) | 46.62 (13.56to132.74) | 1.10 (0.73to1.47) | | 0.06 (0.02to0.19) | 2.18 (0.62to5.79) | | 0.19 (0.05to0.51) | 2.97 (0.87to7.91) | 1.25 (0.91to1.59) |
| Burundi | 0.70 (0.20to2.43) | 32.70 (9.00to107.87) | | 1.10 (0.32to4.13) | 27.93 (8.08to94.36) | -0.70 (-0.76to-0.63) | | 0.03 (0.01to0.11) | 1.89 (0.50to5.77) | | 0.05 (0.01to0.17) | 1.70 (0.49to4.91) | -0.45 (-0.51to-0.39) |
| Cabo Verde | 0.15 (0.04to0.39) | 63.66 (17.58to163.01) | | 0.33 (0.10to0.83) | 80.81 (23.50to197.20) | 0.22 (-0.12to0.56) | | 0.01 (0.00to0.03) | 4.42 (1.28to11.12) | | 0.02 (0.01to0.05) | 5.68 (1.73to13.12) | 0.36 (-0.05to0.76) |
| Cambodia | 2.33 (0.57to6.62) | 61.24 (14.70to164.78) | | 5.69 (1.39to16.33) | 57.92 (14.87to157.48) | -0.23 (-0.29to-0.17) | | 0.11 (0.03to0.31) | 3.77 (0.93to9.89) | | 0.33 (0.08to0.87) | 4.02 (1.08to10.22) | 0.22 (0.17to0.27) |
| Cameroon | 2.46 (0.64to6.42) | 66.95 (18.53to169.80) | | 8.56 (2.30to22.85) | 83.98 (23.98to212.29) | 0.90 (0.66to1.13) | | 0.13 (0.04to0.33) | 4.39 (1.26to10.50) | | 0.43 (0.12to1.08) | 5.27 (1.56to12.38) | 0.76 (0.58to0.94) |
| Canada | 37.87 (9.35to91.63) | 118.63 (29.63to283.44) | | 39.08 (10.85to87.99) | 52.74 (14.03to122.74) | -3.27 (-3.48to-3.05) | | 2.69 (0.71to6.23) | 8.74 (2.38to19.98) | | 3.26 (0.96to6.89) | 4.06 (1.18to8.69) | -3.19 (-3.39to-2.98) |
| Central African Republic | 1.20 (0.31to3.16) | 116.63 (34.18to297.12) | | 2.19 (0.56to6.04) | 117.64 (34.68to301.41) | 0.14 (0.09to0.20) | | 0.05 (0.01to0.13) | 6.53 (2.07to16.00) | | 0.09 (0.03to0.24) | 6.79 (2.18to16.42) | 0.29 (0.22to0.37) |
| Chad | 1.32 (0.35to3.69) | 52.08 (14.02to142.36) | | 2.76 (0.72to7.70) | 57.50 (15.17to156.14) | 0.58 (0.51to0.65) | | 0.07 (0.02to0.20) | 3.35 (0.95to8.94) | | 0.14 (0.04to0.38) | 3.63 (1.00to9.53) | 0.46 (0.41to0.52) |
| Chile | 5.23 (1.22to14.15) | 60.46 (14.32to160.84) | | 7.26 (1.72to18.52) | 30.31 (7.19to77.38) | -2.26 (-2.37to-2.14) | | 0.34 (0.08to0.90) | 4.59 (1.11to11.86) | | 0.49 (0.12to1.18) | 2.09 (0.51to4.99) | -2.59 (-2.75to-2.43) |
| China | 438.94 (135.33to1075.62) | 71.81 (23.11to163.50) | | 1207.44 (355.78to2842.59) | 74.04 (22.58to169.21) | 0.27 (0.07to0.46) | | 24.94 (7.87to56.77) | 5.41 (1.84to11.51) | | 88.69 (27.44to197.69) | 6.28 (1.96to13.60) | 0.78 (0.54to1.02) |
| Colombia | 10.42 (2.39to31.10) | 66.25 (15.00to189.72) | | 22.02 (4.87to60.29) | 40.57 (8.97to111.21) | -1.87 (-2.05to-1.70) | | 0.59 (0.13to1.66) | 4.40 (0.95to11.88) | | 1.63 (0.36to4.28) | 2.86 (0.63to7.59) | -1.65 (-1.82to-1.48) |
| Comoros | 0.04 (0.01to0.17) | 20.81 (6.61to81.92) | | 0.09 (0.03to0.34) | 20.35 (6.03to71.66) | -0.26 (-0.35to-0.16) | | 0.00 (0.00to0.01) | 1.28 (0.39to4.41) | | 0.00 (0.00to0.02) | 1.29 (0.38to4.19) | -0.11 (-0.19to-0.04) |
| Congo | 1.36 (0.36to3.63) | 142.54 (42.53to365.06) | | 2.88 (0.77to7.34) | 127.45 (37.91to307.03) | -0.45 (-0.55to-0.35) | | 0.06 (0.02to0.15) | 8.13 (2.59to19.54) | | 0.13 (0.04to0.32) | 7.89 (2.54to17.72) | -0.14 (-0.21to-0.06) |
| Cook Islands | 0.02 (0.00to0.04) | 130.34 (37.59to309.36) | | 0.03 (0.01to0.07) | 123.08 (39.46to277.03) | -0.31 (-0.44to-0.19) | | 0.00 (0.00to0.00) | 7.80 (2.51to17.03) | | 0.00 (0.00to0.00) | 7.42 (2.65to14.63) | -0.28 (-0.41to-0.16) |
| Costa Rica | 0.56 (0.14to1.80) | 33.71 (8.17to107.57) | | 1.17 (0.28to3.55) | 22.90 (5.49to69.11) | -1.82 (-2.09to-1.55) | | 0.04 (0.01to0.11) | 2.37 (0.53to7.07) | | 0.09 (0.02to0.26) | 1.66 (0.37to4.85) | -1.79 (-2.09to-1.49) |
| Côte d'Ivoire | 2.90 (0.73to8.27) | 89.22 (23.89to235.29) | | 7.27 (1.85to19.50) | 81.48 (22.71to207.11) | -0.10 (-0.26to0.07) | | 0.13 (0.04to0.35) | 5.59 (1.59to13.78) | | 0.35 (0.10to0.89) | 5.13 (1.46to12.15) | -0.14 (-0.25to-0.04) |
| Croatia | 7.51 (2.55to17.74) | 133.92 (46.69to311.40) | | 7.28 (2.40to16.76) | 77.73 (25.99to179.01) | -1.64 (-1.79to-1.49) | | 0.53 (0.18to1.19) | 10.75 (3.82to23.81) | | 0.63 (0.22to1.39) | 6.82 (2.45to14.99) | -1.20 (-1.39to-1.00) |
| Cuba | 24.45 (7.29to50.87) | 244.00 (74.15to504.89) | | 29.89 (10.17to59.55) | 151.94 (49.91to306.91) | -1.98 (-2.25to-1.71) | | 1.60 (0.52to3.11) | 17.05 (5.64to32.64) | | 2.16 (0.80to3.97) | 10.37 (3.78to19.16) | -2.11 (-2.38to-1.83) |
| Cyprus | 1.15 (0.28to2.86) | 177.96 (44.90to427.43) | | 1.42 (0.35to3.54) | 80.67 (21.66to197.15) | -3.27 (-3.45to-3.09) | | 0.07 (0.02to0.17) | 13.87 (3.74to31.48) | | 0.10 (0.03to0.22) | 6.45 (1.94to13.75) | -3.17 (-3.34to-3.00) |
| Czechia | 24.56 (8.33to57.99) | 185.00 (62.66to429.33) | | 22.27 (8.08to49.92) | 101.86 (36.68to228.72) | -2.13 (-2.20to-2.06) | | 1.66 (0.59to3.69) | 13.53 (4.92to29.32) | | 1.92 (0.71to4.10) | 8.65 (3.22to18.48) | -1.56 (-1.66to-1.46) |
| Democratic People's Republic of Korea | 7.71 (2.17to20.95) | 66.34 (19.08to160.00) | | 21.66 (6.18to54.87) | 76.47 (22.26to190.26) | 0.70 (0.46to0.93) | | 0.45 (0.13to1.08) | 4.93 (1.47to11.09) | | 1.46 (0.44to3.41) | 5.84 (1.77to13.11) | 0.82 (0.57to1.08) |
| Democratic Republic of the Congo | 13.00 (3.50to35.07) | 96.72 (29.38to245.97) | | 28.46 (7.70to73.32) | 93.94 (28.01to227.92) | -0.18 (-0.26to-0.11) | | 0.57 (0.17to1.45) | 5.76 (1.84to13.63) | | 1.39 (0.42to3.34) | 5.87 (1.86to13.52) | -0.01 (-0.08to0.05) |
| Denmark | 14.93 (3.81to34.75) | 175.89 (44.23to415.95) | | 6.11 (1.74to13.29) | 49.18 (13.70to110.78) | -5.25 (-5.70to-4.81) | | 1.12 (0.31to2.38) | 12.84 (3.56to27.58) | | 0.53 (0.16to1.08) | 3.98 (1.22to8.19) | -4.85 (-5.22to-4.49) |
| Djibouti | 0.03 (0.01to0.10) | 23.85 (6.90to73.43) | | 0.15 (0.04to0.50) | 30.01 (8.26to91.76) | 0.88 (0.80to0.97) | | 0.00 (0.00to0.00) | 1.51 (0.44to4.54) | | 0.01 (0.00to0.02) | 1.91 (0.53to5.44) | 0.90 (0.82to0.98) |
| Dominica | 0.12 (0.04to0.25) | 160.24 (46.97to342.54) | | 0.10 (0.03to0.21) | 111.06 (34.65to231.51) | -1.45 (-1.64to-1.26) | | 0.01 (0.00to0.02) | 11.33 (3.66to22.06) | | 0.01 (0.00to0.01) | 7.81 (2.63to14.44) | -1.45 (-1.61to-1.29) |
| Dominican Republic | 7.47 (2.27to15.52) | 212.07 (67.88to419.21) | | 29.08 (8.94to59.41) | 316.99 (102.18to633.09) | 2.26 (1.96to2.57) | | 0.38 (0.13to0.71) | 13.20 (4.91to23.71) | | 1.54 (0.57to2.87) | 18.12 (6.89to33.15) | 2.12 (1.77to2.47) |
| Ecuador | 2.96 (0.61to8.04) | 60.33 (12.62to160.09) | | 8.35 (1.84to21.41) | 59.52 (13.66to147.51) | -0.04 (-0.26to0.19) | | 0.17 (0.04to0.44) | 4.17 (0.98to10.31) | | 0.51 (0.12to1.22) | 4.17 (1.01to9.73) | 0.13 (-0.12to0.38) |
| Egypt | 185.32 (66.10to370.66) | 708.41 (280.15to1363.43) | | 384.41 (134.30to793.96) | 674.30 (260.38to1290.90) | -0.13 (-0.22to-0.05) | | 8.76 (3.55to16.28) | 42.65 (18.72to74.29) | | 17.92 (7.07to33.90) | 40.15 (17.44to71.16) | -0.13 (-0.25to-0.02) |
| El Salvador | 0.91 (0.24to3.01) | 31.68 (8.07to102.22) | | 1.99 (0.48to6.20) | 31.48 (7.53to97.14) | -0.00 (-0.13to0.13) | | 0.06 (0.01to0.17) | 2.04 (0.47to6.10) | | 0.16 (0.03to0.44) | 2.30 (0.52to6.46) | 0.47 (0.36to0.58) |
| Equatorial Guinea | 0.18 (0.05to0.50) | 104.20 (29.54to268.48) | | 0.38 (0.11to0.90) | 94.19 (29.94to210.66) | -0.61 (-0.82to-0.39) | | 0.01 (0.00to0.02) | 5.88 (1.82to14.93) | | 0.02 (0.01to0.04) | 6.30 (2.07to13.45) | 0.03 (-0.12to0.18) |
| Eritrea | 0.22 (0.06to0.81) | 25.66 (7.43to85.15) | | 0.68 (0.19to2.61) | 30.71 (8.98to97.96) | 0.79 (0.72to0.86) | | 0.01 (0.00to0.03) | 1.45 (0.42to4.40) | | 0.03 (0.01to0.10) | 1.90 (0.55to5.49) | 1.06 (0.99to1.13) |
| Estonia | 3.49 (1.25to7.99) | 180.11 (66.19to405.07) | | 2.73 (0.97to5.94) | 85.49 (30.41to192.50) | -3.12 (-3.38to-2.86) | | 0.27 (0.10to0.59) | 15.11 (5.54to32.50) | | 0.26 (0.09to0.54) | 7.74 (2.88to16.45) | -2.79 (-3.02to-2.56) |
| Eswatini | 0.22 (0.05to0.57) | 83.44 (23.08to212.11) | | 0.57 (0.14to1.46) | 107.22 (30.81to257.26) | 0.99 (0.56to1.42) | | 0.01 (0.00to0.03) | 4.87 (1.43to11.77) | | 0.03 (0.01to0.06) | 6.19 (1.91to14.72) | 0.95 (0.60to1.31) |
| Ethiopia | 5.64 (1.52to20.59) | 29.47 (8.06to98.11) | | 7.59 (2.03to26.38) | 20.46 (5.47to66.69) | -1.55 (-1.63to-1.46) | | 0.22 (0.06to0.73) | 1.50 (0.41to4.61) | | 0.38 (0.10to1.20) | 1.26 (0.34to3.65) | -0.81 (-0.88to-0.75) |
| Fiji | 0.83 (0.23to2.17) | 254.89 (73.83to613.71) | | 1.71 (0.47to4.26) | 260.57 (80.56to602.27) | -0.20 (-0.38to-0.02) | | 0.04 (0.01to0.09) | 14.83 (4.76to32.80) | | 0.08 (0.02to0.18) | 15.73 (5.43to31.72) | -0.13 (-0.33to0.07) |
| Finland | 14.03 (3.41to33.61) | 195.88 (47.52to469.84) | | 12.58 (3.59to27.22) | 89.73 (25.47to199.52) | -3.04 (-3.30to-2.77) | | 0.94 (0.26to2.09) | 13.42 (3.71to29.52) | | 1.15 (0.36to2.29) | 7.36 (2.29to15.11) | -2.31 (-2.52to-2.10) |
| France | 94.54 (30.67to190.22) | 109.38 (34.53to226.59) | | 78.98 (27.59to150.88) | 49.84 (16.03to101.44) | -3.08 (-3.32to-2.84) | | 6.86 (2.45to12.86) | 7.70 (2.77to14.48) | | 7.10 (2.74to12.71) | 3.66 (1.36to6.78) | -2.88 (-3.07to-2.68) |
| Gabon | 0.44 (0.13to1.13) | 90.06 (26.51to219.15) | | 0.83 (0.23to2.06) | 94.26 (29.34to219.17) | 0.14 (-0.02to0.31) | | 0.02 (0.01to0.06) | 5.72 (1.85to13.12) | | 0.05 (0.01to0.11) | 6.34 (2.17to14.04) | 0.34 (0.20to0.47) |
| Gambia | 0.21 (0.06to0.57) | 71.77 (19.73to186.60) | | 0.76 (0.20to2.01) | 89.97 (24.84to234.85) | 0.83 (0.70to0.95) | | 0.01 (0.00to0.03) | 4.71 (1.31to11.70) | | 0.04 (0.01to0.11) | 5.84 (1.71to14.50) | 0.85 (0.74to0.95) |
| Georgia | 11.40 (3.58to27.76) | 218.35 (72.34to519.32) | | 7.99 (2.69to18.52) | 121.59 (40.51to284.87) | -2.53 (-2.84to-2.21) | | 0.76 (0.25to1.72) | 17.07 (5.86to37.67) | | 0.63 (0.22to1.38) | 8.93 (3.11to19.81) | -2.80 (-3.15to-2.44) |
| Germany | 187.57 (45.96to456.68) | 141.34 (34.88to346.57) | | 134.57 (35.01to307.51) | 60.54 (15.22to142.49) | -3.61 (-4.07to-3.14) | | 14.15 (3.67to32.38) | 10.76 (2.85to24.62) | | 12.01 (3.30to26.28) | 4.99 (1.34to11.00) | -3.16 (-3.48to-2.83) |
| Ghana | 3.54 (0.95to9.70) | 68.08 (19.62to176.41) | | 11.10 (3.09to29.54) | 82.22 (23.70to207.52) | 0.70 (0.62to0.77) | | 0.17 (0.05to0.45) | 4.39 (1.31to10.75) | | 0.59 (0.17to1.44) | 5.43 (1.65to12.85) | 0.82 (0.74to0.90) |
| Greece | 12.22 (2.75to32.71) | 82.45 (18.80to219.04) | | 16.94 (4.29to41.19) | 60.40 (14.41to153.92) | -1.71 (-2.19to-1.23) | | 0.86 (0.20to2.17) | 6.20 (1.47to15.22) | | 1.50 (0.38to3.38) | 4.59 (1.18to10.78) | -1.52 (-1.92to-1.13) |
| Greenland | 0.04 (0.01to0.10) | 138.61 (33.71to343.74) | | 0.04 (0.01to0.11) | 71.14 (16.56to177.44) | -2.96 (-3.23to-2.69) | | 0.00 (0.00to0.00) | 9.30 (2.33to21.79) | | 0.00 (0.00to0.01) | 4.93 (1.27to11.38) | -2.95 (-3.26to-2.64) |
| Grenada | 0.16 (0.05to0.34) | 205.75 (58.53to451.24) | | 0.13 (0.04to0.30) | 132.38 (40.68to278.26) | -1.59 (-1.86to-1.32) | | 0.01 (0.00to0.02) | 13.54 (4.19to27.25) | | 0.01 (0.00to0.02) | 8.61 (2.86to16.77) | -1.67 (-1.92to-1.42) |
| Guam | 0.09 (0.03to0.24) | 163.69 (50.02to375.78) | | 0.29 (0.09to0.66) | 155.89 (47.61to357.31) | 0.19 (-0.19to0.57) | | 0.00 (0.00to0.01) | 11.52 (3.78to24.87) | | 0.02 (0.01to0.04) | 9.53 (3.17to20.21) | -0.48 (-0.93to-0.03) |
| Guatemala | 0.67 (0.26to3.46) | 19.88 (7.54to95.55) | | 1.33 (0.46to6.59) | 13.27 (4.44to60.00) | -1.73 (-2.08to-1.38) | | 0.03 (0.01to0.14) | 1.20 (0.43to5.11) | | 0.08 (0.03to0.33) | 1.00 (0.31to3.85) | -1.02 (-1.27to-0.76) |
| Guinea | 1.51 (0.40to4.04) | 51.25 (13.77to133.51) | | 3.03 (0.80to8.28) | 61.59 (16.37to167.16) | 1.03 (0.90to1.16) | | 0.08 (0.02to0.22) | 3.33 (0.92to8.33) | | 0.17 (0.05to0.44) | 3.94 (1.10to9.87) | 0.96 (0.83to1.10) |
| Guinea-Bissau | 0.35 (0.09to0.96) | 97.96 (25.81to257.50) | | 0.63 (0.16to1.80) | 101.63 (26.87to260.87) | 0.32 (0.22to0.42) | | 0.02 (0.00to0.04) | 5.67 (1.63to14.43) | | 0.03 (0.01to0.07) | 6.10 (1.80to14.66) | 0.44 (0.35to0.52) |
| Guyana | 1.09 (0.28to2.66) | 316.52 (88.24to723.49) | | 1.49 (0.41to3.46) | 258.96 (76.98to567.01) | -0.67 (-0.82to-0.53) | | 0.06 (0.02to0.12) | 19.12 (5.79to38.69) | | 0.08 (0.02to0.16) | 15.70 (5.16to30.73) | -0.76 (-0.97to-0.55) |
| Haiti | 7.51 (1.96to18.89) | 265.21 (74.31to633.14) | | 13.41 (3.66to34.06) | 222.88 (63.13to520.64) | -0.44 (-0.57to-0.31) | | 0.36 (0.10to0.83) | 15.94 (4.91to33.65) | | 0.68 (0.20to1.57) | 14.11 (4.45to29.73) | -0.30 (-0.40to-0.20) |
| Honduras | 0.54 (0.15to1.87) | 27.65 (7.51to92.65) | | 2.04 (0.54to6.62) | 39.81 (10.19to124.15) | 1.29 (1.08to1.51) | | 0.03 (0.01to0.10) | 1.74 (0.44to5.53) | | 0.13 (0.03to0.39) | 2.96 (0.67to8.95) | 1.76 (1.44to2.08) |
| Hungary | 17.07 (5.79to41.43) | 126.33 (44.31to304.24) | | 17.51 (6.24to40.51) | 85.39 (30.14to200.83) | -1.51 (-1.66to-1.36) | | 1.12 (0.40to2.57) | 9.24 (3.33to20.94) | | 1.48 (0.53to3.25) | 7.01 (2.53to15.49) | -1.14 (-1.36to-0.92) |
| Iceland | 0.52 (0.14to1.13) | 175.99 (47.31to393.42) | | 0.43 (0.13to0.90) | 70.74 (20.40to152.88) | -3.49 (-3.59to-3.38) | | 0.04 (0.01to0.08) | 12.29 (3.69to24.90) | | 0.04 (0.01to0.07) | 5.44 (1.76to10.75) | -3.12 (-3.22to-3.03) |
| India | 513.15 (147.73to1255.10) | 136.09 (44.45to310.06) | | 1043.01 (347.29to2600.05) | 106.95 (37.58to249.67) | -0.23 (-0.44to-0.02) | | 22.53 (7.14to52.17) | 8.30 (2.86to17.83) | | 61.29 (22.16to134.01) | 7.45 (2.83to15.74) | -0.07 (-0.27to0.13) |
| Indonesia | 78.24 (18.13to214.80) | 88.82 (22.15to226.29) | | 217.97 (50.45to561.34) | 114.45 (28.46to271.54) | 0.87 (0.74to0.99) | | 3.56 (0.91to9.03) | 5.22 (1.42to12.73) | | 10.56 (2.73to24.46) | 7.09 (1.94to15.83) | 1.06 (0.92to1.20) |
| Iran (Islamic Republic of) | 88.51 (28.21to194.53) | 402.01 (145.50to815.49) | | 152.57 (55.07to305.00) | 227.49 (84.01to433.36) | -2.55 (-2.81to-2.29) | | 4.09 (1.48to8.26) | 25.08 (10.03to46.18) | | 9.35 (3.65to17.20) | 15.63 (6.30to28.06) | -2.17 (-2.42to-1.92) |
| Iraq | 43.55 (17.09to82.31) | 596.59 (249.61to1086.40) | | 101.57 (39.84to192.35) | 500.79 (218.52to901.80) | -0.85 (-0.92to-0.78) | | 2.25 (1.02to3.90) | 34.96 (16.77to58.59) | | 5.14 (2.35to9.14) | 31.11 (15.35to51.80) | -0.60 (-0.68to-0.52) |
| Ireland | 12.34 (4.20to25.14) | 309.69 (105.34to618.83) | | 7.75 (2.75to14.66) | 101.59 (35.43to193.70) | -4.60 (-4.87to-4.32) | | 0.77 (0.29to1.44) | 20.48 (7.72to37.68) | | 0.60 (0.24to1.05) | 7.66 (3.03to13.50) | -4.07 (-4.33to-3.81) |
| Israel | 8.53 (2.18to19.11) | 181.51 (47.65to406.25) | | 6.39 (2.03to13.21) | 51.62 (16.25to107.84) | -5.09 (-5.38to-4.81) | | 0.55 (0.16to1.15) | 12.75 (3.79to26.06) | | 0.54 (0.18to1.02) | 4.03 (1.38to7.71) | -4.70 (-4.95to-4.45) |
| Italy | 120.72 (37.67to253.86) | 138.20 (43.18to293.86) | | 98.98 (32.72to194.73) | 57.69 (17.99to121.21) | -3.51 (-3.75to-3.26) | | 8.35 (2.81to16.49) | 9.97 (3.37to19.44) | | 9.15 (3.22to17.26) | 4.63 (1.57to8.91) | -3.01 (-3.19to-2.83) |
| Jamaica | 1.99 (0.67to3.96) | 109.72 (36.58to218.06) | | 3.13 (1.09to6.18) | 100.56 (34.51to202.02) | -0.20 (-0.53to0.14) | | 0.14 (0.05to0.26) | 7.72 (2.81to14.16) | | 0.22 (0.08to0.39) | 6.35 (2.38to11.51) | -0.58 (-0.91to-0.24) |
| Japan | 97.95 (23.62to237.31) | 62.23 (15.56to148.82) | | 105.08 (27.03to244.18) | 25.31 (6.12to62.65) | -3.08 (-3.24to-2.91) | | 6.69 (1.65to15.44) | 4.66 (1.22to10.51) | | 9.58 (2.65to21.21) | 1.79 (0.47to4.02) | -3.32 (-3.51to-3.13) |
| Jordan | 3.24 (1.06to7.20) | 290.88 (105.90to596.71) | | 10.21 (3.54to21.83) | 183.84 (71.56to363.32) | -2.20 (-2.48to-1.92) | | 0.16 (0.06to0.33) | 18.64 (7.41to35.15) | | 0.51 (0.20to0.98) | 12.00 (5.10to21.25) | -2.07 (-2.34to-1.80) |
| Kazakhstan | 22.46 (7.42to51.42) | 207.29 (71.80to461.76) | | 25.96 (8.64to57.62) | 191.16 (67.12to408.67) | -1.14 (-1.88to-0.40) | | 1.48 (0.52to3.14) | 15.39 (5.52to32.00) | | 1.79 (0.63to3.74) | 15.56 (5.71to31.68) | -0.66 (-1.29to-0.03) |
| Kenya | 0.95 (0.29to3.45) | 13.71 (4.17to46.84) | | 3.36 (1.01to11.61) | 17.80 (5.34to59.95) | 1.19 (1.00to1.38) | | 0.05 (0.01to0.16) | 0.89 (0.27to2.80) | | 0.16 (0.05to0.52) | 1.11 (0.34to3.37) | 0.98 (0.84to1.12) |
| Kiribati | 0.10 (0.02to0.26) | 261.45 (78.80to649.45) | | 0.18 (0.05to0.45) | 269.14 (81.17to633.44) | -0.07 (-0.22to0.08) | | 0.00 (0.00to0.01) | 12.78 (4.18to29.06) | | 0.01 (0.00to0.02) | 14.43 (4.95to31.58) | 0.29 (0.17to0.42) |
| Kuwait | 2.84 (1.06to5.51) | 446.12 (199.95to770.58) | | 7.74 (2.88to14.65) | 265.08 (116.52to461.97) | -1.60 (-1.94to-1.26) | | 0.11 (0.05to0.20) | 24.83 (12.56to39.57) | | 0.32 (0.14to0.55) | 14.47 (7.36to23.41) | -1.71 (-2.00to-1.41) |
| Kyrgyzstan | 3.40 (1.12to8.19) | 118.93 (39.31to283.66) | | 6.36 (2.10to14.83) | 175.81 (59.33to403.06) | 1.64 (1.14to2.14) | | 0.24 (0.08to0.53) | 8.54 (2.96to19.17) | | 0.48 (0.16to1.05) | 14.51 (4.96to31.67) | 2.31 (1.73to2.90) |
| Lao People's Democratic Republic | 1.37 (0.34to3.93) | 77.98 (19.53to211.07) | | 2.78 (0.65to7.78) | 77.93 (18.66to209.58) | -0.36 (-0.56to-0.16) | | 0.07 (0.02to0.18) | 4.76 (1.21to12.71) | | 0.15 (0.04to0.40) | 5.35 (1.35to13.59) | 0.11 (-0.04to0.26) |
| Latvia | 9.08 (3.32to19.22) | 257.94 (95.35to546.10) | | 8.34 (3.30to16.55) | 179.40 (70.77to367.70) | -1.51 (-1.71to-1.31) | | 0.68 (0.26to1.37) | 20.45 (7.79to40.65) | | 0.72 (0.29to1.36) | 14.89 (6.05to28.35) | -1.25 (-1.40to-1.11) |
| Lebanon | 13.08 (4.68to25.90) | 626.15 (250.95to1186.78) | | 24.30 (9.82to44.11) | 468.40 (190.01to849.75) | -0.78 (-1.05to-0.50) | | 0.66 (0.27to1.19) | 37.36 (16.37to65.62) | | 1.45 (0.65to2.45) | 28.80 (12.86to48.64) | -0.67 (-0.87to-0.47) |
| Lesotho | 0.37 (0.09to1.05) | 42.04 (10.64to112.96) | | 0.83 (0.19to2.31) | 72.46 (18.11to192.91) | 2.40 (2.13to2.68) | | 0.02 (0.00to0.05) | 2.52 (0.68to6.49) | | 0.04 (0.01to0.10) | 4.24 (1.20to10.75) | 2.32 (2.07to2.57) |
| Liberia | 0.90 (0.25to2.35) | 91.27 (25.47to229.81) | | 1.53 (0.40to3.88) | 87.51 (24.43to217.15) | 0.09 (-0.10to0.28) | | 0.05 (0.01to0.12) | 5.81 (1.71to14.17) | | 0.08 (0.02to0.19) | 5.60 (1.67to13.05) | 0.13 (-0.05to0.31) |
| Libya | 6.38 (2.37to12.58) | 357.23 (141.65to675.09) | | 17.13 (6.51to33.57) | 344.24 (139.95to633.49) | -0.08 (-0.24to0.08) | | 0.33 (0.14to0.60) | 20.95 (9.44to36.71) | | 0.86 (0.38to1.52) | 20.00 (8.97to34.00) | -0.01 (-0.18to0.16) |
| Lithuania | 13.21 (4.94to27.50) | 293.62 (109.80to613.67) | | 14.00 (5.59to27.55) | 206.88 (81.77to421.74) | -1.22 (-1.42to-1.02) | | 1.04 (0.40to2.05) | 24.06 (9.19to47.23) | | 1.25 (0.52to2.32) | 17.87 (7.33to33.51) | -1.00 (-1.20to-0.79) |
| Luxembourg | 0.77 (0.19to1.86) | 143.16 (36.08to344.17) | | 0.56 (0.16to1.20) | 50.41 (13.86to112.49) | -4.00 (-4.26to-3.74) | | 0.05 (0.01to0.12) | 10.28 (2.86to22.71) | | 0.05 (0.01to0.09) | 3.90 (1.14to8.05) | -3.63 (-3.84to-3.43) |
| Madagascar | 1.40 (0.41to4.93) | 30.58 (8.98to100.62) | | 3.02 (0.85to11.76) | 32.67 (9.07to108.61) | 0.17 (0.04to0.29) | | 0.07 (0.02to0.22) | 1.91 (0.55to5.60) | | 0.14 (0.04to0.45) | 2.05 (0.57to6.03) | 0.20 (0.10to0.31) |
| Malawi | 0.97 (0.28to3.34) | 28.49 (8.16to87.90) | | 1.76 (0.51to5.62) | 27.62 (8.01to82.42) | 0.05 (-0.06to0.16) | | 0.04 (0.01to0.13) | 1.65 (0.47to4.62) | | 0.09 (0.03to0.25) | 1.70 (0.50to4.64) | 0.22 (0.12to0.32) |
| Malaysia | 15.29 (4.05to35.19) | 182.28 (51.95to404.30) | | 46.93 (13.28to108.64) | 186.57 (53.92to412.70) | -0.28 (-0.51to-0.04) | | 0.77 (0.23to1.67) | 10.51 (3.17to22.25) | | 2.32 (0.71to4.85) | 10.76 (3.43to21.79) | -0.57 (-0.94to-0.20) |
| Maldives | 0.20 (0.04to0.50) | 254.20 (65.40to592.71) | | 0.33 (0.09to0.81) | 119.79 (35.54to278.55) | -3.30 (-3.51to-3.08) | | 0.01 (0.00to0.02) | 14.99 (4.21to32.98) | | 0.02 (0.01to0.04) | 8.07 (2.52to17.32) | -2.75 (-2.93to-2.56) |
| Mali | 1.78 (0.48to4.94) | 53.94 (14.94to147.35) | | 3.69 (1.00to10.16) | 52.67 (14.61to137.62) | -0.07 (-0.14to-0.01) | | 0.10 (0.03to0.26) | 3.67 (1.02to9.48) | | 0.21 (0.06to0.55) | 3.63 (1.00to9.29) | 0.02 (-0.04to0.08) |
| Malta | 1.50 (0.54to2.74) | 362.73 (135.80to657.28) | | 1.52 (0.62to2.58) | 158.71 (62.25to278.22) | -2.90 (-2.99to-2.82) | | 0.09 (0.04to0.16) | 23.91 (10.04to40.72) | | 0.12 (0.05to0.19) | 11.24 (4.93to18.45) | -2.61 (-2.70to-2.52) |
| Marshall Islands | 0.04 (0.01to0.11) | 287.33 (82.18to680.66) | | 0.11 (0.03to0.29) | 338.37 (99.56to778.81) | 0.48 (0.26to0.70) | | 0.00 (0.00to0.00) | 16.39 (5.40to35.35) | | 0.00 (0.00to0.01) | 18.92 (6.30to38.90) | 0.49 (0.34to0.65) |
| Mauritania | 2.32 (0.80to4.54) | 247.41 (87.82to472.87) | | 3.36 (1.28to6.51) | 175.24 (69.26to332.12) | -1.30 (-1.43to-1.17) | | 0.11 (0.04to0.21) | 14.17 (5.41to26.27) | | 0.18 (0.07to0.33) | 10.87 (4.59to19.50) | -0.99 (-1.12to-0.86) |
| Mauritius | 1.68 (0.40to4.20) | 243.34 (62.54to588.16) | | 1.74 (0.45to4.17) | 105.55 (28.74to246.69) | -3.77 (-4.18to-3.35) | | 0.08 (0.02to0.19) | 14.01 (4.13to32.17) | | 0.10 (0.03to0.22) | 6.70 (1.97to14.26) | -3.35 (-3.72to-2.99) |
| Mexico | 27.73 (5.86to76.66) | 71.18 (15.73to189.76) | | 77.33 (17.46to199.30) | 70.15 (16.23to176.02) | -0.26 (-0.53to0.01) | | 1.55 (0.36to3.99) | 4.80 (1.14to11.99) | | 5.05 (1.27to12.05) | 4.96 (1.28to11.69) | -0.33 (-0.67to0.01) |
| Micronesia (Federated States of) | 0.14 (0.04to0.35) | 314.89 (95.72to755.98) | | 0.24 (0.06to0.60) | 370.03 (113.16to855.03) | 0.44 (0.24to0.63) | | 0.01 (0.00to0.01) | 17.13 (5.66to37.79) | | 0.01 (0.00to0.02) | 20.95 (7.48to43.23) | 0.61 (0.47to0.76) |
| Monaco | 0.11 (0.03to0.24) | 147.02 (42.60to323.40) | | 0.09 (0.03to0.17) | 81.93 (26.59to167.80) | -2.16 (-2.37to-1.94) | | 0.01 (0.00to0.02) | 10.40 (3.38to20.78) | | 0.01 (0.00to0.01) | 6.15 (2.13to11.75) | -1.92 (-2.10to-1.75) |
| Mongolia | 1.74 (0.53to4.42) | 203.06 (66.01to492.06) | | 2.68 (0.84to7.30) | 169.04 (57.56to392.06) | -1.38 (-1.78to-0.97) | | 0.11 (0.04to0.25) | 14.98 (5.04to33.86) | | 0.16 (0.05to0.37) | 13.58 (4.75to29.70) | -0.92 (-1.30to-0.53) |
| Montenegro | 0.52 (0.16to1.27) | 89.53 (28.79to216.84) | | 0.89 (0.29to2.10) | 95.39 (31.53to223.01) | 0.45 (0.33to0.57) | | 0.03 (0.01to0.08) | 6.36 (2.22to14.20) | | 0.06 (0.02to0.14) | 7.10 (2.35to15.68) | 0.70 (0.54to0.86) |
| Morocco | 64.34 (23.56to130.58) | 519.40 (205.57to982.85) | | 141.52 (55.42to272.32) | 504.14 (212.03to924.54) | -0.24 (-0.41to-0.06) | | 3.18 (1.29to5.72) | 30.99 (13.34to53.17) | | 7.59 (3.40to13.46) | 31.87 (15.13to53.90) | -0.04 (-0.29to0.20) |
| Mozambique | 1.01 (0.30to3.49) | 19.78 (5.98to62.94) | | 2.50 (0.71to8.97) | 26.20 (7.57to86.61) | 1.50 (1.29to1.72) | | 0.05 (0.01to0.15) | 1.21 (0.35to3.49) | | 0.12 (0.03to0.38) | 1.59 (0.47to4.62) | 1.39 (1.21to1.56) |
| Myanmar | 16.11 (3.65to45.91) | 78.71 (18.53to216.42) | | 23.65 (5.69to63.49) | 59.04 (14.86to155.24) | -1.17 (-1.25to-1.09) | | 0.77 (0.18to2.08) | 4.71 (1.14to12.13) | | 1.37 (0.36to3.46) | 3.98 (1.09to9.75) | -0.72 (-0.77to-0.66) |
| Namibia | 0.87 (0.24to2.03) | 128.77 (36.68to292.98) | | 1.76 (0.51to3.99) | 132.98 (40.12to293.53) | -0.13 (-0.44to0.17) | | 0.04 (0.01to0.09) | 7.24 (2.20to16.04) | | 0.09 (0.03to0.19) | 7.68 (2.52to16.08) | -0.02 (-0.26to0.23) |
| Nauru | 0.01 (0.00to0.03) | 305.37 (87.69to747.38) | | 0.01 (0.00to0.04) | 371.94 (115.21to898.11) | 0.51 (0.22to0.79) | | 0.00 (0.00to0.00) | 17.22 (5.64to37.99) | | 0.00 (0.00to0.00) | 20.84 (7.25to44.87) | 0.55 (0.32to0.78) |
| Nepal | 5.97 (1.85to15.49) | 74.61 (24.44to179.73) | | 17.36 (5.11to43.29) | 90.22 (28.32to217.01) | 0.68 (0.54to0.83) | | 0.28 (0.09to0.67) | 4.50 (1.51to10.06) | | 0.98 (0.31to2.29) | 6.05 (2.07to13.44) | 1.06 (0.95to1.18) |
| Netherlands | 13.51 (2.87to38.05) | 66.36 (14.09to187.48) | | 8.95 (1.82to24.10) | 24.41 (5.09to66.72) | -4.22 (-4.80to-3.64) | | 0.95 (0.19to2.60) | 4.69 (0.97to12.67) | | 0.75 (0.16to1.90) | 1.94 (0.42to4.94) | -3.57 (-4.05to-3.09) |
| New Zealand | 6.26 (1.40to15.76) | 161.63 (36.48to402.39) | | 6.43 (1.90to13.68) | 77.90 (22.14to171.69) | -2.71 (-2.94to-2.47) | | 0.40 (0.10to0.93) | 10.56 (2.73to24.54) | | 0.54 (0.17to1.05) | 5.99 (1.84to11.96) | -2.18 (-2.33to-2.02) |
| Nicaragua | 0.34 (0.09to1.13) | 24.84 (6.35to81.77) | | 1.46 (0.36to4.50) | 41.48 (9.62to121.89) | 1.43 (1.00to1.86) | | 0.02 (0.01to0.07) | 1.74 (0.41to5.34) | | 0.10 (0.02to0.30) | 3.44 (0.77to9.79) | 1.98 (1.45to2.52) |
| Niger | 1.64 (0.41to4.38) | 73.00 (19.37to189.25) | | 4.35 (1.13to12.30) | 68.94 (18.95to184.47) | -0.16 (-0.26to-0.06) | | 0.08 (0.02to0.21) | 4.63 (1.34to11.49) | | 0.22 (0.06to0.58) | 4.47 (1.29to11.49) | -0.04 (-0.13to0.06) |
| Nigeria | 29.36 (7.95to84.56) | 77.06 (21.94to210.06) | | 49.31 (12.73to132.87) | 67.54 (18.65to170.40) | -0.69 (-0.86to-0.52) | | 1.55 (0.46to4.08) | 4.96 (1.48to12.37) | | 2.67 (0.76to6.61) | 4.49 (1.31to10.85) | -0.55 (-0.68to-0.41) |
| Niue | 0.00 (0.00to0.01) | 212.48 (66.84to492.53) | | 0.01 (0.00to0.01) | 236.87 (75.54to524.72) | 0.17 (0.01to0.33) | | 0.00 (0.00to0.00) | 13.26 (4.52to27.31) | | 0.00 (0.00to0.00) | 14.37 (5.08to28.52) | 0.11 (-0.03to0.24) |
| North Macedonia | 1.86 (0.59to4.63) | 115.46 (38.13to281.92) | | 2.87 (0.94to7.14) | 111.62 (37.27to267.34) | -0.38 (-0.66to-0.09) | | 0.11 (0.04to0.26) | 8.05 (2.80to18.53) | | 0.19 (0.06to0.45) | 9.06 (3.18to20.34) | 0.34 (-0.03to0.71) |
| Northern Mariana Islands | 0.02 (0.01to0.06) | 137.19 (45.00to301.20) | | 0.08 (0.02to0.20) | 174.58 (56.57to393.26) | 1.01 (0.91to1.10) | | 0.00 (0.00to0.00) | 8.24 (2.82to16.57) | | 0.00 (0.00to0.01) | 10.40 (3.76to21.13) | 0.99 (0.93to1.05) |
| Norway | 12.34 (3.11to28.35) | 174.79 (42.42to409.26) | | 5.80 (1.67to12.26) | 54.77 (15.21to119.39) | -4.36 (-4.49to-4.23) | | 0.85 (0.23to1.82) | 11.37 (3.12to24.65) | | 0.51 (0.16to1.00) | 4.24 (1.32to8.56) | -3.80 (-3.95to-3.64) |
| Oman | 4.39 (1.49to9.69) | 778.31 (310.65to1535.77) | | 7.86 (3.02to15.01) | 608.81 (276.53to1025.90) | -0.76 (-0.93to-0.59) | | 0.20 (0.08to0.39) | 47.03 (20.33to86.37) | | 0.36 (0.16to0.64) | 41.68 (20.93to66.69) | -0.31 (-0.50to-0.12) |
| Pakistan | 83.83 (25.34to195.18) | 156.75 (50.10to354.95) | | 211.74 (61.75to513.34) | 208.10 (65.05to469.87) | 0.74 (0.38to1.11) | | 4.27 (1.51to9.19) | 9.13 (3.40to19.41) | | 9.53 (3.03to21.27) | 11.98 (4.32to24.73) | 0.79 (0.49to1.09) |
| Palau | 0.02 (0.01to0.05) | 230.60 (66.68to548.34) | | 0.05 (0.01to0.12) | 251.18 (78.94to572.13) | 0.19 (0.09to0.29) | | 0.00 (0.00to0.00) | 13.26 (4.20to29.55) | | 0.00 (0.00to0.00) | 14.39 (5.08to29.85) | 0.17 (0.09to0.25) |
| Palestine | 3.76 (1.36to7.75) | 471.85 (183.30to933.69) | | 6.94 (2.50to13.90) | 344.68 (139.46to650.07) | -1.53 (-1.69to-1.37) | | 0.20 (0.09to0.38) | 28.64 (12.61to51.68) | | 0.36 (0.15to0.67) | 22.26 (10.11to38.15) | -1.26 (-1.43to-1.09) |
| Panama | 0.36 (0.10to1.19) | 25.47 (6.67to83.23) | | 0.67 (0.17to2.13) | 15.82 (4.00to50.47) | -1.48 (-1.75to-1.21) | | 0.02 (0.01to0.07) | 1.81 (0.44to5.56) | | 0.05 (0.01to0.15) | 1.19 (0.26to3.50) | -1.35 (-1.62to-1.08) |
| Papua New Guinea | 1.82 (0.38to5.23) | 112.22 (28.06to297.18) | | 6.24 (1.43to17.74) | 147.79 (40.49to377.48) | 1.07 (0.91to1.23) | | 0.07 (0.02to0.20) | 6.38 (1.71to15.47) | | 0.26 (0.07to0.68) | 8.63 (2.62to20.39) | 1.18 (1.02to1.33) |
| Paraguay | 1.16 (0.24to3.21) | 56.52 (11.90to156.46) | | 3.26 (0.71to9.20) | 61.61 (13.31to168.85) | 0.44 (0.13to0.74) | | 0.07 (0.02to0.20) | 3.91 (0.82to10.18) | | 0.21 (0.05to0.53) | 4.02 (0.91to10.16) | 0.17 (-0.12to0.46) |
| Peru | 6.38 (1.34to17.57) | 57.45 (11.91to154.53) | | 11.52 (2.57to29.63) | 35.46 (7.90to90.25) | -1.51 (-1.82to-1.20) | | 0.39 (0.08to1.01) | 3.85 (0.86to9.86) | | 0.82 (0.19to1.92) | 2.45 (0.58to5.80) | -1.41 (-1.69to-1.13) |
| Philippines | 5.96 (1.60to18.90) | 27.35 (6.94to81.75) | | 28.70 (7.52to97.79) | 41.45 (10.53to127.49) | 2.15 (1.72to2.60) | | 0.34 (0.08to1.00) | 2.16 (0.55to5.79) | | 1.48 (0.37to4.43) | 2.65 (0.65to7.66) | 1.42 (1.06to1.77) |
| Poland | 98.11 (31.37to231.43) | 238.69 (77.90to558.63) | | 69.89 (23.60to155.29) | 94.64 (31.32to212.76) | -3.58 (-3.77to-3.40) | | 6.19 (2.19to13.65) | 16.46 (5.97to35.67) | | 5.74 (2.08to11.91) | 7.49 (2.73to15.64) | -3.12 (-3.27to-2.97) |
| Portugal | 19.10 (5.53to41.03) | 147.36 (43.48to314.38) | | 15.49 (4.99to29.87) | 59.00 (18.06to122.93) | -3.94 (-4.32to-3.55) | | 1.24 (0.38to2.48) | 10.54 (3.40to20.48) | | 1.27 (0.44to2.31) | 4.22 (1.43to7.86) | -3.82 (-4.20to-3.43) |
| Puerto Rico | 7.31 (2.47to14.40) | 207.16 (70.50to408.09) | | 6.27 (2.22to12.40) | 83.82 (28.41to174.18) | -3.34 (-3.52to-3.16) | | 0.46 (0.17to0.85) | 14.02 (5.22to25.54) | | 0.46 (0.17to0.84) | 5.26 (1.97to9.87) | -3.65 (-3.82to-3.47) |
| Qatar | 0.69 (0.24to1.42) | 776.37 (331.92to1348.93) | | 2.43 (0.83to4.82) | 457.60 (219.73to737.37) | -2.07 (-2.35to-1.79) | | 0.03 (0.01to0.05) | 49.37 (23.66to80.12) | | 0.09 (0.04to0.17) | 35.25 (18.27to55.37) | -1.33 (-1.62to-1.05) |
| Republic of Korea | 29.25 (6.51to78.28) | 124.86 (31.28to299.27) | | 23.94 (6.01to54.74) | 28.72 (7.46to65.07) | -4.87 (-5.27to-4.48) | | 1.59 (0.40to3.79) | 9.24 (2.51to20.29) | | 1.91 (0.51to4.09) | 2.44 (0.67to5.20) | -4.42 (-4.80to-4.04) |
| Republic of Moldova | 7.55 (2.56to17.46) | 227.38 (79.96to500.10) | | 9.28 (3.22to20.86) | 159.08 (55.24to359.98) | -1.67 (-1.94to-1.40) | | 0.54 (0.19to1.19) | 19.32 (6.98to40.96) | | 0.76 (0.28to1.63) | 13.17 (4.78to28.10) | -1.82 (-2.09to-1.54) |
| Romania | 41.17 (14.55to94.49) | 176.40 (62.97to393.26) | | 49.66 (18.24to107.01) | 124.36 (45.86to273.87) | -1.67 (-1.90to-1.44) | | 2.77 (0.99to5.96) | 14.05 (5.21to29.44) | | 4.06 (1.55to8.27) | 9.96 (3.79to20.60) | -1.54 (-1.77to-1.32) |
| Russian Federation | 263.70 (88.73to619.79) | 167.31 (58.87to383.92) | | 337.82 (118.66to765.51) | 141.00 (49.58to325.14) | -0.56 (-1.02to-0.09) | | 17.68 (6.36to38.89) | 12.79 (4.66to27.59) | | 26.92 (9.94to56.97) | 11.40 (4.22to24.10) | -0.27 (-0.73to0.18) |
| Rwanda | 0.70 (0.20to2.40) | 27.64 (7.74to88.37) | | 1.01 (0.28to3.36) | 20.68 (5.73to62.15) | -1.56 (-1.79to-1.33) | | 0.03 (0.01to0.10) | 1.66 (0.46to5.08) | | 0.05 (0.01to0.16) | 1.39 (0.39to3.94) | -0.96 (-1.13to-0.80) |
| Saint Kitts and Nevis | 0.11 (0.03to0.23) | 296.56 (91.17to629.02) | | 0.08 (0.02to0.17) | 138.61 (42.36to283.12) | -2.65 (-2.90to-2.41) | | 0.01 (0.00to0.01) | 19.66 (6.53to38.25) | | 0.00 (0.00to0.01) | 9.30 (3.23to17.65) | -2.27 (-2.49to-2.05) |
| Saint Lucia | 0.14 (0.04to0.29) | 175.29 (53.32to369.28) | | 0.18 (0.06to0.37) | 86.84 (26.72to176.84) | -2.87 (-3.28to-2.45) | | 0.01 (0.00to0.02) | 13.08 (4.39to24.60) | | 0.01 (0.00to0.02) | 6.01 (2.05to11.23) | -3.27 (-3.71to-2.82) |
| Saint Vincent and the Grenadines | 0.15 (0.04to0.31) | 214.26 (64.03to447.86) | | 0.21 (0.07to0.43) | 169.20 (54.03to334.75) | -0.83 (-1.11to-0.54) | | 0.01 (0.00to0.02) | 15.20 (4.97to29.42) | | 0.01 (0.00to0.03) | 12.21 (4.26to22.30) | -0.71 (-1.01to-0.41) |
| Samoa | 0.19 (0.06to0.44) | 237.44 (81.72to525.75) | | 0.35 (0.11to0.79) | 255.38 (85.70to556.16) | 0.11 (-0.01to0.23) | | 0.01 (0.00to0.02) | 14.31 (5.37to28.58) | | 0.02 (0.01to0.04) | 15.33 (5.74to30.30) | 0.12 (0.03to0.21) |
| San Marino | 0.03 (0.01to0.06) | 81.65 (23.87to175.33) | | 0.04 (0.01to0.08) | 51.73 (14.70to118.76) | -1.61 (-1.77to-1.46) | | 0.00 (0.00to0.00) | 6.21 (2.01to12.14) | | 0.00 (0.00to0.01) | 3.94 (1.25to8.15) | -1.61 (-1.73to-1.48) |
| Sao Tome and Principe | 0.04 (0.01to0.09) | 63.94 (17.94to163.13) | | 0.08 (0.02to0.21) | 91.28 (26.67to228.98) | 1.13 (1.01to1.25) | | 0.00 (0.00to0.01) | 4.42 (1.34to10.69) | | 0.00 (0.00to0.01) | 6.33 (1.96to15.11) | 1.25 (1.18to1.33) |
| Saudi Arabia | 32.32 (12.81to61.37) | 572.67 (256.21to1009.26) | | 128.97 (55.75to217.23) | 630.23 (318.57to1003.06) | 1.18 (0.75to1.62) | | 1.42 (0.65to2.46) | 31.85 (15.86to51.69) | | 4.42 (2.09to7.18) | 32.27 (17.83to49.11) | 0.40 (0.13to0.67) |
| Senegal | 3.02 (0.78to7.55) | 105.21 (28.88to256.82) | | 6.42 (1.65to15.98) | 96.20 (26.42to232.74) | -0.36 (-0.47to-0.25) | | 0.15 (0.04to0.37) | 6.45 (1.95to15.21) | | 0.34 (0.10to0.82) | 6.06 (1.83to14.13) | -0.25 (-0.33to-0.18) |
| Serbia | 12.50 (4.15to29.72) | 128.75 (43.96to300.88) | | 15.82 (5.25to36.51) | 108.49 (36.64to249.38) | -0.99 (-1.20to-0.78) | | 0.86 (0.30to1.92) | 9.89 (3.49to22.05) | | 1.21 (0.42to2.70) | 9.35 (3.30to20.69) | -0.53 (-0.71to-0.35) |
| Seychelles | 0.06 (0.01to0.14) | 100.74 (25.55to252.69) | | 0.08 (0.02to0.20) | 78.79 (20.14to197.29) | -1.12 (-1.26to-0.98) | | 0.00 (0.00to0.01) | 6.42 (1.73to15.18) | | 0.00 (0.00to0.01) | 5.16 (1.40to11.96) | -0.89 (-1.03to-0.76) |
| Sierra Leone | 1.42 (0.38to3.83) | 81.84 (22.21to216.21) | | 2.57 (0.67to6.95) | 82.00 (22.41to209.84) | 0.29 (0.15to0.43) | | 0.08 (0.02to0.21) | 5.25 (1.50to13.17) | | 0.14 (0.04to0.34) | 5.22 (1.50to12.64) | 0.26 (0.13to0.38) |
| Singapore | 2.60 (0.58to6.88) | 130.67 (29.45to331.91) | | 3.38 (0.83to8.38) | 45.51 (11.34to109.57) | -3.90 (-4.04to-3.76) | | 0.14 (0.03to0.33) | 8.17 (1.96to18.88) | | 0.23 (0.06to0.50) | 3.21 (0.84to7.09) | -3.47 (-3.64to-3.30) |
| Slovakia | 11.45 (3.59to27.34) | 201.14 (64.92to473.60) | | 10.86 (3.54to25.04) | 121.10 (39.48to281.08) | -1.84 (-2.08to-1.59) | | 0.76 (0.26to1.70) | 14.31 (5.12to31.75) | | 0.87 (0.30to1.90) | 10.02 (3.48to21.73) | -1.22 (-1.42to-1.03) |
| Slovenia | 1.74 (0.54to4.42) | 74.43 (23.30to187.19) | | 1.80 (0.59to4.21) | 36.72 (11.82to88.37) | -2.99 (-3.28to-2.71) | | 0.12 (0.04to0.28) | 5.50 (1.76to12.84) | | 0.15 (0.05to0.34) | 2.82 (0.96to6.41) | -2.85 (-3.10to-2.60) |
| Solomon Islands | 0.45 (0.10to1.26) | 330.99 (82.95to846.66) | | 1.20 (0.29to3.24) | 386.39 (102.66to988.09) | 0.47 (0.31to0.62) | | 0.02 (0.00to0.04) | 16.17 (4.41to38.62) | | 0.04 (0.01to0.11) | 19.79 (5.81to44.99) | 0.61 (0.47to0.74) |
| Somalia | 0.63 (0.18to2.44) | 28.62 (8.34to95.73) | | 1.76 (0.51to7.34) | 30.34 (8.89to103.39) | 0.54 (0.42to0.67) | | 0.03 (0.01to0.09) | 1.64 (0.47to5.05) | | 0.07 (0.02to0.25) | 1.78 (0.52to5.39) | 0.67 (0.53to0.81) |
| South Africa | 19.41 (5.68to44.97) | 92.38 (28.77to208.38) | | 34.69 (10.93to77.59) | 84.98 (27.76to185.00) | -0.02 (-0.66to0.63) | | 0.90 (0.30to1.90) | 5.06 (1.68to10.48) | | 1.92 (0.65to3.95) | 5.56 (1.86to11.10) | 0.66 (0.05to1.28) |
| South Sudan | 0.46 (0.13to1.54) | 22.19 (6.26to70.04) | | 0.72 (0.19to2.41) | 22.38 (6.11to69.31) | 0.17 (0.08to0.26) | | 0.02 (0.01to0.07) | 1.40 (0.38to4.02) | | 0.04 (0.01to0.11) | 1.41 (0.38to4.06) | 0.19 (0.09to0.28) |
| Spain | 59.45 (15.29to133.27) | 112.53 (29.16to255.03) | | 54.30 (15.74to114.71) | 50.04 (13.03to112.24) | -3.23 (-3.48to-2.99) | | 4.08 (1.15to8.45) | 8.03 (2.31to16.54) | | 4.61 (1.42to8.97) | 3.58 (1.08to7.18) | -3.16 (-3.37to-2.95) |
| Sri Lanka | 7.07 (1.71to20.35) | 78.82 (19.48to212.40) | | 11.82 (2.87to33.67) | 52.10 (13.30to142.73) | -0.89 (-1.07to-0.70) | | 0.40 (0.09to1.07) | 5.45 (1.28to14.09) | | 0.74 (0.19to1.92) | 3.73 (0.91to9.66) | -0.60 (-0.84to-0.37) |
| Sudan | 94.17 (44.12to157.85) | 1025.53 (514.45to1653.44) | | 143.49 (69.45to247.29) | 775.27 (407.72to1254.64) | -1.14 (-1.22to-1.07) | | 4.03 (2.09to6.25) | 53.15 (29.11to80.41) | | 6.52 (3.59to10.27) | 42.78 (24.93to65.08) | -0.92 (-1.00to-0.85) |
| Suriname | 0.53 (0.14to1.19) | 215.96 (62.28to465.49) | | 0.88 (0.25to1.93) | 151.79 (44.17to323.98) | -1.34 (-1.76to-0.91) | | 0.03 (0.01to0.06) | 13.42 (4.14to26.44) | | 0.05 (0.02to0.10) | 8.95 (3.02to17.25) | -1.47 (-1.95to-0.99) |
| Sweden | 24.01 (5.81to57.28) | 147.19 (35.27to357.90) | | 13.05 (3.44to30.84) | 53.42 (13.40to130.79) | -4.58 (-4.92to-4.24) | | 1.82 (0.47to4.09) | 10.82 (2.81to24.38) | | 1.19 (0.33to2.66) | 4.34 (1.20to9.91) | -4.05 (-4.34to-3.76) |
| Switzerland | 15.09 (4.45to32.81) | 136.59 (38.84to304.67) | | 11.46 (3.66to23.35) | 55.12 (16.91to117.08) | -3.58 (-3.74to-3.42) | | 1.15 (0.36to2.31) | 10.13 (3.19to20.55) | | 1.11 (0.38to2.11) | 4.70 (1.58to9.19) | -3.12 (-3.32to-2.92) |
| Syrian Arab Republic | 34.73 (13.09to72.64) | 698.62 (287.76to1372.09) | | 68.26 (26.03to135.25) | 646.10 (273.99to1207.81) | -0.67 (-0.83to-0.50) | | 1.74 (0.76to3.21) | 41.66 (19.21to73.10) | | 3.49 (1.51to6.37) | 42.59 (20.26to72.28) | -0.26 (-0.42to-0.11) |
| Taiwan (Province of China) | 6.70 (1.73to17.04) | 57.33 (15.86to135.98) | | 13.43 (3.88to30.79) | 33.35 (9.65to77.01) | -1.80 (-2.00to-1.60) | | 0.41 (0.11to0.98) | 4.42 (1.27to9.91) | | 1.04 (0.31to2.17) | 2.50 (0.76to5.28) | -1.98 (-2.18to-1.78) |
| Tajikistan | 2.97 (0.93to7.47) | 111.29 (34.86to275.97) | | 6.56 (2.03to17.54) | 218.79 (71.62to515.38) | 2.55 (2.32to2.77) | | 0.20 (0.07to0.48) | 7.61 (2.52to18.29) | | 0.39 (0.13to0.93) | 17.13 (5.75to38.26) | 3.02 (2.69to3.34) |
| Thailand | 15.39 (3.78to40.75) | 53.82 (13.79to136.28) | | 37.63 (9.54to96.77) | 38.13 (9.91to96.24) | -1.71 (-1.88to-1.54) | | 0.91 (0.23to2.26) | 3.89 (1.05to9.23) | | 2.50 (0.68to5.87) | 2.59 (0.70to6.01) | -1.96 (-2.13to-1.79) |
| Timor-Leste | 0.12 (0.03to0.36) | 60.55 (16.40to157.34) | | 0.55 (0.13to1.52) | 83.34 (20.85to220.73) | 1.22 (1.08to1.36) | | 0.01 (0.00to0.02) | 4.13 (1.09to10.26) | | 0.03 (0.01to0.09) | 6.09 (1.55to15.44) | 1.50 (1.37to1.63) |
| Togo | 0.83 (0.23to2.30) | 80.86 (23.21to210.60) | | 2.57 (0.67to7.14) | 84.50 (23.73to213.61) | 0.20 (0.11to0.28) | | 0.04 (0.01to0.11) | 5.21 (1.51to12.72) | | 0.12 (0.03to0.32) | 5.35 (1.61to12.84) | 0.15 (0.10to0.21) |
| Tokelau | 0.00 (0.00to0.01) | 194.44 (57.27to467.78) | | 0.00 (0.00to0.01) | 212.34 (66.55to487.70) | 0.33 (0.18to0.49) | | 0.00 (0.00to0.00) | 11.50 (3.70to25.53) | | 0.00 (0.00to0.00) | 12.93 (4.54to26.12) | 0.51 (0.35to0.66) |
| Tonga | 0.06 (0.02to0.15) | 120.35 (36.51to282.62) | | 0.10 (0.03to0.24) | 132.88 (41.48to304.27) | 0.14 (-0.12to0.41) | | 0.00 (0.00to0.01) | 7.33 (2.45to15.62) | | 0.01 (0.00to0.01) | 8.21 (2.85to17.01) | 0.26 (0.02to0.50) |
| Trinidad and Tobago | 2.55 (0.79to5.44) | 324.63 (104.81to677.69) | | 3.68 (1.22to7.62) | 202.81 (68.31to415.30) | -2.22 (-2.46to-1.97) | | 0.14 (0.05to0.27) | 20.17 (7.54to37.96) | | 0.21 (0.08to0.40) | 12.22 (4.62to22.69) | -2.27 (-2.50to-2.03) |
| Tunisia | 6.31 (1.71to16.04) | 151.35 (43.00to371.01) | | 16.92 (4.95to40.31) | 147.77 (44.20to346.66) | -0.30 (-0.41to-0.19) | | 0.36 (0.10to0.87) | 10.59 (3.13to24.78) | | 1.11 (0.34to2.46) | 10.63 (3.31to22.74) | -0.46 (-0.76to-0.16) |
| Turkey | 113.13 (35.73to241.96) | 331.29 (110.65to667.45) | | 154.95 (59.55to298.80) | 183.36 (71.03to348.56) | -2.45 (-2.69to-2.20) | | 5.67 (2.00to11.05) | 18.89 (7.07to36.00) | | 9.75 (4.06to17.24) | 12.20 (5.18to21.37) | -1.59 (-1.85to-1.34) |
| Turkmenistan | 3.17 (1.00to7.80) | 206.64 (67.97to486.49) | | 7.24 (2.30to17.74) | 228.44 (75.53to530.91) | -0.58 (-0.98to-0.18) | | 0.20 (0.07to0.46) | 15.46 (5.31to34.43) | | 0.46 (0.16to1.04) | 16.65 (5.66to36.94) | -0.63 (-1.00to-0.27) |
| Tuvalu | 0.01 (0.00to0.04) | 226.90 (65.85to553.34) | | 0.02 (0.01to0.06) | 249.06 (78.65to582.93) | 0.28 (0.17to0.39) | | 0.00 (0.00to0.00) | 13.05 (4.21to29.34) | | 0.00 (0.00to0.00) | 15.04 (5.08to31.33) | 0.43 (0.31to0.55) |
| Uganda | 1.08 (0.30to3.56) | 19.88 (5.58to61.53) | | 2.61 (0.73to8.63) | 21.94 (6.19to64.12) | 0.15 (-0.10to0.41) | | 0.06 (0.02to0.17) | 1.30 (0.36to3.75) | | 0.14 (0.04to0.38) | 1.42 (0.41to3.86) | 0.21 (0.00to0.41) |
| Ukraine | 92.72 (29.74to221.07) | 142.55 (46.38to334.75) | | 132.41 (42.40to324.75) | 168.30 (53.99to417.61) | 0.10 (-0.23to0.44) | | 6.57 (2.20to14.99) | 11.26 (3.82to25.21) | | 9.88 (3.10to22.99) | 12.77 (4.03to29.73) | -0.04 (-0.36to0.27) |
| United Arab Emirates | 3.43 (1.32to6.09) | 732.79 (351.74to1153.00) | | 24.23 (8.86to45.35) | 471.27 (218.63to770.89) | -1.87 (-2.30to-1.43) | | 0.11 (0.05to0.19) | 41.90 (21.68to63.81) | | 0.67 (0.27to1.23) | 26.02 (13.00to40.84) | -1.96 (-2.48to-1.44) |
| United Kingdom | 201.54 (57.28to441.86) | 220.58 (61.07to494.33) | | 104.63 (34.29to215.79) | 79.27 (24.52to169.48) | -4.17 (-4.41to-3.92) | | 13.22 (4.10to26.90) | 14.26 (4.40to29.02) | | 8.12 (2.87to15.34) | 5.53 (1.93to10.56) | -3.90 (-4.14to-3.66) |
| United Republic of Tanzania | 1.88 (0.55to6.51) | 20.82 (6.00to66.39) | | 5.02 (1.45to16.18) | 23.98 (6.89to71.29) | 0.51 (0.47to0.54) | | 0.10 (0.03to0.30) | 1.36 (0.38to4.01) | | 0.28 (0.08to0.81) | 1.59 (0.45to4.39) | 0.56 (0.51to0.62) |
| United States of America | 449.83 (107.19to1109.63) | 136.83 (32.56to335.57) | | 309.64 (68.50to794.23) | 54.10 (11.72to142.82) | -3.57 (-3.80to-3.33) | | 32.23 (8.37to73.15) | 9.51 (2.48to21.63) | | 23.16 (5.52to56.48) | 3.61 (0.83to8.97) | -3.75 (-4.07to-3.43) |
| United States Virgin Islands | 0.11 (0.03to0.27) | 158.72 (44.52to373.30) | | 0.23 (0.07to0.52) | 135.42 (41.25to299.31) | -0.46 (-0.67to-0.25) | | 0.01 (0.00to0.02) | 11.74 (3.36to25.87) | | 0.02 (0.00to0.03) | 10.41 (3.23to21.37) | -0.39 (-0.61to-0.18) |
| Uruguay | 3.18 (0.53to9.43) | 83.79 (13.97to248.37) | | 2.74 (0.49to7.32) | 49.65 (8.62to134.80) | -1.74 (-1.88to-1.60) | | 0.19 (0.04to0.54) | 5.12 (0.97to14.47) | | 0.19 (0.04to0.47) | 2.95 (0.57to7.65) | -1.90 (-2.04to-1.77) |
| Uzbekistan | 15.33 (5.02to37.92) | 153.78 (50.38to373.93) | | 41.31 (12.27to106.77) | 392.77 (131.51to901.15) | 3.47 (2.94to4.01) | | 1.11 (0.37to2.52) | 11.94 (4.04to26.70) | | 2.22 (0.69to5.30) | 31.86 (11.06to68.85) | 3.67 (3.15to4.19) |
| Vanuatu | 0.08 (0.02to0.25) | 148.99 (39.80to403.82) | | 0.28 (0.08to0.78) | 183.92 (50.88to482.22) | 0.53 (0.41to0.66) | | 0.00 (0.00to0.01) | 9.04 (2.52to22.23) | | 0.01 (0.00to0.03) | 11.40 (3.26to26.92) | 0.69 (0.61to0.77) |
| Venezuela (Bolivarian Republic of) | 3.95 (1.09to13.43) | 43.80 (11.56to141.27) | | 10.10 (2.65to34.23) | 36.70 (9.40to118.27) | -0.94 (-1.14to-0.74) | | 0.22 (0.06to0.68) | 2.81 (0.68to8.47) | | 0.67 (0.16to2.08) | 2.51 (0.57to7.72) | -0.67 (-0.89to-0.44) |
| Viet Nam | 14.45 (3.69to41.25) | 40.49 (10.31to113.50) | | 31.27 (7.94to88.86) | 39.79 (9.81to111.49) | -0.10 (-0.21to0.02) | | 0.91 (0.22to2.49) | 2.84 (0.71to7.69) | | 2.10 (0.52to5.48) | 2.96 (0.74to7.68) | 0.07 (-0.03to0.18) |
| Yemen | 25.27 (8.14to56.88) | 566.97 (207.86to1189.52) | | 56.81 (20.06to126.85) | 476.12 (188.13to965.75) | -0.74 (-0.85to-0.62) | | 1.11 (0.42to2.29) | 32.20 (13.38to60.51) | | 2.73 (1.12to5.40) | 28.97 (12.69to52.32) | -0.47 (-0.56to-0.37) |
| Zambia | 1.25 (0.32to3.66) | 48.94 (12.89to137.70) | | 2.68 (0.65to7.73) | 45.55 (11.62to120.03) | -0.47 (-0.70to-0.24) | | 0.06 (0.01to0.16) | 2.78 (0.74to7.55) | | 0.12 (0.03to0.32) | 2.73 (0.75to6.91) | -0.24 (-0.43to-0.04) |
| Zimbabwe | 1.43 (0.40to4.22) | 42.01 (11.84to117.29) | | 3.35 (0.88to10.01) | 56.17 (15.02to160.82) | 1.22 (1.05to1.39) | | 0.07 (0.02to0.20) | 2.70 (0.76to7.21) | | 0.17 (0.04to0.47) | 3.49 (0.97to9.47) | 1.08 (0.92to1.24) |

| **Supplemental table 5. DALYs and mortality of stroke attributable to LPA in 204 countries and territories in 1990 and 2019** | | | | | | | | | | | | | |
| --- | --- | --- | --- | --- | --- | --- | --- | --- | --- | --- | --- | --- | --- |
|  | **DALYs (95% UI)** | | | | | |  | **Mortality (95% UI)** | | | | | |
|  | **1990** | |  | **2019** | | **EAPC** |  | **1990** | |  | **2019** | | **EAPC** |
| **location** | **Number(no.×10^3^)** | **ASR per100,000** |  | **Number(no.×10^3^)** | **ASR per100,000** |  |  | **Number(no.×10^3^)** | **ASR per100,000** |  | **Number(no.×10^3^)** | **ASR per100,000** |  |
| Afghanistan | 5.63 (1.03to14.07) | 98.56 (19.19to231.34) | | 13.66 (2.28to36.26) | 129.89 (25.13to306.86) | 1.21 (1.01to1.41) | | 0.29 (0.06to0.67) | 6.37 (1.35to14.19) | | 0.63 (0.13to1.48) | 8.11 (1.72to17.97) | 1.04 (0.87to1.21) |
| Albania | 0.32 (0.06to0.99) | 18.63 (3.59to54.74) | | 0.71 (0.14to2.02) | 17.03 (3.31to49.02) | 0.13 (-0.15to0.41) | | 0.02 (0.00to0.05) | 1.27 (0.26to3.49) | | 0.05 (0.01to0.13) | 1.17 (0.24to3.29) | 0.19 (-0.12to0.50) |
| Algeria | 10.59 (1.93to25.47) | 138.23 (28.28to301.86) | | 29.63 (6.02to65.67) | 110.29 (23.46to231.60) | -0.61 (-0.68to-0.55) | | 0.56 (0.11to1.22) | 10.67 (2.30to22.03) | | 1.76 (0.40to3.54) | 8.23 (1.91to16.18) | -0.68 (-0.75to-0.61) |
| American Samoa | 0.01 (0.00to0.02) | 48.99 (8.50to124.73) | | 0.02 (0.00to0.05) | 46.16 (7.69to114.85) | -0.51 (-0.66to-0.35) | | 0.00 (0.00to0.00) | 2.86 (0.54to6.80) | | 0.00 (0.00to0.00) | 2.55 (0.48to5.88) | -0.70 (-0.86to-0.53) |
| Andorra | 0.01 (0.00to0.03) | 22.14 (2.93to64.13) | | 0.02 (0.00to0.06) | 14.36 (1.99to36.89) | -1.59 (-1.77to-1.42) | | 0.00 (0.00to0.00) | 1.68 (0.23to4.71) | | 0.00 (0.00to0.00) | 1.04 (0.15to2.54) | -1.69 (-1.89to-1.49) |
| Angola | 0.86 (0.12to2.77) | 30.23 (4.61to88.47) | | 2.69 (0.38to8.01) | 34.28 (5.84to96.52) | 0.33 (0.26to0.40) | | 0.04 (0.01to0.11) | 1.79 (0.29to4.98) | | 0.12 (0.02to0.35) | 2.21 (0.40to6.06) | 0.62 (0.52to0.72) |
| Antigua and Barbuda | 0.03 (0.00to0.06) | 42.82 (6.30to112.34) | | 0.03 (0.00to0.08) | 36.99 (5.26to90.85) | -0.87 (-1.11to-0.64) | | 0.00 (0.00to0.00) | 3.23 (0.45to8.05) | | 0.00 (0.00to0.01) | 2.90 (0.40to6.78) | -0.78 (-1.03to-0.53) |
| Argentina | 2.81 (0.57to12.66) | 9.39 (1.85to41.84) | | 3.07 (0.54to12.67) | 5.55 (0.97to23.29) | -1.56 (-1.89to-1.24) | | 0.17 (0.03to0.70) | 0.62 (0.12to2.56) | | 0.21 (0.03to0.76) | 0.37 (0.06to1.36) | -1.55 (-1.93to-1.18) |
| Armenia | 0.84 (0.16to2.57) | 38.48 (7.53to114.37) | | 1.01 (0.19to2.90) | 24.60 (4.60to70.70) | -2.22 (-2.52to-1.92) | | 0.05 (0.01to0.15) | 2.77 (0.53to7.53) | | 0.07 (0.01to0.20) | 1.82 (0.36to4.96) | -2.22 (-2.55to-1.89) |
| Australia | 6.34 (0.84to17.56) | 34.93 (4.75to95.49) | | 7.58 (1.07to19.33) | 16.13 (2.21to42.76) | -3.20 (-3.36to-3.03) | | 0.47 (0.06to1.25) | 2.82 (0.37to7.32) | | 0.66 (0.09to1.61) | 1.27 (0.18to3.10) | -3.37 (-3.60to-3.15) |
| Austria | 6.14 (0.94to16.17) | 48.27 (7.35to127.16) | | 3.10 (0.50to7.90) | 14.67 (2.39to38.45) | -4.62 (-4.89to-4.35) | | 0.49 (0.07to1.26) | 3.96 (0.61to10.16) | | 0.25 (0.04to0.60) | 1.06 (0.16to2.56) | -5.16 (-5.45to-4.86) |
| Azerbaijan | 0.86 (0.16to2.77) | 19.25 (3.78to58.24) | | 2.21 (0.37to6.84) | 34.85 (6.35to98.86) | 2.93 (2.52to3.35) | | 0.05 (0.01to0.14) | 1.18 (0.23to3.20) | | 0.13 (0.02to0.37) | 2.69 (0.53to7.30) | 3.97 (3.47to4.48) |
| Bahamas | 0.05 (0.01to0.15) | 41.33 (5.90to106.64) | | 0.11 (0.02to0.28) | 32.99 (4.72to83.18) | -0.84 (-0.98to-0.71) | | 0.00 (0.00to0.01) | 2.97 (0.43to7.29) | | 0.01 (0.00to0.02) | 2.35 (0.36to5.48) | -0.95 (-1.13to-0.77) |
| Bahrain | 0.11 (0.02to0.26) | 91.44 (20.66to194.33) | | 0.32 (0.05to0.76) | 56.44 (12.52to117.47) | -1.56 (-1.83to-1.28) | | 0.00 (0.00to0.01) | 6.29 (1.54to12.99) | | 0.01 (0.00to0.03) | 4.03 (0.97to8.00) | -1.26 (-1.62to-0.90) |
| Bangladesh | 12.90 (2.09to37.18) | 35.76 (6.09to98.35) | | 38.96 (6.99to108.66) | 36.16 (6.66to97.92) | 0.21 (-0.40to0.82) | | 0.83 (0.14to2.25) | 2.76 (0.49to7.28) | | 2.77 (0.53to7.40) | 2.94 (0.57to7.68) | 0.43 (-0.27to1.13) |
| Barbados | 0.21 (0.03to0.50) | 65.07 (10.35to158.00) | | 0.23 (0.03to0.53) | 46.10 (7.25to108.60) | -1.43 (-1.67to-1.19) | | 0.02 (0.00to0.04) | 5.17 (0.79to11.86) | | 0.02 (0.00to0.04) | 3.60 (0.56to8.04) | -1.46 (-1.70to-1.23) |
| Belarus | 6.00 (1.22to17.27) | 48.15 (9.86to137.62) | | 7.17 (1.47to20.14) | 42.63 (8.80to122.25) | -0.95 (-1.28to-0.62) | | 0.40 (0.08to1.05) | 3.38 (0.71to8.66) | | 0.54 (0.11to1.38) | 3.15 (0.66to8.09) | -0.73 (-1.03to-0.44) |
| Belgium | 6.85 (1.00to18.16) | 42.91 (6.41to114.14) | | 5.17 (0.76to12.63) | 18.26 (2.57to46.84) | -3.23 (-3.43to-3.04) | | 0.53 (0.08to1.35) | 3.43 (0.50to8.65) | | 0.45 (0.07to1.07) | 1.41 (0.21to3.37) | -3.33 (-3.48to-3.17) |
| Belize | 0.03 (0.00to0.07) | 30.69 (4.69to79.50) | | 0.07 (0.01to0.17) | 27.67 (4.13to70.38) | -0.51 (-0.86to-0.15) | | 0.00 (0.00to0.00) | 2.28 (0.33to5.65) | | 0.00 (0.00to0.01) | 1.99 (0.28to4.71) | -0.60 (-0.98to-0.21) |
| Benin | 0.45 (0.06to1.42) | 25.28 (3.83to77.70) | | 0.96 (0.14to3.04) | 23.88 (3.76to72.51) | -0.29 (-0.37to-0.22) | | 0.02 (0.00to0.07) | 1.57 (0.25to4.44) | | 0.05 (0.01to0.15) | 1.55 (0.27to4.54) | -0.11 (-0.19to-0.04) |
| Bermuda | 0.03 (0.00to0.07) | 47.39 (7.10to118.31) | | 0.03 (0.01to0.08) | 23.06 (3.73to56.40) | -2.61 (-2.74to-2.47) | | 0.00 (0.00to0.00) | 3.58 (0.54to8.29) | | 0.00 (0.00to0.01) | 1.74 (0.27to3.92) | -2.66 (-2.78to-2.54) |
| Bhutan | 0.05 (0.01to0.14) | 33.13 (7.51to81.01) | | 0.14 (0.03to0.36) | 30.90 (6.29to76.20) | -0.13 (-0.17to-0.08) | | 0.00 (0.00to0.01) | 2.43 (0.53to5.86) | | 0.01 (0.00to0.02) | 2.34 (0.51to5.60) | -0.08 (-0.11to-0.04) |
| Bolivia (Plurinational State of) | 0.45 (0.05to1.61) | 16.78 (1.90to57.12) | | 1.12 (0.12to3.65) | 15.27 (1.59to48.87) | -0.47 (-0.71to-0.23) | | 0.02 (0.00to0.09) | 1.14 (0.13to3.83) | | 0.07 (0.01to0.23) | 1.13 (0.11to3.72) | -0.21 (-0.44to0.02) |
| Bosnia and Herzegovina | 1.67 (0.29to5.39) | 49.08 (8.85to152.07) | | 2.93 (0.52to8.57) | 51.39 (9.21to148.49) | 0.03 (-0.29to0.35) | | 0.09 (0.02to0.26) | 3.15 (0.61to9.06) | | 0.20 (0.04to0.56) | 3.71 (0.70to10.49) | 0.64 (0.25to1.03) |
| Botswana | 0.19 (0.03to0.59) | 43.16 (6.48to127.82) | | 0.47 (0.07to1.37) | 46.30 (7.27to136.06) | 0.04 (-0.21to0.28) | | 0.01 (0.00to0.03) | 2.84 (0.45to8.15) | | 0.02 (0.00to0.07) | 3.13 (0.53to8.92) | 0.14 (-0.10to0.37) |
| Brazil | 82.06 (12.09to196.39) | 111.37 (17.63to249.79) | | 123.13 (25.96to251.45) | 55.61 (12.00to111.97) | -2.34 (-2.48to-2.21) | | 4.54 (0.72to9.85) | 7.64 (1.34to15.79) | | 8.33 (1.98to15.99) | 3.90 (0.94to7.44) | -2.23 (-2.38to-2.08) |
| Brunei Darussalam | 0.04 (0.00to0.13) | 59.61 (6.38to186.12) | | 0.06 (0.01to0.20) | 33.01 (3.66to98.79) | -2.28 (-2.44to-2.12) | | 0.00 (0.00to0.01) | 3.84 (0.44to11.25) | | 0.00 (0.00to0.01) | 2.17 (0.25to6.10) | -2.25 (-2.49to-2.02) |
| Bulgaria | 6.73 (1.30to19.68) | 65.44 (12.99to185.73) | | 10.00 (2.11to27.13) | 66.39 (13.84to183.08) | 0.10 (-0.09to0.28) | | 0.40 (0.08to1.13) | 4.74 (1.00to12.75) | | 0.75 (0.16to2.02) | 5.10 (1.11to13.48) | 0.29 (0.11to0.46) |
| Burkina Faso | 0.33 (0.05to1.32) | 9.51 (1.60to34.15) | | 0.78 (0.13to2.83) | 10.35 (1.82to36.11) | 0.34 (0.17to0.50) | | 0.01 (0.00to0.05) | 0.58 (0.10to1.96) | | 0.04 (0.01to0.12) | 0.66 (0.12to2.13) | 0.62 (0.41to0.82) |
| Burundi | 0.29 (0.06to1.20) | 14.15 (2.72to56.27) | | 0.40 (0.07to1.78) | 11.65 (2.20to44.89) | -0.83 (-0.89to-0.77) | | 0.01 (0.00to0.05) | 0.80 (0.16to2.92) | | 0.02 (0.00to0.07) | 0.74 (0.14to2.58) | -0.38 (-0.43to-0.34) |
| Cabo Verde | 0.03 (0.00to0.08) | 10.67 (1.56to33.98) | | 0.08 (0.01to0.24) | 17.77 (2.70to54.34) | 1.16 (0.90to1.42) | | 0.00 (0.00to0.00) | 0.58 (0.10to1.73) | | 0.01 (0.00to0.02) | 1.19 (0.19to3.53) | 1.73 (1.40to2.07) |
| Cambodia | 0.91 (0.12to3.26) | 27.91 (3.53to90.52) | | 2.56 (0.34to8.48) | 28.43 (3.69to91.69) | -0.05 (-0.12to0.02) | | 0.05 (0.01to0.15) | 1.87 (0.23to5.99) | | 0.15 (0.02to0.50) | 2.05 (0.26to6.45) | 0.20 (0.13to0.27) |
| Cameroon | 0.71 (0.09to2.22) | 19.96 (2.89to59.52) | | 2.21 (0.29to6.86) | 22.84 (3.43to67.80) | 0.45 (0.25to0.66) | | 0.03 (0.01to0.10) | 1.27 (0.20to3.64) | | 0.10 (0.02to0.31) | 1.47 (0.25to4.23) | 0.51 (0.30to0.72) |
| Canada | 7.41 (0.87to22.30) | 23.74 (2.75to70.95) | | 9.95 (1.33to27.73) | 13.22 (1.77to37.65) | -2.54 (-2.75to-2.32) | | 0.49 (0.06to1.46) | 1.66 (0.19to4.87) | | 0.73 (0.10to1.99) | 0.87 (0.12to2.37) | -2.93 (-3.16to-2.70) |
| Central African Republic | 0.29 (0.04to0.93) | 35.12 (5.52to100.88) | | 0.56 (0.08to1.75) | 37.74 (5.93to107.77) | 0.37 (0.31to0.44) | | 0.01 (0.00to0.04) | 2.13 (0.34to5.72) | | 0.02 (0.00to0.07) | 2.41 (0.42to6.73) | 0.59 (0.52to0.66) |
| Chad | 0.42 (0.06to1.49) | 17.10 (2.58to57.91) | | 0.80 (0.12to2.74) | 17.05 (2.68to55.22) | 0.06 (0.01to0.10) | | 0.02 (0.00to0.07) | 1.04 (0.17to3.21) | | 0.04 (0.01to0.12) | 1.06 (0.18to3.21) | 0.11 (0.08to0.14) |
| Chile | 1.99 (0.23to6.60) | 22.44 (2.68to72.60) | | 3.42 (0.40to10.84) | 14.42 (1.68to45.63) | -1.27 (-1.39to-1.16) | | 0.12 (0.01to0.38) | 1.51 (0.18to4.79) | | 0.24 (0.03to0.77) | 1.03 (0.13to3.27) | -0.93 (-1.09to-0.77) |
| China | 265.61 (40.30to787.67) | 42.46 (6.75to113.80) | | 655.90 (102.49to1916.24) | 38.31 (6.04to107.81) | -0.66 (-0.80to-0.53) | | 13.69 (2.10to38.32) | 2.87 (0.47to7.73) | | 38.30 (5.96to107.98) | 2.57 (0.42to7.08) | -0.67 (-0.90to-0.44) |
| Colombia | 2.68 (0.29to9.37) | 17.31 (1.93to58.07) | | 4.29 (0.44to13.81) | 7.85 (0.80to25.12) | -3.09 (-3.31to-2.87) | | 0.14 (0.02to0.47) | 1.12 (0.13to3.64) | | 0.30 (0.03to0.95) | 0.52 (0.05to1.67) | -3.06 (-3.24to-2.88) |
| Comoros | 0.02 (0.00to0.11) | 11.48 (2.51to50.28) | | 0.05 (0.01to0.20) | 10.68 (2.41to43.94) | -0.41 (-0.50to-0.32) | | 0.00 (0.00to0.00) | 0.65 (0.14to2.61) | | 0.00 (0.00to0.01) | 0.66 (0.15to2.48) | -0.08 (-0.15to-0.01) |
| Congo | 0.37 (0.05to1.09) | 44.78 (6.88to125.70) | | 0.83 (0.12to2.38) | 42.77 (7.22to119.26) | -0.24 (-0.31to-0.16) | | 0.02 (0.00to0.05) | 2.72 (0.46to7.46) | | 0.04 (0.01to0.11) | 2.78 (0.49to7.47) | 0.03 (-0.04to0.09) |
| Cook Islands | 0.00 (0.00to0.01) | 36.36 (5.45to97.71) | | 0.01 (0.00to0.02) | 33.55 (6.23to86.72) | -0.44 (-0.56to-0.33) | | 0.00 (0.00to0.00) | 2.02 (0.32to5.35) | | 0.00 (0.00to0.00) | 1.73 (0.31to4.14) | -0.69 (-0.83to-0.56) |
| Costa Rica | 0.14 (0.02to0.51) | 8.38 (1.15to30.67) | | 0.31 (0.04to1.09) | 6.13 (0.74to21.42) | -1.60 (-1.98to-1.22) | | 0.01 (0.00to0.03) | 0.60 (0.08to2.14) | | 0.02 (0.00to0.08) | 0.44 (0.05to1.49) | -1.68 (-2.08to-1.27) |
| Côte d'Ivoire | 0.81 (0.10to2.67) | 25.08 (3.61to74.99) | | 1.91 (0.25to6.26) | 22.34 (3.33to66.58) | -0.40 (-0.52to-0.28) | | 0.03 (0.00to0.09) | 1.52 (0.24to4.40) | | 0.08 (0.01to0.25) | 1.40 (0.23to4.01) | -0.30 (-0.40to-0.20) |
| Croatia | 2.84 (0.57to8.28) | 50.25 (10.26to142.46) | | 2.89 (0.59to7.91) | 29.46 (6.01to79.22) | -1.94 (-2.21to-1.67) | | 0.19 (0.04to0.53) | 3.78 (0.79to10.33) | | 0.23 (0.05to0.61) | 2.35 (0.50to6.24) | -1.67 (-1.97to-1.36) |
| Cuba | 3.82 (0.50to10.12) | 38.25 (5.06to100.94) | | 7.20 (1.08to17.68) | 36.27 (5.26to90.24) | -0.16 (-0.29to-0.03) | | 0.25 (0.03to0.63) | 2.69 (0.36to6.79) | | 0.55 (0.08to1.25) | 2.61 (0.38to5.96) | -0.12 (-0.25to0.01) |
| Cyprus | 0.27 (0.03to0.82) | 48.64 (5.51to141.16) | | 0.34 (0.04to0.96) | 20.46 (2.55to55.56) | -3.38 (-3.54to-3.22) | | 0.02 (0.00to0.06) | 4.32 (0.48to12.48) | | 0.03 (0.00to0.08) | 1.90 (0.24to5.03) | -3.17 (-3.35to-2.99) |
| Czechia | 9.99 (1.99to27.52) | 75.48 (15.09to205.80) | | 5.79 (1.14to15.41) | 26.61 (5.18to70.82) | -3.98 (-4.26to-3.70) | | 0.70 (0.14to1.82) | 5.74 (1.22to14.92) | | 0.45 (0.09to1.18) | 2.03 (0.41to5.28) | -4.08 (-4.46to-3.70) |
| Democratic People's Republic of Korea | 5.23 (0.79to16.85) | 43.47 (6.65to130.40) | | 12.27 (1.87to37.66) | 41.74 (6.49to125.57) | -0.12 (-0.27to0.04) | | 0.25 (0.04to0.74) | 2.63 (0.41to7.39) | | 0.66 (0.10to1.91) | 2.50 (0.39to7.05) | -0.15 (-0.35to0.06) |
| Democratic Republic of the Congo | 3.37 (0.45to10.18) | 30.21 (4.63to85.84) | | 7.90 (1.23to22.93) | 30.09 (5.29to84.51) | -0.07 (-0.12to-0.02) | | 0.14 (0.02to0.42) | 1.91 (0.31to5.32) | | 0.41 (0.07to1.13) | 2.01 (0.37to5.50) | 0.13 (0.08to0.18) |
| Denmark | 2.98 (0.38to8.76) | 34.03 (4.29to101.11) | | 2.16 (0.29to6.11) | 17.16 (2.24to49.08) | -2.81 (-3.06to-2.56) | | 0.22 (0.03to0.64) | 2.53 (0.33to7.22) | | 0.18 (0.03to0.49) | 1.34 (0.19to3.60) | -2.59 (-2.82to-2.36) |
| Djibouti | 0.01 (0.00to0.06) | 13.36 (2.65to50.29) | | 0.07 (0.01to0.29) | 15.25 (2.88to58.35) | 0.50 (0.45to0.56) | | 0.00 (0.00to0.00) | 0.77 (0.15to2.73) | | 0.00 (0.00to0.01) | 0.94 (0.18to3.37) | 0.76 (0.69to0.82) |
| Dominica | 0.03 (0.00to0.08) | 40.38 (5.33to105.76) | | 0.04 (0.01to0.09) | 40.97 (6.54to102.70) | 0.32 (0.20to0.44) | | 0.00 (0.00to0.01) | 3.07 (0.44to7.86) | | 0.00 (0.00to0.01) | 3.16 (0.46to7.63) | 0.42 (0.29to0.55) |
| Dominican Republic | 1.25 (0.19to3.21) | 38.60 (6.39to95.95) | | 4.64 (0.65to12.08) | 53.10 (7.66to136.10) | 1.77 (1.58to1.96) | | 0.07 (0.01to0.17) | 2.73 (0.46to6.21) | | 0.29 (0.05to0.70) | 3.59 (0.57to8.35) | 1.87 (1.62to2.11) |
| Ecuador | 0.77 (0.08to2.51) | 15.70 (1.66to50.67) | | 1.82 (0.20to5.49) | 13.39 (1.50to39.96) | -0.61 (-0.80to-0.41) | | 0.04 (0.00to0.13) | 1.05 (0.11to3.22) | | 0.12 (0.01to0.36) | 0.99 (0.11to3.00) | -0.17 (-0.37to0.03) |
| Egypt | 17.73 (3.17to45.13) | 73.63 (13.88to175.55) | | 55.49 (8.91to143.79) | 100.02 (17.45to238.36) | 1.51 (1.30to1.72) | | 0.80 (0.16to1.88) | 4.35 (0.93to9.57) | | 2.25 (0.39to5.42) | 5.34 (1.07to12.23) | 1.19 (0.95to1.43) |
| El Salvador | 0.23 (0.03to0.87) | 7.98 (1.17to30.58) | | 0.38 (0.05to1.34) | 6.03 (0.75to21.34) | -1.15 (-1.45to-0.86) | | 0.01 (0.00to0.05) | 0.52 (0.07to1.88) | | 0.03 (0.00to0.10) | 0.40 (0.05to1.41) | -1.08 (-1.39to-0.77) |
| Equatorial Guinea | 0.05 (0.01to0.16) | 32.71 (5.04to95.50) | | 0.14 (0.02to0.40) | 37.26 (6.29to106.52) | 0.40 (0.31to0.48) | | 0.00 (0.00to0.01) | 1.94 (0.31to5.40) | | 0.01 (0.00to0.02) | 2.45 (0.44to6.81) | 0.76 (0.69to0.83) |
| Eritrea | 0.09 (0.02to0.38) | 11.81 (2.19to44.17) | | 0.26 (0.05to1.08) | 13.82 (2.61to49.14) | 0.53 (0.48to0.58) | | 0.00 (0.00to0.01) | 0.65 (0.12to2.28) | | 0.01 (0.00to0.04) | 0.88 (0.17to3.09) | 1.06 (0.96to1.15) |
| Estonia | 1.26 (0.25to3.69) | 63.54 (12.96to184.12) | | 0.54 (0.11to1.42) | 17.34 (3.59to47.96) | -5.67 (-6.20to-5.13) | | 0.09 (0.02to0.24) | 4.79 (0.99to12.91) | | 0.04 (0.01to0.11) | 1.27 (0.27to3.29) | -5.89 (-6.49to-5.28) |
| Eswatini | 0.09 (0.01to0.26) | 39.19 (6.01to116.68) | | 0.19 (0.03to0.57) | 44.53 (7.06to128.70) | 0.52 (0.21to0.84) | | 0.00 (0.00to0.01) | 2.58 (0.42to7.45) | | 0.01 (0.00to0.03) | 3.04 (0.52to8.64) | 0.67 (0.35to1.00) |
| Ethiopia | 1.28 (0.22to5.45) | 8.30 (1.48to32.76) | | 2.61 (0.49to10.59) | 7.59 (1.47to28.83) | -0.43 (-0.49to-0.36) | | 0.05 (0.01to0.19) | 0.48 (0.09to1.82) | | 0.13 (0.03to0.48) | 0.50 (0.10to1.80) | 0.07 (0.04to0.11) |
| Fiji | 0.13 (0.02to0.38) | 45.15 (6.62to122.42) | | 0.27 (0.04to0.76) | 48.09 (8.10to129.28) | -0.25 (-0.46to-0.03) | | 0.00 (0.00to0.01) | 2.60 (0.42to6.84) | | 0.01 (0.00to0.03) | 2.80 (0.51to7.10) | -0.32 (-0.59to-0.06) |
| Finland | 3.11 (0.38to9.20) | 43.43 (5.34to128.27) | | 2.91 (0.41to8.06) | 20.20 (2.67to57.50) | -2.95 (-3.17to-2.72) | | 0.22 (0.03to0.64) | 3.19 (0.42to9.28) | | 0.25 (0.03to0.67) | 1.58 (0.22to4.23) | -2.73 (-2.93to-2.53) |
| France | 34.52 (4.73to85.16) | 37.62 (5.28to93.81) | | 28.48 (4.34to66.50) | 16.21 (2.48to39.84) | -3.06 (-3.27to-2.85) | | 2.89 (0.42to6.76) | 3.17 (0.48to7.38) | | 2.55 (0.38to5.72) | 1.21 (0.18to2.75) | -3.53 (-3.75to-3.30) |
| Gabon | 0.15 (0.02to0.44) | 33.16 (5.35to91.81) | | 0.29 (0.05to0.85) | 35.97 (6.48to100.34) | 0.25 (0.08to0.41) | | 0.01 (0.00to0.02) | 2.10 (0.36to5.61) | | 0.02 (0.00to0.05) | 2.39 (0.46to6.66) | 0.42 (0.26to0.59) |
| Gambia | 0.06 (0.01to0.22) | 21.53 (3.40to66.39) | | 0.20 (0.03to0.63) | 24.00 (3.87to72.16) | 0.35 (0.26to0.43) | | 0.00 (0.00to0.01) | 1.33 (0.23to3.80) | | 0.01 (0.00to0.03) | 1.55 (0.27to4.54) | 0.55 (0.49to0.61) |
| Georgia | 2.27 (0.43to6.69) | 44.08 (8.40to125.92) | | 2.49 (0.46to7.10) | 37.21 (6.82to105.79) | -0.27 (-0.67to0.13) | | 0.16 (0.03to0.43) | 3.50 (0.65to9.53) | | 0.20 (0.04to0.54) | 2.76 (0.51to7.61) | -0.57 (-1.00to-0.15) |
| Germany | 49.11 (5.61to156.41) | 36.32 (4.23to116.10) | | 34.32 (3.88to100.42) | 15.05 (1.83to45.60) | -3.50 (-3.85to-3.15) | | 3.74 (0.43to11.51) | 2.83 (0.33to8.69) | | 2.76 (0.33to8.03) | 1.11 (0.14to3.25) | -3.70 (-4.02to-3.38) |
| Ghana | 1.63 (0.24to5.36) | 32.58 (5.10to98.98) | | 5.00 (0.74to15.63) | 38.16 (6.20to113.43) | 0.71 (0.62to0.80) | | 0.07 (0.01to0.23) | 2.08 (0.35to6.19) | | 0.25 (0.04to0.73) | 2.50 (0.44to7.04) | 0.85 (0.74to0.96) |
| Greece | 6.46 (0.70to20.20) | 44.19 (4.82to136.71) | | 7.06 (0.83to22.20) | 21.10 (2.48to65.72) | -3.50 (-3.92to-3.08) | | 0.53 (0.06to1.70) | 3.91 (0.42to12.51) | | 0.68 (0.08to2.09) | 1.90 (0.23to5.88) | -3.46 (-3.92to-2.99) |
| Greenland | 0.01 (0.00to0.04) | 56.63 (6.61to170.21) | | 0.01 (0.00to0.05) | 28.60 (3.24to85.60) | -3.14 (-3.46to-2.83) | | 0.00 (0.00to0.00) | 4.01 (0.46to12.01) | | 0.00 (0.00to0.00) | 1.96 (0.22to5.79) | -3.41 (-3.76to-3.05) |
| Grenada | 0.06 (0.01to0.17) | 76.99 (10.08to210.99) | | 0.05 (0.01to0.13) | 53.12 (7.89to134.74) | -1.33 (-1.55to-1.10) | | 0.00 (0.00to0.01) | 5.60 (0.76to14.29) | | 0.00 (0.00to0.01) | 3.98 (0.58to9.80) | -1.24 (-1.46to-1.03) |
| Guam | 0.02 (0.00to0.06) | 36.09 (5.55to99.69) | | 0.05 (0.01to0.13) | 25.82 (3.89to69.65) | -1.25 (-1.57to-0.92) | | 0.00 (0.00to0.00) | 2.25 (0.37to5.90) | | 0.00 (0.00to0.01) | 1.13 (0.19to3.01) | -2.78 (-3.34to-2.23) |
| Guatemala | 0.14 (0.03to0.74) | 4.92 (1.12to24.08) | | 0.39 (0.08to1.94) | 3.91 (0.81to18.23) | -1.43 (-1.69to-1.18) | | 0.01 (0.00to0.03) | 0.33 (0.07to1.54) | | 0.02 (0.00to0.10) | 0.29 (0.06to1.27) | -1.10 (-1.37to-0.83) |
| Guinea | 0.50 (0.07to1.64) | 17.29 (2.64to56.21) | | 0.89 (0.14to2.96) | 18.52 (2.96to58.36) | 0.47 (0.39to0.55) | | 0.03 (0.00to0.08) | 1.08 (0.18to3.32) | | 0.05 (0.01to0.15) | 1.19 (0.20to3.63) | 0.61 (0.53to0.69) |
| Guinea-Bissau | 0.09 (0.01to0.30) | 25.42 (3.70to83.91) | | 0.16 (0.02to0.52) | 26.50 (4.24to83.17) | 0.21 (0.15to0.27) | | 0.00 (0.00to0.01) | 1.46 (0.23to4.40) | | 0.01 (0.00to0.02) | 1.63 (0.28to4.81) | 0.46 (0.40to0.53) |
| Guyana | 0.34 (0.05to1.01) | 104.66 (15.68to295.80) | | 0.39 (0.05to1.08) | 77.98 (10.61to202.19) | -0.94 (-1.11to-0.77) | | 0.02 (0.00to0.05) | 6.81 (0.94to17.99) | | 0.02 (0.00to0.06) | 5.47 (0.77to13.48) | -0.78 (-0.98to-0.59) |
| Haiti | 1.75 (0.20to5.08) | 72.00 (8.60to193.89) | | 3.53 (0.43to10.03) | 66.23 (8.68to178.85) | -0.12 (-0.23to-0.01) | | 0.09 (0.01to0.26) | 5.08 (0.63to13.24) | | 0.20 (0.03to0.54) | 4.83 (0.63to12.31) | -0.04 (-0.12to0.05) |
| Honduras | 0.21 (0.03to0.86) | 10.65 (1.57to43.15) | | 0.81 (0.11to3.20) | 15.84 (2.17to58.77) | 1.38 (1.15to1.61) | | 0.01 (0.00to0.04) | 0.67 (0.10to2.51) | | 0.05 (0.01to0.18) | 1.15 (0.15to3.98) | 1.82 (1.48to2.16) |
| Hungary | 7.11 (1.44to21.33) | 51.80 (10.49to154.40) | | 5.12 (1.02to14.34) | 25.18 (4.89to72.88) | -2.88 (-3.07to-2.68) | | 0.43 (0.09to1.22) | 3.44 (0.74to9.61) | | 0.36 (0.08to0.97) | 1.70 (0.36to4.56) | -2.91 (-3.16to-2.65) |
| Iceland | 0.09 (0.01to0.25) | 31.13 (4.53to84.50) | | 0.09 (0.01to0.23) | 13.96 (2.01to37.17) | -3.25 (-3.45to-3.06) | | 0.01 (0.00to0.02) | 2.33 (0.35to6.01) | | 0.01 (0.00to0.02) | 1.03 (0.15to2.56) | -3.40 (-3.66to-3.13) |
| India | 79.84 (13.69to231.40) | 26.83 (5.12to71.78) | | 181.24 (37.45to505.68) | 20.00 (4.32to51.58) | -1.05 (-1.29to-0.81) | | 3.95 (0.76to10.96) | 1.92 (0.39to4.98) | | 11.34 (2.49to28.95) | 1.50 (0.35to3.71) | -1.13 (-1.45to-0.81) |
| Indonesia | 32.05 (3.73to100.88) | 42.95 (5.19to130.65) | | 101.22 (12.42to321.54) | 62.03 (7.79to188.27) | 1.38 (1.28to1.47) | | 1.55 (0.19to4.75) | 2.73 (0.36to8.24) | | 5.37 (0.68to16.92) | 4.22 (0.56to12.70) | 1.65 (1.54to1.77) |
| Iran (Islamic Republic of) | 20.29 (3.16to54.83) | 104.40 (19.68to255.85) | | 41.94 (7.58to104.57) | 63.38 (12.02to154.69) | -2.02 (-2.19to-1.86) | | 0.94 (0.18to2.33) | 6.81 (1.36to15.74) | | 2.45 (0.50to5.63) | 4.16 (0.88to9.43) | -2.02 (-2.21to-1.82) |
| Iraq | 10.66 (2.07to24.84) | 154.88 (32.86to349.78) | | 28.82 (5.84to68.98) | 155.48 (34.19to351.11) | 0.09 (-0.03to0.21) | | 0.58 (0.13to1.24) | 9.60 (2.31to19.89) | | 1.51 (0.33to3.25) | 10.03 (2.31to20.59) | 0.26 (0.11to0.41) |
| Ireland | 2.28 (0.38to5.44) | 58.75 (9.77to137.76) | | 1.78 (0.27to3.97) | 22.84 (3.53to51.63) | -3.90 (-4.14to-3.66) | | 0.17 (0.03to0.38) | 4.75 (0.76to10.63) | | 0.15 (0.03to0.33) | 1.90 (0.32to4.11) | -3.68 (-3.90to-3.47) |
| Israel | 1.36 (0.17to3.84) | 29.42 (3.89to82.76) | | 1.83 (0.28to4.73) | 14.44 (2.15to37.96) | -3.00 (-3.16to-2.84) | | 0.09 (0.01to0.24) | 2.16 (0.28to5.75) | | 0.14 (0.02to0.34) | 1.05 (0.15to2.55) | -3.13 (-3.33to-2.92) |
| Italy | 46.79 (6.68to118.92) | 52.92 (7.55to133.15) | | 36.38 (5.23to89.06) | 19.08 (2.85to48.27) | -3.93 (-4.18to-3.68) | | 3.64 (0.52to8.86) | 4.36 (0.63to10.46) | | 3.52 (0.52to8.26) | 1.66 (0.24to3.92) | -3.72 (-3.92to-3.52) |
| Jamaica | 1.14 (0.18to2.74) | 60.71 (9.52to145.37) | | 1.67 (0.26to3.88) | 53.02 (8.32to124.17) | -0.45 (-0.72to-0.18) | | 0.09 (0.01to0.20) | 4.76 (0.69to10.88) | | 0.14 (0.02to0.32) | 4.14 (0.65to9.31) | -0.54 (-0.81to-0.27) |
| Japan | 55.11 (5.83to174.10) | 36.44 (3.94to114.18) | | 64.35 (7.31to192.77) | 13.88 (1.50to42.57) | -3.61 (-3.81to-3.41) | | 3.83 (0.42to11.72) | 2.82 (0.32to8.41) | | 5.04 (0.60to14.79) | 0.82 (0.10to2.44) | -4.58 (-4.82to-4.33) |
| Jordan | 1.16 (0.20to3.07) | 118.85 (23.85to298.39) | | 3.62 (0.66to8.85) | 75.02 (15.32to171.38) | -1.99 (-2.25to-1.72) | | 0.06 (0.01to0.15) | 7.90 (1.70to18.40) | | 0.18 (0.04to0.41) | 4.90 (1.06to10.56) | -2.07 (-2.32to-1.81) |
| Kazakhstan | 7.53 (1.43to21.18) | 69.39 (13.62to187.66) | | 9.61 (1.75to25.86) | 69.39 (13.29to182.23) | -0.64 (-1.00to-0.29) | | 0.47 (0.09to1.23) | 4.87 (1.01to12.59) | | 0.61 (0.12to1.58) | 5.27 (1.12to13.46) | -0.34 (-0.66to-0.01) |
| Kenya | 0.62 (0.12to2.77) | 8.87 (1.86to37.33) | | 1.70 (0.34to7.55) | 9.77 (2.07to38.84) | 0.43 (0.35to0.50) | | 0.03 (0.01to0.11) | 0.53 (0.11to2.01) | | 0.08 (0.02to0.30) | 0.61 (0.13to2.26) | 0.59 (0.51to0.67) |
| Kiribati | 0.02 (0.00to0.06) | 61.51 (8.83to169.35) | | 0.03 (0.00to0.10) | 65.29 (9.63to175.39) | 0.00 (-0.15to0.15) | | 0.00 (0.00to0.00) | 3.26 (0.52to8.53) | | 0.00 (0.00to0.00) | 3.73 (0.64to9.54) | 0.29 (0.15to0.43) |
| Kuwait | 0.36 (0.07to0.84) | 69.72 (15.83to145.98) | | 1.47 (0.28to3.29) | 64.03 (14.50to129.68) | 0.63 (-0.06to1.33) | | 0.02 (0.00to0.03) | 4.29 (1.09to8.17) | | 0.07 (0.02to0.14) | 3.89 (1.01to7.50) | 0.78 (0.02to1.55) |
| Kyrgyzstan | 1.49 (0.28to4.55) | 51.77 (9.83to157.31) | | 1.39 (0.25to4.47) | 35.38 (6.59to106.08) | -1.78 (-2.24to-1.31) | | 0.10 (0.02to0.28) | 3.53 (0.71to9.76) | | 0.08 (0.02to0.23) | 2.39 (0.46to6.61) | -1.76 (-2.33to-1.20) |
| Lao People's Democratic Republic | 0.49 (0.07to1.77) | 31.51 (4.12to107.86) | | 1.12 (0.15to3.75) | 33.44 (4.42to108.25) | -0.06 (-0.21to0.10) | | 0.02 (0.00to0.08) | 1.97 (0.25to6.51) | | 0.06 (0.01to0.19) | 2.24 (0.29to6.68) | 0.20 (0.06to0.34) |
| Latvia | 3.51 (0.76to8.99) | 98.79 (21.72to251.93) | | 3.19 (0.70to7.74) | 67.66 (14.85to166.33) | -1.67 (-1.86to-1.48) | | 0.26 (0.06to0.64) | 7.73 (1.78to18.70) | | 0.27 (0.06to0.63) | 5.50 (1.30to12.91) | -1.51 (-1.69to-1.33) |
| Lebanon | 0.96 (0.18to2.23) | 54.01 (11.27to120.93) | | 2.57 (0.52to5.68) | 49.61 (10.08to109.74) | -0.01 (-0.19to0.18) | | 0.05 (0.01to0.11) | 3.60 (0.82to7.68) | | 0.14 (0.03to0.30) | 2.93 (0.58to6.13) | -0.40 (-0.56to-0.24) |
| Lesotho | 0.18 (0.03to0.61) | 22.82 (3.62to72.85) | | 0.33 (0.05to1.00) | 34.71 (5.82to101.69) | 1.91 (1.70to2.13) | | 0.01 (0.00to0.03) | 1.53 (0.26to4.61) | | 0.02 (0.00to0.05) | 2.42 (0.44to6.82) | 2.11 (1.86to2.36) |
| Liberia | 0.23 (0.03to0.69) | 24.66 (3.67to73.00) | | 0.38 (0.05to1.15) | 22.10 (3.55to65.24) | -0.25 (-0.38to-0.12) | | 0.01 (0.00to0.03) | 1.54 (0.25to4.23) | | 0.02 (0.00to0.05) | 1.43 (0.25to4.05) | -0.11 (-0.22to-0.00) |
| Libya | 1.22 (0.24to2.86) | 74.63 (16.11to169.73) | | 4.24 (0.78to10.39) | 89.11 (17.69to202.67) | 0.96 (0.80to1.12) | | 0.07 (0.02to0.14) | 4.66 (1.06to9.76) | | 0.21 (0.05to0.46) | 5.07 (1.16to10.93) | 0.70 (0.51to0.89) |
| Lithuania | 2.53 (0.51to6.41) | 55.35 (11.15to140.45) | | 3.38 (0.76to8.05) | 49.84 (11.01to121.61) | -0.53 (-0.74to-0.31) | | 0.17 (0.04to0.41) | 3.75 (0.86to9.11) | | 0.27 (0.06to0.62) | 3.79 (0.89to8.78) | -0.10 (-0.29to0.09) |
| Luxembourg | 0.29 (0.04to0.88) | 54.00 (6.74to160.50) | | 0.16 (0.02to0.45) | 14.41 (1.92to40.64) | -4.82 (-5.09to-4.54) | | 0.02 (0.00to0.07) | 4.41 (0.53to12.68) | | 0.01 (0.00to0.04) | 1.15 (0.16to3.02) | -4.87 (-5.12to-4.61) |
| Madagascar | 0.65 (0.12to2.76) | 14.50 (2.77to56.16) | | 1.45 (0.26to6.40) | 17.16 (3.28to66.58) | 0.48 (0.33to0.62) | | 0.03 (0.01to0.11) | 0.81 (0.15to2.84) | | 0.06 (0.01to0.23) | 1.02 (0.19to3.62) | 0.67 (0.49to0.85) |
| Malawi | 0.45 (0.08to1.82) | 15.07 (2.81to54.68) | | 0.92 (0.17to3.55) | 15.79 (2.98to55.02) | 0.26 (0.18to0.34) | | 0.02 (0.00to0.07) | 0.93 (0.17to3.22) | | 0.05 (0.01to0.16) | 1.03 (0.19to3.40) | 0.39 (0.30to0.47) |
| Malaysia | 3.77 (0.47to10.92) | 47.25 (6.45to132.85) | | 9.30 (1.25to26.04) | 40.74 (5.80to112.58) | -0.39 (-0.57to-0.20) | | 0.18 (0.03to0.51) | 2.69 (0.40to7.27) | | 0.45 (0.07to1.23) | 2.41 (0.37to6.21) | -0.43 (-0.66to-0.20) |
| Maldives | 0.03 (0.00to0.08) | 39.71 (4.76to114.96) | | 0.07 (0.01to0.19) | 25.13 (3.38to69.66) | -2.06 (-2.24to-1.88) | | 0.00 (0.00to0.00) | 2.51 (0.34to6.97) | | 0.00 (0.00to0.01) | 1.64 (0.23to4.42) | -1.93 (-2.10to-1.76) |
| Mali | 0.56 (0.08to1.93) | 17.04 (2.70to54.10) | | 1.07 (0.16to3.58) | 15.56 (2.31to51.21) | -0.38 (-0.45to-0.30) | | 0.03 (0.00to0.08) | 1.09 (0.18to3.28) | | 0.06 (0.01to0.17) | 1.05 (0.17to3.18) | -0.17 (-0.23to-0.10) |
| Malta | 0.28 (0.04to0.63) | 68.89 (11.75to153.95) | | 0.30 (0.05to0.62) | 29.91 (5.47to62.30) | -3.21 (-3.36to-3.05) | | 0.02 (0.00to0.04) | 5.10 (0.96to10.65) | | 0.02 (0.00to0.05) | 2.30 (0.45to4.56) | -3.01 (-3.17to-2.86) |
| Marshall Islands | 0.01 (0.00to0.02) | 55.42 (9.06to144.68) | | 0.01 (0.00to0.04) | 57.20 (8.70to154.41) | 0.01 (-0.09to0.12) | | 0.00 (0.00to0.00) | 3.30 (0.57to8.32) | | 0.00 (0.00to0.00) | 3.33 (0.59to8.32) | -0.01 (-0.08to0.05) |
| Mauritania | 0.57 (0.09to1.45) | 63.08 (11.23to156.03) | | 0.83 (0.14to2.12) | 44.50 (8.38to109.30) | -1.36 (-1.49to-1.24) | | 0.03 (0.00to0.06) | 3.54 (0.68to8.31) | | 0.04 (0.01to0.10) | 2.67 (0.53to6.10) | -1.10 (-1.21to-0.98) |
| Mauritius | 0.42 (0.05to1.30) | 68.45 (8.68to205.22) | | 0.49 (0.07to1.39) | 31.64 (4.48to88.34) | -3.63 (-4.06to-3.21) | | 0.02 (0.00to0.06) | 4.23 (0.61to12.23) | | 0.03 (0.00to0.08) | 2.05 (0.31to5.53) | -3.49 (-3.91to-3.06) |
| Mexico | 7.50 (0.76to24.57) | 19.48 (2.09to63.01) | | 13.15 (1.47to42.04) | 12.12 (1.39to38.14) | -1.99 (-2.25to-1.73) | | 0.40 (0.05to1.29) | 1.29 (0.15to4.19) | | 0.80 (0.09to2.46) | 0.80 (0.09to2.43) | -2.23 (-2.54to-1.92) |
| Micronesia (Federated States of) | 0.02 (0.00to0.06) | 58.19 (9.33to148.49) | | 0.03 (0.00to0.09) | 61.90 (10.92to157.77) | 0.04 (-0.06to0.14) | | 0.00 (0.00to0.00) | 3.39 (0.59to8.32) | | 0.00 (0.00to0.00) | 3.75 (0.69to8.90) | 0.19 (0.11to0.26) |
| Monaco | 0.04 (0.01to0.11) | 51.76 (7.61to133.68) | | 0.03 (0.00to0.07) | 26.45 (3.82to66.12) | -2.49 (-2.81to-2.17) | | 0.00 (0.00to0.01) | 4.37 (0.65to10.61) | | 0.00 (0.00to0.01) | 2.04 (0.30to4.80) | -2.83 (-3.18to-2.48) |
| Mongolia | 0.09 (0.02to0.33) | 9.74 (1.76to31.89) | | 0.29 (0.05to1.05) | 15.19 (2.80to47.76) | 1.66 (1.18to2.13) | | 0.00 (0.00to0.01) | 0.50 (0.09to1.41) | | 0.01 (0.00to0.04) | 0.96 (0.18to2.65) | 2.63 (2.02to3.24) |
| Montenegro | 0.10 (0.02to0.30) | 18.71 (3.59to52.23) | | 0.23 (0.04to0.65) | 25.54 (4.73to69.55) | 1.74 (1.46to2.03) | | 0.01 (0.00to0.02) | 1.30 (0.26to3.55) | | 0.02 (0.00to0.04) | 1.92 (0.39to5.11) | 2.13 (1.78to2.48) |
| Morocco | 10.71 (2.12to25.48) | 97.63 (20.24to218.63) | | 33.24 (6.44to77.41) | 126.47 (25.16to277.94) | 1.04 (0.93to1.14) | | 0.57 (0.12to1.24) | 6.41 (1.40to13.68) | | 1.82 (0.39to3.82) | 8.22 (1.83to16.74) | 0.96 (0.78to1.13) |
| Mozambique | 0.73 (0.14to3.16) | 15.11 (2.99to60.87) | | 1.87 (0.36to7.80) | 20.55 (4.05to79.06) | 1.51 (1.36to1.67) | | 0.03 (0.01to0.13) | 0.89 (0.18to3.30) | | 0.09 (0.02to0.33) | 1.20 (0.24to4.29) | 1.42 (1.29to1.56) |
| Myanmar | 8.39 (0.98to29.21) | 46.43 (5.52to144.07) | | 16.80 (2.11to52.60) | 45.26 (5.78to136.89) | -0.15 (-0.20to-0.11) | | 0.44 (0.05to1.35) | 3.11 (0.37to9.49) | | 1.06 (0.14to3.24) | 3.30 (0.42to10.16) | 0.15 (0.09to0.21) |
| Namibia | 0.33 (0.05to0.92) | 54.09 (8.67to150.28) | | 0.63 (0.10to1.65) | 52.86 (8.29to136.66) | -0.34 (-0.57to-0.10) | | 0.02 (0.00to0.05) | 3.46 (0.57to9.36) | | 0.04 (0.01to0.10) | 3.57 (0.63to9.37) | -0.14 (-0.38to0.10) |
| Nauru | 0.00 (0.00to0.01) | 67.16 (9.84to189.63) | | 0.00 (0.00to0.01) | 73.08 (11.35to204.00) | 0.09 (-0.12to0.29) | | 0.00 (0.00to0.00) | 3.95 (0.62to10.68) | | 0.00 (0.00to0.00) | 4.17 (0.70to10.80) | 0.05 (-0.15to0.24) |
| Nepal | 1.49 (0.25to4.87) | 22.57 (3.88to65.10) | | 3.80 (0.67to10.91) | 21.82 (4.12to59.31) | -0.22 (-0.45to0.01) | | 0.08 (0.01to0.24) | 1.62 (0.29to4.49) | | 0.24 (0.05to0.67) | 1.66 (0.31to4.48) | -0.03 (-0.25to0.20) |
| Netherlands | 3.95 (0.43to12.60) | 19.23 (2.10to61.13) | | 3.95 (0.42to12.46) | 10.65 (1.13to33.47) | -2.65 (-3.04to-2.26) | | 0.30 (0.03to1.01) | 1.51 (0.16to5.05) | | 0.33 (0.03to1.09) | 0.84 (0.08to2.74) | -2.56 (-2.95to-2.18) |
| New Zealand | 1.15 (0.13to3.48) | 30.41 (3.50to91.69) | | 1.53 (0.21to4.06) | 17.82 (2.49to47.51) | -2.02 (-2.17to-1.86) | | 0.08 (0.01to0.25) | 2.39 (0.27to6.92) | | 0.14 (0.02to0.35) | 1.47 (0.21to3.73) | -1.92 (-2.07to-1.77) |
| Nicaragua | 0.13 (0.02to0.53) | 9.91 (1.51to37.44) | | 0.34 (0.05to1.33) | 9.67 (1.27to36.38) | -0.68 (-1.09to-0.27) | | 0.01 (0.00to0.03) | 0.65 (0.09to2.48) | | 0.02 (0.00to0.08) | 0.73 (0.09to2.49) | -0.32 (-0.87to0.24) |
| Niger | 0.47 (0.06to1.58) | 21.53 (3.17to65.17) | | 1.14 (0.16to3.74) | 19.35 (2.90to57.28) | -0.44 (-0.53to-0.35) | | 0.02 (0.00to0.06) | 1.34 (0.21to3.96) | | 0.05 (0.01to0.15) | 1.27 (0.20to3.61) | -0.20 (-0.26to-0.14) |
| Nigeria | 8.73 (1.25to27.26) | 24.29 (3.69to73.04) | | 14.96 (2.11to45.93) | 21.19 (3.36to62.13) | -0.70 (-0.86to-0.55) | | 0.45 (0.07to1.36) | 1.56 (0.26to4.58) | | 0.75 (0.12to2.20) | 1.37 (0.23to3.97) | -0.70 (-0.87to-0.53) |
| Niue | 0.00 (0.00to0.00) | 49.21 (7.63to131.89) | | 0.00 (0.00to0.00) | 46.44 (8.17to122.10) | -0.43 (-0.52to-0.34) | | 0.00 (0.00to0.00) | 3.02 (0.53to7.61) | | 0.00 (0.00to0.00) | 2.67 (0.45to6.76) | -0.68 (-0.78to-0.58) |
| North Macedonia | 1.33 (0.26to3.99) | 90.64 (17.67to256.93) | | 2.44 (0.47to6.80) | 96.71 (19.14to265.70) | 0.16 (-0.11to0.44) | | 0.09 (0.02to0.25) | 6.99 (1.39to18.77) | | 0.17 (0.03to0.47) | 8.13 (1.65to21.95) | 0.59 (0.25to0.92) |
| Northern Mariana Islands | 0.01 (0.00to0.02) | 52.53 (8.48to138.02) | | 0.01 (0.00to0.04) | 37.30 (6.23to95.68) | -1.72 (-2.00to-1.44) | | 0.00 (0.00to0.00) | 3.21 (0.55to8.11) | | 0.00 (0.00to0.00) | 2.09 (0.34to5.19) | -2.10 (-2.44to-1.77) |
| Norway | 3.10 (0.41to8.79) | 40.95 (5.30to117.05) | | 1.94 (0.28to5.22) | 17.71 (2.61to49.16) | -3.13 (-3.22to-3.04) | | 0.24 (0.03to0.67) | 3.14 (0.41to8.59) | | 0.15 (0.02to0.39) | 1.23 (0.17to3.19) | -3.61 (-3.74to-3.48) |
| Oman | 0.53 (0.09to1.43) | 108.02 (20.83to263.64) | | 1.28 (0.23to3.03) | 113.26 (24.78to235.79) | 0.78 (0.54to1.03) | | 0.02 (0.00to0.06) | 6.71 (1.43to14.95) | | 0.06 (0.01to0.12) | 7.72 (1.72to15.33) | 1.19 (0.84to1.53) |
| Pakistan | 19.01 (3.30to50.76) | 39.12 (6.98to102.14) | | 35.36 (6.10to96.87) | 42.09 (7.98to110.07) | -0.01 (-0.21to0.19) | | 1.15 (0.22to2.98) | 2.73 (0.54to6.97) | | 1.89 (0.38to4.90) | 2.89 (0.62to7.15) | -0.03 (-0.21to0.14) |
| Palau | 0.00 (0.00to0.01) | 50.67 (7.29to139.18) | | 0.01 (0.00to0.03) | 55.46 (9.15to148.00) | 0.24 (0.17to0.30) | | 0.00 (0.00to0.00) | 2.79 (0.47to7.38) | | 0.00 (0.00to0.00) | 2.98 (0.52to7.48) | 0.14 (0.08to0.20) |
| Palestine | 1.02 (0.21to2.40) | 140.22 (28.75to319.57) | | 2.15 (0.43to5.15) | 122.92 (26.53to276.52) | -0.22 (-0.75to0.32) | | 0.06 (0.01to0.14) | 9.72 (2.11to21.14) | | 0.12 (0.03to0.27) | 8.72 (1.99to18.19) | -0.14 (-0.69to0.41) |
| Panama | 0.14 (0.02to0.51) | 9.85 (1.52to35.97) | | 0.30 (0.04to1.11) | 7.17 (0.92to26.44) | -1.42 (-1.59to-1.26) | | 0.01 (0.00to0.03) | 0.71 (0.10to2.63) | | 0.02 (0.00to0.08) | 0.55 (0.07to1.91) | -1.28 (-1.48to-1.09) |
| Papua New Guinea | 0.30 (0.03to0.94) | 22.32 (3.10to63.61) | | 0.92 (0.10to2.70) | 25.80 (3.75to70.30) | 0.52 (0.45to0.59) | | 0.01 (0.00to0.03) | 1.19 (0.17to3.35) | | 0.03 (0.00to0.10) | 1.45 (0.23to3.91) | 0.72 (0.65to0.80) |
| Paraguay | 0.44 (0.05to1.44) | 22.35 (2.40to72.91) | | 0.98 (0.10to3.20) | 19.46 (1.98to63.17) | -0.52 (-0.71to-0.33) | | 0.03 (0.00to0.10) | 1.69 (0.17to5.53) | | 0.07 (0.01to0.23) | 1.42 (0.14to4.66) | -0.58 (-0.81to-0.36) |
| Peru | 1.34 (0.15to4.43) | 12.46 (1.40to40.24) | | 2.63 (0.28to8.11) | 8.04 (0.86to25.08) | -1.56 (-1.83to-1.28) | | 0.09 (0.01to0.27) | 0.88 (0.09to2.80) | | 0.19 (0.02to0.58) | 0.56 (0.06to1.73) | -1.51 (-1.76to-1.25) |
| Philippines | 2.66 (0.43to10.73) | 12.11 (1.95to42.72) | | 9.68 (1.56to38.45) | 14.69 (2.38to56.02) | 1.17 (0.80to1.53) | | 0.12 (0.02to0.44) | 0.83 (0.13to2.83) | | 0.45 (0.07to1.64) | 0.86 (0.14to2.95) | 0.67 (0.25to1.09) |
| Poland | 21.26 (3.98to57.67) | 52.09 (10.09to139.40) | | 22.40 (4.62to58.92) | 30.17 (6.07to79.77) | -2.36 (-2.58to-2.13) | | 1.31 (0.27to3.45) | 3.53 (0.75to9.14) | | 1.67 (0.35to4.24) | 2.15 (0.45to5.46) | -2.26 (-2.56to-1.97) |
| Portugal | 15.02 (1.98to39.84) | 118.27 (15.89to307.19) | | 10.13 (1.44to24.36) | 33.41 (4.61to81.95) | -5.17 (-5.62to-4.72) | | 1.13 (0.16to2.93) | 9.90 (1.35to24.94) | | 0.93 (0.14to2.18) | 2.86 (0.42to6.71) | -5.11 (-5.57to-4.64) |
| Puerto Rico | 0.94 (0.16to2.23) | 27.73 (4.68to65.54) | | 1.18 (0.18to2.81) | 14.59 (2.18to35.76) | -2.54 (-2.80to-2.27) | | 0.07 (0.01to0.16) | 2.20 (0.35to4.98) | | 0.09 (0.01to0.22) | 0.98 (0.15to2.30) | -3.38 (-3.69to-3.07) |
| Qatar | 0.06 (0.01to0.17) | 79.35 (17.56to170.62) | | 0.31 (0.04to0.83) | 57.81 (13.46to118.27) | -1.14 (-1.36to-0.91) | | 0.00 (0.00to0.00) | 5.35 (1.29to11.10) | | 0.01 (0.00to0.01) | 4.23 (1.05to8.28) | -0.77 (-1.05to-0.48) |
| Republic of Korea | 15.95 (1.64to52.03) | 73.80 (8.02to230.06) | | 18.27 (1.99to55.15) | 21.79 (2.37to65.18) | -4.86 (-5.13to-4.59) | | 0.83 (0.09to2.60) | 5.01 (0.56to15.32) | | 1.19 (0.13to3.44) | 1.51 (0.17to4.34) | -4.83 (-5.15to-4.51) |
| Republic of Moldova | 1.45 (0.28to4.25) | 42.78 (8.58to117.12) | | 2.00 (0.41to5.64) | 34.29 (6.95to96.84) | -0.36 (-0.74to0.03) | | 0.10 (0.02to0.25) | 3.37 (0.67to8.87) | | 0.14 (0.03to0.37) | 2.43 (0.52to6.30) | -0.81 (-1.24to-0.38) |
| Romania | 17.41 (3.45to47.89) | 74.44 (15.10to199.07) | | 23.58 (5.30to59.89) | 57.22 (12.66to147.72) | -1.33 (-1.51to-1.14) | | 1.09 (0.23to2.86) | 5.57 (1.21to14.25) | | 1.84 (0.43to4.52) | 4.40 (1.03to10.76) | -1.17 (-1.34to-0.99) |
| Russian Federation | 131.70 (25.74to391.98) | 84.28 (16.73to237.90) | | 142.81 (29.29to385.15) | 59.07 (12.28to160.49) | -1.60 (-2.14to-1.06) | | 8.74 (1.78to23.62) | 6.39 (1.33to16.90) | | 11.25 (2.44to29.52) | 4.71 (1.04to12.36) | -1.43 (-2.02to-0.84) |
| Rwanda | 0.32 (0.06to1.33) | 13.81 (2.60to53.84) | | 0.47 (0.09to1.85) | 10.50 (2.04to37.64) | -1.40 (-1.59to-1.21) | | 0.01 (0.00to0.05) | 0.80 (0.15to2.99) | | 0.02 (0.00to0.08) | 0.70 (0.13to2.37) | -0.81 (-0.95to-0.68) |
| Saint Kitts and Nevis | 0.05 (0.01to0.12) | 128.34 (18.89to328.86) | | 0.04 (0.01to0.10) | 73.13 (11.63to182.82) | -1.70 (-1.92to-1.49) | | 0.00 (0.00to0.01) | 9.77 (1.40to23.75) | | 0.00 (0.00to0.01) | 5.75 (0.87to13.46) | -1.39 (-1.61to-1.16) |
| Saint Lucia | 0.06 (0.01to0.16) | 81.67 (12.60to208.35) | | 0.10 (0.01to0.24) | 48.17 (7.63to117.70) | -2.35 (-2.76to-1.95) | | 0.00 (0.00to0.01) | 6.45 (0.89to15.95) | | 0.01 (0.00to0.02) | 3.84 (0.56to8.91) | -2.46 (-2.87to-2.05) |
| Saint Vincent and the Grenadines | 0.05 (0.01to0.13) | 71.12 (9.69to190.41) | | 0.06 (0.01to0.15) | 48.92 (6.76to122.50) | -0.83 (-1.28to-0.39) | | 0.00 (0.00to0.01) | 5.23 (0.73to13.26) | | 0.00 (0.00to0.01) | 3.73 (0.54to8.71) | -0.65 (-1.11to-0.20) |
| Samoa | 0.04 (0.01to0.10) | 51.09 (9.12to133.43) | | 0.06 (0.01to0.17) | 48.86 (8.46to127.12) | -0.31 (-0.38to-0.24) | | 0.00 (0.00to0.00) | 3.03 (0.53to7.33) | | 0.00 (0.00to0.01) | 2.82 (0.52to7.01) | -0.40 (-0.46to-0.34) |
| San Marino | 0.01 (0.00to0.03) | 32.60 (4.63to87.51) | | 0.02 (0.00to0.05) | 21.78 (2.86to58.30) | -1.33 (-1.42to-1.24) | | 0.00 (0.00to0.00) | 2.65 (0.37to6.63) | | 0.00 (0.00to0.00) | 1.73 (0.24to4.49) | -1.40 (-1.50to-1.30) |
| Sao Tome and Principe | 0.01 (0.00to0.04) | 24.71 (4.02to73.86) | | 0.03 (0.00to0.08) | 29.36 (4.67to88.38) | 0.48 (0.40to0.56) | | 0.00 (0.00to0.00) | 1.51 (0.27to4.46) | | 0.00 (0.00to0.00) | 1.83 (0.31to5.38) | 0.64 (0.59to0.70) |
| Saudi Arabia | 7.44 (1.49to16.21) | 156.32 (36.39to316.30) | | 26.11 (5.18to52.78) | 159.92 (40.88to298.44) | 0.49 (0.28to0.70) | | 0.37 (0.09to0.75) | 10.20 (2.57to19.55) | | 0.88 (0.22to1.69) | 9.20 (2.59to16.49) | -0.29 (-0.41to-0.16) |
| Senegal | 0.78 (0.10to2.41) | 27.85 (4.11to81.54) | | 1.58 (0.23to4.77) | 24.56 (3.89to70.25) | -0.56 (-0.63to-0.48) | | 0.04 (0.01to0.10) | 1.65 (0.27to4.66) | | 0.08 (0.01to0.23) | 1.55 (0.27to4.41) | -0.33 (-0.41to-0.24) |
| Serbia | 7.65 (1.46to22.30) | 80.16 (16.05to225.48) | | 10.33 (1.99to27.88) | 67.92 (13.02to185.26) | -0.77 (-0.97to-0.57) | | 0.52 (0.10to1.41) | 5.89 (1.21to15.85) | | 0.76 (0.15to2.05) | 5.58 (1.13to14.97) | -0.30 (-0.48to-0.13) |
| Seychelles | 0.02 (0.00to0.06) | 34.37 (4.42to103.52) | | 0.03 (0.00to0.08) | 26.60 (3.34to79.45) | -0.77 (-0.98to-0.56) | | 0.00 (0.00to0.00) | 2.10 (0.27to6.17) | | 0.00 (0.00to0.00) | 1.63 (0.22to4.82) | -0.65 (-0.92to-0.38) |
| Sierra Leone | 0.42 (0.06to1.32) | 24.68 (3.51to75.82) | | 0.75 (0.11to2.41) | 23.99 (3.74to71.54) | 0.04 (-0.02to0.10) | | 0.02 (0.00to0.06) | 1.49 (0.23to4.38) | | 0.04 (0.01to0.11) | 1.49 (0.25to4.24) | 0.16 (0.07to0.24) |
| Singapore | 0.78 (0.08to2.55) | 43.22 (4.65to138.31) | | 0.90 (0.10to2.73) | 12.59 (1.40to37.66) | -4.57 (-4.68to-4.45) | | 0.04 (0.00to0.12) | 2.64 (0.30to7.97) | | 0.05 (0.01to0.14) | 0.72 (0.08to1.99) | -4.69 (-4.84to-4.54) |
| Slovakia | 2.45 (0.47to7.09) | 42.99 (8.39to121.88) | | 2.64 (0.50to7.36) | 29.31 (5.49to81.50) | -1.36 (-1.53to-1.20) | | 0.15 (0.03to0.40) | 2.80 (0.56to7.53) | | 0.17 (0.03to0.47) | 1.96 (0.39to5.26) | -1.22 (-1.37to-1.07) |
| Slovenia | 1.02 (0.19to2.92) | 43.18 (8.26to122.75) | | 0.82 (0.16to2.24) | 16.18 (3.04to44.56) | -3.45 (-3.81to-3.10) | | 0.07 (0.01to0.19) | 3.13 (0.63to8.57) | | 0.07 (0.01to0.18) | 1.24 (0.25to3.32) | -3.27 (-3.59to-2.95) |
| Solomon Islands | 0.05 (0.01to0.16) | 46.59 (6.20to136.62) | | 0.14 (0.02to0.44) | 60.43 (8.65to170.09) | 0.84 (0.76to0.92) | | 0.00 (0.00to0.01) | 2.65 (0.38to7.54) | | 0.01 (0.00to0.02) | 3.74 (0.61to10.10) | 1.14 (1.07to1.22) |
| Somalia | 0.26 (0.05to1.09) | 12.94 (2.44to50.10) | | 0.65 (0.12to2.94) | 13.13 (2.53to51.11) | 0.29 (0.20to0.37) | | 0.01 (0.00to0.04) | 0.73 (0.13to2.70) | | 0.03 (0.01to0.11) | 0.81 (0.15to2.99) | 0.69 (0.58to0.80) |
| South Africa | 7.17 (1.10to19.31) | 37.43 (6.33to99.11) | | 15.65 (2.69to41.73) | 41.70 (7.73to107.19) | 0.92 (0.23to1.60) | | 0.37 (0.07to0.94) | 2.29 (0.43to5.78) | | 0.92 (0.18to2.33) | 2.87 (0.56to7.10) | 1.33 (0.62to2.04) |
| South Sudan | 0.23 (0.04to0.94) | 11.70 (2.24to43.33) | | 0.34 (0.06to1.34) | 11.28 (2.03to41.23) | -0.05 (-0.12to0.02) | | 0.01 (0.00to0.04) | 0.68 (0.13to2.29) | | 0.02 (0.00to0.06) | 0.68 (0.12to2.45) | 0.13 (0.06to0.21) |
| Spain | 24.23 (3.05to68.82) | 45.61 (5.77to128.89) | | 18.34 (2.49to47.28) | 14.70 (1.91to39.81) | -4.20 (-4.45to-3.96) | | 1.89 (0.23to5.18) | 3.76 (0.47to10.18) | | 1.67 (0.22to4.20) | 1.17 (0.16to2.98) | -4.38 (-4.64to-4.13) |
| Sri Lanka | 2.45 (0.32to8.24) | 30.80 (3.88to100.20) | | 4.47 (0.58to14.41) | 21.12 (2.71to67.30) | -0.71 (-0.97to-0.45) | | 0.15 (0.02to0.46) | 2.29 (0.28to7.09) | | 0.27 (0.03to0.85) | 1.51 (0.18to4.66) | -0.66 (-1.00to-0.31) |
| Sudan | 13.97 (3.32to27.27) | 175.07 (45.79to335.74) | | 35.33 (8.05to69.83) | 203.86 (52.24to391.56) | 0.71 (0.57to0.85) | | 0.67 (0.19to1.27) | 10.72 (2.97to19.71) | | 1.63 (0.43to3.09) | 11.69 (3.36to21.25) | 0.41 (0.30to0.52) |
| Suriname | 0.11 (0.02to0.31) | 48.69 (7.19to129.43) | | 0.27 (0.04to0.69) | 48.91 (6.93to125.64) | -0.23 (-0.61to0.16) | | 0.01 (0.00to0.02) | 3.41 (0.48to8.49) | | 0.02 (0.00to0.04) | 3.40 (0.50to8.12) | -0.23 (-0.65to0.19) |
| Sweden | 4.69 (0.54to14.50) | 28.08 (3.19to87.17) | | 3.69 (0.43to11.16) | 15.15 (1.73to45.80) | -2.96 (-3.17to-2.75) | | 0.36 (0.04to1.08) | 2.10 (0.26to6.23) | | 0.32 (0.04to0.90) | 1.11 (0.14to3.17) | -2.98 (-3.18to-2.78) |
| Switzerland | 4.01 (0.55to10.66) | 34.29 (4.87to91.66) | | 2.85 (0.40to7.26) | 13.28 (1.87to34.36) | -3.35 (-3.56to-3.14) | | 0.33 (0.05to0.84) | 2.81 (0.40to7.22) | | 0.26 (0.04to0.63) | 1.05 (0.15to2.59) | -3.49 (-3.68to-3.30) |
| Syrian Arab Republic | 4.69 (0.94to11.59) | 103.88 (21.94to240.24) | | 8.38 (1.55to20.33) | 86.09 (17.45to195.79) | -1.22 (-1.49to-0.95) | | 0.25 (0.05to0.56) | 6.54 (1.45to14.05) | | 0.42 (0.09to0.91) | 5.58 (1.25to11.39) | -1.17 (-1.45to-0.89) |
| Taiwan (Province of China) | 4.13 (0.56to12.54) | 34.92 (4.99to98.66) | | 6.57 (1.02to17.94) | 16.50 (2.53to45.07) | -2.83 (-3.03to-2.63) | | 0.21 (0.03to0.62) | 2.23 (0.33to6.32) | | 0.37 (0.06to1.03) | 0.90 (0.14to2.51) | -3.42 (-3.67to-3.17) |
| Tajikistan | 0.26 (0.05to0.78) | 9.54 (1.83to29.55) | | 0.54 (0.10to1.77) | 17.96 (3.36to53.70) | 2.32 (1.82to2.81) | | 0.02 (0.00to0.05) | 0.63 (0.12to1.75) | | 0.03 (0.01to0.09) | 1.32 (0.25to3.73) | 2.58 (1.89to3.27) |
| Thailand | 5.77 (0.72to19.15) | 19.56 (2.46to61.35) | | 15.25 (1.90to47.52) | 15.52 (1.95to47.67) | -1.23 (-1.37to-1.09) | | 0.27 (0.03to0.82) | 1.11 (0.14to3.42) | | 0.82 (0.11to2.55) | 0.86 (0.11to2.63) | -1.31 (-1.45to-1.16) |
| Timor-Leste | 0.05 (0.01to0.18) | 27.77 (3.56to86.73) | | 0.24 (0.03to0.77) | 37.08 (4.90to121.60) | 1.16 (1.03to1.29) | | 0.00 (0.00to0.01) | 1.83 (0.23to5.67) | | 0.01 (0.00to0.04) | 2.55 (0.33to8.10) | 1.31 (1.18to1.44) |
| Togo | 0.26 (0.03to0.85) | 24.61 (3.64to74.70) | | 0.68 (0.10to2.20) | 23.52 (3.87to69.87) | -0.22 (-0.28to-0.17) | | 0.01 (0.00to0.03) | 1.49 (0.24to4.30) | | 0.03 (0.00to0.09) | 1.47 (0.26to4.17) | -0.09 (-0.14to-0.03) |
| Tokelau | 0.00 (0.00to0.00) | 40.51 (5.84to112.10) | | 0.00 (0.00to0.00) | 42.08 (6.71to111.83) | 0.13 (0.01to0.25) | | 0.00 (0.00to0.00) | 2.41 (0.39to6.46) | | 0.00 (0.00to0.00) | 2.49 (0.44to6.40) | 0.17 (0.05to0.29) |
| Tonga | 0.01 (0.00to0.04) | 29.79 (5.03to81.60) | | 0.03 (0.00to0.07) | 32.99 (5.82to85.63) | 0.27 (0.11to0.43) | | 0.00 (0.00to0.00) | 1.68 (0.30to4.46) | | 0.00 (0.00to0.00) | 1.93 (0.36to4.80) | 0.48 (0.31to0.64) |
| Trinidad and Tobago | 0.61 (0.09to1.57) | 82.23 (13.34to203.63) | | 0.88 (0.13to2.16) | 51.21 (7.78to124.53) | -1.98 (-2.18to-1.78) | | 0.04 (0.01to0.09) | 6.08 (0.91to14.27) | | 0.06 (0.01to0.14) | 3.65 (0.58to8.38) | -2.04 (-2.25to-1.82) |
| Tunisia | 1.30 (0.20to4.00) | 34.12 (5.54to98.03) | | 4.57 (0.67to13.36) | 40.64 (6.08to115.44) | 0.37 (0.06to0.67) | | 0.07 (0.01to0.21) | 2.50 (0.42to7.00) | | 0.29 (0.05to0.80) | 2.83 (0.45to7.70) | -0.00 (-0.45to0.44) |
| Turkey | 13.32 (2.44to33.57) | 43.72 (8.06to106.48) | | 37.92 (7.34to91.64) | 46.37 (9.07to110.26) | 0.86 (0.47to1.26) | | 0.78 (0.15to1.81) | 2.92 (0.57to6.77) | | 2.46 (0.53to5.54) | 3.16 (0.70to7.03) | 1.19 (0.66to1.73) |
| Turkmenistan | 0.57 (0.10to1.91) | 35.40 (6.56to110.45) | | 1.66 (0.27to5.42) | 49.08 (8.48to149.73) | 1.25 (0.89to1.61) | | 0.03 (0.01to0.09) | 2.26 (0.43to6.21) | | 0.09 (0.02to0.25) | 2.97 (0.55to8.20) | 0.96 (0.59to1.32) |
| Tuvalu | 0.00 (0.00to0.01) | 44.55 (6.85to121.93) | | 0.00 (0.00to0.01) | 47.48 (7.24to126.90) | 0.07 (-0.03to0.18) | | 0.00 (0.00to0.00) | 2.63 (0.44to6.69) | | 0.00 (0.00to0.00) | 2.90 (0.50to7.41) | 0.16 (0.03to0.30) |
| Uganda | 0.58 (0.11to2.36) | 10.97 (2.17to41.77) | | 1.27 (0.24to4.98) | 11.47 (2.27to40.88) | -0.08 (-0.32to0.15) | | 0.03 (0.01to0.10) | 0.65 (0.13to2.33) | | 0.06 (0.01to0.21) | 0.72 (0.14to2.45) | 0.16 (-0.09to0.40) |
| Ukraine | 36.32 (6.99to112.32) | 55.22 (10.75to167.55) | | 28.71 (5.50to90.15) | 36.76 (7.13to119.50) | -2.26 (-2.53to-1.99) | | 2.42 (0.47to6.95) | 4.10 (0.80to11.55) | | 1.98 (0.37to5.72) | 2.55 (0.49to7.37) | -2.58 (-2.87to-2.29) |
| United Arab Emirates | 0.72 (0.11to1.72) | 218.60 (49.65to443.68) | | 6.22 (0.78to14.81) | 153.25 (34.19to310.26) | -1.43 (-1.98to-0.88) | | 0.02 (0.00to0.05) | 13.59 (3.47to26.48) | | 0.14 (0.02to0.32) | 8.47 (2.15to16.11) | -1.86 (-2.51to-1.21) |
| United Kingdom | 44.85 (6.62to117.47) | 47.17 (6.87to124.55) | | 29.51 (4.69to72.16) | 20.28 (3.14to50.53) | -3.40 (-3.59to-3.22) | | 3.49 (0.52to8.85) | 3.79 (0.56to9.52) | | 2.64 (0.44to6.10) | 1.68 (0.28to3.91) | -3.36 (-3.57to-3.15) |
| United Republic of Tanzania | 0.95 (0.18to3.96) | 10.59 (2.05to41.93) | | 2.88 (0.55to11.67) | 14.10 (2.72to52.98) | 1.28 (1.13to1.43) | | 0.04 (0.01to0.16) | 0.61 (0.12to2.24) | | 0.15 (0.03to0.52) | 0.88 (0.17to3.01) | 1.66 (1.46to1.87) |
| United States of America | 78.00 (9.35to231.51) | 23.19 (2.74to69.36) | | 71.44 (7.98to223.49) | 12.32 (1.32to39.09) | -2.54 (-2.79to-2.29) | | 4.99 (0.61to14.62) | 1.45 (0.18to4.25) | | 4.34 (0.51to13.58) | 0.65 (0.08to2.03) | -3.33 (-3.71to-2.95) |
| United States Virgin Islands | 0.01 (0.00to0.04) | 22.40 (2.86to64.37) | | 0.03 (0.00to0.10) | 21.61 (3.16to58.86) | -0.06 (-0.26to0.15) | | 0.00 (0.00to0.00) | 1.71 (0.24to4.87) | | 0.00 (0.00to0.01) | 1.68 (0.25to4.46) | 0.02 (-0.18to0.22) |
| Uruguay | 1.17 (0.12to4.06) | 30.87 (3.13to106.85) | | 1.13 (0.11to3.88) | 18.56 (1.73to64.10) | -1.82 (-1.99to-1.65) | | 0.07 (0.01to0.25) | 1.97 (0.20to6.82) | | 0.09 (0.01to0.30) | 1.27 (0.12to4.39) | -1.66 (-1.89to-1.42) |
| Uzbekistan | 3.40 (0.63to11.59) | 32.12 (6.11to102.99) | | 6.48 (1.09to21.55) | 48.52 (8.76to144.36) | 0.86 (0.13to1.59) | | 0.19 (0.04to0.55) | 1.95 (0.38to5.59) | | 0.28 (0.05to0.87) | 3.35 (0.64to9.27) | 1.26 (0.33to2.20) |
| Vanuatu | 0.02 (0.00to0.07) | 36.35 (4.93to113.38) | | 0.06 (0.01to0.18) | 39.64 (5.04to120.64) | 0.08 (-0.03to0.19) | | 0.00 (0.00to0.00) | 2.08 (0.29to6.34) | | 0.00 (0.00to0.01) | 2.31 (0.31to6.65) | 0.17 (0.07to0.26) |
| Venezuela (Bolivarian Republic of) | 0.84 (0.12to3.20) | 9.81 (1.44to34.99) | | 2.18 (0.29to8.02) | 8.30 (1.07to30.09) | -0.74 (-0.96to-0.52) | | 0.05 (0.01to0.17) | 0.66 (0.09to2.39) | | 0.15 (0.02to0.52) | 0.60 (0.07to2.02) | -0.50 (-0.73to-0.27) |
| Viet Nam | 10.37 (1.48to35.82) | 30.00 (4.24to101.24) | | 27.20 (3.69to96.21) | 34.99 (4.69to118.76) | 0.92 (0.74to1.10) | | 0.65 (0.09to2.23) | 2.10 (0.28to6.97) | | 1.75 (0.22to5.81) | 2.48 (0.31to8.23) | 0.98 (0.77to1.20) |
| Yemen | 3.46 (0.65to8.57) | 96.65 (19.69to227.14) | | 12.68 (2.31to32.12) | 117.38 (23.34to274.93) | 0.82 (0.71to0.93) | | 0.17 (0.03to0.40) | 6.46 (1.40to14.38) | | 0.64 (0.13to1.45) | 7.63 (1.65to16.80) | 0.68 (0.59to0.78) |
| Zambia | 0.40 (0.06to1.34) | 18.18 (2.78to59.40) | | 1.38 (0.21to4.48) | 27.34 (4.39to86.85) | 1.45 (1.33to1.56) | | 0.02 (0.00to0.06) | 1.13 (0.18to3.54) | | 0.07 (0.01to0.22) | 1.82 (0.30to5.69) | 1.72 (1.58to1.85) |
| Zimbabwe | 0.53 (0.09to1.96) | 17.09 (3.05to59.61) | | 1.15 (0.19to4.13) | 20.95 (3.64to70.53) | 1.03 (0.81to1.24) | | 0.03 (0.00to0.09) | 1.15 (0.21to3.85) | | 0.06 (0.01to0.19) | 1.37 (0.25to4.31) | 0.94 (0.72to1.17) |

| **Supplemental table 6. DALYs and mortality of** **type 2 diabetes attributable to LPA in 204 countries and territories in 1990 and 2019** | | | | | | | | | | | | | |
| --- | --- | --- | --- | --- | --- | --- | --- | --- | --- | --- | --- | --- | --- |
|  | **DALYs (95% UI)** | | | | | |  | **Mortality (95% UI)** | | | | | |
|  | **1990** | |  | **2019** | | **EAPC** |  | **1990** | |  | **2019** | | **EAPC** |
| **location** | **Number(no.×10^3^)** | **ASR per100,000** |  | **Number(no.×10^3^)** | **ASR per100,000** |  |  | **Number(no.×10^3^)** | **ASR per100,000** |  | **Number(no.×10^3^)** | **ASR per100,000** |  |
| Afghanistan | 8.73 (3.96to16.46) | 122.65 (57.47to225.11) | | 26.16 (11.50to49.55) | 203.61 (97.30to364.02) | 2.08 (1.96to2.20) | | 0.24 (0.11to0.45) | 3.81 (1.85to6.86) | | 0.57 (0.25to1.10) | 5.70 (2.68to10.55) | 1.82 (1.64to2.00) |
| Albania | 0.21 (0.09to0.43) | 10.93 (4.87to21.39) | | 0.69 (0.29to1.37) | 16.37 (6.80to32.53) | 1.21 (1.07to1.36) | | 0.00 (0.00to0.01) | 0.22 (0.11to0.41) | | 0.01 (0.00to0.02) | 0.22 (0.10to0.41) | -0.42 (-0.84to0.00) |
| Algeria | 10.13 (4.88to17.76) | 88.73 (45.21to152.16) | | 47.85 (24.42to81.42) | 142.10 (74.29to238.95) | 1.93 (1.74to2.13) | | 0.22 (0.11to0.37) | 2.72 (1.40to4.51) | | 0.80 (0.43to1.28) | 3.06 (1.66to4.79) | 0.93 (0.64to1.22) |
| American Samoa | 0.07 (0.03to0.12) | 289.78 (140.38to499.61) | | 0.22 (0.10to0.38) | 443.06 (216.74to774.02) | 1.20 (0.80to1.60) | | 0.00 (0.00to0.00) | 9.80 (5.09to15.84) | | 0.01 (0.00to0.01) | 13.07 (7.04to20.97) | 0.79 (0.41to1.17) |
| Andorra | 0.02 (0.01to0.03) | 30.02 (12.91to56.34) | | 0.06 (0.03to0.12) | 44.98 (20.44to83.75) | 1.36 (1.34to1.38) | | 0.00 (0.00to0.00) | 0.91 (0.42to1.61) | | 0.00 (0.00to0.00) | 0.77 (0.38to1.26) | -0.62 (-0.68to-0.55) |
| Angola | 2.72 (1.02to5.67) | 72.06 (29.76to141.93) | | 9.49 (3.65to19.00) | 87.84 (36.23to164.36) | 0.68 (0.64to0.73) | | 0.08 (0.03to0.15) | 2.67 (1.15to5.05) | | 0.25 (0.10to0.47) | 3.24 (1.43to5.86) | 0.62 (0.58to0.65) |
| Antigua and Barbuda | 0.10 (0.05to0.16) | 179.53 (84.91to305.41) | | 0.23 (0.11to0.39) | 230.80 (114.10to381.53) | 0.56 (0.43to0.68) | | 0.00 (0.00to0.01) | 7.09 (3.67to11.30) | | 0.01 (0.00to0.01) | 8.34 (4.55to12.87) | 0.18 (0.00to0.36) |
| Argentina | 2.20 (0.78to5.33) | 7.01 (2.47to17.05) | | 5.25 (2.00to11.72) | 9.69 (3.69to21.67) | 1.81 (1.31to2.30) | | 0.08 (0.03to0.19) | 0.29 (0.09to0.67) | | 0.18 (0.07to0.37) | 0.32 (0.12to0.67) | 0.92 (0.39to1.46) |
| Armenia | 0.59 (0.25to1.16) | 23.33 (10.34to44.54) | | 1.99 (0.89to3.87) | 47.37 (21.22to91.79) | 1.91 (1.42to2.40) | | 0.02 (0.01to0.03) | 0.77 (0.35to1.40) | | 0.07 (0.03to0.13) | 1.67 (0.77to3.08) | 1.86 (1.18to2.54) |
| Australia | 7.31 (3.35to12.71) | 37.94 (17.29to66.05) | | 22.25 (11.01to37.41) | 54.39 (26.28to94.33) | 1.11 (0.91to1.30) | | 0.25 (0.13to0.41) | 1.37 (0.69to2.21) | | 0.62 (0.33to0.96) | 1.30 (0.70to2.03) | -0.49 (-0.84to-0.13) |
| Austria | 4.36 (2.12to7.35) | 35.71 (17.01to61.11) | | 8.64 (4.12to15.04) | 47.83 (22.38to85.47) | 0.76 (0.37to1.15) | | 0.18 (0.09to0.29) | 1.41 (0.72to2.26) | | 0.30 (0.15to0.47) | 1.35 (0.69to2.17) | 0.05 (-0.42to0.51) |
| Azerbaijan | 0.83 (0.35to1.72) | 17.12 (7.34to33.83) | | 3.76 (1.52to7.71) | 42.62 (18.42to80.90) | 3.01 (2.76to3.25) | | 0.02 (0.01to0.04) | 0.45 (0.20to0.87) | | 0.09 (0.04to0.18) | 1.31 (0.59to2.36) | 3.48 (3.08to3.87) |
| Bahamas | 0.27 (0.12to0.47) | 177.21 (84.76to298.68) | | 0.74 (0.34to1.30) | 189.83 (92.50to325.69) | -0.07 (-0.20to0.06) | | 0.01 (0.00to0.01) | 6.37 (3.40to10.15) | | 0.02 (0.01to0.03) | 5.37 (2.92to8.54) | -1.05 (-1.26to-0.85) |
| Bahrain | 0.53 (0.27to0.85) | 318.02 (174.25to487.63) | | 4.94 (2.56to8.00) | 528.03 (291.48to807.84) | 1.88 (1.53to2.23) | | 0.01 (0.01to0.02) | 12.85 (7.37to19.14) | | 0.11 (0.06to0.18) | 22.13 (12.41to32.91) | 2.28 (1.77to2.80) |
| Bangladesh | 18.32 (8.23to33.88) | 46.30 (21.33to82.47) | | 62.34 (28.86to116.18) | 54.16 (25.43to96.25) | 0.12 (-0.43to0.68) | | 0.76 (0.37to1.32) | 2.33 (1.14to3.92) | | 2.67 (1.27to4.55) | 2.79 (1.33to4.70) | 0.10 (-0.63to0.84) |
| Barbados | 0.71 (0.38to1.12) | 241.45 (123.82to387.89) | | 1.25 (0.69to2.00) | 258.29 (138.28to423.76) | -0.12 (-0.25to0.02) | | 0.03 (0.02to0.05) | 9.95 (5.49to15.21) | | 0.05 (0.03to0.07) | 9.57 (5.38to14.52) | -0.54 (-0.74to-0.33) |
| Belarus | 1.45 (0.65to2.78) | 11.34 (5.13to21.76) | | 1.89 (0.85to3.71) | 11.64 (5.13to22.86) | -0.37 (-0.54to-0.20) | | 0.03 (0.01to0.05) | 0.22 (0.10to0.38) | | 0.02 (0.01to0.04) | 0.15 (0.07to0.26) | -2.40 (-2.77to-2.03) |
| Belgium | 6.90 (3.38to11.92) | 44.80 (21.52to78.54) | | 11.72 (5.70to21.16) | 53.51 (24.41to99.65) | -0.03 (-0.40to0.34) | | 0.24 (0.12to0.37) | 1.51 (0.79to2.37) | | 0.25 (0.13to0.39) | 0.88 (0.46to1.35) | -2.28 (-2.54to-2.02) |
| Belize | 0.12 (0.06to0.21) | 135.76 (64.21to228.03) | | 0.64 (0.30to1.10) | 228.38 (112.93to387.16) | 1.84 (1.28to2.41) | | 0.00 (0.00to0.01) | 5.46 (2.90to8.78) | | 0.02 (0.01to0.03) | 7.81 (4.22to12.21) | 1.39 (0.73to2.05) |
| Benin | 0.77 (0.31to1.50) | 41.17 (17.00to78.64) | | 2.96 (1.20to5.92) | 66.19 (28.13to125.63) | 1.68 (1.44to1.92) | | 0.03 (0.01to0.05) | 1.67 (0.72to3.11) | | 0.09 (0.04to0.18) | 2.51 (1.11to4.72) | 1.49 (1.19to1.78) |
| Bermuda | 0.07 (0.04to0.12) | 118.50 (59.51to193.46) | | 0.13 (0.07to0.22) | 104.31 (52.30to178.52) | -0.70 (-0.81to-0.60) | | 0.00 (0.00to0.00) | 4.46 (2.42to6.83) | | 0.00 (0.00to0.01) | 2.58 (1.39to4.08) | -2.16 (-2.26to-2.07) |
| Bhutan | 0.11 (0.05to0.21) | 53.59 (25.25to94.46) | | 0.47 (0.23to0.83) | 91.40 (44.60to156.55) | 2.20 (2.10to2.30) | | 0.00 (0.00to0.01) | 2.10 (1.02to3.64) | | 0.02 (0.01to0.03) | 3.82 (1.98to6.34) | 2.36 (2.29to2.43) |
| Bolivia (Plurinational State of) | 1.23 (0.40to2.70) | 40.78 (14.13to85.37) | | 5.75 (2.13to11.49) | 68.55 (26.11to135.12) | 1.69 (1.64to1.74) | | 0.04 (0.02to0.09) | 1.62 (0.57to3.31) | | 0.21 (0.08to0.40) | 2.75 (1.08to5.20) | 1.74 (1.68to1.80) |
| Bosnia and Herzegovina | 0.90 (0.37to1.77) | 23.44 (10.04to45.30) | | 3.84 (1.67to7.25) | 64.16 (28.20to120.34) | 4.01 (3.40to4.62) | | 0.02 (0.01to0.04) | 0.65 (0.29to1.19) | | 0.13 (0.06to0.24) | 2.26 (0.97to4.09) | 5.36 (4.53to6.19) |
| Botswana | 0.50 (0.20to1.03) | 93.93 (38.71to182.87) | | 2.19 (0.89to4.28) | 172.92 (74.10to323.06) | 2.03 (1.62to2.45) | | 0.02 (0.01to0.04) | 4.12 (1.75to7.78) | | 0.08 (0.03to0.15) | 7.48 (3.18to13.92) | 1.90 (1.46to2.35) |
| Brazil | 171.05 (93.46to262.00) | 184.79 (103.77to278.81) | | 424.72 (246.90to644.02) | 178.27 (103.40to269.11) | -0.02 (-0.07to0.04) | | 4.31 (2.50to6.26) | 5.54 (3.29to8.00) | | 11.32 (6.78to16.19) | 5.00 (2.98to7.13) | -0.28 (-0.35to-0.22) |
| Brunei Darussalam | 0.23 (0.08to0.45) | 258.60 (103.76to488.98) | | 0.60 (0.22to1.18) | 214.50 (87.55to393.78) | -0.28 (-0.51to-0.04) | | 0.01 (0.00to0.01) | 11.15 (4.89to19.77) | | 0.02 (0.01to0.03) | 8.05 (3.70to13.74) | -0.62 (-0.91to-0.32) |
| Bulgaria | 3.46 (1.54to6.60) | 28.12 (12.45to53.58) | | 4.84 (2.23to8.95) | 33.43 (15.24to62.65) | 0.23 (-0.03to0.50) | | 0.09 (0.04to0.17) | 0.79 (0.38to1.42) | | 0.13 (0.06to0.23) | 0.81 (0.38to1.46) | -0.60 (-0.95to-0.24) |
| Burkina Faso | 1.01 (0.40to2.14) | 26.10 (10.83to54.00) | | 2.72 (1.05to5.73) | 33.33 (13.49to66.57) | 0.50 (0.25to0.75) | | 0.04 (0.01to0.07) | 1.16 (0.49to2.31) | | 0.09 (0.03to0.18) | 1.34 (0.54to2.57) | 0.18 (-0.02to0.38) |
| Burundi | 0.46 (0.17to1.05) | 21.53 (8.01to48.64) | | 0.75 (0.27to1.74) | 19.23 (7.03to41.46) | -0.59 (-0.68to-0.51) | | 0.02 (0.01to0.04) | 0.98 (0.37to2.07) | | 0.03 (0.01to0.06) | 0.85 (0.31to1.80) | -0.66 (-0.76to-0.57) |
| Cabo Verde | 0.05 (0.02to0.10) | 20.35 (8.31to40.94) | | 0.25 (0.11to0.49) | 61.38 (26.41to116.94) | 3.40 (2.94to3.86) | | 0.00 (0.00to0.00) | 0.49 (0.21to0.93) | | 0.01 (0.00to0.02) | 2.27 (1.01to4.16) | 4.42 (3.66to5.20) |
| Cambodia | 1.39 (0.51to2.91) | 35.36 (13.57to71.40) | | 4.94 (1.84to10.05) | 47.20 (18.41to93.72) | 0.96 (0.82to1.11) | | 0.05 (0.02to0.11) | 1.58 (0.63to3.12) | | 0.16 (0.06to0.31) | 1.73 (0.70to3.44) | 0.27 (0.18to0.36) |
| Cameroon | 2.27 (0.93to4.49) | 55.89 (24.01to107.36) | | 10.12 (3.99to19.82) | 92.29 (38.80to173.42) | 1.68 (1.33to2.03) | | 0.08 (0.04to0.16) | 2.48 (1.06to4.64) | | 0.35 (0.15to0.65) | 3.90 (1.77to7.07) | 1.55 (1.24to1.86) |
| Canada | 8.88 (3.86to15.96) | 27.37 (11.92to49.13) | | 24.79 (11.16to46.18) | 35.76 (15.93to67.71) | 0.29 (-0.02to0.61) | | 0.39 (0.18to0.67) | 1.24 (0.58to2.13) | | 0.81 (0.39to1.34) | 1.03 (0.50to1.72) | -1.47 (-2.11to-0.82) |
| Central African Republic | 1.01 (0.38to2.08) | 87.24 (35.27to171.60) | | 2.16 (0.82to4.55) | 100.84 (41.52to198.60) | 0.58 (0.45to0.71) | | 0.03 (0.01to0.06) | 3.23 (1.36to6.21) | | 0.06 (0.02to0.11) | 3.54 (1.58to6.69) | 0.43 (0.27to0.58) |
| Chad | 0.78 (0.31to1.61) | 29.72 (11.68to60.05) | | 2.30 (0.88to4.90) | 45.34 (17.81to93.27) | 1.66 (1.50to1.82) | | 0.03 (0.01to0.06) | 1.18 (0.48to2.33) | | 0.07 (0.03to0.15) | 1.75 (0.69to3.48) | 1.47 (1.25to1.69) |
| Chile | 3.05 (1.12to6.25) | 31.00 (11.72to63.31) | | 12.71 (5.25to23.57) | 52.83 (21.79to97.99) | 1.85 (1.56to2.14) | | 0.08 (0.03to0.16) | 0.90 (0.36to1.75) | | 0.31 (0.14to0.57) | 1.30 (0.58to2.38) | 1.15 (0.83to1.47) |
| China | 180.71 (78.76to335.26) | 23.64 (10.72to41.97) | | 452.07 (192.47to861.49) | 23.61 (10.34to44.17) | -0.42 (-0.58to-0.26) | | 4.23 (1.96to7.64) | 0.68 (0.32to1.18) | | 11.86 (5.47to20.89) | 0.72 (0.33to1.25) | -0.06 (-0.24to0.12) |
| Colombia | 6.90 (2.31to15.02) | 40.66 (13.83to86.41) | | 25.04 (9.03to51.77) | 47.17 (16.72to98.31) | -0.04 (-0.31to0.24) | | 0.16 (0.06to0.33) | 1.09 (0.40to2.19) | | 0.49 (0.19to0.98) | 0.90 (0.34to1.79) | -1.61 (-2.04to-1.18) |
| Comoros | 0.02 (0.01to0.06) | 11.89 (4.58to29.50) | | 0.06 (0.02to0.14) | 13.13 (5.15to30.60) | 0.12 (-0.01to0.25) | | 0.00 (0.00to0.00) | 0.58 (0.23to1.37) | | 0.00 (0.00to0.01) | 0.63 (0.24to1.37) | 0.02 (-0.10to0.14) |
| Congo | 1.17 (0.44to2.36) | 112.43 (45.45to217.73) | | 3.23 (1.28to6.28) | 124.84 (51.72to228.00) | 0.37 (0.28to0.46) | | 0.04 (0.01to0.07) | 4.43 (1.85to8.41) | | 0.09 (0.04to0.17) | 4.73 (2.17to8.40) | 0.21 (0.13to0.29) |
| Cook Islands | 0.04 (0.02to0.06) | 289.43 (133.50to503.96) | | 0.09 (0.04to0.15) | 368.02 (181.01to635.89) | 0.62 (0.40to0.84) | | 0.00 (0.00to0.00) | 12.71 (6.26to21.50) | | 0.00 (0.00to0.01) | 13.91 (7.26to22.40) | 0.07 (-0.18to0.33) |
| Costa Rica | 0.32 (0.11to0.76) | 18.64 (6.38to43.49) | | 1.26 (0.43to2.81) | 24.74 (8.34to55.78) | 0.47 (0.23to0.71) | | 0.01 (0.00to0.02) | 0.47 (0.16to1.03) | | 0.02 (0.01to0.05) | 0.49 (0.16to1.04) | -0.76 (-1.25to-0.28) |
| Côte d'Ivoire | 1.82 (0.68to3.67) | 51.04 (20.44to99.71) | | 6.91 (2.68to13.96) | 72.23 (30.02to138.46) | 1.29 (1.02to1.56) | | 0.06 (0.02to0.11) | 2.15 (0.88to4.05) | | 0.21 (0.09to0.40) | 2.85 (1.20to5.40) | 0.91 (0.59to1.22) |
| Croatia | 1.55 (0.69to2.97) | 24.99 (11.14to47.53) | | 3.12 (1.40to5.86) | 34.96 (15.74to67.41) | 0.95 (0.85to1.06) | | 0.04 (0.02to0.07) | 0.67 (0.32to1.19) | | 0.08 (0.04to0.14) | 0.81 (0.38to1.42) | 0.25 (-0.08to0.58) |
| Cuba | 10.96 (5.19to19.11) | 107.14 (50.45to186.86) | | 20.22 (9.75to35.49) | 109.24 (52.74to195.23) | -0.08 (-0.47to0.31) | | 0.28 (0.15to0.43) | 2.76 (1.48to4.35) | | 0.29 (0.16to0.45) | 1.46 (0.78to2.28) | -2.43 (-3.18to-1.68) |
| Cyprus | 0.92 (0.38to1.65) | 126.55 (52.64to222.76) | | 1.50 (0.65to2.70) | 78.94 (34.54to140.99) | -2.10 (-2.26to-1.95) | | 0.05 (0.02to0.08) | 7.64 (3.36to12.93) | | 0.06 (0.03to0.10) | 3.48 (1.62to5.63) | -3.04 (-3.19to-2.90) |
| Czechia | 4.69 (2.11to9.00) | 34.21 (15.23to65.33) | | 13.50 (6.27to24.89) | 65.49 (30.10to123.06) | 2.90 (2.52to3.28) | | 0.11 (0.05to0.19) | 0.79 (0.38to1.40) | | 0.29 (0.14to0.50) | 1.31 (0.62to2.20) | 3.77 (2.90to4.65) |
| Democratic People's Republic of Korea | 3.36 (1.33to7.00) | 24.44 (10.19to47.88) | | 8.66 (3.51to16.87) | 28.12 (11.62to54.68) | 0.51 (0.43to0.59) | | 0.10 (0.04to0.21) | 0.88 (0.38to1.66) | | 0.24 (0.10to0.44) | 0.82 (0.36to1.49) | -0.15 (-0.31to0.01) |
| Democratic Republic of the Congo | 11.10 (4.31to23.28) | 74.95 (30.53to146.39) | | 28.09 (10.84to57.75) | 80.40 (33.39to158.36) | 0.16 (0.05to0.27) | | 0.32 (0.13to0.65) | 2.99 (1.29to5.60) | | 0.72 (0.30to1.38) | 2.76 (1.22to4.96) | -0.40 (-0.48to-0.31) |
| Denmark | 2.08 (0.92to3.78) | 26.03 (11.18to48.67) | | 4.54 (2.06to7.99) | 40.62 (17.86to74.11) | 1.31 (0.85to1.77) | | 0.08 (0.04to0.14) | 0.95 (0.44to1.60) | | 0.17 (0.08to0.27) | 1.29 (0.62to2.12) | 1.14 (0.58to1.71) |
| Djibouti | 0.02 (0.01to0.04) | 16.83 (6.18to36.93) | | 0.12 (0.04to0.26) | 24.05 (9.32to50.35) | 1.32 (1.24to1.40) | | 0.00 (0.00to0.00) | 0.78 (0.29to1.64) | | 0.00 (0.00to0.01) | 1.11 (0.44to2.29) | 1.34 (1.26to1.41) |
| Dominica | 0.14 (0.07to0.24) | 198.54 (92.87to337.00) | | 0.22 (0.11to0.38) | 247.33 (123.03to423.31) | 0.50 (0.38to0.61) | | 0.01 (0.00to0.01) | 8.23 (4.21to13.31) | | 0.01 (0.00to0.01) | 8.43 (4.58to13.50) | -0.15 (-0.30to0.00) |
| Dominican Republic | 3.27 (1.62to5.44) | 85.75 (43.94to141.07) | | 15.16 (7.78to25.50) | 158.91 (82.98to264.20) | 2.62 (2.44to2.81) | | 0.09 (0.05to0.15) | 2.86 (1.53to4.55) | | 0.41 (0.22to0.67) | 4.61 (2.46to7.53) | 2.29 (2.08to2.49) |
| Ecuador | 1.80 (0.65to3.68) | 35.11 (13.19to70.01) | | 10.76 (4.31to21.07) | 73.25 (29.94to141.23) | 2.48 (2.28to2.67) | | 0.06 (0.02to0.11) | 1.19 (0.49to2.30) | | 0.36 (0.15to0.68) | 2.69 (1.16to4.98) | 2.80 (2.52to3.08) |
| Egypt | 29.01 (14.60to47.40) | 99.37 (52.29to157.42) | | 120.10 (61.55to201.90) | 181.76 (96.47to296.55) | 2.29 (2.21to2.36) | | 0.85 (0.46to1.33) | 3.49 (1.94to5.42) | | 2.90 (1.51to4.83) | 5.34 (2.90to8.62) | 1.74 (1.63to1.85) |
| El Salvador | 0.44 (0.15to1.06) | 15.27 (5.32to35.92) | | 2.33 (0.75to5.36) | 39.07 (12.50to89.76) | 3.30 (3.00to3.60) | | 0.01 (0.00to0.03) | 0.44 (0.15to1.00) | | 0.08 (0.03to0.18) | 1.29 (0.42to2.80) | 3.80 (3.41to4.20) |
| Equatorial Guinea | 0.15 (0.06to0.30) | 76.95 (31.18to152.49) | | 0.65 (0.26to1.25) | 136.68 (59.58to256.27) | 2.43 (2.28to2.58) | | 0.00 (0.00to0.01) | 2.83 (1.18to5.46) | | 0.02 (0.01to0.04) | 5.47 (2.48to9.84) | 2.75 (2.60to2.89) |
| Eritrea | 0.18 (0.06to0.45) | 20.83 (7.81to49.94) | | 0.58 (0.21to1.35) | 26.15 (9.71to56.61) | 0.95 (0.84to1.06) | | 0.01 (0.00to0.01) | 0.89 (0.33to2.02) | | 0.02 (0.01to0.04) | 1.19 (0.45to2.50) | 1.07 (0.91to1.23) |
| Estonia | 0.20 (0.09to0.38) | 9.84 (4.40to18.94) | | 0.48 (0.21to0.91) | 17.47 (7.60to33.91) | 2.51 (2.22to2.81) | | 0.00 (0.00to0.01) | 0.16 (0.08to0.28) | | 0.01 (0.00to0.02) | 0.28 (0.13to0.49) | 2.34 (1.52to3.18) |
| Eswatini | 0.37 (0.15to0.72) | 136.92 (55.66to261.80) | | 1.47 (0.60to2.78) | 264.62 (110.58to494.25) | 2.40 (1.71to3.09) | | 0.01 (0.01to0.03) | 6.07 (2.59to11.51) | | 0.05 (0.02to0.10) | 11.73 (5.18to21.82) | 2.43 (1.73to3.13) |
| Ethiopia | 5.27 (1.83to12.49) | 28.03 (10.17to62.19) | | 6.72 (2.48to14.84) | 18.19 (6.88to38.55) | -1.81 (-1.89to-1.73) | | 0.18 (0.06to0.40) | 1.19 (0.45to2.67) | | 0.26 (0.10to0.54) | 0.86 (0.33to1.69) | -1.40 (-1.47to-1.33) |
| Fiji | 1.27 (0.53to2.48) | 358.58 (160.82to664.66) | | 4.94 (2.21to9.14) | 672.73 (317.76to1188.43) | 1.74 (1.25to2.23) | | 0.04 (0.02to0.08) | 15.11 (7.27to27.26) | | 0.17 (0.08to0.30) | 29.02 (14.77to49.57) | 1.73 (1.20to2.27) |
| Finland | 2.38 (1.04to4.49) | 33.63 (14.68to64.19) | | 5.15 (2.22to9.73) | 46.26 (18.65to91.61) | 0.85 (0.50to1.20) | | 0.05 (0.03to0.09) | 0.73 (0.36to1.21) | | 0.06 (0.03to0.09) | 0.40 (0.19to0.65) | -2.67 (-2.98to-2.35) |
| France | 24.59 (12.65to40.08) | 29.33 (14.94to48.36) | | 50.84 (26.70to82.04) | 37.94 (19.15to63.80) | 0.63 (0.31to0.95) | | 1.04 (0.57to1.56) | 1.16 (0.63to1.75) | | 1.99 (1.09to3.00) | 1.10 (0.60to1.67) | -0.32 (-0.87to0.24) |
| Gabon | 0.48 (0.19to0.97) | 90.69 (37.57to178.68) | | 1.29 (0.52to2.58) | 128.71 (55.48to245.45) | 1.19 (0.94to1.43) | | 0.02 (0.01to0.03) | 3.80 (1.61to7.26) | | 0.04 (0.02to0.08) | 5.31 (2.39to9.84) | 1.11 (0.84to1.38) |
| Gambia | 0.11 (0.04to0.22) | 34.95 (13.89to69.11) | | 0.53 (0.21to1.06) | 59.97 (24.64to116.79) | 1.75 (1.56to1.94) | | 0.00 (0.00to0.01) | 1.48 (0.59to2.85) | | 0.02 (0.01to0.04) | 2.50 (1.05to4.81) | 1.68 (1.46to1.91) |
| Georgia | 1.05 (0.44to2.03) | 17.34 (7.48to33.54) | | 2.56 (1.13to5.02) | 41.86 (18.27to82.43) | 4.08 (3.65to4.51) | | 0.03 (0.01to0.05) | 0.44 (0.21to0.81) | | 0.07 (0.03to0.13) | 1.10 (0.50to2.04) | 4.51 (3.89to5.14) |
| Germany | 54.26 (22.33to101.93) | 41.49 (16.87to78.67) | | 92.23 (39.10to169.87) | 48.16 (19.34to92.71) | -0.55 (-1.16to0.08) | | 2.04 (0.92to3.52) | 1.51 (0.69to2.63) | | 2.57 (1.20to4.32) | 1.08 (0.50to1.82) | -1.89 (-2.22to-1.57) |
| Ghana | 2.37 (0.93to4.88) | 42.31 (17.13to83.80) | | 11.57 (4.71to23.18) | 77.51 (32.78to152.84) | 2.22 (1.87to2.58) | | 0.08 (0.03to0.16) | 1.73 (0.72to3.36) | | 0.37 (0.16to0.72) | 3.00 (1.33to5.62) | 1.94 (1.61to2.28) |
| Greece | 3.20 (1.22to6.28) | 20.90 (7.99to40.90) | | 6.92 (2.68to13.56) | 29.14 (10.52to59.63) | 0.74 (0.12to1.36) | | 0.09 (0.04to0.17) | 0.63 (0.27to1.13) | | 0.13 (0.06to0.23) | 0.44 (0.19to0.77) | -0.85 (-1.45to-0.25) |
| Greenland | 0.01 (0.00to0.02) | 28.50 (11.34to54.15) | | 0.02 (0.01to0.04) | 32.46 (12.99to62.63) | -0.04 (-0.25to0.16) | | 0.00 (0.00to0.00) | 1.29 (0.55to2.33) | | 0.00 (0.00to0.00) | 0.78 (0.34to1.40) | -2.44 (-2.74to-2.14) |
| Grenada | 0.15 (0.07to0.24) | 202.21 (93.07to349.45) | | 0.28 (0.13to0.49) | 261.55 (129.24to438.67) | 0.88 (0.73to1.03) | | 0.01 (0.00to0.01) | 8.06 (4.15to13.14) | | 0.01 (0.00to0.01) | 9.62 (5.10to15.28) | 0.64 (0.38to0.91) |
| Guam | 0.07 (0.03to0.13) | 101.71 (46.63to177.42) | | 0.17 (0.08to0.32) | 93.87 (42.58to173.06) | -0.15 (-0.45to0.15) | | 0.00 (0.00to0.00) | 4.19 (2.03to7.03) | | 0.00 (0.00to0.01) | 2.29 (1.14to3.91) | -2.29 (-2.69to-1.89) |
| Guatemala | 0.17 (0.08to0.51) | 4.95 (2.11to14.13) | | 1.80 (0.75to5.08) | 16.61 (6.78to47.15) | 3.70 (3.14to4.27) | | 0.00 (0.00to0.01) | 0.14 (0.05to0.36) | | 0.06 (0.02to0.16) | 0.63 (0.23to1.61) | 4.31 (3.49to5.13) |
| Guinea | 1.03 (0.43to2.04) | 33.17 (14.09to65.21) | | 2.56 (1.01to5.12) | 50.29 (20.28to99.57) | 1.49 (1.33to1.65) | | 0.04 (0.02to0.08) | 1.44 (0.61to2.72) | | 0.09 (0.04to0.18) | 2.10 (0.85to3.90) | 1.40 (1.19to1.61) |
| Guinea-Bissau | 0.22 (0.08to0.47) | 57.94 (22.43to118.73) | | 0.51 (0.19to1.04) | 77.31 (31.01to151.31) | 0.94 (0.72to1.15) | | 0.01 (0.00to0.02) | 2.37 (0.96to4.72) | | 0.02 (0.01to0.03) | 3.16 (1.31to6.09) | 0.93 (0.69to1.17) |
| Guyana | 0.97 (0.42to1.73) | 255.85 (117.17to443.11) | | 2.30 (1.05to4.14) | 363.37 (175.04to633.95) | 0.94 (0.52to1.36) | | 0.03 (0.01to0.05) | 9.38 (4.63to15.45) | | 0.07 (0.03to0.12) | 12.41 (6.46to20.63) | 0.61 (0.13to1.09) |
| Haiti | 7.34 (3.09to13.76) | 229.12 (104.03to413.47) | | 15.40 (6.49to30.51) | 224.86 (103.50to426.97) | 0.06 (-0.03to0.14) | | 0.24 (0.11to0.44) | 8.96 (4.28to15.58) | | 0.43 (0.20to0.82) | 7.58 (3.54to13.72) | -0.45 (-0.52to-0.38) |
| Honduras | 0.28 (0.09to0.72) | 13.75 (4.62to34.17) | | 1.40 (0.46to3.39) | 24.01 (7.99to57.85) | 1.87 (1.75to1.99) | | 0.00 (0.00to0.01) | 0.25 (0.08to0.60) | | 0.03 (0.01to0.07) | 0.53 (0.18to1.27) | 2.62 (2.31to2.93) |
| Hungary | 3.26 (1.44to6.28) | 22.35 (9.97to42.76) | | 6.02 (2.69to11.36) | 31.12 (13.72to60.19) | 1.42 (1.18to1.67) | | 0.08 (0.04to0.15) | 0.58 (0.28to1.04) | | 0.14 (0.07to0.25) | 0.67 (0.31to1.20) | 0.83 (0.43to1.23) |
| Iceland | 0.07 (0.03to0.13) | 26.01 (11.94to47.28) | | 0.23 (0.11to0.42) | 44.05 (19.74to81.44) | 1.75 (1.71to1.78) | | 0.00 (0.00to0.00) | 0.62 (0.30to0.98) | | 0.00 (0.00to0.01) | 0.53 (0.27to0.85) | -0.87 (-1.05to-0.69) |
| India | 194.04 (86.51to355.79) | 49.58 (23.67to88.87) | | 576.28 (277.33to1049.49) | 56.71 (28.13to100.42) | 1.14 (0.89to1.40) | | 5.24 (2.48to9.42) | 1.88 (0.93to3.24) | | 19.07 (9.59to32.29) | 2.25 (1.15to3.75) | 0.99 (0.64to1.34) |
| Indonesia | 55.57 (20.33to115.44) | 58.15 (22.94to116.32) | | 216.49 (83.93to420.31) | 100.56 (41.09to187.02) | 1.87 (1.76to1.98) | | 1.77 (0.71to3.49) | 2.22 (0.95to4.23) | | 6.97 (2.93to13.19) | 3.88 (1.70to7.03) | 2.01 (1.90to2.13) |
| Iran (Islamic Republic of) | 16.20 (7.64to28.26) | 64.68 (32.89to108.84) | | 89.98 (43.93to151.68) | 123.28 (61.48to202.37) | 2.51 (2.37to2.65) | | 0.31 (0.16to0.51) | 1.78 (0.96to2.79) | | 2.00 (1.08to3.07) | 3.17 (1.73to4.84) | 2.27 (2.03to2.51) |
| Iraq | 17.47 (8.89to28.73) | 220.56 (117.34to355.85) | | 58.59 (30.15to95.91) | 248.36 (136.67to392.43) | 0.36 (0.29to0.43) | | 0.54 (0.28to0.83) | 7.67 (4.16to11.80) | | 1.42 (0.79to2.24) | 7.56 (4.35to11.44) | -0.11 (-0.23to0.02) |
| Ireland | 1.52 (0.81to2.42) | 37.55 (20.03to59.82) | | 4.12 (2.10to6.91) | 56.95 (29.00to96.70) | 1.16 (0.96to1.36) | | 0.06 (0.03to0.09) | 1.48 (0.83to2.21) | | 0.08 (0.04to0.12) | 0.99 (0.57to1.50) | -1.62 (-1.82to-1.42) |
| Israel | 2.72 (1.27to4.63) | 56.64 (26.11to96.65) | | 9.40 (4.65to15.91) | 80.70 (39.31to139.04) | 0.42 (-0.28to1.13) | | 0.10 (0.05to0.16) | 2.16 (1.09to3.50) | | 0.37 (0.19to0.58) | 2.90 (1.49to4.54) | -0.13 (-1.14to0.89) |
| Italy | 64.87 (32.97to105.27) | 73.15 (36.82to120.42) | | 95.33 (46.94to160.68) | 67.81 (32.24to117.96) | -0.42 (-0.75to-0.08) | | 2.42 (1.28to3.71) | 2.67 (1.43to4.09) | | 3.09 (1.61to4.79) | 1.65 (0.86to2.59) | -1.82 (-1.96to-1.68) |
| Jamaica | 3.85 (1.98to6.10) | 217.22 (109.60to347.28) | | 9.41 (4.96to15.48) | 314.11 (165.99to516.42) | 0.99 (0.76to1.22) | | 0.16 (0.08to0.23) | 8.53 (4.64to12.96) | | 0.37 (0.20to0.58) | 11.71 (6.39to18.30) | 0.86 (0.58to1.14) |
| Japan | 47.06 (18.57to90.51) | 28.02 (11.02to53.82) | | 81.58 (35.36to151.00) | 26.02 (9.60to52.09) | -0.49 (-0.72to-0.25) | | 1.01 (0.46to1.73) | 0.64 (0.29to1.08) | | 1.02 (0.47to1.73) | 0.21 (0.10to0.36) | -3.80 (-4.26to-3.33) |
| Jordan | 2.17 (1.04to3.83) | 183.29 (93.19to308.42) | | 10.36 (4.97to17.79) | 168.04 (85.76to274.71) | -0.56 (-0.91to-0.21) | | 0.08 (0.04to0.13) | 8.35 (4.45to13.51) | | 0.27 (0.15to0.44) | 6.02 (3.28to9.36) | -1.39 (-1.80to-0.97) |
| Kazakhstan | 2.93 (1.26to5.68) | 23.98 (10.74to45.59) | | 8.84 (3.94to16.98) | 53.25 (24.31to99.47) | 2.16 (1.81to2.52) | | 0.05 (0.02to0.08) | 0.41 (0.21to0.73) | | 0.18 (0.09to0.31) | 1.23 (0.60to2.10) | 2.41 (1.59to3.23) |
| Kenya | 0.83 (0.31to1.93) | 11.36 (4.38to25.74) | | 2.89 (1.07to6.66) | 14.74 (5.51to32.45) | 1.06 (0.98to1.13) | | 0.03 (0.01to0.07) | 0.53 (0.21to1.12) | | 0.10 (0.04to0.22) | 0.65 (0.25to1.38) | 0.80 (0.73to0.86) |
| Kiribati | 0.14 (0.06to0.28) | 361.33 (158.25to677.28) | | 0.37 (0.15to0.71) | 513.97 (223.43to939.41) | 0.76 (0.26to1.26) | | 0.00 (0.00to0.01) | 13.57 (6.32to24.12) | | 0.01 (0.00to0.02) | 19.90 (9.34to34.70) | 0.86 (0.35to1.37) |
| Kuwait | 1.22 (0.64to1.99) | 180.64 (100.96to278.13) | | 6.36 (3.24to10.61) | 200.74 (107.55to323.04) | 0.17 (-0.26to0.60) | | 0.02 (0.01to0.03) | 5.14 (3.06to7.43) | | 0.07 (0.04to0.10) | 3.40 (1.96to5.01) | -1.64 (-2.31to-0.97) |
| Kyrgyzstan | 0.34 (0.14to0.68) | 11.31 (4.88to22.54) | | 0.73 (0.30to1.47) | 16.50 (7.04to32.07) | 0.66 (0.42to0.90) | | 0.01 (0.00to0.01) | 0.24 (0.11to0.45) | | 0.01 (0.01to0.02) | 0.30 (0.14to0.57) | -0.18 (-0.56to0.20) |
| Lao People's Democratic Republic | 0.76 (0.27to1.69) | 41.11 (15.80to87.08) | | 2.03 (0.76to4.18) | 53.94 (21.30to109.45) | 0.56 (0.36to0.76) | | 0.03 (0.01to0.06) | 1.66 (0.63to3.42) | | 0.06 (0.02to0.12) | 1.97 (0.81to3.78) | 0.19 (0.01to0.37) |
| Latvia | 0.63 (0.30to1.17) | 17.73 (8.28to32.96) | | 1.35 (0.66to2.38) | 33.39 (15.78to60.60) | 2.54 (2.33to2.75) | | 0.01 (0.01to0.02) | 0.34 (0.16to0.58) | | 0.03 (0.02to0.06) | 0.74 (0.37to1.24) | 3.42 (2.97to3.87) |
| Lebanon | 2.51 (1.26to4.23) | 112.53 (58.19to185.23) | | 7.97 (4.13to13.24) | 153.06 (79.29to253.77) | 1.17 (0.97to1.36) | | 0.06 (0.03to0.09) | 3.15 (1.72to4.90) | | 0.14 (0.07to0.21) | 2.65 (1.43to4.17) | -0.40 (-0.65to-0.14) |
| Lesotho | 0.55 (0.22to1.11) | 58.85 (23.47to116.50) | | 1.75 (0.68to3.52) | 145.54 (59.57to286.48) | 3.77 (3.47to4.08) | | 0.02 (0.01to0.04) | 2.55 (1.06to5.04) | | 0.07 (0.03to0.13) | 6.57 (2.77to12.64) | 3.95 (3.61to4.30) |
| Liberia | 0.59 (0.24to1.14) | 55.93 (24.33to106.38) | | 1.51 (0.61to2.99) | 80.19 (33.76to152.54) | 1.47 (1.32to1.62) | | 0.02 (0.01to0.04) | 2.16 (0.98to3.93) | | 0.04 (0.02to0.08) | 2.88 (1.27to5.37) | 1.15 (1.00to1.30) |
| Libya | 1.90 (0.93to3.20) | 100.51 (51.32to165.88) | | 9.43 (4.71to15.88) | 175.87 (90.65to292.19) | 2.19 (2.06to2.32) | | 0.04 (0.02to0.06) | 2.25 (1.20to3.57) | | 0.13 (0.07to0.22) | 3.00 (1.57to4.91) | 1.53 (1.29to1.78) |
| Lithuania | 0.75 (0.35to1.42) | 16.56 (7.65to31.35) | | 1.34 (0.64to2.43) | 23.37 (10.94to43.87) | 1.34 (1.18to1.50) | | 0.01 (0.01to0.02) | 0.28 (0.14to0.48) | | 0.02 (0.01to0.04) | 0.34 (0.17to0.59) | 0.62 (0.44to0.80) |
| Luxembourg | 0.16 (0.07to0.28) | 28.83 (13.07to51.93) | | 0.54 (0.23to1.02) | 54.43 (22.53to104.98) | 2.05 (1.69to2.42) | | 0.01 (0.00to0.01) | 1.21 (0.60to2.00) | | 0.01 (0.00to0.01) | 0.73 (0.36to1.20) | -1.87 (-2.01to-1.72) |
| Madagascar | 0.71 (0.27to1.58) | 15.45 (5.98to33.23) | | 1.59 (0.60to3.81) | 17.33 (6.61to38.35) | 0.38 (0.30to0.46) | | 0.03 (0.01to0.06) | 0.70 (0.28to1.49) | | 0.05 (0.02to0.12) | 0.74 (0.28to1.61) | 0.14 (0.02to0.25) |
| Malawi | 0.86 (0.33to1.87) | 25.27 (10.07to53.01) | | 1.76 (0.70to3.80) | 27.43 (11.17to55.56) | 0.66 (0.52to0.81) | | 0.03 (0.01to0.07) | 1.14 (0.46to2.31) | | 0.06 (0.02to0.13) | 1.16 (0.48to2.38) | 0.28 (0.16to0.40) |
| Malaysia | 7.28 (3.16to13.20) | 82.86 (37.86to146.63) | | 21.97 (9.96to40.36) | 83.48 (38.80to149.07) | -0.04 (-0.26to0.19) | | 0.21 (0.10to0.36) | 2.64 (1.27to4.46) | | 0.36 (0.18to0.64) | 1.57 (0.79to2.73) | -2.66 (-3.19to-2.12) |
| Maldives | 0.07 (0.03to0.13) | 81.27 (36.16to148.43) | | 0.21 (0.09to0.39) | 73.36 (34.12to128.75) | -0.71 (-0.99to-0.44) | | 0.00 (0.00to0.00) | 3.13 (1.46to5.42) | | 0.01 (0.00to0.01) | 2.16 (1.06to3.60) | -1.74 (-2.04to-1.43) |
| Mali | 1.15 (0.44to2.37) | 30.95 (12.63to61.46) | | 3.36 (1.29to6.93) | 43.64 (17.79to87.55) | 1.12 (0.94to1.29) | | 0.04 (0.02to0.08) | 1.33 (0.56to2.60) | | 0.12 (0.05to0.23) | 1.78 (0.75to3.39) | 0.93 (0.70to1.16) |
| Malta | 0.48 (0.26to0.72) | 112.64 (62.29to171.35) | | 0.92 (0.50to1.45) | 107.34 (57.52to172.73) | -0.13 (-0.24to-0.02) | | 0.02 (0.01to0.02) | 3.95 (2.35to5.70) | | 0.02 (0.01to0.03) | 2.09 (1.19to3.03) | -2.13 (-2.35to-1.91) |
| Marshall Islands | 0.05 (0.02to0.09) | 273.27 (122.29to492.72) | | 0.17 (0.07to0.32) | 447.29 (199.88to821.43) | 1.44 (1.10to1.78) | | 0.00 (0.00to0.00) | 9.33 (4.53to15.79) | | 0.00 (0.00to0.01) | 13.85 (6.59to23.75) | 1.15 (0.84to1.47) |
| Mauritania | 1.20 (0.63to1.97) | 121.59 (64.37to198.32) | | 2.61 (1.39to4.24) | 128.09 (69.31to204.49) | -0.04 (-0.16to0.08) | | 0.04 (0.02to0.07) | 4.91 (2.65to7.79) | | 0.09 (0.05to0.14) | 5.11 (2.76to8.05) | 0.00 (-0.14to0.14) |
| Mauritius | 0.84 (0.35to1.57) | 114.88 (49.82to213.87) | | 5.06 (2.27to9.08) | 287.49 (131.64to508.23) | 4.72 (3.93to5.51) | | 0.03 (0.01to0.05) | 3.79 (1.74to6.68) | | 0.18 (0.09to0.32) | 11.17 (5.38to19.03) | 5.79 (4.75to6.84) |
| Mexico | 63.80 (24.27to122.96) | 145.98 (57.22to276.86) | | 179.60 (69.68to344.58) | 153.39 (60.18to291.79) | 0.20 (-0.04to0.45) | | 1.84 (0.75to3.43) | 4.78 (1.97to8.80) | | 5.59 (2.31to10.30) | 5.14 (2.16to9.39) | -0.02 (-0.27to0.24) |
| Micronesia (Federated States of) | 0.13 (0.06to0.23) | 273.01 (124.65to481.89) | | 0.39 (0.17to0.74) | 533.72 (247.39to970.94) | 2.12 (1.58to2.66) | | 0.00 (0.00to0.01) | 10.73 (5.29to18.14) | | 0.01 (0.01to0.02) | 20.86 (9.91to36.20) | 2.11 (1.63to2.59) |
| Monaco | 0.02 (0.01to0.03) | 24.59 (11.20to44.65) | | 0.04 (0.02to0.07) | 45.02 (20.72to83.24) | 2.11 (2.08to2.13) | | 0.00 (0.00to0.00) | 0.42 (0.22to0.69) | | 0.00 (0.00to0.00) | 0.45 (0.24to0.73) | 0.42 (0.25to0.59) |
| Mongolia | 0.08 (0.03to0.15) | 7.89 (3.33to15.57) | | 0.24 (0.09to0.51) | 11.41 (4.80to21.93) | 1.29 (1.23to1.36) | | 0.00 (0.00to0.00) | 0.20 (0.09to0.38) | | 0.00 (0.00to0.01) | 0.25 (0.12to0.47) | 0.37 (0.12to0.62) |
| Montenegro | 0.19 (0.08to0.36) | 30.57 (13.67to58.53) | | 0.45 (0.20to0.86) | 45.96 (20.41to87.82) | 1.46 (1.42to1.50) | | 0.00 (0.00to0.01) | 0.75 (0.36to1.34) | | 0.01 (0.00to0.02) | 0.96 (0.46to1.66) | 1.21 (1.09to1.33) |
| Morocco | 9.84 (4.88to16.83) | 73.32 (37.42to122.08) | | 46.45 (23.36to78.48) | 147.42 (76.91to244.66) | 2.66 (2.56to2.76) | | 0.22 (0.12to0.36) | 1.95 (1.05to3.10) | | 0.95 (0.51to1.57) | 3.55 (1.94to5.67) | 2.39 (2.21to2.56) |
| Mozambique | 0.94 (0.36to2.21) | 18.12 (7.22to41.34) | | 2.45 (0.90to5.66) | 25.23 (9.52to55.76) | 1.50 (1.38to1.62) | | 0.04 (0.01to0.08) | 0.85 (0.34to1.86) | | 0.09 (0.03to0.20) | 1.12 (0.42to2.42) | 1.27 (1.15to1.39) |
| Myanmar | 14.60 (5.50to30.43) | 68.03 (26.75to136.58) | | 32.87 (12.81to64.67) | 77.77 (31.99to150.43) | 0.40 (0.35to0.45) | | 0.53 (0.21to1.08) | 2.91 (1.18to5.70) | | 1.20 (0.50to2.25) | 3.25 (1.40to5.95) | 0.32 (0.28to0.36) |
| Namibia | 0.95 (0.44to1.69) | 133.45 (64.26to234.19) | | 2.24 (1.05to3.99) | 165.00 (78.40to290.50) | 0.41 (0.00to0.81) | | 0.03 (0.02to0.06) | 5.43 (2.66to9.42) | | 0.08 (0.04to0.14) | 6.68 (3.22to11.37) | 0.33 (-0.11to0.77) |
| Nauru | 0.01 (0.00to0.02) | 260.16 (116.95to494.80) | | 0.02 (0.01to0.04) | 428.56 (195.45to821.61) | 1.45 (1.22to1.68) | | 0.00 (0.00to0.00) | 11.09 (5.14to20.14) | | 0.00 (0.00to0.00) | 17.33 (8.37to31.96) | 1.26 (1.04to1.49) |
| Nepal | 1.95 (0.83to3.93) | 25.73 (11.67to48.06) | | 9.44 (4.11to18.01) | 48.69 (22.18to89.99) | 2.20 (2.02to2.37) | | 0.05 (0.02to0.09) | 0.92 (0.43to1.67) | | 0.28 (0.13to0.48) | 1.84 (0.87to3.15) | 2.57 (2.31to2.84) |
| Netherlands | 4.84 (1.73to9.88) | 23.69 (8.44to48.18) | | 7.29 (2.70to14.96) | 20.83 (7.50to43.61) | -1.18 (-1.83to-0.53) | | 0.23 (0.09to0.44) | 1.13 (0.43to2.18) | | 0.27 (0.11to0.50) | 0.69 (0.27to1.27) | -2.10 (-2.61to-1.59) |
| New Zealand | 1.19 (0.50to2.19) | 30.97 (12.91to57.29) | | 3.33 (1.57to5.80) | 44.23 (20.27to78.66) | 0.80 (0.57to1.03) | | 0.04 (0.02to0.07) | 1.02 (0.46to1.78) | | 0.09 (0.04to0.14) | 1.03 (0.51to1.65) | -0.64 (-1.09to-0.18) |
| Nicaragua | 0.32 (0.11to0.77) | 22.05 (7.67to52.72) | | 1.67 (0.57to4.00) | 40.77 (14.17to94.78) | 1.80 (1.49to2.11) | | 0.01 (0.00to0.02) | 0.69 (0.24to1.57) | | 0.06 (0.02to0.12) | 1.62 (0.56to3.53) | 2.44 (1.81to3.07) |
| Niger | 0.81 (0.31to1.70) | 32.75 (13.34to64.15) | | 3.24 (1.26to6.49) | 47.00 (19.74to91.82) | 1.20 (1.09to1.32) | | 0.03 (0.01to0.06) | 1.52 (0.63to2.89) | | 0.11 (0.05to0.21) | 2.01 (0.85to3.79) | 0.99 (0.80to1.18) |
| Nigeria | 18.58 (7.51to36.78) | 45.78 (19.48to89.07) | | 43.45 (17.86to83.90) | 55.54 (23.64to103.54) | 0.52 (0.38to0.66) | | 0.69 (0.29to1.31) | 2.00 (0.88to3.72) | | 1.56 (0.67to2.90) | 2.47 (1.09to4.46) | 0.69 (0.52to0.85) |
| Niue | 0.01 (0.00to0.01) | 232.11 (105.93to422.73) | | 0.01 (0.00to0.02) | 398.02 (184.44to718.09) | 1.68 (1.40to1.97) | | 0.00 (0.00to0.00) | 9.34 (4.46to16.41) | | 0.00 (0.00to0.00) | 14.38 (7.00to25.68) | 1.31 (1.00to1.61) |
| North Macedonia | 0.61 (0.27to1.17) | 34.50 (15.78to65.00) | | 2.00 (0.90to3.78) | 64.81 (30.01to120.65) | 2.44 (2.14to2.75) | | 0.02 (0.01to0.03) | 1.09 (0.53to2.00) | | 0.06 (0.03to0.11) | 2.24 (1.03to4.10) | 3.04 (2.53to3.54) |
| Northern Mariana Islands | 0.03 (0.01to0.05) | 151.65 (73.14to267.81) | | 0.11 (0.05to0.20) | 214.70 (101.95to372.46) | 1.21 (0.76to1.66) | | 0.00 (0.00to0.00) | 5.71 (2.84to9.60) | | 0.00 (0.00to0.00) | 7.01 (3.56to11.41) | 0.88 (0.36to1.41) |
| Norway | 2.46 (1.12to4.33) | 37.40 (16.47to68.43) | | 4.31 (1.97to7.72) | 47.02 (21.03to86.31) | 0.31 (0.18to0.44) | | 0.06 (0.03to0.10) | 0.83 (0.42to1.34) | | 0.09 (0.04to0.14) | 0.75 (0.39to1.20) | -0.39 (-0.76to-0.02) |
| Oman | 1.07 (0.51to1.84) | 168.58 (85.87to281.02) | | 4.48 (2.34to7.29) | 259.84 (147.02to394.98) | 1.73 (1.55to1.91) | | 0.03 (0.02to0.05) | 6.62 (3.44to10.70) | | 0.10 (0.06to0.16) | 9.98 (5.87to14.65) | 1.87 (1.68to2.05) |
| Pakistan | 37.40 (17.39to66.89) | 69.08 (33.33to121.43) | | 135.23 (61.10to242.17) | 132.97 (62.90to230.34) | 2.32 (2.04to2.60) | | 1.18 (0.57to1.99) | 2.49 (1.22to4.16) | | 4.13 (1.96to7.13) | 5.24 (2.57to8.85) | 2.64 (2.33to2.94) |
| Palau | 0.02 (0.01to0.04) | 205.85 (92.84to372.30) | | 0.08 (0.03to0.15) | 366.14 (169.12to658.44) | 1.89 (1.59to2.19) | | 0.00 (0.00to0.00) | 8.16 (3.79to14.15) | | 0.00 (0.00to0.00) | 13.71 (6.68to23.13) | 1.68 (1.37to1.99) |
| Palestine | 1.57 (0.79to2.53) | 190.21 (97.02to302.75) | | 5.96 (3.13to9.78) | 271.98 (150.88to434.51) | 1.19 (0.88to1.50) | | 0.06 (0.03to0.09) | 8.11 (4.35to12.60) | | 0.19 (0.10to0.29) | 10.86 (6.10to16.51) | 0.97 (0.53to1.41) |
| Panama | 0.23 (0.08to0.54) | 15.87 (5.57to37.20) | | 1.24 (0.41to2.77) | 29.92 (9.81to67.25) | 1.89 (1.73to2.05) | | 0.01 (0.00to0.02) | 0.50 (0.18to1.13) | | 0.04 (0.01to0.09) | 1.01 (0.34to2.22) | 2.04 (1.73to2.35) |
| Papua New Guinea | 2.61 (1.02to5.38) | 143.32 (59.86to283.59) | | 10.45 (4.04to21.12) | 216.16 (89.98to413.55) | 1.31 (1.14to1.49) | | 0.08 (0.03to0.16) | 5.75 (2.47to10.72) | | 0.30 (0.12to0.59) | 8.35 (3.75to15.50) | 1.15 (0.96to1.34) |
| Paraguay | 0.67 (0.23to1.40) | 32.15 (11.38to66.31) | | 4.33 (1.57to8.64) | 81.42 (30.30to161.10) | 3.33 (3.03to3.64) | | 0.03 (0.01to0.05) | 1.34 (0.50to2.61) | | 0.17 (0.07to0.33) | 3.43 (1.36to6.46) | 3.48 (3.02to3.95) |
| Peru | 2.43 (0.86to4.96) | 21.37 (7.73to42.28) | | 11.91 (4.74to23.06) | 37.35 (14.87to72.34) | 2.17 (1.99to2.35) | | 0.07 (0.03to0.14) | 0.70 (0.26to1.35) | | 0.34 (0.14to0.62) | 1.06 (0.44to1.95) | 1.79 (1.52to2.07) |
| Philippines | 5.43 (2.02to12.19) | 22.65 (8.58to47.34) | | 17.56 (6.26to39.45) | 25.15 (9.24to55.32) | 0.35 (0.19to0.51) | | 0.21 (0.08to0.43) | 1.17 (0.45to2.40) | | 0.62 (0.22to1.37) | 1.04 (0.38to2.20) | -0.57 (-0.81to-0.34) |
| Poland | 18.90 (8.56to34.52) | 43.35 (19.71to79.47) | | 33.47 (15.48to61.41) | 49.15 (22.08to90.50) | 0.62 (0.29to0.95) | | 0.40 (0.19to0.70) | 0.92 (0.45to1.60) | | 0.68 (0.33to1.16) | 0.90 (0.44to1.54) | 0.21 (-0.05to0.48) |
| Portugal | 10.90 (5.14to18.49) | 78.31 (37.19to133.77) | | 21.24 (10.85to35.39) | 90.73 (44.34to156.48) | -0.04 (-0.37to0.30) | | 0.36 (0.18to0.57) | 2.66 (1.37to4.14) | | 0.67 (0.36to1.01) | 2.23 (1.21to3.40) | -1.33 (-1.73to-0.93) |
| Puerto Rico | 6.81 (3.65to11.21) | 189.23 (101.19to310.77) | | 14.83 (8.02to24.41) | 223.07 (115.46to374.05) | 0.46 (0.16to0.77) | | 0.22 (0.13to0.34) | 6.42 (3.61to9.64) | | 0.47 (0.26to0.75) | 5.86 (3.15to9.42) | -0.62 (-0.95to-0.29) |
| Qatar | 0.40 (0.21to0.66) | 413.58 (229.47to624.89) | | 4.84 (2.45to8.16) | 516.96 (286.24to781.98) | 1.03 (0.56to1.50) | | 0.01 (0.01to0.02) | 19.33 (11.10to28.98) | | 0.06 (0.03to0.09) | 22.53 (12.80to33.35) | 0.95 (0.45to1.45) |
| Republic of Korea | 15.51 (5.48to30.95) | 52.47 (20.06to100.67) | | 46.94 (19.59to87.14) | 53.29 (22.51to98.89) | -0.65 (-1.21to-0.08) | | 0.39 (0.16to0.75) | 1.61 (0.69to2.94) | | 1.44 (0.67to2.38) | 1.71 (0.80to2.84) | -0.56 (-1.48to0.36) |
| Republic of Moldova | 0.65 (0.27to1.26) | 15.71 (6.84to30.29) | | 1.11 (0.49to2.17) | 19.10 (8.32to37.57) | 0.54 (0.43to0.65) | | 0.01 (0.01to0.02) | 0.29 (0.14to0.53) | | 0.01 (0.01to0.03) | 0.25 (0.12to0.44) | -1.20 (-1.49to-0.92) |
| Romania | 5.43 (2.38to10.33) | 19.70 (8.77to37.76) | | 11.08 (5.17to20.44) | 30.28 (13.60to57.06) | 1.57 (1.33to1.81) | | 0.10 (0.05to0.19) | 0.39 (0.18to0.69) | | 0.18 (0.09to0.32) | 0.45 (0.21to0.79) | 0.70 (0.28to1.11) |
| Russian Federation | 19.05 (8.51to35.47) | 10.96 (4.91to20.23) | | 42.80 (20.11to77.64) | 17.72 (8.31to32.45) | 2.18 (1.87to2.50) | | 0.36 (0.17to0.65) | 0.22 (0.11to0.39) | | 1.19 (0.55to2.11) | 0.49 (0.23to0.87) | 3.22 (2.41to4.04) |
| Rwanda | 0.62 (0.23to1.50) | 23.50 (8.90to55.40) | | 1.02 (0.39to2.37) | 19.93 (7.72to44.54) | -1.14 (-1.38to-0.90) | | 0.02 (0.01to0.06) | 1.09 (0.42to2.38) | | 0.04 (0.01to0.08) | 0.94 (0.36to1.94) | -1.05 (-1.27to-0.83) |
| Saint Kitts and Nevis | 0.09 (0.04to0.14) | 231.06 (109.70to394.09) | | 0.15 (0.07to0.26) | 230.09 (113.54to391.89) | -0.08 (-0.20to0.04) | | 0.00 (0.00to0.01) | 9.06 (4.73to14.61) | | 0.00 (0.00to0.01) | 7.54 (4.09to12.01) | -0.39 (-0.56to-0.23) |
| Saint Lucia | 0.24 (0.11to0.40) | 275.48 (130.19to464.70) | | 0.59 (0.29to1.00) | 275.53 (137.85to465.15) | -0.61 (-0.81to-0.40) | | 0.01 (0.00to0.01) | 10.83 (5.72to17.42) | | 0.02 (0.01to0.03) | 8.72 (4.72to13.80) | -1.73 (-2.09to-1.37) |
| Saint Vincent and the Grenadines | 0.18 (0.09to0.31) | 258.48 (125.74to438.82) | | 0.42 (0.21to0.71) | 311.84 (156.28to525.99) | 0.28 (0.11to0.45) | | 0.01 (0.00to0.01) | 11.01 (5.75to17.49) | | 0.01 (0.01to0.02) | 11.27 (6.11to17.64) | -0.26 (-0.47to-0.04) |
| Samoa | 0.17 (0.08to0.32) | 200.12 (94.54to362.27) | | 0.43 (0.20to0.77) | 294.97 (140.32to513.20) | 1.11 (0.76to1.47) | | 0.01 (0.00to0.01) | 7.69 (3.78to13.40) | | 0.01 (0.01to0.02) | 10.46 (5.26to17.48) | 0.86 (0.51to1.21) |
| San Marino | 0.01 (0.01to0.02) | 33.01 (15.57to58.35) | | 0.03 (0.01to0.05) | 49.54 (22.52to90.28) | 1.50 (1.45to1.55) | | 0.00 (0.00to0.00) | 0.98 (0.50to1.59) | | 0.00 (0.00to0.00) | 0.83 (0.39to1.43) | -0.33 (-0.44to-0.22) |
| Sao Tome and Principe | 0.02 (0.01to0.03) | 24.93 (10.21to48.30) | | 0.04 (0.02to0.08) | 39.71 (16.68to77.97) | 1.50 (1.40to1.60) | | 0.00 (0.00to0.00) | 0.68 (0.28to1.28) | | 0.00 (0.00to0.00) | 1.01 (0.43to1.85) | 1.28 (1.20to1.36) |
| Saudi Arabia | 10.06 (5.25to16.06) | 156.71 (85.07to244.14) | | 52.64 (28.76to80.78) | 213.81 (120.60to324.13) | 1.51 (1.28to1.74) | | 0.22 (0.12to0.35) | 4.48 (2.45to6.86) | | 0.57 (0.32to0.85) | 3.81 (2.19to5.48) | -0.88 (-1.11to-0.65) |
| Senegal | 2.10 (0.89to3.96) | 68.84 (30.23to126.57) | | 6.86 (2.88to12.98) | 96.08 (42.08to176.42) | 1.19 (0.96to1.42) | | 0.06 (0.03to0.12) | 2.43 (1.08to4.37) | | 0.20 (0.09to0.36) | 3.33 (1.48to5.84) | 1.01 (0.76to1.25) |
| Serbia | 3.70 (1.63to7.08) | 33.98 (15.52to64.35) | | 7.71 (3.45to14.57) | 48.18 (21.37to91.60) | 1.30 (1.18to1.41) | | 0.10 (0.05to0.19) | 1.04 (0.49to1.92) | | 0.23 (0.11to0.42) | 1.45 (0.67to2.61) | 1.56 (1.32to1.81) |
| Seychelles | 0.02 (0.01to0.04) | 37.67 (15.81to72.27) | | 0.09 (0.03to0.17) | 83.25 (35.01to159.68) | 2.71 (2.56to2.87) | | 0.00 (0.00to0.00) | 1.03 (0.46to1.88) | | 0.00 (0.00to0.00) | 1.97 (0.89to3.57) | 2.21 (2.01to2.41) |
| Sierra Leone | 0.57 (0.23to1.18) | 31.95 (12.94to65.13) | | 1.66 (0.65to3.23) | 50.55 (20.64to95.27) | 1.78 (1.58to1.98) | | 0.02 (0.01to0.05) | 1.50 (0.63to2.87) | | 0.06 (0.03to0.11) | 2.23 (0.94to4.05) | 1.54 (1.30to1.78) |
| Singapore | 1.43 (0.53to2.81) | 65.56 (25.36to126.71) | | 3.00 (1.15to6.28) | 38.15 (14.87to78.45) | -1.86 (-2.12to-1.60) | | 0.03 (0.01to0.06) | 1.80 (0.80to3.13) | | 0.02 (0.01to0.03) | 0.27 (0.13to0.45) | -6.66 (-7.72to-5.60) |
| Slovakia | 1.63 (0.72to3.12) | 27.36 (12.10to52.16) | | 2.82 (1.25to5.37) | 31.08 (13.69to59.88) | 0.37 (0.06to0.69) | | 0.04 (0.02to0.08) | 0.76 (0.37to1.41) | | 0.05 (0.03to0.10) | 0.59 (0.27to1.05) | -0.72 (-1.03to-0.41) |
| Slovenia | 0.59 (0.26to1.09) | 24.20 (10.78to45.06) | | 1.26 (0.55to2.38) | 29.09 (12.44to56.88) | -0.33 (-0.72to0.07) | | 0.02 (0.01to0.03) | 0.67 (0.31to1.20) | | 0.03 (0.01to0.05) | 0.53 (0.25to0.97) | -2.90 (-3.78to-2.00) |
| Solomon Islands | 0.24 (0.09to0.54) | 162.56 (66.22to341.42) | | 1.09 (0.42to2.22) | 317.26 (133.08to609.03) | 2.24 (1.94to2.54) | | 0.01 (0.00to0.02) | 5.71 (2.50to11.55) | | 0.03 (0.01to0.06) | 11.28 (5.08to20.34) | 2.25 (1.95to2.56) |
| Somalia | 0.45 (0.16to1.09) | 20.81 (7.72to47.90) | | 1.22 (0.45to2.80) | 21.55 (8.29to48.02) | 0.32 (0.25to0.38) | | 0.02 (0.01to0.04) | 0.93 (0.35to2.10) | | 0.04 (0.01to0.09) | 0.94 (0.36to2.01) | 0.30 (0.22to0.39) |
| South Africa | 25.50 (11.86to43.81) | 120.86 (57.38to206.50) | | 76.05 (34.55to131.00) | 175.57 (80.95to297.75) | 1.85 (1.27to2.43) | | 0.83 (0.40to1.38) | 4.44 (2.17to7.30) | | 2.75 (1.30to4.58) | 7.27 (3.52to11.91) | 2.36 (1.69to3.03) |
| South Sudan | 0.39 (0.14to0.85) | 18.25 (6.84to38.76) | | 0.68 (0.26to1.52) | 20.90 (7.89to43.98) | 0.50 (0.47to0.53) | | 0.02 (0.01to0.03) | 0.85 (0.32to1.77) | | 0.02 (0.01to0.05) | 0.93 (0.36to1.90) | 0.36 (0.33to0.39) |
| Spain | 36.15 (16.60to64.26) | 65.87 (30.18to117.98) | | 60.22 (28.03to107.98) | 63.48 (28.16to119.08) | -0.56 (-0.80to-0.32) | | 1.19 (0.59to1.93) | 2.21 (1.10to3.58) | | 1.53 (0.81to2.43) | 1.17 (0.61to1.84) | -2.51 (-2.71to-2.30) |
| Sri Lanka | 3.31 (1.25to6.72) | 36.82 (14.00to72.36) | | 19.73 (7.50to39.08) | 83.42 (32.60to162.91) | 3.62 (3.29to3.95) | | 0.11 (0.04to0.21) | 1.42 (0.57to2.75) | | 0.72 (0.28to1.41) | 3.55 (1.42to7.00) | 4.09 (3.61to4.57) |
| Sudan | 9.31 (5.13to14.62) | 96.62 (53.60to150.32) | | 34.43 (19.08to53.83) | 167.93 (93.72to258.13) | 2.13 (2.04to2.22) | | 0.18 (0.09to0.28) | 2.18 (1.15to3.46) | | 0.49 (0.26to0.81) | 3.01 (1.57to4.84) | 1.41 (1.23to1.59) |
| Suriname | 0.37 (0.16to0.65) | 143.37 (65.43to246.93) | | 1.43 (0.67to2.50) | 236.07 (114.14to406.45) | 1.85 (1.67to2.03) | | 0.01 (0.01to0.02) | 4.46 (2.29to7.21) | | 0.03 (0.02to0.06) | 6.06 (3.18to9.83) | 1.09 (0.78to1.40) |
| Sweden | 4.13 (1.80to7.55) | 27.41 (11.51to51.44) | | 6.57 (2.80to12.17) | 31.82 (13.01to61.21) | -0.50 (-0.94to-0.07) | | 0.15 (0.07to0.25) | 0.90 (0.43to1.54) | | 0.22 (0.10to0.38) | 0.84 (0.38to1.44) | -0.97 (-1.25to-0.69) |
| Switzerland | 5.12 (2.54to8.65) | 47.85 (23.11to82.02) | | 7.75 (3.66to13.72) | 45.26 (20.45to82.89) | -0.51 (-0.61to-0.40) | | 0.21 (0.11to0.32) | 1.80 (0.93to2.83) | | 0.20 (0.11to0.31) | 0.91 (0.46to1.41) | -3.07 (-3.37to-2.76) |
| Syrian Arab Republic | 5.50 (2.67to9.33) | 103.76 (53.93to171.63) | | 15.57 (7.66to26.74) | 126.35 (65.27to209.81) | 0.23 (0.03to0.43) | | 0.13 (0.07to0.21) | 2.90 (1.63to4.61) | | 0.25 (0.13to0.41) | 2.61 (1.39to4.13) | -1.07 (-1.40to-0.74) |
| Taiwan (Province of China) | 8.05 (3.32to15.71) | 56.42 (24.57to105.62) | | 26.44 (11.82to48.56) | 66.03 (29.37to120.31) | -0.24 (-0.62to0.15) | | 0.30 (0.13to0.55) | 2.44 (1.12to4.25) | | 1.08 (0.51to1.90) | 2.69 (1.27to4.74) | -0.69 (-1.22to-0.15) |
| Tajikistan | 0.42 (0.17to0.86) | 15.01 (6.23to30.08) | | 1.87 (0.72to3.97) | 40.75 (17.20to80.07) | 3.86 (3.61to4.11) | | 0.01 (0.00to0.02) | 0.39 (0.17to0.76) | | 0.05 (0.02to0.10) | 1.30 (0.57to2.50) | 4.83 (4.50to5.15) |
| Thailand | 12.69 (4.99to25.61) | 39.77 (16.51to75.64) | | 49.23 (20.66to94.32) | 48.58 (20.51to93.07) | 0.10 (-0.20to0.40) | | 0.42 (0.17to0.81) | 1.49 (0.62to2.77) | | 1.34 (0.57to2.52) | 1.36 (0.58to2.54) | -1.13 (-1.56to-0.70) |
| Timor-Leste | 0.07 (0.03to0.16) | 32.36 (12.92to65.66) | | 0.32 (0.12to0.65) | 43.56 (17.07to86.89) | 0.91 (0.70to1.12) | | 0.00 (0.00to0.00) | 1.33 (0.53to2.60) | | 0.01 (0.00to0.02) | 1.52 (0.61to3.02) | 0.47 (0.24to0.70) |
| Togo | 0.41 (0.16to0.81) | 36.66 (14.87to70.85) | | 1.73 (0.66to3.58) | 53.32 (21.63to101.83) | 1.18 (0.96to1.40) | | 0.01 (0.01to0.03) | 1.63 (0.68to3.08) | | 0.06 (0.02to0.11) | 2.28 (0.96to4.29) | 1.08 (0.83to1.32) |
| Tokelau | 0.00 (0.00to0.00) | 204.96 (93.00to373.16) | | 0.00 (0.00to0.01) | 301.45 (139.45to548.87) | 1.25 (1.01to1.49) | | 0.00 (0.00to0.00) | 7.94 (3.81to13.71) | | 0.00 (0.00to0.00) | 11.30 (5.54to19.96) | 1.18 (0.91to1.46) |
| Tonga | 0.13 (0.06to0.23) | 234.04 (108.39to414.15) | | 0.24 (0.11to0.43) | 306.21 (144.03to538.03) | 0.82 (0.56to1.08) | | 0.00 (0.00to0.01) | 9.53 (4.62to16.12) | | 0.01 (0.00to0.02) | 11.92 (6.01to20.50) | 0.79 (0.53to1.06) |
| Trinidad and Tobago | 3.73 (1.82to6.27) | 447.16 (221.34to745.99) | | 8.34 (4.35to13.88) | 444.56 (232.79to733.89) | -0.39 (-0.56to-0.22) | | 0.13 (0.07to0.20) | 16.55 (9.00to25.94) | | 0.28 (0.15to0.45) | 15.23 (8.16to24.79) | -0.65 (-0.86to-0.44) |
| Tunisia | 1.43 (0.58to2.79) | 31.02 (12.91to59.09) | | 7.66 (3.23to14.62) | 62.32 (26.47to117.24) | 2.90 (2.73to3.07) | | 0.03 (0.01to0.06) | 0.78 (0.35to1.42) | | 0.14 (0.06to0.26) | 1.28 (0.57to2.31) | 1.76 (1.61to1.90) |
| Turkey | 45.98 (22.42to77.88) | 132.39 (66.47to218.62) | | 100.81 (52.94to167.54) | 115.10 (60.96to189.54) | 0.01 (-0.33to0.35) | | 1.61 (0.83to2.59) | 5.22 (2.69to8.22) | | 2.75 (1.54to4.25) | 3.36 (1.87to5.20) | -1.27 (-1.55to-0.98) |
| Turkmenistan | 0.29 (0.12to0.60) | 15.78 (6.70to30.88) | | 1.44 (0.57to2.86) | 36.41 (15.43to70.73) | 2.57 (2.37to2.76) | | 0.01 (0.00to0.01) | 0.39 (0.18to0.73) | | 0.03 (0.01to0.06) | 0.82 (0.36to1.56) | 1.85 (1.50to2.20) |
| Tuvalu | 0.01 (0.01to0.03) | 214.93 (92.98to408.01) | | 0.03 (0.01to0.06) | 315.99 (143.57to589.98) | 1.20 (0.94to1.46) | | 0.00 (0.00to0.00) | 8.57 (4.02to15.20) | | 0.00 (0.00to0.00) | 12.34 (5.79to22.37) | 1.09 (0.80to1.38) |
| Uganda | 1.12 (0.41to2.63) | 19.68 (7.41to44.97) | | 2.93 (1.11to6.76) | 24.06 (9.27to53.20) | 0.55 (0.39to0.70) | | 0.04 (0.02to0.10) | 0.90 (0.34to2.01) | | 0.10 (0.04to0.22) | 1.04 (0.40to2.16) | 0.33 (0.18to0.47) |
| Ukraine | 5.84 (2.47to11.63) | 8.41 (3.53to16.74) | | 7.15 (2.96to14.22) | 9.33 (3.80to18.97) | 0.22 (0.14to0.29) | | 0.09 (0.04to0.18) | 0.14 (0.07to0.26) | | 0.09 (0.04to0.18) | 0.12 (0.05to0.23) | -1.33 (-1.58to-1.07) |
| United Arab Emirates | 1.47 (0.84to2.26) | 342.20 (195.44to502.86) | | 18.30 (10.03to29.09) | 352.98 (199.05to537.36) | 0.11 (-0.35to0.58) | | 0.03 (0.02to0.05) | 13.79 (7.69to20.69) | | 0.24 (0.13to0.40) | 10.41 (5.83to15.87) | -0.96 (-1.65to-0.26) |
| United Kingdom | 36.16 (17.20to61.90) | 41.20 (19.25to72.17) | | 76.39 (36.47to137.99) | 68.50 (31.01to128.89) | 1.98 (1.86to2.10) | | 0.97 (0.50to1.52) | 1.04 (0.54to1.63) | | 0.91 (0.49to1.41) | 0.62 (0.33to0.97) | -2.01 (-2.17to-1.84) |
| United Republic of Tanzania | 1.56 (0.61to3.44) | 16.43 (6.46to34.97) | | 4.44 (1.68to9.81) | 20.61 (7.83to43.77) | 0.93 (0.89to0.98) | | 0.06 (0.03to0.14) | 0.83 (0.34to1.73) | | 0.18 (0.07to0.38) | 0.97 (0.37to2.02) | 0.55 (0.51to0.59) |
| United States of America | 151.74 (65.06to282.72) | 48.42 (20.30to91.34) | | 268.54 (112.24to521.56) | 50.59 (20.80to99.81) | 0.52 (0.29to0.75) | | 4.21 (1.93to7.26) | 1.26 (0.57to2.19) | | 5.45 (2.39to10.00) | 0.91 (0.40to1.68) | -1.70 (-2.33to-1.08) |
| United States Virgin Islands | 0.08 (0.03to0.14) | 98.69 (42.18to176.48) | | 0.22 (0.11to0.40) | 123.87 (60.38to220.60) | 0.74 (0.55to0.94) | | 0.00 (0.00to0.00) | 4.07 (1.84to7.09) | | 0.01 (0.00to0.01) | 4.28 (2.20to7.13) | 0.16 (-0.08to0.40) |
| Uruguay | 0.83 (0.27to1.80) | 21.25 (6.85to45.90) | | 2.10 (0.79to4.07) | 39.81 (14.51to79.07) | 2.34 (2.11to2.57) | | 0.04 (0.01to0.08) | 0.97 (0.32to2.01) | | 0.07 (0.03to0.13) | 1.19 (0.45to2.18) | 0.85 (0.75to0.95) |
| Uzbekistan | 1.36 (0.56to2.80) | 12.26 (5.13to24.39) | | 9.95 (3.92to20.25) | 50.25 (21.40to96.45) | 5.05 (4.71to5.39) | | 0.03 (0.01to0.06) | 0.31 (0.14to0.59) | | 0.27 (0.11to0.52) | 1.64 (0.73to3.05) | 5.85 (5.26to6.44) |
| Vanuatu | 0.05 (0.02to0.10) | 74.19 (30.04to155.26) | | 0.23 (0.09to0.46) | 139.96 (57.91to274.44) | 2.20 (2.08to2.31) | | 0.00 (0.00to0.00) | 2.83 (1.16to5.75) | | 0.01 (0.00to0.01) | 5.39 (2.29to10.32) | 2.35 (2.22to2.49) |
| Venezuela (Bolivarian Republic of) | 2.36 (0.80to5.48) | 25.61 (8.74to59.09) | | 9.86 (3.30to22.53) | 35.29 (11.78to79.71) | 0.97 (0.76to1.19) | | 0.07 (0.03to0.17) | 0.89 (0.31to1.98) | | 0.33 (0.11to0.73) | 1.26 (0.41to2.74) | 0.97 (0.72to1.21) |
| Viet Nam | 10.56 (3.89to21.85) | 28.85 (10.66to59.90) | | 32.31 (12.28to67.18) | 40.17 (15.46to81.64) | 1.36 (1.21to1.52) | | 0.47 (0.18to0.96) | 1.42 (0.53to2.82) | | 1.30 (0.52to2.54) | 1.78 (0.71to3.50) | 0.92 (0.75to1.09) |
| Yemen | 2.78 (1.31to5.09) | 58.39 (28.99to102.41) | | 11.46 (5.16to21.00) | 86.60 (41.90to151.19) | 1.56 (1.46to1.66) | | 0.06 (0.03to0.11) | 1.63 (0.81to2.87) | | 0.22 (0.11to0.39) | 2.04 (1.04to3.50) | 1.05 (0.90to1.21) |
| Zambia | 1.19 (0.45to2.53) | 45.90 (18.19to93.47) | | 3.32 (1.26to6.90) | 54.87 (21.75to110.54) | 0.46 (0.31to0.60) | | 0.04 (0.02to0.09) | 2.01 (0.79to3.97) | | 0.11 (0.04to0.22) | 2.30 (0.93to4.44) | 0.25 (0.07to0.44) |
| Zimbabwe | 1.03 (0.39to2.15) | 28.03 (11.03to57.69) | | 3.16 (1.18to6.91) | 49.47 (19.31to103.75) | 2.21 (1.96to2.46) | | 0.03 (0.01to0.07) | 1.14 (0.47to2.26) | | 0.10 (0.04to0.21) | 1.94 (0.78to3.85) | 2.16 (1.81to2.50) |

| **Supplemental table 7. DALY rates of cardiovascular diseases attributable to LPA by sex and age groups (1990-2019)** | | | | | | | | | | | | | | | | | | | |
| --- | --- | --- | --- | --- | --- | --- | --- | --- | --- | --- | --- | --- | --- | --- | --- | --- | --- | --- | --- |
|  | **25-44 years** | | |  | **45-59 years** | | |  | **60-74 years** | | |  | **75-94 years** | | |  | **95+ years** | | |
|  | Male | Female | M/F ratio |  | Male | Female | M/F ratio |  | Male | Female | M/F ratio |  | Male | Female | M/F ratio |  | Male | Female | M/F ratio |
| Global |  |  |  |  |  |  |  |  |  |  |  |  |  |  |  |  |  |  |  |
| 1990 | 121.90 | 93.79 | 1.30 |  | 574.61 | 372.88 | 1.54 |  | 2164.28 | 1703.99 | 1.27 |  | 12350.89 | 13832.02 | 0.89 |  | 4838.43 | 6417.90 | 0.75 |
| 1991 | 117.83 | 89.23 | 1.32 |  | 560.44 | 353.75 | 1.58 |  | 2156.68 | 1688.46 | 1.28 |  | 12180.38 | 13646.77 | 0.89 |  | 4825.26 | 6346.03 | 0.76 |
| 1992 | 115.98 | 87.27 | 1.33 |  | 550.85 | 344.82 | 1.60 |  | 2160.97 | 1686.71 | 1.28 |  | 12108.89 | 13541.26 | 0.89 |  | 4830.72 | 6315.19 | 0.76 |
| 1993 | 116.19 | 85.69 | 1.36 |  | 552.73 | 340.98 | 1.62 |  | 2187.92 | 1700.22 | 1.29 |  | 12199.73 | 13705.94 | 0.89 |  | 4875.70 | 6386.06 | 0.76 |
| 1994 | 116.98 | 85.30 | 1.37 |  | 551.28 | 336.63 | 1.64 |  | 2186.91 | 1688.95 | 1.29 |  | 12121.95 | 13635.09 | 0.89 |  | 4855.64 | 6391.04 | 0.76 |
| 1995 | 115.21 | 84.27 | 1.37 |  | 544.39 | 329.75 | 1.65 |  | 2162.79 | 1661.12 | 1.30 |  | 12024.57 | 13484.94 | 0.89 |  | 4795.07 | 6353.74 | 0.75 |
| 1996 | 112.69 | 83.16 | 1.36 |  | 532.80 | 326.55 | 1.63 |  | 2114.55 | 1631.61 | 1.30 |  | 11833.90 | 13276.07 | 0.89 |  | 4681.99 | 6306.93 | 0.74 |
| 1997 | 111.00 | 83.32 | 1.33 |  | 521.15 | 324.57 | 1.61 |  | 2070.47 | 1613.28 | 1.28 |  | 11671.03 | 13103.16 | 0.89 |  | 4593.84 | 6255.17 | 0.73 |
| 1998 | 109.56 | 83.90 | 1.31 |  | 507.90 | 321.64 | 1.58 |  | 2019.39 | 1597.26 | 1.26 |  | 11581.80 | 13006.03 | 0.89 |  | 4525.63 | 6224.14 | 0.73 |
| 1999 | 109.87 | 84.72 | 1.30 |  | 503.34 | 322.65 | 1.56 |  | 1974.79 | 1586.26 | 1.24 |  | 11478.48 | 13033.61 | 0.88 |  | 4441.21 | 6209.20 | 0.72 |
| 2000 | 109.79 | 84.42 | 1.30 |  | 498.07 | 320.72 | 1.55 |  | 1946.67 | 1563.22 | 1.25 |  | 11243.56 | 12843.37 | 0.88 |  | 4324.33 | 6087.02 | 0.71 |
| 2001 | 109.07 | 83.90 | 1.30 |  | 493.94 | 317.47 | 1.56 |  | 1924.57 | 1547.58 | 1.24 |  | 11088.07 | 12604.08 | 0.88 |  | 4272.53 | 5946.00 | 0.72 |
| 2002 | 108.72 | 82.43 | 1.32 |  | 491.37 | 313.69 | 1.57 |  | 1904.56 | 1534.75 | 1.24 |  | 11015.75 | 12397.75 | 0.89 |  | 4254.59 | 5815.01 | 0.73 |
| 2003 | 108.10 | 80.28 | 1.35 |  | 490.12 | 309.30 | 1.58 |  | 1880.09 | 1506.87 | 1.25 |  | 10870.49 | 12107.28 | 0.90 |  | 4184.50 | 5653.71 | 0.74 |
| 2004 | 107.61 | 78.80 | 1.37 |  | 482.66 | 302.50 | 1.60 |  | 1824.49 | 1451.73 | 1.26 |  | 10583.02 | 11641.57 | 0.91 |  | 4041.03 | 5451.24 | 0.74 |
| 2005 | 107.31 | 77.89 | 1.38 |  | 483.27 | 297.47 | 1.62 |  | 1798.43 | 1427.90 | 1.26 |  | 10433.24 | 11442.25 | 0.91 |  | 3943.94 | 5370.22 | 0.73 |
| 2006 | 107.03 | 77.52 | 1.38 |  | 476.93 | 290.25 | 1.64 |  | 1756.02 | 1401.70 | 1.25 |  | 10175.49 | 11141.80 | 0.91 |  | 3857.82 | 5279.73 | 0.73 |
| 2007 | 107.44 | 77.18 | 1.39 |  | 472.91 | 285.62 | 1.66 |  | 1727.07 | 1375.15 | 1.26 |  | 10055.56 | 10923.17 | 0.92 |  | 3780.26 | 5191.98 | 0.73 |
| 2008 | 108.73 | 77.77 | 1.40 |  | 472.18 | 283.04 | 1.67 |  | 1720.17 | 1356.22 | 1.27 |  | 10039.16 | 10814.73 | 0.93 |  | 3722.38 | 5127.96 | 0.73 |
| 2009 | 108.72 | 77.70 | 1.40 |  | 470.27 | 281.13 | 1.67 |  | 1701.53 | 1338.29 | 1.27 |  | 9932.21 | 10619.62 | 0.94 |  | 3647.10 | 4979.85 | 0.73 |
| 2010 | 109.12 | 77.78 | 1.40 |  | 468.64 | 281.18 | 1.67 |  | 1694.65 | 1330.48 | 1.27 |  | 9846.61 | 10466.11 | 0.94 |  | 3619.52 | 4909.93 | 0.74 |
| 2011 | 108.70 | 77.35 | 1.41 |  | 464.40 | 278.72 | 1.67 |  | 1663.80 | 1314.10 | 1.27 |  | 9688.46 | 10299.74 | 0.94 |  | 3563.27 | 4855.05 | 0.73 |
| 2012 | 108.27 | 77.27 | 1.40 |  | 461.12 | 278.09 | 1.66 |  | 1623.07 | 1297.72 | 1.25 |  | 9535.37 | 10079.12 | 0.95 |  | 3525.74 | 4766.59 | 0.74 |
| 2013 | 108.21 | 78.31 | 1.38 |  | 456.80 | 279.08 | 1.64 |  | 1570.92 | 1259.39 | 1.25 |  | 9555.91 | 9972.78 | 0.96 |  | 3534.66 | 4682.89 | 0.75 |
| 2014 | 107.90 | 78.61 | 1.37 |  | 451.65 | 285.29 | 1.58 |  | 1525.07 | 1216.19 | 1.25 |  | 9417.83 | 9850.91 | 0.96 |  | 3500.53 | 4616.59 | 0.76 |
| 2015 | 108.83 | 79.29 | 1.37 |  | 458.24 | 292.97 | 1.56 |  | 1523.63 | 1217.82 | 1.25 |  | 9257.38 | 9723.72 | 0.95 |  | 3439.71 | 4547.80 | 0.76 |
| 2016 | 111.20 | 80.41 | 1.38 |  | 464.59 | 294.81 | 1.58 |  | 1537.92 | 1218.19 | 1.26 |  | 9135.90 | 9554.26 | 0.96 |  | 3407.32 | 4481.49 | 0.76 |
| 2017 | 115.45 | 82.12 | 1.41 |  | 471.53 | 296.04 | 1.59 |  | 1549.69 | 1214.39 | 1.28 |  | 9100.51 | 9477.00 | 0.96 |  | 3407.64 | 4441.87 | 0.77 |
| 2018 | 117.10 | 80.52 | 1.45 |  | 459.97 | 280.19 | 1.64 |  | 1505.64 | 1168.60 | 1.29 |  | 9087.37 | 9475.90 | 0.96 |  | 3372.73 | 4434.88 | 0.76 |
| 2019 | 118.58 | 78.07 | 1.52 |  | 448.45 | 263.06 | 1.70 |  | 1458.41 | 1115.11 | 1.31 |  | 9090.82 | 9425.58 | 0.96 |  | 3337.10 | 4417.52 | 0.76 |
| Low SDI |  |  |  |  |  |  |  |  |  |  |  |  |  |  |  |  |  |  |  |
| 1990 | 97.33 | 85.12 | 1.14 |  | 560.44 | 412.45 | 1.36 |  | 1835.87 | 1610.45 | 1.14 |  | 7591.24 | 7113.38 | 1.07 |  | 2801.00 | 2544.23 | 1.10 |
| 1991 | 95.29 | 80.55 | 1.18 |  | 558.57 | 384.27 | 1.45 |  | 1868.94 | 1574.07 | 1.19 |  | 7602.04 | 7110.07 | 1.07 |  | 2833.64 | 2553.22 | 1.11 |
| 1992 | 95.05 | 78.92 | 1.20 |  | 559.22 | 372.04 | 1.50 |  | 1899.84 | 1555.69 | 1.22 |  | 7623.98 | 7134.91 | 1.07 |  | 2862.26 | 2577.30 | 1.11 |
| 1993 | 95.74 | 77.78 | 1.23 |  | 563.46 | 365.25 | 1.54 |  | 1936.61 | 1540.35 | 1.26 |  | 7642.24 | 7150.01 | 1.07 |  | 2898.44 | 2583.38 | 1.12 |
| 1994 | 96.94 | 76.51 | 1.27 |  | 566.74 | 363.70 | 1.56 |  | 1964.63 | 1528.38 | 1.29 |  | 7634.97 | 7120.99 | 1.07 |  | 2935.78 | 2592.60 | 1.13 |
| 1995 | 97.59 | 76.80 | 1.27 |  | 571.54 | 361.44 | 1.58 |  | 1971.28 | 1520.89 | 1.30 |  | 7621.69 | 7109.88 | 1.07 |  | 2939.46 | 2637.65 | 1.11 |
| 1996 | 98.25 | 76.95 | 1.28 |  | 573.92 | 365.20 | 1.57 |  | 1967.25 | 1523.64 | 1.29 |  | 7639.93 | 7119.48 | 1.07 |  | 2941.94 | 2675.78 | 1.10 |
| 1997 | 99.42 | 78.35 | 1.27 |  | 576.18 | 373.36 | 1.54 |  | 1969.09 | 1543.62 | 1.28 |  | 7716.01 | 7193.57 | 1.07 |  | 2951.41 | 2681.36 | 1.10 |
| 1998 | 99.83 | 80.11 | 1.25 |  | 571.28 | 382.90 | 1.49 |  | 1945.65 | 1575.78 | 1.23 |  | 7718.08 | 7248.48 | 1.06 |  | 2946.20 | 2707.17 | 1.09 |
| 1999 | 100.40 | 82.02 | 1.22 |  | 568.05 | 394.52 | 1.44 |  | 1906.09 | 1591.26 | 1.20 |  | 7654.22 | 7238.51 | 1.06 |  | 2934.43 | 2713.23 | 1.08 |
| 2000 | 101.90 | 83.25 | 1.22 |  | 566.77 | 398.67 | 1.42 |  | 1908.84 | 1604.89 | 1.19 |  | 7694.72 | 7301.62 | 1.05 |  | 2922.63 | 2720.82 | 1.07 |
| 2001 | 103.65 | 84.94 | 1.22 |  | 567.96 | 399.32 | 1.42 |  | 1920.17 | 1630.40 | 1.18 |  | 7712.57 | 7392.31 | 1.04 |  | 2918.08 | 2748.40 | 1.06 |
| 2002 | 104.93 | 85.52 | 1.23 |  | 562.57 | 395.43 | 1.42 |  | 1909.98 | 1640.11 | 1.16 |  | 7773.28 | 7482.54 | 1.04 |  | 2933.88 | 2768.81 | 1.06 |
| 2003 | 106.96 | 85.67 | 1.25 |  | 555.28 | 391.57 | 1.42 |  | 1917.99 | 1631.81 | 1.18 |  | 7974.68 | 7463.02 | 1.07 |  | 3007.20 | 2811.73 | 1.07 |
| 2004 | 108.39 | 85.68 | 1.27 |  | 544.82 | 386.13 | 1.41 |  | 1893.82 | 1606.70 | 1.18 |  | 7848.28 | 7447.59 | 1.05 |  | 3028.02 | 2841.22 | 1.07 |
| 2005 | 109.16 | 85.17 | 1.28 |  | 537.89 | 377.31 | 1.43 |  | 1881.71 | 1605.60 | 1.17 |  | 7868.97 | 7507.31 | 1.05 |  | 3058.96 | 2876.17 | 1.06 |
| 2006 | 110.19 | 85.43 | 1.29 |  | 529.33 | 370.44 | 1.43 |  | 1878.37 | 1619.56 | 1.16 |  | 7927.72 | 7545.48 | 1.05 |  | 3095.68 | 2890.04 | 1.07 |
| 2007 | 112.05 | 85.27 | 1.31 |  | 527.72 | 364.21 | 1.45 |  | 1890.52 | 1612.20 | 1.17 |  | 8015.04 | 7499.48 | 1.07 |  | 3153.75 | 2894.15 | 1.09 |
| 2008 | 113.38 | 86.30 | 1.31 |  | 520.27 | 361.51 | 1.44 |  | 1883.61 | 1618.37 | 1.16 |  | 8084.08 | 7503.96 | 1.08 |  | 3194.26 | 2908.82 | 1.10 |
| 2009 | 113.47 | 85.55 | 1.33 |  | 518.46 | 357.30 | 1.45 |  | 1875.72 | 1613.94 | 1.16 |  | 7979.89 | 7349.87 | 1.09 |  | 3230.30 | 2935.15 | 1.10 |
| 2010 | 113.14 | 84.54 | 1.34 |  | 517.34 | 355.85 | 1.45 |  | 1857.12 | 1592.87 | 1.17 |  | 7886.68 | 7169.52 | 1.10 |  | 3295.64 | 2931.60 | 1.12 |
| 2011 | 112.73 | 84.03 | 1.34 |  | 512.98 | 353.06 | 1.45 |  | 1837.65 | 1589.45 | 1.16 |  | 8150.64 | 7126.13 | 1.14 |  | 3689.44 | 2910.25 | 1.27 |
| 2012 | 112.21 | 83.50 | 1.34 |  | 509.22 | 357.01 | 1.43 |  | 1817.56 | 1618.63 | 1.12 |  | 8022.58 | 7184.34 | 1.12 |  | 3499.21 | 2900.29 | 1.21 |
| 2013 | 111.43 | 82.54 | 1.35 |  | 507.28 | 355.91 | 1.43 |  | 1785.57 | 1587.58 | 1.12 |  | 8095.72 | 7283.55 | 1.11 |  | 3381.06 | 2913.17 | 1.16 |
| 2014 | 109.22 | 81.70 | 1.34 |  | 503.35 | 366.05 | 1.38 |  | 1725.16 | 1554.52 | 1.11 |  | 7868.33 | 7342.97 | 1.07 |  | 3250.12 | 2897.34 | 1.12 |
| 2015 | 109.13 | 80.69 | 1.35 |  | 510.04 | 373.00 | 1.37 |  | 1721.79 | 1549.18 | 1.11 |  | 7765.03 | 7240.92 | 1.07 |  | 3161.97 | 2839.89 | 1.11 |
| 2016 | 107.45 | 79.25 | 1.36 |  | 505.48 | 371.67 | 1.36 |  | 1703.82 | 1540.36 | 1.11 |  | 7559.13 | 7138.92 | 1.06 |  | 3026.91 | 2770.98 | 1.09 |
| 2017 | 106.78 | 78.77 | 1.36 |  | 506.73 | 374.35 | 1.35 |  | 1698.09 | 1537.65 | 1.10 |  | 7456.85 | 7166.95 | 1.04 |  | 2935.88 | 2739.97 | 1.07 |
| 2018 | 105.89 | 75.92 | 1.39 |  | 501.14 | 356.55 | 1.41 |  | 1628.31 | 1444.43 | 1.13 |  | 7423.39 | 7177.18 | 1.03 |  | 2892.07 | 2734.64 | 1.06 |
| 2019 | 104.69 | 72.57 | 1.44 |  | 491.42 | 334.81 | 1.47 |  | 1531.57 | 1325.28 | 1.16 |  | 7213.18 | 6975.00 | 1.03 |  | 2792.58 | 2651.35 | 1.05 |
| Low-middle SDI |  |  |  |  |  |  |  |  |  |  |  |  |  |  |  |  |  |  |  |
| 1990 | 128.15 | 126.52 | 1.01 |  | 571.20 | 488.82 | 1.17 |  | 2094.18 | 1941.91 | 1.08 |  | 10094.67 | 9621.59 | 1.05 |  | 4381.14 | 4123.95 | 1.06 |
| 1991 | 125.92 | 118.00 | 1.07 |  | 576.69 | 443.34 | 1.30 |  | 2158.86 | 1906.96 | 1.13 |  | 10062.16 | 9673.45 | 1.04 |  | 4362.86 | 4137.32 | 1.05 |
| 1992 | 125.01 | 114.14 | 1.10 |  | 577.62 | 423.36 | 1.36 |  | 2224.20 | 1898.65 | 1.17 |  | 10139.98 | 9782.63 | 1.04 |  | 4364.00 | 4163.00 | 1.05 |
| 1993 | 125.23 | 110.78 | 1.13 |  | 585.56 | 407.52 | 1.44 |  | 2280.22 | 1873.06 | 1.22 |  | 10292.81 | 9889.22 | 1.04 |  | 4464.67 | 4214.41 | 1.06 |
| 1994 | 126.91 | 108.70 | 1.17 |  | 588.38 | 401.56 | 1.47 |  | 2299.08 | 1839.31 | 1.25 |  | 10329.99 | 9890.95 | 1.04 |  | 4508.37 | 4227.11 | 1.07 |
| 1995 | 126.92 | 107.34 | 1.18 |  | 589.49 | 397.95 | 1.48 |  | 2289.98 | 1811.36 | 1.26 |  | 10325.67 | 9857.61 | 1.05 |  | 4490.13 | 4205.19 | 1.07 |
| 1996 | 125.98 | 107.03 | 1.18 |  | 589.04 | 405.95 | 1.45 |  | 2261.63 | 1802.49 | 1.25 |  | 10330.15 | 9815.33 | 1.05 |  | 4473.23 | 4169.82 | 1.07 |
| 1997 | 126.72 | 109.53 | 1.16 |  | 592.58 | 420.75 | 1.41 |  | 2262.61 | 1850.78 | 1.22 |  | 10439.00 | 9886.34 | 1.06 |  | 4514.58 | 4155.03 | 1.09 |
| 1998 | 126.25 | 111.58 | 1.13 |  | 579.76 | 428.74 | 1.35 |  | 2198.18 | 1874.25 | 1.17 |  | 10495.36 | 9998.91 | 1.05 |  | 4568.22 | 4146.03 | 1.10 |
| 1999 | 126.53 | 113.88 | 1.11 |  | 573.42 | 438.53 | 1.31 |  | 2088.16 | 1854.65 | 1.13 |  | 10424.61 | 10065.78 | 1.04 |  | 4556.77 | 4089.58 | 1.11 |
| 2000 | 126.66 | 113.33 | 1.12 |  | 572.57 | 439.40 | 1.30 |  | 2062.88 | 1840.11 | 1.12 |  | 10316.54 | 10133.87 | 1.02 |  | 4465.06 | 4074.08 | 1.10 |
| 2001 | 126.01 | 112.75 | 1.12 |  | 568.00 | 433.69 | 1.31 |  | 2070.90 | 1856.25 | 1.12 |  | 10290.28 | 10277.22 | 1.00 |  | 4427.82 | 4085.91 | 1.08 |
| 2002 | 125.08 | 110.94 | 1.13 |  | 563.77 | 426.04 | 1.32 |  | 2063.11 | 1868.16 | 1.10 |  | 10516.14 | 10417.95 | 1.01 |  | 4533.24 | 4083.80 | 1.11 |
| 2003 | 123.22 | 107.00 | 1.15 |  | 562.95 | 414.61 | 1.36 |  | 2057.31 | 1822.30 | 1.13 |  | 10747.69 | 10363.77 | 1.04 |  | 4717.82 | 4090.00 | 1.15 |
| 2004 | 123.46 | 103.95 | 1.19 |  | 551.60 | 402.12 | 1.37 |  | 2012.21 | 1747.28 | 1.15 |  | 10662.55 | 10244.97 | 1.04 |  | 4723.17 | 4081.32 | 1.16 |
| 2005 | 123.58 | 102.20 | 1.21 |  | 556.01 | 393.66 | 1.41 |  | 2029.73 | 1761.55 | 1.15 |  | 10696.44 | 10334.78 | 1.03 |  | 4692.49 | 4087.92 | 1.15 |
| 2006 | 125.74 | 102.62 | 1.23 |  | 561.81 | 388.71 | 1.45 |  | 2065.70 | 1800.73 | 1.15 |  | 10836.42 | 10386.59 | 1.04 |  | 4744.14 | 4075.13 | 1.16 |
| 2007 | 127.91 | 103.25 | 1.24 |  | 565.15 | 388.57 | 1.45 |  | 2076.90 | 1810.17 | 1.15 |  | 10813.16 | 10377.43 | 1.04 |  | 4755.65 | 4106.52 | 1.16 |
| 2008 | 129.67 | 104.48 | 1.24 |  | 569.97 | 386.57 | 1.47 |  | 2102.08 | 1798.29 | 1.17 |  | 10781.19 | 10372.52 | 1.04 |  | 4728.03 | 4178.20 | 1.13 |
| 2009 | 129.52 | 103.55 | 1.25 |  | 576.52 | 386.28 | 1.49 |  | 2105.75 | 1778.88 | 1.18 |  | 10535.13 | 10013.42 | 1.05 |  | 4687.46 | 4183.77 | 1.12 |
| 2010 | 130.13 | 103.68 | 1.26 |  | 585.11 | 393.34 | 1.49 |  | 2118.76 | 1790.86 | 1.18 |  | 10323.37 | 9706.16 | 1.06 |  | 4655.98 | 4184.19 | 1.11 |
| 2011 | 131.07 | 104.02 | 1.26 |  | 590.52 | 398.04 | 1.48 |  | 2104.32 | 1821.44 | 1.16 |  | 9901.68 | 9596.19 | 1.03 |  | 4442.27 | 4130.11 | 1.08 |
| 2012 | 131.15 | 104.13 | 1.26 |  | 597.82 | 403.91 | 1.48 |  | 2073.70 | 1849.21 | 1.12 |  | 9640.47 | 9579.05 | 1.01 |  | 4305.28 | 4038.08 | 1.07 |
| 2013 | 128.92 | 106.73 | 1.21 |  | 594.98 | 414.83 | 1.43 |  | 2011.97 | 1797.38 | 1.12 |  | 10626.97 | 9926.71 | 1.07 |  | 4691.88 | 4070.82 | 1.15 |
| 2014 | 127.14 | 107.71 | 1.18 |  | 584.46 | 437.24 | 1.34 |  | 1946.53 | 1729.40 | 1.13 |  | 10916.44 | 10163.40 | 1.07 |  | 4775.75 | 4118.32 | 1.16 |
| 2015 | 125.18 | 107.72 | 1.16 |  | 591.78 | 457.81 | 1.29 |  | 1950.01 | 1768.93 | 1.10 |  | 10449.72 | 9985.54 | 1.05 |  | 4628.93 | 4090.29 | 1.13 |
| 2016 | 124.50 | 106.37 | 1.17 |  | 586.94 | 453.69 | 1.29 |  | 1973.00 | 1789.17 | 1.10 |  | 10029.74 | 9791.48 | 1.02 |  | 4474.64 | 4028.42 | 1.11 |
| 2017 | 124.20 | 105.27 | 1.18 |  | 576.52 | 445.35 | 1.29 |  | 1986.89 | 1786.00 | 1.11 |  | 10002.20 | 9770.43 | 1.02 |  | 4385.20 | 4007.07 | 1.09 |
| 2018 | 124.44 | 100.95 | 1.23 |  | 549.85 | 404.13 | 1.36 |  | 1888.86 | 1662.41 | 1.14 |  | 10122.30 | 9787.96 | 1.03 |  | 4336.27 | 4009.66 | 1.08 |
| 2019 | 125.18 | 95.30 | 1.31 |  | 522.43 | 360.34 | 1.45 |  | 1769.02 | 1529.11 | 1.16 |  | 10019.71 | 9642.19 | 1.04 |  | 4262.23 | 3974.49 | 1.07 |
| Middle SDI |  |  |  |  |  |  |  |  |  |  |  |  |  |  |  |  |  |  |  |
| 1990 | 127.27 | 103.05 | 1.24 |  | 541.94 | 387.17 | 1.40 |  | 2056.59 | 1735.44 | 1.19 |  | 11862.29 | 10779.59 | 1.10 |  | 4882.96 | 4295.49 | 1.14 |
| 1991 | 125.50 | 99.44 | 1.26 |  | 543.24 | 373.81 | 1.45 |  | 2106.23 | 1752.67 | 1.20 |  | 11799.26 | 10861.35 | 1.09 |  | 4848.71 | 4346.10 | 1.12 |
| 1992 | 124.59 | 98.50 | 1.26 |  | 541.48 | 370.86 | 1.46 |  | 2138.89 | 1779.68 | 1.20 |  | 11820.43 | 11073.90 | 1.07 |  | 4865.35 | 4423.80 | 1.10 |
| 1993 | 124.58 | 95.58 | 1.30 |  | 542.96 | 364.93 | 1.49 |  | 2172.06 | 1789.74 | 1.21 |  | 11910.73 | 11250.03 | 1.06 |  | 4890.53 | 4483.82 | 1.09 |
| 1994 | 125.93 | 95.00 | 1.33 |  | 542.49 | 357.56 | 1.52 |  | 2183.85 | 1774.67 | 1.23 |  | 11894.09 | 11286.54 | 1.05 |  | 4863.96 | 4496.55 | 1.08 |
| 1995 | 124.71 | 92.56 | 1.35 |  | 540.58 | 347.34 | 1.56 |  | 2169.03 | 1745.63 | 1.24 |  | 11885.53 | 11299.45 | 1.05 |  | 4814.46 | 4493.86 | 1.07 |
| 1996 | 124.28 | 91.21 | 1.36 |  | 541.95 | 346.79 | 1.56 |  | 2147.09 | 1738.52 | 1.24 |  | 11872.54 | 11374.79 | 1.04 |  | 4720.51 | 4520.10 | 1.04 |
| 1997 | 123.93 | 91.48 | 1.35 |  | 541.06 | 347.54 | 1.56 |  | 2123.54 | 1736.16 | 1.22 |  | 11811.51 | 11370.04 | 1.04 |  | 4572.35 | 4513.28 | 1.01 |
| 1998 | 123.02 | 91.93 | 1.34 |  | 533.07 | 345.79 | 1.54 |  | 2087.01 | 1739.29 | 1.20 |  | 11856.34 | 11387.36 | 1.04 |  | 4442.56 | 4505.65 | 0.99 |
| 1999 | 123.20 | 92.10 | 1.34 |  | 528.80 | 344.28 | 1.54 |  | 2038.37 | 1731.79 | 1.18 |  | 11791.19 | 11476.82 | 1.03 |  | 4278.66 | 4489.84 | 0.95 |
| 2000 | 123.49 | 91.52 | 1.35 |  | 524.74 | 341.52 | 1.54 |  | 2023.44 | 1719.14 | 1.18 |  | 11706.88 | 11678.95 | 1.00 |  | 4108.87 | 4508.75 | 0.91 |
| 2001 | 123.19 | 90.17 | 1.37 |  | 523.36 | 337.66 | 1.55 |  | 2019.15 | 1713.38 | 1.18 |  | 11789.18 | 11835.88 | 1.00 |  | 4078.63 | 4547.36 | 0.90 |
| 2002 | 122.90 | 87.67 | 1.40 |  | 521.71 | 333.66 | 1.56 |  | 2008.53 | 1703.23 | 1.18 |  | 11917.70 | 11886.00 | 1.00 |  | 4122.31 | 4532.57 | 0.91 |
| 2003 | 120.98 | 84.34 | 1.43 |  | 518.74 | 328.35 | 1.58 |  | 1997.30 | 1683.37 | 1.19 |  | 12075.61 | 11905.61 | 1.01 |  | 4158.28 | 4551.71 | 0.91 |
| 2004 | 118.59 | 81.89 | 1.45 |  | 509.07 | 321.17 | 1.59 |  | 1956.06 | 1635.11 | 1.20 |  | 12159.58 | 11897.19 | 1.02 |  | 4165.08 | 4564.82 | 0.91 |
| 2005 | 116.45 | 79.51 | 1.46 |  | 506.13 | 314.03 | 1.61 |  | 1931.27 | 1599.39 | 1.21 |  | 12099.41 | 11906.12 | 1.02 |  | 4060.43 | 4516.55 | 0.90 |
| 2006 | 115.56 | 78.49 | 1.47 |  | 505.80 | 308.02 | 1.64 |  | 1905.11 | 1581.67 | 1.20 |  | 11771.86 | 11812.64 | 1.00 |  | 3884.49 | 4418.50 | 0.88 |
| 2007 | 115.16 | 77.20 | 1.49 |  | 503.55 | 301.94 | 1.67 |  | 1888.75 | 1556.07 | 1.21 |  | 11769.75 | 11738.43 | 1.00 |  | 3808.83 | 4382.21 | 0.87 |
| 2008 | 116.44 | 77.74 | 1.50 |  | 505.71 | 299.99 | 1.69 |  | 1904.23 | 1548.36 | 1.23 |  | 12000.82 | 11843.04 | 1.01 |  | 3801.85 | 4402.21 | 0.86 |
| 2009 | 116.88 | 78.14 | 1.50 |  | 511.03 | 301.18 | 1.70 |  | 1910.13 | 1550.24 | 1.23 |  | 12163.33 | 11899.79 | 1.02 |  | 3791.52 | 4441.20 | 0.85 |
| 2010 | 117.67 | 78.57 | 1.50 |  | 510.56 | 302.48 | 1.69 |  | 1919.13 | 1558.76 | 1.23 |  | 12287.50 | 12013.51 | 1.02 |  | 3771.59 | 4543.90 | 0.83 |
| 2011 | 117.78 | 77.77 | 1.51 |  | 510.50 | 298.40 | 1.71 |  | 1909.34 | 1541.96 | 1.24 |  | 12266.14 | 11950.11 | 1.03 |  | 3674.06 | 4556.95 | 0.81 |
| 2012 | 118.19 | 78.18 | 1.51 |  | 511.48 | 297.75 | 1.72 |  | 1889.55 | 1529.75 | 1.24 |  | 12233.04 | 11623.55 | 1.05 |  | 3703.37 | 4472.52 | 0.83 |
| 2013 | 119.03 | 78.58 | 1.51 |  | 510.39 | 296.77 | 1.72 |  | 1848.72 | 1494.35 | 1.24 |  | 12302.16 | 11575.22 | 1.06 |  | 3786.27 | 4475.12 | 0.85 |
| 2014 | 118.63 | 78.09 | 1.52 |  | 508.92 | 301.14 | 1.69 |  | 1817.84 | 1449.46 | 1.25 |  | 12201.29 | 11486.39 | 1.06 |  | 3836.76 | 4462.03 | 0.86 |
| 2015 | 119.63 | 78.79 | 1.52 |  | 519.84 | 308.93 | 1.68 |  | 1827.17 | 1449.33 | 1.26 |  | 12054.77 | 11267.95 | 1.07 |  | 3878.35 | 4391.75 | 0.88 |
| 2016 | 121.10 | 80.48 | 1.50 |  | 524.22 | 311.24 | 1.68 |  | 1841.50 | 1452.23 | 1.27 |  | 11913.18 | 11189.91 | 1.06 |  | 3901.98 | 4390.04 | 0.89 |
| 2017 | 125.17 | 82.86 | 1.51 |  | 525.59 | 312.36 | 1.68 |  | 1845.08 | 1456.36 | 1.27 |  | 11776.66 | 11231.25 | 1.05 |  | 3947.69 | 4454.45 | 0.89 |
| 2018 | 127.71 | 81.21 | 1.57 |  | 505.97 | 291.91 | 1.73 |  | 1790.42 | 1395.75 | 1.28 |  | 11773.57 | 11211.59 | 1.05 |  | 3960.33 | 4492.37 | 0.88 |
| 2019 | 129.84 | 78.78 | 1.65 |  | 486.91 | 269.99 | 1.80 |  | 1729.84 | 1315.88 | 1.31 |  | 11839.52 | 11074.92 | 1.07 |  | 3962.76 | 4476.26 | 0.89 |
| High-middle SDI |  |  |  |  |  |  |  |  |  |  |  |  |  |  |  |  |  |  |  |
| 1990 | 141.46 | 80.58 | 1.76 |  | 664.47 | 340.12 | 1.95 |  | 2468.99 | 1780.21 | 1.39 |  | 16266.72 | 17146.19 | 0.95 |  | 6612.95 | 7642.32 | 0.87 |
| 1991 | 137.14 | 77.62 | 1.77 |  | 642.57 | 331.07 | 1.94 |  | 2419.42 | 1776.64 | 1.36 |  | 15917.16 | 16805.24 | 0.95 |  | 6527.54 | 7434.18 | 0.88 |
| 1992 | 136.03 | 76.22 | 1.78 |  | 638.33 | 329.08 | 1.94 |  | 2421.32 | 1795.74 | 1.35 |  | 15726.12 | 16612.44 | 0.95 |  | 6446.88 | 7335.51 | 0.88 |
| 1993 | 138.66 | 77.18 | 1.80 |  | 658.88 | 340.44 | 1.94 |  | 2484.65 | 1864.23 | 1.33 |  | 15824.81 | 16990.18 | 0.93 |  | 6404.91 | 7425.96 | 0.86 |
| 1994 | 139.80 | 78.96 | 1.77 |  | 669.50 | 344.12 | 1.95 |  | 2508.83 | 1892.00 | 1.33 |  | 15675.53 | 17078.30 | 0.92 |  | 6288.78 | 7459.90 | 0.84 |
| 1995 | 134.38 | 78.77 | 1.71 |  | 652.73 | 336.79 | 1.94 |  | 2486.23 | 1867.22 | 1.33 |  | 15418.44 | 16823.69 | 0.92 |  | 6068.30 | 7363.56 | 0.82 |
| 1996 | 126.03 | 76.47 | 1.65 |  | 616.77 | 324.86 | 1.90 |  | 2420.40 | 1825.27 | 1.33 |  | 15086.77 | 16571.84 | 0.91 |  | 5832.59 | 7319.27 | 0.80 |
| 1997 | 118.57 | 74.28 | 1.60 |  | 580.34 | 310.85 | 1.87 |  | 2348.41 | 1779.35 | 1.32 |  | 14911.63 | 16513.06 | 0.90 |  | 5699.50 | 7370.62 | 0.77 |
| 1998 | 113.87 | 73.63 | 1.55 |  | 555.91 | 298.95 | 1.86 |  | 2288.86 | 1738.01 | 1.32 |  | 14829.84 | 16567.70 | 0.90 |  | 5647.98 | 7485.40 | 0.75 |
| 1999 | 114.06 | 73.71 | 1.55 |  | 554.57 | 297.91 | 1.86 |  | 2297.29 | 1742.82 | 1.32 |  | 14817.26 | 16926.59 | 0.88 |  | 5511.27 | 7587.85 | 0.73 |
| 2000 | 112.50 | 72.50 | 1.55 |  | 544.46 | 294.02 | 1.85 |  | 2286.91 | 1723.28 | 1.33 |  | 14516.20 | 16791.90 | 0.86 |  | 5403.70 | 7557.92 | 0.71 |
| 2001 | 109.43 | 70.74 | 1.55 |  | 531.91 | 288.66 | 1.84 |  | 2260.57 | 1701.02 | 1.33 |  | 14438.88 | 16487.02 | 0.88 |  | 5492.45 | 7474.92 | 0.73 |
| 2002 | 106.79 | 67.48 | 1.58 |  | 524.43 | 285.53 | 1.84 |  | 2256.69 | 1693.22 | 1.33 |  | 14599.02 | 16359.68 | 0.89 |  | 5767.41 | 7523.37 | 0.77 |
| 2003 | 104.93 | 63.87 | 1.64 |  | 519.59 | 282.85 | 1.84 |  | 2228.92 | 1672.31 | 1.33 |  | 14501.01 | 16158.82 | 0.90 |  | 5764.94 | 7420.38 | 0.78 |
| 2004 | 102.63 | 61.42 | 1.67 |  | 508.50 | 274.82 | 1.85 |  | 2156.10 | 1611.40 | 1.34 |  | 14201.34 | 15563.98 | 0.91 |  | 5529.36 | 7110.12 | 0.78 |
| 2005 | 100.84 | 60.22 | 1.67 |  | 508.90 | 271.18 | 1.88 |  | 2112.51 | 1580.65 | 1.34 |  | 14055.78 | 15371.03 | 0.91 |  | 5346.52 | 6972.15 | 0.77 |
| 2006 | 96.95 | 58.72 | 1.65 |  | 484.09 | 257.37 | 1.88 |  | 1993.11 | 1507.19 | 1.32 |  | 13610.28 | 14857.77 | 0.92 |  | 5268.97 | 6914.02 | 0.76 |
| 2007 | 94.96 | 58.13 | 1.63 |  | 468.19 | 248.51 | 1.88 |  | 1928.05 | 1461.13 | 1.32 |  | 13435.74 | 14555.25 | 0.92 |  | 5169.22 | 6912.35 | 0.75 |
| 2008 | 94.23 | 57.44 | 1.64 |  | 459.38 | 240.98 | 1.91 |  | 1903.05 | 1429.90 | 1.33 |  | 13409.12 | 14438.70 | 0.93 |  | 5075.46 | 6958.41 | 0.73 |
| 2009 | 91.77 | 56.84 | 1.61 |  | 440.64 | 231.75 | 1.90 |  | 1852.58 | 1398.21 | 1.32 |  | 13294.09 | 14265.12 | 0.93 |  | 4955.42 | 6923.97 | 0.72 |
| 2010 | 90.90 | 56.40 | 1.61 |  | 430.15 | 225.11 | 1.91 |  | 1835.32 | 1375.63 | 1.33 |  | 13238.56 | 14117.25 | 0.94 |  | 4866.50 | 6830.93 | 0.71 |
| 2011 | 88.19 | 55.56 | 1.59 |  | 413.99 | 218.45 | 1.90 |  | 1764.91 | 1326.08 | 1.33 |  | 12966.55 | 13748.12 | 0.94 |  | 4696.53 | 6655.11 | 0.71 |
| 2012 | 85.72 | 54.84 | 1.56 |  | 400.81 | 213.12 | 1.88 |  | 1692.88 | 1277.07 | 1.33 |  | 12775.82 | 13359.71 | 0.96 |  | 4603.86 | 6469.58 | 0.71 |
| 2013 | 85.36 | 56.23 | 1.52 |  | 389.73 | 211.28 | 1.84 |  | 1614.52 | 1229.15 | 1.31 |  | 12549.88 | 13128.78 | 0.96 |  | 4543.38 | 6324.79 | 0.72 |
| 2014 | 85.85 | 56.62 | 1.52 |  | 381.71 | 211.60 | 1.80 |  | 1550.15 | 1178.56 | 1.32 |  | 12239.70 | 12923.17 | 0.95 |  | 4576.07 | 6209.18 | 0.74 |
| 2015 | 87.11 | 57.41 | 1.52 |  | 383.61 | 213.59 | 1.80 |  | 1531.85 | 1161.01 | 1.32 |  | 12113.72 | 12779.03 | 0.95 |  | 4578.92 | 6119.74 | 0.75 |
| 2016 | 90.23 | 57.34 | 1.57 |  | 391.79 | 212.27 | 1.85 |  | 1528.97 | 1129.72 | 1.35 |  | 11961.23 | 12487.22 | 0.96 |  | 4588.11 | 6047.79 | 0.76 |
| 2017 | 95.15 | 57.11 | 1.67 |  | 399.22 | 209.74 | 1.90 |  | 1527.68 | 1102.32 | 1.39 |  | 11806.01 | 12324.33 | 0.96 |  | 4654.54 | 6046.01 | 0.77 |
| 2018 | 96.60 | 56.41 | 1.71 |  | 389.18 | 203.63 | 1.91 |  | 1500.54 | 1088.96 | 1.38 |  | 11723.33 | 12340.83 | 0.95 |  | 4671.92 | 6070.86 | 0.77 |
| 2019 | 97.91 | 55.24 | 1.77 |  | 380.78 | 197.08 | 1.93 |  | 1489.25 | 1077.66 | 1.38 |  | 11812.39 | 12336.05 | 0.96 |  | 4719.43 | 6107.45 | 0.77 |
| High SDI |  |  |  |  |  |  |  |  |  |  |  |  |  |  |  |  |  |  |  |
| 1990 | 89.80 | 66.17 | 1.36 |  | 517.43 | 289.62 | 1.79 |  | 2101.43 | 1493.97 | 1.41 |  | 11491.00 | 13893.14 | 0.83 |  | 4344.47 | 6841.80 | 0.63 |
| 1991 | 79.15 | 61.63 | 1.28 |  | 470.46 | 272.74 | 1.72 |  | 2011.89 | 1448.91 | 1.39 |  | 11289.42 | 13634.02 | 0.83 |  | 4371.64 | 6761.77 | 0.65 |
| 1992 | 72.26 | 58.00 | 1.25 |  | 431.55 | 256.63 | 1.68 |  | 1935.05 | 1402.39 | 1.38 |  | 11167.99 | 13419.06 | 0.83 |  | 4408.96 | 6713.58 | 0.66 |
| 1993 | 68.17 | 56.07 | 1.22 |  | 407.22 | 247.38 | 1.65 |  | 1888.61 | 1379.62 | 1.37 |  | 11222.64 | 13454.76 | 0.83 |  | 4487.14 | 6780.49 | 0.66 |
| 1994 | 65.43 | 54.34 | 1.20 |  | 385.56 | 237.83 | 1.62 |  | 1820.61 | 1334.37 | 1.36 |  | 11089.13 | 13237.78 | 0.84 |  | 4488.30 | 6761.82 | 0.66 |
| 1995 | 63.68 | 53.75 | 1.18 |  | 373.42 | 232.09 | 1.61 |  | 1761.83 | 1292.00 | 1.36 |  | 10993.97 | 13078.47 | 0.84 |  | 4467.94 | 6725.74 | 0.66 |
| 1996 | 61.73 | 52.87 | 1.17 |  | 357.82 | 224.17 | 1.60 |  | 1673.95 | 1228.95 | 1.36 |  | 10697.72 | 12753.99 | 0.84 |  | 4361.94 | 6640.64 | 0.66 |
| 1997 | 60.94 | 52.39 | 1.16 |  | 343.97 | 216.85 | 1.59 |  | 1590.00 | 1171.16 | 1.36 |  | 10372.94 | 12403.23 | 0.84 |  | 4273.13 | 6541.00 | 0.65 |
| 1998 | 60.39 | 52.13 | 1.16 |  | 334.89 | 210.98 | 1.59 |  | 1528.96 | 1122.00 | 1.36 |  | 10156.61 | 12136.94 | 0.84 |  | 4182.15 | 6449.37 | 0.65 |
| 1999 | 60.24 | 52.27 | 1.15 |  | 328.27 | 208.08 | 1.58 |  | 1469.36 | 1077.88 | 1.36 |  | 9963.38 | 11929.45 | 0.84 |  | 4119.73 | 6399.14 | 0.64 |
| 2000 | 60.01 | 52.33 | 1.15 |  | 321.58 | 205.41 | 1.57 |  | 1394.03 | 1019.45 | 1.37 |  | 9598.28 | 11486.28 | 0.84 |  | 4004.81 | 6205.68 | 0.65 |
| 2001 | 59.92 | 53.38 | 1.12 |  | 319.26 | 205.08 | 1.56 |  | 1325.01 | 967.07 | 1.37 |  | 9187.40 | 11027.60 | 0.83 |  | 3880.54 | 5990.60 | 0.65 |
| 2002 | 61.82 | 55.29 | 1.12 |  | 322.66 | 204.80 | 1.58 |  | 1266.44 | 919.63 | 1.38 |  | 8751.22 | 10579.24 | 0.83 |  | 3704.84 | 5760.88 | 0.64 |
| 2003 | 64.99 | 58.15 | 1.12 |  | 329.26 | 205.67 | 1.60 |  | 1209.57 | 875.14 | 1.38 |  | 8239.05 | 10034.79 | 0.82 |  | 3532.49 | 5528.11 | 0.64 |
| 2004 | 68.39 | 60.88 | 1.12 |  | 332.67 | 204.87 | 1.62 |  | 1141.54 | 821.42 | 1.39 |  | 7646.11 | 9333.00 | 0.82 |  | 3349.07 | 5293.44 | 0.63 |
| 2005 | 72.52 | 64.21 | 1.13 |  | 339.36 | 206.11 | 1.65 |  | 1097.76 | 783.73 | 1.40 |  | 7339.47 | 8925.37 | 0.82 |  | 3283.09 | 5213.85 | 0.63 |
| 2006 | 74.67 | 65.27 | 1.14 |  | 336.49 | 202.76 | 1.66 |  | 1048.77 | 742.17 | 1.41 |  | 7040.84 | 8534.14 | 0.83 |  | 3201.21 | 5094.42 | 0.63 |
| 2007 | 77.24 | 65.86 | 1.17 |  | 333.71 | 199.65 | 1.67 |  | 1005.81 | 704.80 | 1.43 |  | 6803.05 | 8215.18 | 0.83 |  | 3115.07 | 4952.92 | 0.63 |
| 2008 | 80.79 | 66.95 | 1.21 |  | 331.93 | 198.63 | 1.67 |  | 972.31 | 675.88 | 1.44 |  | 6613.94 | 7952.61 | 0.83 |  | 3040.77 | 4810.05 | 0.63 |
| 2009 | 83.38 | 67.76 | 1.23 |  | 328.89 | 197.16 | 1.67 |  | 938.57 | 646.77 | 1.45 |  | 6379.48 | 7624.19 | 0.84 |  | 2945.31 | 4558.67 | 0.65 |
| 2010 | 84.62 | 67.81 | 1.25 |  | 323.92 | 194.63 | 1.66 |  | 910.99 | 622.34 | 1.46 |  | 6193.22 | 7351.14 | 0.84 |  | 2927.17 | 4442.36 | 0.66 |
| 2011 | 84.23 | 67.23 | 1.25 |  | 317.27 | 192.35 | 1.65 |  | 876.08 | 601.42 | 1.46 |  | 6035.52 | 7175.02 | 0.84 |  | 2926.53 | 4413.86 | 0.66 |
| 2012 | 83.91 | 66.31 | 1.27 |  | 307.30 | 188.24 | 1.63 |  | 831.77 | 574.83 | 1.45 |  | 5841.42 | 6971.23 | 0.84 |  | 2898.69 | 4364.74 | 0.66 |
| 2013 | 85.41 | 66.63 | 1.28 |  | 301.30 | 185.16 | 1.63 |  | 796.58 | 553.49 | 1.44 |  | 5614.48 | 6727.53 | 0.83 |  | 2820.26 | 4273.00 | 0.66 |
| 2014 | 86.94 | 67.78 | 1.28 |  | 295.76 | 183.07 | 1.62 |  | 766.51 | 533.54 | 1.44 |  | 5367.13 | 6452.73 | 0.83 |  | 2700.95 | 4186.51 | 0.65 |
| 2015 | 91.53 | 69.69 | 1.31 |  | 298.33 | 182.83 | 1.63 |  | 758.73 | 527.15 | 1.44 |  | 5237.48 | 6309.95 | 0.83 |  | 2614.13 | 4119.17 | 0.63 |
| 2016 | 101.94 | 76.45 | 1.33 |  | 318.81 | 191.29 | 1.67 |  | 779.74 | 533.48 | 1.46 |  | 5215.12 | 6127.75 | 0.85 |  | 2580.11 | 4016.54 | 0.64 |
| 2017 | 115.91 | 85.23 | 1.36 |  | 351.97 | 202.73 | 1.74 |  | 817.35 | 541.63 | 1.51 |  | 5278.69 | 5979.17 | 0.88 |  | 2564.39 | 3914.22 | 0.66 |
| 2018 | 119.55 | 87.13 | 1.37 |  | 361.29 | 204.02 | 1.77 |  | 812.34 | 540.54 | 1.50 |  | 5221.66 | 5934.84 | 0.88 |  | 2491.41 | 3856.43 | 0.65 |
| 2019 | 122.84 | 88.18 | 1.39 |  | 371.12 | 207.33 | 1.79 |  | 801.89 | 539.60 | 1.49 |  | 5142.88 | 5906.71 | 0.87 |  | 2416.39 | 3793.04 | 0.64 |

| **Supplemental table 8. DALY rates of type 2 diabetes attributable to LPA by sex and age groups (1990-2019)** | | | | | | | | | | | | | | | | | | | |
| --- | --- | --- | --- | --- | --- | --- | --- | --- | --- | --- | --- | --- | --- | --- | --- | --- | --- | --- | --- |
|  | **25-44 years** | | |  | **45-59 years** | | |  | **60-74 years** | | |  | **75-94 years** | | |  | **95+ years** | | |
|  | Male | Female | M/F ratio |  | Male | Female | M/F ratio |  | Male | Female | M/F ratio |  | Male | Female | M/F ratio |  | Male | Female | M/F ratio |
| Global |  |  |  |  |  |  |  |  |  |  |  |  |  |  |  |  |  |  |  |
| 1990 | 29.65 | 44.44 | 0.67 |  | 165.80 | 225.28 | 0.74 |  | 575.73 | 693.98 | 0.83 |  | 1,716.35 | 1,828.49 | 0.94 |  | 450.37 | 515.16 | 0.87 |
| 1991 | 28.82 | 42.50 | 0.68 |  | 165.15 | 217.92 | 0.76 |  | 589.16 | 699.22 | 0.84 |  | 1,738.81 | 1,841.57 | 0.94 |  | 457.79 | 516.54 | 0.89 |
| 1992 | 28.51 | 41.49 | 0.69 |  | 165.10 | 214.32 | 0.77 |  | 603.32 | 708.10 | 0.85 |  | 1,769.70 | 1,862.97 | 0.95 |  | 464.71 | 517.31 | 0.90 |
| 1993 | 28.47 | 40.75 | 0.70 |  | 166.65 | 212.63 | 0.78 |  | 618.91 | 717.10 | 0.86 |  | 1,811.82 | 1,895.72 | 0.96 |  | 476.37 | 523.91 | 0.91 |
| 1994 | 28.76 | 40.57 | 0.71 |  | 168.37 | 212.03 | 0.79 |  | 631.32 | 723.80 | 0.87 |  | 1,844.58 | 1,915.96 | 0.96 |  | 485.41 | 525.30 | 0.92 |
| 1995 | 28.97 | 40.48 | 0.72 |  | 170.55 | 212.20 | 0.80 |  | 640.08 | 727.40 | 0.88 |  | 1,880.49 | 1,939.34 | 0.97 |  | 494.20 | 528.14 | 0.94 |
| 1996 | 29.07 | 40.62 | 0.72 |  | 171.89 | 214.21 | 0.80 |  | 644.73 | 732.46 | 0.88 |  | 1,917.99 | 1,966.57 | 0.98 |  | 500.73 | 530.75 | 0.94 |
| 1997 | 29.38 | 41.43 | 0.71 |  | 172.97 | 217.82 | 0.79 |  | 648.17 | 740.61 | 0.88 |  | 1,957.79 | 1,993.79 | 0.98 |  | 509.46 | 537.06 | 0.95 |
| 1998 | 29.85 | 42.52 | 0.70 |  | 174.41 | 221.27 | 0.79 |  | 650.37 | 748.78 | 0.87 |  | 2,001.27 | 2,026.92 | 0.99 |  | 516.94 | 541.05 | 0.96 |
| 1999 | 30.46 | 43.69 | 0.70 |  | 176.42 | 225.58 | 0.78 |  | 649.56 | 755.69 | 0.86 |  | 2,033.57 | 2,062.32 | 0.99 |  | 524.25 | 549.60 | 0.95 |
| 2000 | 30.97 | 44.52 | 0.70 |  | 178.55 | 228.49 | 0.78 |  | 654.67 | 760.37 | 0.86 |  | 2,060.81 | 2,087.73 | 0.99 |  | 530.83 | 557.65 | 0.95 |
| 2001 | 31.21 | 44.98 | 0.69 |  | 179.94 | 229.63 | 0.78 |  | 661.57 | 767.44 | 0.86 |  | 2,089.64 | 2,118.59 | 0.99 |  | 540.89 | 569.72 | 0.95 |
| 2002 | 31.62 | 45.61 | 0.69 |  | 182.06 | 231.54 | 0.79 |  | 671.78 | 780.03 | 0.86 |  | 2,128.03 | 2,159.30 | 0.99 |  | 553.21 | 583.08 | 0.95 |
| 2003 | 32.16 | 46.26 | 0.70 |  | 185.03 | 233.74 | 0.79 |  | 683.37 | 791.40 | 0.86 |  | 2,149.03 | 2,183.56 | 0.98 |  | 562.37 | 595.45 | 0.94 |
| 2004 | 32.72 | 46.86 | 0.70 |  | 186.87 | 234.62 | 0.80 |  | 689.55 | 795.55 | 0.87 |  | 2,147.14 | 2,181.89 | 0.98 |  | 564.34 | 600.72 | 0.94 |
| 2005 | 33.22 | 47.47 | 0.70 |  | 189.68 | 235.80 | 0.80 |  | 699.32 | 804.63 | 0.87 |  | 2,164.83 | 2,202.85 | 0.98 |  | 570.01 | 611.87 | 0.93 |
| 2006 | 33.64 | 47.90 | 0.70 |  | 191.54 | 236.00 | 0.81 |  | 705.02 | 808.57 | 0.87 |  | 2,173.56 | 2,212.51 | 0.98 |  | 576.41 | 619.44 | 0.93 |
| 2007 | 34.28 | 48.50 | 0.71 |  | 194.34 | 237.38 | 0.82 |  | 710.27 | 810.39 | 0.88 |  | 2,191.51 | 2,228.11 | 0.98 |  | 586.14 | 630.29 | 0.93 |
| 2008 | 35.21 | 49.51 | 0.71 |  | 197.83 | 239.79 | 0.83 |  | 720.13 | 814.14 | 0.88 |  | 2,218.26 | 2,247.82 | 0.99 |  | 596.24 | 638.44 | 0.93 |
| 2009 | 36.13 | 50.54 | 0.71 |  | 202.10 | 242.89 | 0.83 |  | 726.81 | 816.81 | 0.89 |  | 2,217.46 | 2,236.03 | 0.99 |  | 601.39 | 636.08 | 0.95 |
| 2010 | 36.99 | 51.35 | 0.72 |  | 205.04 | 245.26 | 0.84 |  | 732.82 | 819.89 | 0.89 |  | 2,211.92 | 2,222.69 | 1.00 |  | 608.52 | 636.22 | 0.96 |
| 2011 | 37.80 | 52.46 | 0.72 |  | 207.52 | 247.61 | 0.84 |  | 732.75 | 821.74 | 0.89 |  | 2,194.58 | 2,218.06 | 0.99 |  | 607.89 | 634.57 | 0.96 |
| 2012 | 38.84 | 54.18 | 0.72 |  | 209.82 | 251.03 | 0.84 |  | 727.58 | 822.36 | 0.88 |  | 2,177.77 | 2,210.49 | 0.99 |  | 605.36 | 631.74 | 0.96 |
| 2013 | 40.25 | 56.44 | 0.71 |  | 212.91 | 255.80 | 0.83 |  | 719.93 | 814.30 | 0.88 |  | 2,200.42 | 2,214.20 | 0.99 |  | 609.23 | 628.18 | 0.97 |
| 2014 | 41.73 | 58.64 | 0.71 |  | 217.10 | 262.83 | 0.83 |  | 715.44 | 805.46 | 0.89 |  | 2,210.25 | 2,218.64 | 1.00 |  | 609.35 | 625.61 | 0.97 |
| 2015 | 43.28 | 60.46 | 0.72 |  | 222.89 | 268.88 | 0.83 |  | 720.16 | 807.97 | 0.89 |  | 2,184.99 | 2,213.91 | 0.99 |  | 603.35 | 622.77 | 0.97 |
| 2016 | 46.01 | 62.58 | 0.74 |  | 232.59 | 273.78 | 0.85 |  | 740.89 | 812.40 | 0.91 |  | 2,192.78 | 2,205.15 | 0.99 |  | 608.62 | 620.45 | 0.98 |
| 2017 | 49.45 | 64.87 | 0.76 |  | 243.22 | 278.43 | 0.87 |  | 761.55 | 816.27 | 0.93 |  | 2,221.16 | 2,204.84 | 1.01 |  | 616.27 | 617.59 | 1.00 |
| 2018 | 50.87 | 64.48 | 0.79 |  | 242.06 | 270.31 | 0.90 |  | 748.13 | 797.59 | 0.94 |  | 2,239.30 | 2,215.93 | 1.01 |  | 614.67 | 616.58 | 1.00 |
| 2019 | 52.41 | 63.26 | 0.83 |  | 240.61 | 260.39 | 0.92 |  | 730.01 | 773.66 | 0.94 |  | 2,252.14 | 2,225.08 | 1.01 |  | 613.04 | 615.33 | 1.00 |
| Low SDI |  |  |  |  |  |  |  |  |  |  |  |  |  |  |  |  |  |  |  |
| 1990 | 25.85 | 37.68 | 0.69 |  | 201.63 | 220.44 | 0.91 |  | 695.61 | 687.27 | 1.01 |  | 2,013.21 | 1,818.70 | 1.11 |  | 543.48 | 442.55 | 1.23 |
| 1991 | 25.53 | 35.99 | 0.71 |  | 202.25 | 208.57 | 0.97 |  | 717.05 | 680.62 | 1.05 |  | 2,037.64 | 1,840.24 | 1.11 |  | 553.11 | 449.29 | 1.23 |
| 1992 | 25.55 | 35.06 | 0.73 |  | 204.11 | 202.61 | 1.01 |  | 738.48 | 679.99 | 1.09 |  | 2,064.52 | 1,869.08 | 1.10 |  | 562.51 | 458.77 | 1.23 |
| 1993 | 25.70 | 34.66 | 0.74 |  | 206.54 | 199.69 | 1.03 |  | 759.24 | 681.36 | 1.11 |  | 2,088.68 | 1,897.71 | 1.10 |  | 573.17 | 465.50 | 1.23 |
| 1994 | 25.90 | 34.43 | 0.75 |  | 208.95 | 199.00 | 1.05 |  | 776.15 | 684.87 | 1.13 |  | 2,107.47 | 1,916.58 | 1.10 |  | 583.97 | 473.90 | 1.23 |
| 1995 | 26.20 | 34.86 | 0.75 |  | 212.05 | 199.21 | 1.06 |  | 786.96 | 689.42 | 1.14 |  | 2,130.63 | 1,938.59 | 1.10 |  | 587.13 | 485.73 | 1.21 |
| 1996 | 26.55 | 35.65 | 0.74 |  | 213.93 | 202.92 | 1.05 |  | 792.18 | 698.86 | 1.13 |  | 2,164.94 | 1,968.70 | 1.10 |  | 590.89 | 497.82 | 1.19 |
| 1997 | 26.90 | 36.85 | 0.73 |  | 215.42 | 209.99 | 1.03 |  | 797.47 | 716.03 | 1.11 |  | 2,213.68 | 2,017.89 | 1.10 |  | 596.26 | 505.86 | 1.18 |
| 1998 | 27.24 | 38.47 | 0.71 |  | 216.98 | 219.25 | 0.99 |  | 798.54 | 739.99 | 1.08 |  | 2,250.03 | 2,066.13 | 1.09 |  | 598.62 | 517.77 | 1.16 |
| 1999 | 27.54 | 40.10 | 0.69 |  | 218.66 | 229.09 | 0.95 |  | 793.58 | 758.13 | 1.05 |  | 2,265.33 | 2,096.68 | 1.08 |  | 599.33 | 526.91 | 1.14 |
| 2000 | 27.84 | 41.18 | 0.68 |  | 220.34 | 235.90 | 0.93 |  | 802.29 | 775.56 | 1.03 |  | 2,303.55 | 2,148.76 | 1.07 |  | 603.89 | 535.22 | 1.13 |
| 2001 | 28.12 | 41.99 | 0.67 |  | 223.18 | 241.67 | 0.92 |  | 814.66 | 798.24 | 1.02 |  | 2,327.70 | 2,203.86 | 1.06 |  | 610.54 | 549.23 | 1.11 |
| 2002 | 28.36 | 42.63 | 0.67 |  | 225.08 | 245.71 | 0.92 |  | 823.25 | 816.83 | 1.01 |  | 2,364.87 | 2,262.18 | 1.05 |  | 621.59 | 562.33 | 1.11 |
| 2003 | 28.76 | 43.33 | 0.66 |  | 227.42 | 250.37 | 0.91 |  | 839.65 | 832.93 | 1.01 |  | 2,438.30 | 2,301.41 | 1.06 |  | 645.76 | 579.29 | 1.11 |
| 2004 | 29.15 | 43.90 | 0.66 |  | 228.78 | 253.78 | 0.90 |  | 845.49 | 843.18 | 1.00 |  | 2,431.10 | 2,337.68 | 1.04 |  | 657.89 | 594.31 | 1.11 |
| 2005 | 29.54 | 44.54 | 0.66 |  | 230.84 | 255.93 | 0.90 |  | 851.77 | 858.44 | 0.99 |  | 2,456.19 | 2,383.84 | 1.03 |  | 673.34 | 608.38 | 1.11 |
| 2006 | 29.70 | 44.67 | 0.66 |  | 230.31 | 255.22 | 0.90 |  | 854.16 | 864.30 | 0.99 |  | 2,465.86 | 2,413.44 | 1.02 |  | 684.58 | 617.89 | 1.11 |
| 2007 | 30.06 | 44.86 | 0.67 |  | 231.94 | 254.39 | 0.91 |  | 862.49 | 865.08 | 1.00 |  | 2,482.41 | 2,419.39 | 1.03 |  | 698.56 | 627.06 | 1.11 |
| 2008 | 30.47 | 45.15 | 0.67 |  | 232.85 | 254.60 | 0.91 |  | 863.34 | 869.00 | 0.99 |  | 2,490.89 | 2,438.23 | 1.02 |  | 708.77 | 636.99 | 1.11 |
| 2009 | 30.88 | 45.42 | 0.68 |  | 235.03 | 255.24 | 0.92 |  | 867.28 | 876.14 | 0.99 |  | 2,465.48 | 2,419.24 | 1.02 |  | 717.33 | 645.97 | 1.11 |
| 2010 | 31.32 | 45.84 | 0.68 |  | 236.89 | 257.35 | 0.92 |  | 867.35 | 878.20 | 0.99 |  | 2,445.60 | 2,403.21 | 1.02 |  | 733.76 | 652.02 | 1.13 |
| 2011 | 31.74 | 46.64 | 0.68 |  | 238.92 | 260.88 | 0.92 |  | 868.94 | 889.17 | 0.98 |  | 2,509.99 | 2,420.48 | 1.04 |  | 808.54 | 656.41 | 1.23 |
| 2012 | 32.16 | 47.67 | 0.67 |  | 240.54 | 267.41 | 0.90 |  | 871.80 | 912.46 | 0.96 |  | 2,497.15 | 2,451.44 | 1.02 |  | 779.51 | 655.04 | 1.19 |
| 2013 | 32.96 | 48.87 | 0.67 |  | 243.36 | 272.96 | 0.89 |  | 870.51 | 913.72 | 0.95 |  | 2,536.35 | 2,491.68 | 1.02 |  | 764.57 | 659.85 | 1.16 |
| 2014 | 33.72 | 50.08 | 0.67 |  | 246.37 | 282.22 | 0.87 |  | 861.91 | 917.01 | 0.94 |  | 2,503.27 | 2,527.18 | 0.99 |  | 744.11 | 659.93 | 1.13 |
| 2015 | 34.24 | 50.69 | 0.68 |  | 249.16 | 287.61 | 0.87 |  | 863.77 | 920.22 | 0.94 |  | 2,486.06 | 2,521.49 | 0.99 |  | 726.75 | 651.44 | 1.12 |
| 2016 | 34.73 | 50.79 | 0.68 |  | 249.16 | 288.37 | 0.86 |  | 863.77 | 922.09 | 0.94 |  | 2,450.52 | 2,514.96 | 0.97 |  | 702.21 | 644.01 | 1.09 |
| 2017 | 35.54 | 51.00 | 0.70 |  | 250.40 | 289.54 | 0.86 |  | 864.08 | 922.46 | 0.94 |  | 2,437.37 | 2,522.71 | 0.97 |  | 683.31 | 639.93 | 1.07 |
| 2018 | 35.75 | 49.75 | 0.72 |  | 247.65 | 279.34 | 0.89 |  | 835.37 | 881.96 | 0.95 |  | 2,443.24 | 2,538.09 | 0.96 |  | 680.91 | 642.39 | 1.06 |
| 2019 | 36.09 | 48.21 | 0.75 |  | 243.26 | 264.68 | 0.92 |  | 791.94 | 823.28 | 0.96 |  | 2,409.23 | 2,507.13 | 0.96 |  | 668.57 | 633.31 | 1.06 |
| Low-middle SDI |  |  |  |  |  |  |  |  |  |  |  |  |  |  |  |  |  |  |  |
| 1990 | 28.54 | 45.05 | 0.63 |  | 173.17 | 241.15 | 0.72 |  | 674.74 | 771.61 | 0.87 |  | 2,344.44 | 2,115.14 | 1.11 |  | 610.16 | 531.09 | 1.15 |
| 1991 | 28.84 | 42.88 | 0.67 |  | 177.78 | 225.43 | 0.79 |  | 709.61 | 775.21 | 0.92 |  | 2,357.94 | 2,155.00 | 1.09 |  | 613.48 | 534.32 | 1.15 |
| 1992 | 29.14 | 41.60 | 0.70 |  | 182.26 | 217.99 | 0.84 |  | 744.22 | 786.39 | 0.95 |  | 2,394.70 | 2,212.17 | 1.08 |  | 620.34 | 541.96 | 1.14 |
| 1993 | 29.57 | 40.72 | 0.73 |  | 187.86 | 213.05 | 0.88 |  | 776.32 | 792.99 | 0.98 |  | 2,448.06 | 2,264.50 | 1.08 |  | 635.03 | 551.15 | 1.15 |
| 1994 | 30.12 | 40.29 | 0.75 |  | 191.87 | 212.30 | 0.90 |  | 798.23 | 797.44 | 1.00 |  | 2,481.84 | 2,297.86 | 1.08 |  | 648.78 | 555.33 | 1.17 |
| 1995 | 30.45 | 40.12 | 0.76 |  | 195.19 | 213.44 | 0.91 |  | 808.56 | 801.70 | 1.01 |  | 2,519.13 | 2,337.56 | 1.08 |  | 666.44 | 567.94 | 1.17 |
| 1996 | 30.46 | 40.58 | 0.75 |  | 196.20 | 219.32 | 0.89 |  | 811.44 | 810.43 | 1.00 |  | 2,561.91 | 2,376.34 | 1.08 |  | 674.19 | 581.90 | 1.16 |
| 1997 | 30.50 | 41.81 | 0.73 |  | 197.44 | 229.42 | 0.86 |  | 817.28 | 838.17 | 0.98 |  | 2,625.94 | 2,434.63 | 1.08 |  | 698.33 | 594.32 | 1.18 |
| 1998 | 30.56 | 43.53 | 0.70 |  | 196.90 | 239.84 | 0.82 |  | 808.70 | 861.47 | 0.94 |  | 2,675.08 | 2,511.75 | 1.07 |  | 720.95 | 610.52 | 1.18 |
| 1999 | 30.98 | 45.16 | 0.69 |  | 197.98 | 251.01 | 0.79 |  | 788.30 | 873.37 | 0.90 |  | 2,696.71 | 2,579.35 | 1.05 |  | 737.01 | 622.59 | 1.18 |
| 2000 | 31.34 | 45.93 | 0.68 |  | 200.98 | 258.74 | 0.78 |  | 793.83 | 887.80 | 0.89 |  | 2,755.31 | 2,638.43 | 1.04 |  | 749.49 | 633.60 | 1.18 |
| 2001 | 31.79 | 46.19 | 0.69 |  | 203.01 | 262.41 | 0.77 |  | 809.15 | 908.98 | 0.89 |  | 2,812.29 | 2,718.15 | 1.03 |  | 769.81 | 651.17 | 1.18 |
| 2002 | 32.09 | 46.25 | 0.69 |  | 205.70 | 265.64 | 0.77 |  | 823.23 | 931.77 | 0.88 |  | 2,906.03 | 2,809.32 | 1.03 |  | 807.54 | 681.23 | 1.19 |
| 2003 | 32.40 | 46.19 | 0.70 |  | 209.85 | 268.15 | 0.78 |  | 836.77 | 939.68 | 0.89 |  | 2,976.20 | 2,843.96 | 1.05 |  | 849.83 | 701.10 | 1.21 |
| 2004 | 32.99 | 46.19 | 0.71 |  | 212.48 | 269.33 | 0.79 |  | 839.81 | 934.43 | 0.90 |  | 2,987.31 | 2,853.09 | 1.05 |  | 866.41 | 719.15 | 1.20 |
| 2005 | 33.44 | 46.59 | 0.72 |  | 217.09 | 270.91 | 0.80 |  | 857.60 | 954.87 | 0.90 |  | 3,027.52 | 2,915.85 | 1.04 |  | 882.47 | 741.74 | 1.19 |
| 2006 | 33.95 | 46.73 | 0.73 |  | 220.12 | 270.40 | 0.81 |  | 873.71 | 972.26 | 0.90 |  | 3,082.36 | 2,962.07 | 1.04 |  | 912.47 | 759.22 | 1.20 |
| 2007 | 34.51 | 47.21 | 0.73 |  | 223.10 | 271.84 | 0.82 |  | 881.54 | 981.83 | 0.90 |  | 3,084.08 | 2,993.50 | 1.03 |  | 931.20 | 790.61 | 1.18 |
| 2008 | 35.10 | 47.85 | 0.73 |  | 226.26 | 272.76 | 0.83 |  | 894.29 | 985.04 | 0.91 |  | 3,083.05 | 3,023.01 | 1.02 |  | 939.24 | 826.61 | 1.14 |
| 2009 | 35.58 | 48.30 | 0.74 |  | 230.09 | 273.64 | 0.84 |  | 898.63 | 982.77 | 0.91 |  | 3,009.11 | 2,935.10 | 1.03 |  | 937.78 | 842.67 | 1.11 |
| 2010 | 36.20 | 48.77 | 0.74 |  | 234.09 | 277.11 | 0.84 |  | 907.30 | 992.28 | 0.91 |  | 2,961.75 | 2,879.27 | 1.03 |  | 942.61 | 860.16 | 1.10 |
| 2011 | 37.09 | 49.87 | 0.74 |  | 238.97 | 283.45 | 0.84 |  | 914.85 | 1,018.09 | 0.90 |  | 2,870.72 | 2,893.65 | 0.99 |  | 902.48 | 862.71 | 1.05 |
| 2012 | 38.23 | 51.49 | 0.74 |  | 244.53 | 293.04 | 0.83 |  | 922.09 | 1,049.79 | 0.88 |  | 2,843.53 | 2,945.15 | 0.97 |  | 879.87 | 862.05 | 1.02 |
| 2013 | 39.52 | 53.55 | 0.74 |  | 248.45 | 304.42 | 0.82 |  | 915.92 | 1,044.21 | 0.88 |  | 3,054.57 | 3,060.30 | 1.00 |  | 919.10 | 878.79 | 1.05 |
| 2014 | 40.72 | 55.53 | 0.73 |  | 251.80 | 319.30 | 0.79 |  | 910.85 | 1,034.94 | 0.88 |  | 3,241.64 | 3,171.63 | 1.02 |  | 958.25 | 897.18 | 1.07 |
| 2015 | 41.71 | 56.89 | 0.73 |  | 257.92 | 330.49 | 0.78 |  | 921.89 | 1,054.42 | 0.87 |  | 3,161.65 | 3,169.59 | 1.00 |  | 941.11 | 903.85 | 1.04 |
| 2016 | 42.28 | 57.57 | 0.73 |  | 260.31 | 329.81 | 0.79 |  | 942.05 | 1,061.93 | 0.89 |  | 3,104.60 | 3,154.72 | 0.98 |  | 927.25 | 899.62 | 1.03 |
| 2017 | 42.75 | 58.14 | 0.74 |  | 260.42 | 327.08 | 0.80 |  | 954.38 | 1,063.89 | 0.90 |  | 3,152.76 | 3,172.06 | 0.99 |  | 930.69 | 905.06 | 1.03 |
| 2018 | 43.71 | 56.57 | 0.77 |  | 253.39 | 305.86 | 0.83 |  | 916.46 | 1,014.90 | 0.90 |  | 3,254.87 | 3,220.44 | 1.01 |  | 939.16 | 912.33 | 1.03 |
| 2019 | 45.24 | 54.07 | 0.84 |  | 244.67 | 278.18 | 0.88 |  | 861.17 | 947.48 | 0.91 |  | 3,284.36 | 3,233.48 | 1.02 |  | 941.68 | 913.75 | 1.03 |
| Middle SDI |  |  |  |  |  |  |  |  |  |  |  |  |  |  |  |  |  |  |  |
| 1990 | 32.74 | 48.76 | 0.67 |  | 170.31 | 250.07 | 0.68 |  | 581.01 | 772.41 | 0.75 |  | 1,883.83 | 1,959.50 | 0.96 |  | 523.68 | 484.88 | 1.08 |
| 1991 | 32.50 | 46.54 | 0.70 |  | 173.14 | 242.83 | 0.71 |  | 603.12 | 783.41 | 0.77 |  | 1,920.04 | 1,993.47 | 0.96 |  | 535.38 | 500.84 | 1.07 |
| 1992 | 32.69 | 45.79 | 0.71 |  | 176.01 | 241.75 | 0.73 |  | 623.60 | 802.91 | 0.78 |  | 1,960.83 | 2,047.36 | 0.96 |  | 551.14 | 521.43 | 1.06 |
| 1993 | 32.81 | 44.58 | 0.74 |  | 179.55 | 240.36 | 0.75 |  | 643.65 | 815.69 | 0.79 |  | 2,005.60 | 2,087.59 | 0.96 |  | 567.65 | 532.65 | 1.07 |
| 1994 | 33.35 | 44.55 | 0.75 |  | 183.20 | 240.66 | 0.76 |  | 660.12 | 826.54 | 0.80 |  | 2,046.41 | 2,122.13 | 0.96 |  | 581.73 | 542.57 | 1.07 |
| 1995 | 33.59 | 44.08 | 0.76 |  | 186.97 | 240.23 | 0.78 |  | 671.54 | 832.22 | 0.81 |  | 2,087.50 | 2,154.78 | 0.97 |  | 596.50 | 548.75 | 1.09 |
| 1996 | 33.74 | 43.97 | 0.77 |  | 189.75 | 243.25 | 0.78 |  | 680.44 | 846.70 | 0.80 |  | 2,132.50 | 2,199.44 | 0.97 |  | 609.32 | 556.97 | 1.09 |
| 1997 | 34.41 | 45.11 | 0.76 |  | 191.79 | 249.50 | 0.77 |  | 687.04 | 866.60 | 0.79 |  | 2,162.79 | 2,230.09 | 0.97 |  | 611.56 | 564.18 | 1.08 |
| 1998 | 35.23 | 46.19 | 0.76 |  | 192.32 | 252.06 | 0.76 |  | 692.56 | 880.42 | 0.79 |  | 2,186.77 | 2,245.97 | 0.97 |  | 612.73 | 569.95 | 1.08 |
| 1999 | 36.03 | 47.59 | 0.76 |  | 192.39 | 254.52 | 0.76 |  | 693.00 | 890.54 | 0.78 |  | 2,198.70 | 2,260.61 | 0.97 |  | 614.92 | 575.12 | 1.07 |
| 2000 | 36.91 | 48.81 | 0.76 |  | 194.04 | 255.73 | 0.76 |  | 701.08 | 898.43 | 0.78 |  | 2,214.09 | 2,289.33 | 0.97 |  | 618.36 | 585.23 | 1.06 |
| 2001 | 37.07 | 49.11 | 0.75 |  | 194.29 | 254.05 | 0.76 |  | 706.10 | 902.74 | 0.78 |  | 2,228.28 | 2,324.35 | 0.96 |  | 623.79 | 599.02 | 1.04 |
| 2002 | 37.46 | 49.74 | 0.75 |  | 196.37 | 255.29 | 0.77 |  | 715.81 | 913.96 | 0.78 |  | 2,252.51 | 2,365.36 | 0.95 |  | 632.49 | 609.09 | 1.04 |
| 2003 | 37.64 | 50.16 | 0.75 |  | 198.61 | 256.82 | 0.77 |  | 724.86 | 923.23 | 0.79 |  | 2,266.58 | 2,390.45 | 0.95 |  | 631.79 | 617.94 | 1.02 |
| 2004 | 37.58 | 50.34 | 0.75 |  | 199.62 | 257.31 | 0.78 |  | 729.97 | 924.83 | 0.79 |  | 2,289.79 | 2,411.22 | 0.95 |  | 630.86 | 623.56 | 1.01 |
| 2005 | 37.67 | 50.37 | 0.75 |  | 203.12 | 258.73 | 0.79 |  | 740.11 | 929.57 | 0.80 |  | 2,321.27 | 2,446.57 | 0.95 |  | 622.82 | 620.11 | 1.00 |
| 2006 | 37.81 | 50.31 | 0.75 |  | 206.26 | 258.87 | 0.80 |  | 747.87 | 932.84 | 0.80 |  | 2,319.42 | 2,464.21 | 0.94 |  | 608.18 | 609.95 | 1.00 |
| 2007 | 38.14 | 50.31 | 0.76 |  | 209.15 | 258.39 | 0.81 |  | 755.76 | 931.50 | 0.81 |  | 2,340.93 | 2,480.11 | 0.94 |  | 605.84 | 609.11 | 0.99 |
| 2008 | 39.11 | 51.22 | 0.76 |  | 214.34 | 261.35 | 0.82 |  | 772.67 | 938.16 | 0.82 |  | 2,395.58 | 2,511.05 | 0.95 |  | 612.43 | 609.02 | 1.01 |
| 2009 | 40.11 | 52.30 | 0.77 |  | 220.75 | 265.89 | 0.83 |  | 785.09 | 944.62 | 0.83 |  | 2,425.11 | 2,515.41 | 0.96 |  | 620.70 | 613.73 | 1.01 |
| 2010 | 40.98 | 52.99 | 0.77 |  | 224.43 | 268.89 | 0.83 |  | 797.42 | 952.69 | 0.84 |  | 2,450.74 | 2,526.98 | 0.97 |  | 627.65 | 626.76 | 1.00 |
| 2011 | 41.87 | 53.81 | 0.78 |  | 228.78 | 270.97 | 0.84 |  | 805.38 | 953.03 | 0.85 |  | 2,465.80 | 2,537.38 | 0.97 |  | 629.46 | 632.03 | 1.00 |
| 2012 | 43.27 | 55.38 | 0.78 |  | 234.48 | 274.62 | 0.85 |  | 813.29 | 953.73 | 0.85 |  | 2,483.59 | 2,537.97 | 0.98 |  | 637.47 | 638.03 | 1.00 |
| 2013 | 45.00 | 57.39 | 0.78 |  | 241.71 | 280.08 | 0.86 |  | 818.55 | 948.02 | 0.86 |  | 2,556.17 | 2,576.95 | 0.99 |  | 659.76 | 653.56 | 1.01 |
| 2014 | 46.79 | 59.36 | 0.79 |  | 250.79 | 288.67 | 0.87 |  | 826.67 | 938.29 | 0.88 |  | 2,598.95 | 2,602.02 | 1.00 |  | 678.73 | 666.94 | 1.02 |
| 2015 | 48.80 | 61.35 | 0.80 |  | 260.92 | 296.48 | 0.88 |  | 840.67 | 938.69 | 0.90 |  | 2,592.12 | 2,596.80 | 1.00 |  | 691.47 | 667.59 | 1.04 |
| 2016 | 52.05 | 64.86 | 0.80 |  | 272.99 | 305.21 | 0.89 |  | 863.79 | 946.60 | 0.91 |  | 2,613.65 | 2,603.28 | 1.00 |  | 714.41 | 672.39 | 1.06 |
| 2017 | 56.01 | 68.49 | 0.82 |  | 283.14 | 311.96 | 0.91 |  | 881.35 | 952.76 | 0.93 |  | 2,639.12 | 2,612.42 | 1.01 |  | 736.91 | 681.60 | 1.08 |
| 2018 | 57.73 | 67.61 | 0.85 |  | 277.82 | 297.92 | 0.93 |  | 867.85 | 923.66 | 0.94 |  | 2,681.79 | 2,622.52 | 1.02 |  | 752.09 | 690.98 | 1.09 |
| 2019 | 58.91 | 65.14 | 0.90 |  | 270.74 | 280.09 | 0.97 |  | 847.04 | 882.21 | 0.96 |  | 2,709.06 | 2,610.26 | 1.04 |  | 762.05 | 692.77 | 1.10 |
| High-middle SDI |  |  |  |  |  |  |  |  |  |  |  |  |  |  |  |  |  |  |  |
| 1990 | 32.08 | 40.97 | 0.78 |  | 159.77 | 205.89 | 0.78 |  | 569.07 | 667.52 | 0.85 |  | 1,604.85 | 1,706.92 | 0.94 |  | 444.69 | 484.21 | 0.92 |
| 1991 | 31.05 | 39.84 | 0.78 |  | 157.44 | 203.34 | 0.77 |  | 569.81 | 673.73 | 0.85 |  | 1,615.61 | 1,712.43 | 0.94 |  | 453.55 | 491.93 | 0.92 |
| 1992 | 30.38 | 39.21 | 0.77 |  | 155.49 | 201.02 | 0.77 |  | 572.60 | 679.98 | 0.84 |  | 1,634.80 | 1,722.31 | 0.95 |  | 460.52 | 495.59 | 0.93 |
| 1993 | 30.01 | 39.10 | 0.77 |  | 155.62 | 201.72 | 0.77 |  | 578.16 | 689.12 | 0.84 |  | 1,665.36 | 1,747.35 | 0.95 |  | 475.55 | 511.98 | 0.93 |
| 1994 | 29.98 | 39.11 | 0.77 |  | 156.14 | 200.87 | 0.78 |  | 583.86 | 696.38 | 0.84 |  | 1,691.29 | 1,769.11 | 0.96 |  | 488.57 | 521.67 | 0.94 |
| 1995 | 29.86 | 39.13 | 0.76 |  | 157.14 | 200.45 | 0.78 |  | 588.28 | 696.00 | 0.85 |  | 1,726.34 | 1,793.01 | 0.96 |  | 503.30 | 537.51 | 0.94 |
| 1996 | 29.58 | 38.88 | 0.76 |  | 157.25 | 198.83 | 0.79 |  | 586.03 | 691.71 | 0.85 |  | 1,767.52 | 1,820.93 | 0.97 |  | 509.68 | 535.74 | 0.95 |
| 1997 | 29.24 | 38.60 | 0.76 |  | 156.50 | 195.74 | 0.80 |  | 577.17 | 681.59 | 0.85 |  | 1,816.01 | 1,847.63 | 0.98 |  | 520.93 | 553.10 | 0.94 |
| 1998 | 29.19 | 38.83 | 0.75 |  | 158.57 | 194.61 | 0.81 |  | 569.24 | 673.82 | 0.84 |  | 1,865.34 | 1,885.23 | 0.99 |  | 524.58 | 560.69 | 0.94 |
| 1999 | 29.48 | 39.05 | 0.75 |  | 162.20 | 196.00 | 0.83 |  | 565.06 | 669.96 | 0.84 |  | 1,898.37 | 1,916.24 | 0.99 |  | 525.48 | 565.80 | 0.93 |
| 2000 | 29.40 | 38.94 | 0.75 |  | 163.76 | 196.18 | 0.83 |  | 562.48 | 665.11 | 0.85 |  | 1,915.00 | 1,938.65 | 0.99 |  | 526.36 | 580.03 | 0.91 |
| 2001 | 29.10 | 38.69 | 0.75 |  | 164.25 | 195.59 | 0.84 |  | 568.93 | 669.76 | 0.85 |  | 1,975.87 | 1,979.45 | 1.00 |  | 546.47 | 607.47 | 0.90 |
| 2002 | 28.71 | 38.10 | 0.75 |  | 164.22 | 195.35 | 0.84 |  | 584.51 | 685.16 | 0.85 |  | 2,060.41 | 2,049.52 | 1.01 |  | 579.51 | 646.24 | 0.90 |
| 2003 | 28.59 | 37.51 | 0.76 |  | 165.12 | 195.35 | 0.85 |  | 605.76 | 706.05 | 0.86 |  | 2,118.71 | 2,115.31 | 1.00 |  | 612.22 | 686.78 | 0.89 |
| 2004 | 28.60 | 37.31 | 0.77 |  | 164.93 | 193.91 | 0.85 |  | 623.43 | 721.70 | 0.86 |  | 2,138.61 | 2,130.56 | 1.00 |  | 628.93 | 699.26 | 0.90 |
| 2005 | 28.62 | 37.40 | 0.77 |  | 165.06 | 192.67 | 0.86 |  | 634.71 | 732.70 | 0.87 |  | 2,154.01 | 2,154.43 | 1.00 |  | 641.75 | 718.39 | 0.89 |
| 2006 | 28.80 | 37.93 | 0.76 |  | 165.05 | 191.40 | 0.86 |  | 635.84 | 730.74 | 0.87 |  | 2,152.44 | 2,161.32 | 1.00 |  | 656.07 | 735.45 | 0.89 |
| 2007 | 29.38 | 38.83 | 0.76 |  | 166.97 | 192.19 | 0.87 |  | 637.50 | 728.50 | 0.88 |  | 2,171.50 | 2,178.05 | 1.00 |  | 669.95 | 760.47 | 0.88 |
| 2008 | 30.05 | 39.86 | 0.75 |  | 168.48 | 192.24 | 0.88 |  | 643.30 | 727.14 | 0.88 |  | 2,189.63 | 2,193.33 | 1.00 |  | 678.84 | 776.42 | 0.87 |
| 2009 | 30.68 | 41.00 | 0.75 |  | 170.40 | 192.96 | 0.88 |  | 647.86 | 727.54 | 0.89 |  | 2,190.06 | 2,180.24 | 1.00 |  | 681.05 | 781.81 | 0.87 |
| 2010 | 31.31 | 41.89 | 0.75 |  | 171.24 | 193.07 | 0.89 |  | 650.70 | 725.52 | 0.90 |  | 2,185.82 | 2,164.26 | 1.01 |  | 682.72 | 778.12 | 0.88 |
| 2011 | 31.67 | 43.33 | 0.73 |  | 170.55 | 193.94 | 0.88 |  | 641.61 | 717.85 | 0.89 |  | 2,164.80 | 2,146.23 | 1.01 |  | 674.51 | 769.53 | 0.88 |
| 2012 | 31.94 | 45.62 | 0.70 |  | 168.03 | 195.05 | 0.86 |  | 619.62 | 703.22 | 0.88 |  | 2,138.16 | 2,109.11 | 1.01 |  | 663.16 | 751.77 | 0.88 |
| 2013 | 32.70 | 48.89 | 0.67 |  | 166.41 | 197.60 | 0.84 |  | 599.45 | 690.01 | 0.87 |  | 2,103.62 | 2,076.90 | 1.01 |  | 650.47 | 729.65 | 0.89 |
| 2014 | 33.70 | 51.90 | 0.65 |  | 166.12 | 200.91 | 0.83 |  | 585.06 | 677.83 | 0.86 |  | 2,067.70 | 2,053.61 | 1.01 |  | 636.10 | 697.10 | 0.91 |
| 2015 | 34.95 | 54.28 | 0.64 |  | 167.94 | 204.18 | 0.82 |  | 580.17 | 675.03 | 0.86 |  | 2,043.24 | 2,042.96 | 1.00 |  | 638.09 | 684.71 | 0.93 |
| 2016 | 38.63 | 55.27 | 0.70 |  | 180.11 | 205.65 | 0.88 |  | 602.67 | 663.85 | 0.91 |  | 2,057.05 | 2,025.30 | 1.02 |  | 656.26 | 683.81 | 0.96 |
| 2017 | 43.71 | 56.26 | 0.78 |  | 193.86 | 206.86 | 0.94 |  | 626.16 | 653.18 | 0.96 |  | 2,069.84 | 2,018.70 | 1.03 |  | 677.93 | 684.92 | 0.99 |
| 2018 | 44.75 | 56.03 | 0.80 |  | 194.26 | 205.51 | 0.95 |  | 620.74 | 647.00 | 0.96 |  | 2,063.52 | 2,018.43 | 1.02 |  | 678.98 | 680.91 | 1.00 |
| 2019 | 45.49 | 54.69 | 0.83 |  | 195.18 | 205.30 | 0.95 |  | 618.62 | 645.57 | 0.96 |  | 2,082.24 | 2,027.51 | 1.03 |  | 681.10 | 676.04 | 1.01 |
| High SDI |  |  |  |  |  |  |  |  |  |  |  |  |  |  |  |  |  |  |  |
| 1990 | 23.61 | 43.72 | 0.54 |  | 148.30 | 205.13 | 0.72 |  | 480.76 | 610.87 | 0.79 |  | 1,449.72 | 1,793.50 | 0.81 |  | 389.49 | 534.77 | 0.73 |
| 1991 | 20.67 | 41.35 | 0.50 |  | 139.64 | 199.14 | 0.70 |  | 481.48 | 612.48 | 0.79 |  | 1,471.98 | 1,796.35 | 0.82 |  | 395.68 | 529.44 | 0.75 |
| 1992 | 19.08 | 39.58 | 0.48 |  | 133.10 | 193.94 | 0.69 |  | 485.07 | 614.32 | 0.79 |  | 1,500.10 | 1,803.32 | 0.83 |  | 398.83 | 523.44 | 0.76 |
| 1993 | 18.35 | 38.64 | 0.47 |  | 129.82 | 191.61 | 0.68 |  | 493.65 | 621.16 | 0.79 |  | 1,543.67 | 1,831.90 | 0.84 |  | 406.34 | 525.10 | 0.77 |
| 1994 | 18.20 | 38.16 | 0.48 |  | 128.13 | 190.19 | 0.67 |  | 500.40 | 623.26 | 0.80 |  | 1,574.75 | 1,841.29 | 0.86 |  | 410.51 | 521.25 | 0.79 |
| 1995 | 18.39 | 38.39 | 0.48 |  | 128.18 | 191.07 | 0.67 |  | 507.66 | 626.44 | 0.81 |  | 1,607.82 | 1,856.41 | 0.87 |  | 413.42 | 517.78 | 0.80 |
| 1996 | 18.81 | 38.92 | 0.48 |  | 128.64 | 192.16 | 0.67 |  | 514.45 | 627.13 | 0.82 |  | 1,635.79 | 1,870.93 | 0.87 |  | 417.26 | 519.44 | 0.80 |
| 1997 | 19.47 | 40.03 | 0.49 |  | 130.07 | 193.59 | 0.67 |  | 524.08 | 627.87 | 0.83 |  | 1,666.60 | 1,885.78 | 0.88 |  | 423.19 | 521.09 | 0.81 |
| 1998 | 20.33 | 41.46 | 0.49 |  | 133.39 | 195.74 | 0.68 |  | 540.07 | 632.27 | 0.85 |  | 1,719.04 | 1,911.07 | 0.90 |  | 430.70 | 521.99 | 0.83 |
| 1999 | 21.26 | 42.81 | 0.50 |  | 136.95 | 198.37 | 0.69 |  | 554.73 | 638.73 | 0.87 |  | 1,771.10 | 1,949.81 | 0.91 |  | 439.64 | 531.87 | 0.83 |
| 2000 | 22.10 | 44.14 | 0.50 |  | 139.80 | 201.55 | 0.69 |  | 562.23 | 638.26 | 0.88 |  | 1,799.71 | 1,964.46 | 0.92 |  | 447.81 | 536.89 | 0.83 |
| 2001 | 22.97 | 46.04 | 0.50 |  | 142.51 | 204.90 | 0.70 |  | 563.10 | 634.91 | 0.89 |  | 1,807.90 | 1,972.47 | 0.92 |  | 453.36 | 542.56 | 0.84 |
| 2002 | 24.67 | 49.14 | 0.50 |  | 147.07 | 209.06 | 0.70 |  | 563.66 | 632.67 | 0.89 |  | 1,806.37 | 1,978.31 | 0.91 |  | 454.57 | 546.50 | 0.83 |
| 2003 | 27.11 | 52.92 | 0.51 |  | 153.16 | 214.33 | 0.71 |  | 562.80 | 630.86 | 0.89 |  | 1,784.47 | 1,967.75 | 0.91 |  | 452.77 | 549.70 | 0.82 |
| 2004 | 29.75 | 56.54 | 0.53 |  | 158.40 | 217.84 | 0.73 |  | 558.09 | 624.68 | 0.89 |  | 1,748.11 | 1,934.06 | 0.90 |  | 448.63 | 551.11 | 0.81 |
| 2005 | 31.95 | 59.64 | 0.54 |  | 162.92 | 221.84 | 0.73 |  | 558.84 | 623.47 | 0.90 |  | 1,747.81 | 1,927.61 | 0.91 |  | 455.94 | 561.48 | 0.81 |
| 2006 | 33.38 | 61.82 | 0.54 |  | 164.91 | 224.82 | 0.73 |  | 558.19 | 621.33 | 0.90 |  | 1,751.03 | 1,917.48 | 0.91 |  | 463.41 | 567.48 | 0.82 |
| 2007 | 35.15 | 64.11 | 0.55 |  | 168.00 | 229.47 | 0.73 |  | 561.17 | 624.62 | 0.90 |  | 1,769.25 | 1,922.21 | 0.92 |  | 475.27 | 573.32 | 0.83 |
| 2008 | 37.30 | 66.58 | 0.56 |  | 171.40 | 234.91 | 0.73 |  | 567.06 | 629.96 | 0.90 |  | 1,792.09 | 1,928.83 | 0.93 |  | 488.16 | 576.43 | 0.85 |
| 2009 | 39.51 | 68.98 | 0.57 |  | 175.32 | 240.40 | 0.73 |  | 571.27 | 632.58 | 0.90 |  | 1,796.98 | 1,920.42 | 0.94 |  | 494.88 | 566.87 | 0.87 |
| 2010 | 41.45 | 71.01 | 0.58 |  | 178.39 | 242.83 | 0.73 |  | 571.77 | 630.10 | 0.91 |  | 1,788.74 | 1,900.05 | 0.94 |  | 503.35 | 562.20 | 0.90 |
| 2011 | 43.05 | 72.79 | 0.59 |  | 179.37 | 243.17 | 0.74 |  | 562.82 | 621.93 | 0.90 |  | 1,764.55 | 1,877.75 | 0.94 |  | 506.17 | 559.47 | 0.90 |
| 2012 | 44.83 | 74.95 | 0.60 |  | 178.67 | 241.87 | 0.74 |  | 546.65 | 609.70 | 0.90 |  | 1,728.96 | 1,855.80 | 0.93 |  | 504.31 | 558.31 | 0.90 |
| 2013 | 47.15 | 77.65 | 0.61 |  | 179.19 | 240.98 | 0.74 |  | 531.80 | 598.15 | 0.89 |  | 1,683.45 | 1,824.61 | 0.92 |  | 496.67 | 552.67 | 0.90 |
| 2014 | 49.69 | 80.55 | 0.62 |  | 181.09 | 240.97 | 0.75 |  | 521.58 | 588.69 | 0.89 |  | 1,639.06 | 1,792.00 | 0.91 |  | 485.17 | 552.69 | 0.88 |
| 2015 | 52.23 | 82.98 | 0.63 |  | 184.60 | 241.69 | 0.76 |  | 519.16 | 585.10 | 0.89 |  | 1,614.72 | 1,774.79 | 0.91 |  | 474.63 | 552.11 | 0.86 |
| 2016 | 57.45 | 87.64 | 0.66 |  | 196.80 | 249.59 | 0.79 |  | 538.26 | 596.63 | 0.90 |  | 1,631.37 | 1,754.23 | 0.93 |  | 475.54 | 547.27 | 0.87 |
| 2017 | 63.96 | 93.51 | 0.68 |  | 217.09 | 261.78 | 0.83 |  | 572.34 | 615.10 | 0.93 |  | 1,663.84 | 1,737.37 | 0.96 |  | 475.65 | 538.00 | 0.88 |
| 2018 | 67.21 | 97.01 | 0.69 |  | 226.47 | 267.81 | 0.85 |  | 573.16 | 622.61 | 0.92 |  | 1,646.21 | 1,737.20 | 0.95 |  | 464.60 | 532.92 | 0.87 |
| 2019 | 72.13 | 101.71 | 0.71 |  | 240.04 | 279.37 | 0.86 |  | 575.00 | 636.09 | 0.90 |  | 1,635.13 | 1,752.72 | 0.93 |  | 456.99 | 530.47 | 0.86 |
